# Protein Sequence Analysis landscape: A Systematic Review of Task Types, Databases, Datasets, Word Embeddings Methods, and Language Models

**DOI:** 10.1093/database/baaf027

**Published:** 2025-05-30

**Authors:** Muhammad Nabeel Asim, Tayyaba Asif, Faiza Hassan, Andreas Dengel

**Affiliations:** German Research Center for Artificial Intelligence, Kaiserslautern 67663, Germany; Intelligentx GmbH (intelligentx.com), Kaiserslautern, Germany; Department of Computer Science, Rheinland Pfälzische Technische Universität, Kaiserslautern 67663, Germany; Department of Computer Science, Rheinland Pfälzische Technische Universität, Kaiserslautern 67663, Germany; German Research Center for Artificial Intelligence, Kaiserslautern 67663, Germany; Department of Computer Science, Rheinland Pfälzische Technische Universität, Kaiserslautern 67663, Germany; Intelligentx GmbH (intelligentx.com), Kaiserslautern, Germany

## Abstract

Protein sequence analysis examines the order of amino acids within protein sequences to unlock diverse types of a wealth of knowledge about biological processes and genetic disorders. It helps in forecasting disease susceptibility by finding unique protein signatures, or biomarkers that are linked to particular disease states. Protein Sequence analysis through wet-lab experiments is expensive, time-consuming and error prone. To facilitate large-scale proteomics sequence analysis, the biological community is striving for utilizing AI competence for transitioning from wet-lab to computer aided applications. However, Proteomics and AI are two distinct fields and development of AI-driven protein sequence analysis applications requires knowledge of both domains. To bridge the gap between both fields, various review articles have been written. However, these articles focus revolves around few individual tasks or specific applications rather than providing a comprehensive overview about wide tasks and applications. Following the need of a comprehensive literature that presents a holistic view of wide array of tasks and applications, contributions of this manuscript are manifold: It bridges the gap between Proteomics and AI fields by presenting a comprehensive array of AI-driven applications for 63 distinct protein sequence analysis tasks. It equips AI researchers by facilitating biological foundations of 63 protein sequence analysis tasks. It enhances development of AI-driven protein sequence analysis applications by providing comprehensive details of 68 protein databases. It presents a rich data landscape, encompassing 627 benchmark datasets of 63 diverse protein sequence analysis tasks. It highlights the utilization of 25 unique word embedding methods and 13 language models in AI-driven protein sequence analysis applications. It accelerates the development of AI-driven applications by facilitating current state-of-the-art performances across 63 protein sequence analysis tasks.

## Introduction

Protein sequence analysis is a scientific way to utilize diverse types of strategies for examining the order of amino acids within protein sequences. This analysis objective is to unlock diverse types of a wealth of knowledge about biological processes and genetic disorders (1). Researchers are gaining deep understanding about biological processes in which proteins are involved, such as enzyme activity (2), cell signalling (3), and immune responses (4). Researchers are also gaining understanding about genetic disorders by pinpointing mutations that alter proteins functionalities (5). It helps in forecasting diseases susceptibility by finding unique protein signatures, or biomarkers that are linked to particular disease states (6). Specifically, this analysis enables researchers to identify individuals at higher risk for developing certain diseases before symptoms even appear. Protein sequence analysis process through wet-lab experiments requires significant costs due to involvement of specialized chemicals and equipment (7). The process demands extensive time commitments due to the necessity of lengthy protocols and extended periods for biological growth (7). Additionally, even the most skilled scientists can introduce errors when conducting large-scale experiments, whether in adhering to protocols, selecting appropriate chemicals, or managing external conditions such as temperature and equipment sanitation (7). These factors collectively contribute to the high expense, time consumption, and potential for error in traditional wet-lab protein sequence analysis (7).

Advancements in next-generation sequencing technologies have generated an enormous volume of protein sequence data that is accessible in public databases (8). The vast availability of publicly accessible data has enable large-scale protein sequence analysis by shifting from traditional wet lab experimental methods to AI-driven protein sequence analysis applications (9). Despite noteworthy achievements in development of AI-driven protein sequence analysis applications, there remains a significant room for further development of more powerful AI-driven applications (10). A primary reason behind sub-optimal predictive performance of AI-driven applications is their limited ability to effectively extract meaningful patterns from proteins sequences that are made up from repetitive patterns of 20 unique amino acids (11). Specifically, the repetitive patterns of amino acids within protein sequences encompasses a wealth of information such as protein modifications (12–22), sub-cellular localization (23–34), protein–protein interactions (35–46), and protein–virus interactions (47–52). To provide a high level overview about working paradigm of AI-driven protein sequence analysis applications across various tasks, these applications can be broadly categorized into three distinct classes: classification (13, 53–60), regression (61–64), and clustering (65). Classification applications objective is to assign protein sequences into predefined classes based on specific patterns of amino acids within protein sequences (54–57, 66). Regression applications objective is to predict a continuous numerical value based on specific patterns of amino acids within protein sequences (61–64, 66). Clustering applications groups similar protein sequences together into clusters or make groups based on their inherent similarities in sequences (66).

**Figure 1.**
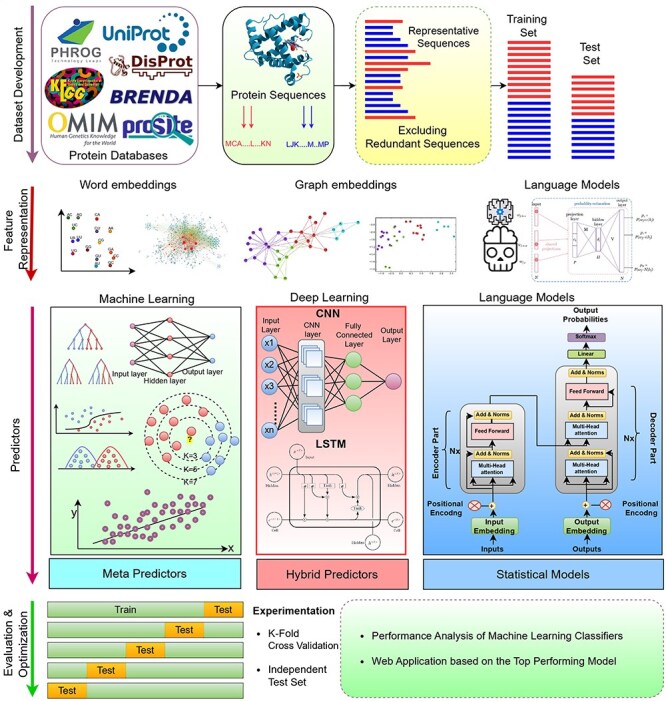
Artificial Intelligence Driven Predictive Framework for Analyzing Protein Sequences Across Diverse Bioinformatics Tasks.

Figure 1 graphically represents a generalized AI-driven protein sequence analysis pipeline for diverse types of tasks that fall under three fundamental AI paradigms: classification, regression, and clustering. A high-level examination of Figure 1 reveals that the initial step in development of AI-driven protein sequence analysis application requires benchmark dataset. This dataset is usually developed by acquiring protein sequences and corresponding biological information from two primary sources: wet-lab experiments (7), public databases (67). In the next step, raw protein sequences are transformed into statistical vectors because AI algorithms have inherent dependency over statistical vectors. AI algorithms are then trained using a training set comprising of protein sequences statistical vectors along with associated numerical values or predefined classes. The trained models are subsequently evaluated on a test set by comparing their predicted labels with actual labels. Finally, a web application is developed to utilize the trained models for practical use.

The overall performance of AI-driven protein sequence analysis predictive pipelines is entirely dependent on the quality of the statistical vectors used to represent protein sequences (68). When these vectors effectively capture informative patterns of amino acids in the protein sequences, simple machine learning algorithms can achieve good performance (68). Conversely, complex algorithms may underperform if presented with random statistical vectors lacking these informative patterns (69). To convert protein sequences into statistical vectors by extracting and encoding various amino acid patterns, researchers have developed over 100 encoding methods (41). These methods either capture the positional information of amino acids within protein sequences or utilize the physicochemical properties of amino acids to capture covariance and correlation information. Considering the similarities between protein sequences and textual data, and following the success of word embedding methods and LLMs in capturing and encoding diverse patterns into statistical vectors for various NLP tasks, researchers are harnessing these methods for development of AI-driven protein sequence analysis applications. AI-driven protein sequence analysis realm has witnessed many review articles focused on exploration of domain-specific encoding methods. However, comprehensive literature on utilization of word embedding methods and LLMs is not available. A thorough review of these methods would significantly benefit the research community by highlighting the potential of these powerful NLP methods in development of AI-driven protein sequence analysis applications. With an aim to explore protein sequence analysis realm at large scale and to present integration of word embedding methods and LLMs into AI-driven protein sequence analysis applications for transformative discoveries the contributions of this manuscript are manifold:

- It bridges the knowledge gap between Proteomics and Artificial Intelligence fields. Proteomics scientists can utilize this review article to gain insights about AI potential in the realm of protein sequence analysis, while AI researchers can gain a deeper understanding about protein sequence analysis tasks biological foundations, challenges and opportunities for development of AI-driven protein sequence analysis applications.
- It equips AI researchers with a foundational understanding of 63 distinct protein sequence analysis tasks. To provide a comprehensive overview of proteomic sequence analysis landscape, we categorised 63 tasks into 11 major goals namely Protein Identification, Properties Prediction, function and structure prediction, Modification prediction, Interaction Prediction, Sub-cellular location prediction, Peptide Analysis, Gene Analysis, Mutation Analysis, Disease Analysis, and Drug Analysis.
- It enhances development of AI-driven protein sequence analysis applications by providing cruxes of 68 different biological databases that have been utilized for development of 63 distinct tasks related benchmark datasets. A comprehensive knowledge of these databases can be utilized to develop new benchmark datasets.
- It explains the nature of 63 unique Protein sequence analysis tasks and classify them into 2 primary categories: regression, classification, as well as three secondary categories: binary classification, multi-class classification, and multi-label classification. Protein sequence analysis tasks mapping into core AI tasks will gain interest of AI researchers for development of more effective and specialized AI-driven protein sequence analysis applications at large scale.
- To ensure a fair performance comparison between existing and new AI predictors, it provides the details of 627 benchmark datasets related to 63 unique protein sequence analysis tasks.
- It demonstrates the utilization of 25 unique word embedding methods and 13 language models in AI-driven protein sequence analysis applications for 63 unique protein sequence analysis tasks.
- To facilitate development of new predictors, this review provides a detailed summary of current state-of-the-art predictors, their performances across 63 unique protein sequence analysis tasks, and their availability to scientific community.

## Research methodology

**Figure 2.**
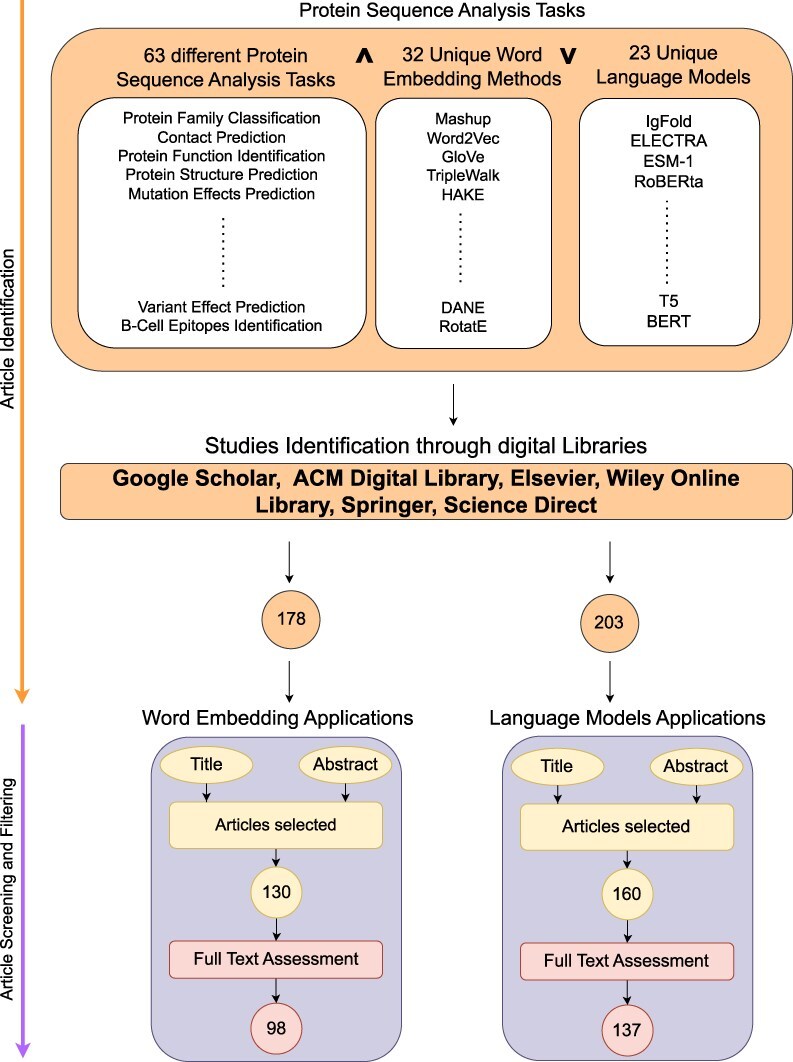
Research methodology.

Figure 2 illustrates a two stage process for search and selection of most relevant articles related to word embeddings and language models applications in the realm of protein sequence analysis. Following sub-sections summarize the details of two stages: (1) article searching and (2) article screening and filtering.

### Article searching

This stage formulates quality search queries to search articles related to word embeddings and language models applications in proteins sequence analysis landscape. Figure 2 demonstrates that article identification module comprises of three different keywords cells namely; Protein analysis tasks, word embedding methods and Language models.Our search encompasses articles published from 2016 to September 2024, a period that captures the evolution of word embeddings and language models in protein sequence analysis. Given the dynamic nature of this field, particularly regarding language models, and the broader scope of this review covering 63 distinct tasks, it is impractical to include all papers that emerge on a regular basis. We have explicitly mentioned this timeline to help readers understand the temporal scope of our analysis and effectively drive the research forward. To design search queries, keywords within the same cell are combined using OR $\lor$ operator while keywords across different cells are concatenated using AND $\land$ operator. For instance sample search queries are protein family classification using FastText word embedding, protein–protein interaction prediction using BERT language model, etc. These search queries are executed on distinct search engines including Google Scholar (https://scholar.google.com/), ACM Digital Library (https://dl.acm.org/), IEEEXplore (https://ieeexplore.ieee.org/), Elsevier (https://www.elsevier.com/), Wiley Online Library (https://www.wiley.com/en-us), Springer (https://www.springer.com/gp) and ScienceDirect (https://www.sciencedirect.com/). Furthermore, snowballing is employed to investigate articles cited in extracted papers to identify more research articles. Execution of queries across multiple academic databases acquired 178 word embedding and 203 language models based research articles which are screened and filtered in second stage.

### Article screening and filtering

In second phase, most relevant articles are selected through a two-step process. Firstly, titles and abstracts of 559 word embedding and language models based articles are reviewed by domain experts. This review yielded 130 word embedding and 160 language models based relevant articles. Subsequent, full-text assessment of these articles identified 98 articles focused on word embedding and 137 articles relevant to language models in protein sequence analysis.

## Biological foundations of protein sequence analysis goals and tasks

**Figure 3.**
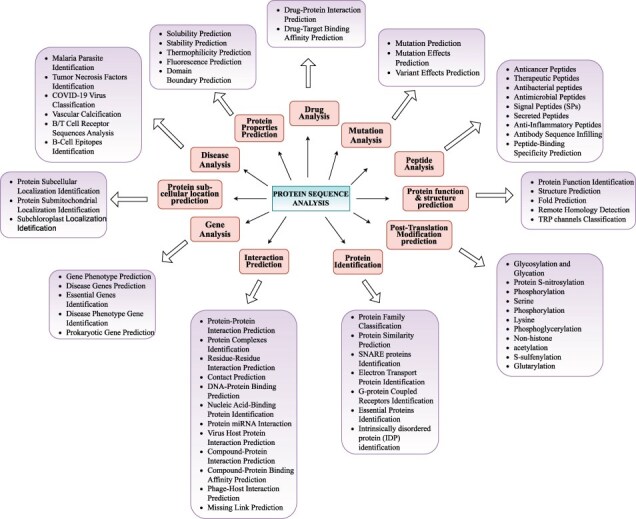
Precise classification of unique protein sequence analysis tasks in 11 major biological goals.

Proteins sequences comprise repetitive patterns of 20 unique amino acids whose arrangements represent diverse types of information such as protein’s structure, function, and interactions. Irregularities or mutations in these arrangements can lead to various biological disorders and diseases such as cystic fibrosis, sickle cell anaemia, Huntington’s disease, Tay-Sachs disease, and different forms of cancer. With an aim to understand roles of proteins in diverse types of biological functions, and their associations with genetic disorders and diseases, researchers are exploring the realm of proteins from various perspectives. We have categorized 63 distinct protein sequence analysis tasks into 11 distinct biological goals namely Protein Identification, Properties Prediction, function and structure prediction, Modification prediction, Interaction Prediction, Sub-cellular location prediction, Peptide Analysis, Gene Analysis, Mutation Analysis, Disease Analysis, and Drug Analysis. A graphical illustration of all 11 goals and their associated tasks is shown in Figure 3. Living organisms contain millions of proteins in simple cells and billions in complex organisms. To thoroughly explore the distinct functionalities and properties of proteins considering the fact proteins within the same family share similar characteristics, scientists study them at family level rather than individually. This family-level exploration and analysis require proteins classification into various families such as kinases phosphatases, G-protein coupled receptors, immunoglobulins heat shock proteins, cytochromes proteases, transcription factors, transporters, and structural proteins. Protein family classification facilitates valuable clues about the structure and function of uncharacteristic proteins in the cell on the basis of known structures and functions of family members. Furthermore researchers are identifying proteins with unique functionalities and properties such as identification of essential proteins, SNARE proteins, electron transport proteins, G-protein coupled receptors, and intrinsically disordered proteins. These proteins actively participate in fundamental biological processes and pathways where their dysfunction can severely hamper proper functioning of living organisms.

Within protein identification landscape, protein similarity prediction groups proteins into clusters based on the distribution of amino acids in their sequences. This approach aids in discovering new classes of proteins with unique characteristics. Moreover protein similarity prediction facilitates the annotation of newly sequenced proteins by inferring functions based on similarity to known proteins thereby accelerating the discovery of biological pathways and processes. While traditional methods like profile searching have been effective for protein annotation and similarity prediction, AI approaches have become increasingly critical due to several factors. The exponential growth in protein sequence databases has made manual and traditional computational analyses impractical. AI methods can handle this scale while detecting subtle patterns in amino acid distributions that might be missed by conventional algorithms. Additionally, AI’s ability to integrate multiple features simultaneously, from sequence patterns to evolutionary information, enables more accurate predictions, especially for proteins with low sequence similarity to known groups. These advantages are particularly valuable when dealing with novel proteins or those from understudied organisms, where traditional profile-based methods might fail to detect important functional relationships. For the pharmaceutical industry, protein similarity prediction is invaluable in drug discovery and development where identifying proteins similar to known drug targets can reveal new therapeutic opportunities. Protein properties prediction encompasses various characteristics including solubility, stability, thermophilicity, fluorescence, and domain boundaries which provide crucial insights into protein behaviour and potential applications. Protein solubility refers to the ability of a protein to dissolve and remain in solution without aggregating or precipitating out where various factors such as amino acid composition hydrophobicity and interactions of different molecules within cellular environment influence this property. Solubility prediction assists scientists to identify proteins that are more prone to misfold or aggregate to enable them to comprehend underlying mechanisms of complex diseases and develop potential interventions. Protein stability and thermophilicity predictions facilitate researchers to engineer proteins with improved stability for various applications such as enzymes for industrial processes, therapeutic proteins or high-temperature environments. Protein fluorescence prediction aids in studying protein interactions and designing biosensors while domain boundaries prediction helps in understanding the modular nature of proteins protein engineering and functional annotation.

Function and structure prediction focuses on determining proteins roles and three-dimensional conformations which are essential for understanding their behaviour in cellular environments. Within cellular environment proteins act as essential workhorses where each protein possesses a unique function such as enzyme activity or structural support and structure such as *α*-helix, *β*-strand, and turns. Proteins functions hold valuable information about biological activities such as catalysing biochemical reactions providing structural support and facilitating cellular communication and transport. Structure prediction reveals how a protein might interact with other molecules where comprehensive information about function and structure of a protein is useful for understanding cellular machines working paradigm in cellular environment. Remote homology detection and fold prediction further enhance our understanding of protein evolution and potential functional relationships. The protein interactions landscape offers valuable insights about how proteins work together within biological systems interactions role in cellular communication and how irregularities in interactions contribute to disease mechanisms and affect biological processes. Protein interactions are the cornerstone of nearly all cellular processes because they mediate signalling pathways genetic expression and cellular machinery functions. The landscape includes interactions between proteins and various molecules including DNA/genes (70), viruses, RNAs (71), and compounds. Each type of interaction prediction yields unique insights. Like protein–gene interactions are pivotal in understanding gene regulation and expression process, protein–protein interactions enable understanding of proteins dynamic roles in various biological processes, and virus-host protein interactions shed light on how viruses hijack host’s cellular machinery.

Protein modification prediction is important for understanding protein’s functional landscape including its stability and activity in various biological processes and diseases. Proteins undergo diverse kinds of post-translational modifications including methylation glycosylation acetylation phosphorylation and ubiquitination which modify various properties of proteins such as their structure electrophilicity and interactive capacity that enable them to take part and regulate variety of cellular processes. With over 200 diverse types of post-translational modifications, researchers are putting efforts to identify S-sulfenylation and glutarylation sites in proteins which play key roles in signal transduction regulation of protein activity function and interactions in cellular environment. Scientists are performing protein sub-cellular localization prediction to gain insights into proteins roles in different cellular compartments. Primarily proteins core biological activities are strictly linked with their presence in different cellular compartments such as cell junction, cell membrane, cell projection, cytoplasm, Golgi apparatus, lysosome, mitochondrion, nucleus, secreted endoplasmic reticulum, plastid extracellular signal chloroplast lysosome/vacuole and peroxisome. Within a cell different compartments provide distinct microenvironments where proteins perform specialized functions such as metabolism related activities in mitochondria and DNA replication process in nucleus. Proteins are built from small building blocks called peptides which researchers have categorized based on diverse types of properties like stability bio-availability efficacy action mechanisms involvement in cellular processes utilization in drugs and therapies. The most well-characterized and diverse peptide types include anticancer peptides antibacterial peptides antimicrobial peptides signal peptides secreted Peptides and anti-inflammatory peptides. Each peptide type offers unique therapeutic potential where anti-inflammatory peptides modulate immune system responses anti-cancer peptides target cancer cells through various mechanisms and antibacterial peptides provide alternatives to traditional antibiotics. In the realm of disease analysis protein-centric investigations focus on parasite identification characterization of immune factors viral classification and analysis of protein sequences associated with specific pathological conditions. Researchers are identifying compounds that prevent or reverse disease progression by targeting responsible pathways. Furthermore identification of disease related proteins compounds and inhibitors are accelerating drug repurposing which offers rapid response to emerging diseases while providing immediate treatment options. Gene analysis primarily focuses on gene phenotype prediction disease genes prediction essential genes identification and prokaryotic gene prediction. These genes often lead to chronic diseases such as sickle cell anaemia multiple sclerosis Huntington’s disease type 2 diabetes heart disease and many forms of cancer. Understanding gene phenotypes and essential genes is fundamental for advancing medical research and developing targeted therapies. Mutation analysis encompasses prediction of mutations their effects and variant impacts providing insights into disease mechanisms and treatment responses. This knowledge empowers researchers to elucidate molecular mechanisms of disease and develop targeted therapeutic strategies. Within drug analysis landscape protein–drug interaction and binding affinity prediction streamlines drug development process and facilitates development of personalized medicine approaches.

## A look on protein sequence analysis tasks from the perspective of computer scientists

With rapid advent of AI technologies and biological data growth, researchers are increasingly applying AI methods to various areas of genetics biology. Development of large-scale AI applications requires an in-depth understanding of a wide range of sequence analysis tasks. Genetics biologists understand importance, biological relevance, and pharmaceutical potential of different protein sequence analysis tasks, but they often struggle to select the most suitable machine learning or deep learning models to enhance or replace experimental approaches. Alternatively, computer scientists excel in identifying most appropriate AI-driven predictive pipelines that are most likely to deliver optimal results for specific data types but they may find it challenging to fully grasp complexities of protein sequence analysis tasks. Thus, there is a significant gap between the expertise of computer scientists and genetics biologists. For instance, protein function prediction, protein similarity prediction or mutation effects prediction can be challenging to understand immediately. A detailed analysis of existing literature can greatly help bridge this gap by explaining the fundamentals of such tasks. For instance, protein function prediction initially seems like a multi-class classification task, but it is actually a multi-label classification task. Similarly, protein similarity prediction appears to be a regression task, but it is actually a clustering task and mutation effects prediction seems to be a multi-label classification task but it is actually a multi-class classification. With this core understanding, computer scientists can more precisely develop predictive pipelines tailored to binary, multi-class, multi-label classification, regression and clustering task.

**Figure 4.**
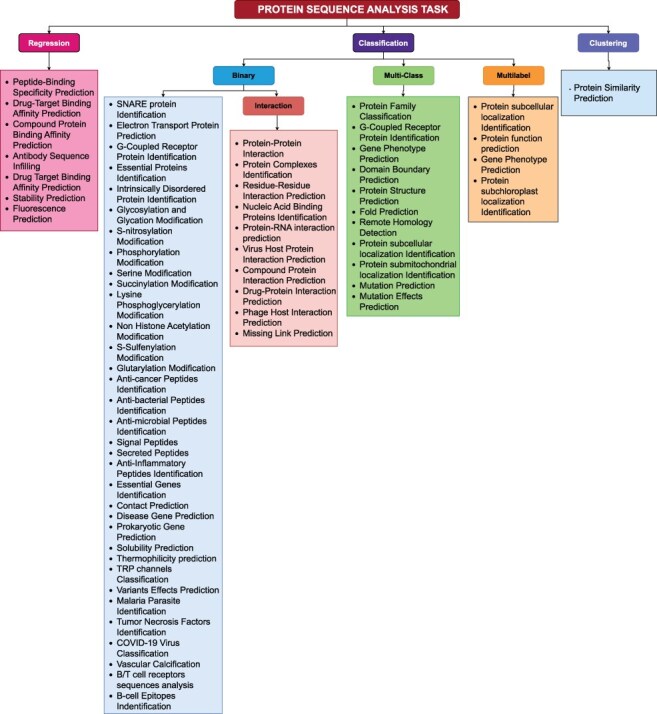
A comprehensive methodical categorization of protein sequence analysis tasks into regression, binary classification, multi-class classification, multi-label classification, and clustering.

To facilitate research community, we have systematically categorized 63 protein sequence analysis tasks based on their nature as illustrated in Figure 4. A high-level overview of Figure 4 reveals that protein sequence analysis tasks can be broadly categorized into 3 primary types: (1) Regression, (2) classification, (3) Clustering. Classification tasks can further be divided into three secondary types: (1) Binary Classification, (2) Multi-class Classification, (3) Multi-label Classification. This section delves into mathematical formulations of unique types of protein sequence analysis tasks.

In binary classification, the primary goal is to predict the outcome of a binary variable (0 or 1). Given a dataset with features $X_i \in \mathbb{R}^{n \times d}$, binary labels $Y_i \in \{0, 1\}$, and a training set $(X_1, Y_1), (X_2, Y_2), \dots$, the main objective is to learn a decision function $f(x): X_i \rightarrow Y_i$ that maps inputs to binary outcomes $\{0, 1\}$ using the hypothesis function $h(X_i)$ derived from the training data. Equation 1 illustrates mathematical expression for decision function *f*(*x*).


(1) $$ f(x)= \begin{cases}1 & if\,h(X_i)\geqslant0.5\\0 & otherwise.\end{cases} $$

Multi-class classification predicts outcome from more than two classes. In a dataset with features $X \in \mathbb{R}^{n \times d}$, labels $y \in {1, 2, \dots, n}$, where *n* indicates total number of classes, and a training dataset $(x_1, y_1), (x_2, y_2), \dots, (x_k, y_k)$ where $x_i \in X$ and $y_i \in Y$, objective is to develop a decision function $f(x): X \rightarrow Y$ that assigns inputs to one of the available classes. Equation 2 depicts mathematical expression for decision function *f*(*x*) in multi-class classification.


(2) $$ f(x)=argmax_n h_n(x). $$

In multi-label classification, each input may be associated with multiple classes simultaneously. For instance, in a dataset with features $X \in \mathbb{R}^{n \times d}$, labels $y \in {1, 2, \dots, n}$ where *n* denotes number of classes, and a training dataset $(x_1, y_1, y_2, \dots), (x_2, y_1, y_4, \dots), \dots, (x_k, y_5, y_k, \dots)$ where $x_i \in X$ and $y_i \in Y$, main objective is to construct a decision function $f(x): X \rightarrow {0, 1}^K$ that assigns inputs to multiple classes simultaneously using hypothesis function $h_n(x)$ for class *n* derived from training data. Equation 3 represents mathematical expression for decision function *f*(*x*) used in multi-class classification.


(3) $$ f(x)=(h_1(x),h_2(x),...,h_n(x)). $$

Moreover, prime objective in regression is to predict a continuous outcome variable. Specifically in regression, for a dataset with features $X \in \mathbb{R}^{n \times d}$, labels $y \in \mathbb{R}$, and training dataset $(x_1, y_1), (x_2, y_2), \dots, (x_k, y_k)$ where $x_i \in X$ and $y_i \in Y$, aim is to learn a function $f: X \rightarrow \mathbb{R}$ that predicts continuous outputs using hypothesis function *h*(*x*). Equation 4 depicts mathematical expression for hypothesis function *h*(*x*) learned from the training data.


(4) $$ f(x)=h(x). $$

In clustering, objective is to group similar data points into relevant clusters. Given a dataset of data points $X = {x_1, x_2, \dots, x_k}$, where each $x_i \in \mathbb{R}^d$, main goal is to assign a clusters $C = {C_1, C_2, \dots, C_n}$ to each data point by using a distance metric $d(x, \mu_c)$. Distance metric determines distance between a data point *x* and centroid *µ*_*c*_ of cluster *c*. Equation 11 illustrates mathematical expression of function *f*(*x*) to estimate distance.


(5) $$ f(x) = \text{argmin}_c d(x, \mu_c). $$

## Protein sequence analysis databases

This section presents a comprehensive survey of protein databases that encompasses essential data for the development of AI-driven applications across 63 diverse protein sequence analysis tasks. It equips AI researchers with essential information required to identify appropriate databases for the development of high-quality benchmark datasets, which are the cornerstone for development of AI-driven protein sequence analysis applications.

In the realm of AI-driven protein sequence analysis, a detailed review of 295 research articles indicates that researchers have harnessed a remarkable diversity of 100 unique protein databases to develop 627 benchmark datasets for 63 protein sequence analysis tasks. To the best of our knowledge, 68 of these databases are currently publicly accessible, while the remaining 32 are either restricted or no longer available. Table 1 presents a valuable road-map for AI researchers to select optimal databases for development of high-quality benchmark datasets. It offers a concise yet informative overview of 68 publicly accessible databases by highlighting their diverse characteristics such as database name, release date, data types, related species and organisms, data statistics, and data formats.

A closer examination of the ‘data type’ feature in Table 1 reveals that: All databases contain protein data and out of the 68 databases, 13 also contain information related to DNA and RNA. These databases include DisGeNET (72), CARD (73), VariBench (74), ClinVar (75), BioLip (76), CCLE (77), NCBI (78), MtSSPdb (79), GEO (80), KEGG (81), PINA (82), EMBL-EBI (83) and OMIM (84). Moreover, 13 databases contain different data types as follows, transcriptomics: MtSSPdb (79), immune repertoires: OAS database (85), genes, mutations and drugs: GeneCards (86), IMGT (87), and COSMIC (88), host proteins: HPIDB (89), gene and diseases: MalaCards (90), molecules, drugs, compounds, and drugs: ChEMBL (91), DUD-E(92), and BindingDB (93), and chemicals: (94), and DUD (95). Moreover, data related to TCR sequences, antigens, immunoglobulins (IGs), T cell epitopes, microbiome and antibodies is available in McPAS-TCR (96), VDJdb (97), PIRD (98), MGnify (99) and IEDB (100) databases. In addition, Negatome database (101) contains domain pairs sequences, PubChem (102) provides compounds strings, genes, and cell lines, CTD (103) houses data related to chemical-gene interaction, chemical-disease interaction, and chemical-phenotype interactions, intAct (104), provides data related to interactions, interactors, and mutations, and enzymes data is available in BRENDA (105).

In Table 1, we performed a detailed analysis of ‘Species’ feature to categorize databases into three classes: (1) Fewer species coverage, (2) Moderate species coverage, (3) Large species coverage. In the category of fewer species coverage, we have included 35 databases housing data for 20 species or fewer. This category databases names are DisProt (106), PHROGs (107), MtSSPdb (79), PPT-Ohmnet (108), COSMIC (88), HPIDB (89), McPAS-TCR (96), VDJdb (97), DisGeNET (72), HIPPIE (109), MalaCards (90), ClinVar (75), BioLip (76), PDB (110), ConSurf-DB (111), dbPTM (112), CCLE (77), STITCH (94), NCBI (78), intAct (104), Therapeutic Targets Databases (113), Phospho.ELM (114), GeneCards (86), KEGG (81), Prosite (115), UniProtKB (116), OMIM (84), OAS database (85), SAbDab (117), Negatome database (101), DUD-E (92), DUD (95), PDBbind database (118), PhosphoSitesPlus (119) and interPro (120). On the other hand, in the moderate species coverage category, we included eight databases encompassing data for a range of 21 to 80 species. These databases include AlphaFoldDB (121), AmyPro (122), MobiDB (123), IPD-MHC (124), CARD (73), BioGRID (125), GEO (80) and ChEMBL (91). In large species coverage category, we included 25 databases encompassing data more than 80 species. This category related databases are MINT database (126), OGEE (127), DIP (128), IMGT (87), STCRDab (129), PIRD (98), Uniclust30 (130), GLASS (131), MGnify (99), SCOPe (132), BindingDB (93), VariBench (74), PINA (82), TCDB (133), PubChem (102), GOA (134), IEDB (100), CTD (103), STRING (72), RCSB PDB (135), SCOP (136), EMBL-EBI (83), GPCRdb (137), CATH (138) and BRENDA (105).

**Table 1.** An overview of publicly available biological databases: data types, species diversity, and raw sequence statistics for genomic and proteomic information

Database name	Release date	Types of data	Species	Organism	Sequences statistics	Data format
AlphaFoldDB	2021	Protein	48 species	_	214,683,839 protein structures	.txt, .csv, .json, FASTA
DisProt	2021	Protein	Viruses, Archaea, Eukaryota	Bacteria	Disorder function: 558 proteins, 874 regions, Structural state: 3,022 proteins, 6,922 regions, Structural transition: 543 proteins, 894 regions, Cellular component: 29 proteins, 54 regions, Biological process: 248 proteins, 531 regions, Molecular function: 1,203 proteins, 3,889 regions	.json, .tsv, GAF, FASTA
PHROGs	2021	Protein	viruses infecting bacteria or Archaea	_	Protein orthologous groups: 38,880, Proteins: 868,340, Prophages: 12,498	.tsv, .csv, .xlsx, .pdf, FASTA, MSA, HMM
MtSSPdb	2020	Protein, Genomics, Transcriptomics	Medicago truncatula, Panicum virgatum, Arabidopsis thaliana	Plant	Re-annotated genes: 70,094, Small Peptides genes: 4,439, Known SSP gene families: 72	FASTA, .gff, .txt, HMM
OAS database	2018	Protein, Immune repertoires	_	Rabbit, Human, Mouse, Rhesus, Camel, Rat	Unpaired sequences: 2,428,016,345 unique sequences, Paired sequences: 2,038,528 filtered sequences	.csv
PPT-Ohmnet	2018	Protein	Homo sapiens	_	Nodes (human proteins): 4510, Edges (tissue specific interactions): 70 338, Nodes in largest SCC: 4488, Edges in largest SCC: 70 316, Number of triangles: 6 698 541	.txt, .edgelist
COSMIC	2018	Protein, Genes, Mutations, Drugs	Homo sapiens	Animal	Total Genomic variants: 24,599,940, Genomic non-coding variants: 16,748,366,406, Genomic mutations within Exons: 768, Genomic mutations within Intronic and other intragenic regions: 9,217,664, Samples: 1,531,613, Fusions: 19,428, Gene expression variants: 9,215,470, Differentially Methylated CpGs: 7,930,489	FASTA, .tsv
AmyPro	2017	Protein	39 species	_	125 amyloid precursor proteins	.txt, .json, FASTA
HPIDB	2017	Protein, Host	11 species	1	9,957 Influenza interactions, 8,174 Herpes viruses interactions, 6,862 Saccharomyces cerevisiae interactions, 6,515 Papillomaviruses interactions, 4,366 Human immunodeficiency virus interactions, 4,026 Yersinia interactions, 3,069 Bacillus interactions, 2,617 Hepatitis C virus interactions, 1,371 Francisella tularensis, 1,030 Measles virus	FASTA
McPAS-TCR	2017	TCR sequences, Protein	Homo sapiens, Mus musculus	_	386 Human TCR*α*, 3,887 Human TCR*β*, 254 Mouse TCR*α*, 1,194 Mouse TCR*β*	.csv
MobiDB	2017	Protein	24 species	_	Total proteins: 219.7M, Total residues: 75.5B	.tsv, .json
STCRDab	2017	Protein	_	_	Number of PDB entries with a TCR structure: 618, Number of *αβ* TCRs: 851, Number of *γδ* TCRs: 18, Number of TCRs complexed to MHC/MHC-like molecules: 680	.csv, .txt
VDJdb	2017	Protein, TCRs Antigens	Homo sapiens, Macaca mulatta, Mus musculus	_	Homo sapiens Chain TRA: Records: 30,937, Paired records: 24,797, Unique epitopes: 943, Homo sapiens Chain TRB: Records: 43,806, Paired records: 25,722, Unique epitopes: 1,131, Macaca mulatta Chain TRA: Records: 74, Paired records: 0, Unique epitopes: 1, Macaca mulatta Chain TRB: Records: 1,290, Paired records: 0, Unique epitopes: 3, Mus musculus Chain TRA: Records: 1,680, Paired records: 1,620, Unique epitopes: 55, Mus musculus Chain TRB: Records: 2,210, Paired records: 1,626, Unique epitopes: 63	.tsv
PIRD	2016	Protein, IGs, TCRs	_	_	11.395 million sequences, and the phenotypes with the top three abundant sequences were 2.539 million in IgA nephropathy project, 1.924 million in minimal residual disease (MRD) project and 1.920 million in healthy samples	.irf
Uniclust30	2016	Protein	_	_	9.7 million clusters, 7 million singletons	.tsv, FASTA
IPD-MHC	2015	Protein	77 species	92 organisms	629 genes, 11,940 alleles	.dat, .txt, .xml, FASTA
DisGeNET	2015	DNA, RNA, Protein	Homo sapiens	Animal	1,134,942 GDAs between 21,671 Genes, 30,170 diseases, and traits, 369,554 VDAs between 194,515 variants and 14,155 diseases and traits	.txt, RDF, SQL Dump
GLASS	2014	Protein	_	_	562,871 unique GPCR-ligand entries, 1,046,026 experimentally data entries, 3,056 GPCR entries, 825 human GPCR, 733 GPCRs that have experimental association data, 342,539 ligand entries, 241,243 Lipinski-druglike ligand	.tsv, .sdf
MGnify	2014	Microbiome, Protein	_	_	Residues: Sequence: 577,410,242,951, Cluster: 131,163,572,133, Total Sequences: 2,973,257,435, Clusters: 729,215,663, Biome: 491	.tsv, FASTA
SAbDab	2014	Protein	_	_	Total number of antibody structures: 8,634, Number of structures with at least one paired VH/VL: 6,947, Number of FV regions: 17,150, Number of structures with antigen: 8,205, Number of antibodies with affinity data: 739	.tsv, .pdb
SCOPe	2014	Protein	_	_	Class: All alpha proteins, Number of folds: 290, Number of superfamilies: 519, Number of families: 1,089, Class: All beta proteins, Number of folds: 180, Number of superfamilies: 375, Number of families: 993, Class: Alpha and beta proteins (a/b), Number of folds: 148, Number of superfamilies: 247, Number of families: 1,003, Class: Alpha and beta proteins (a+b), Number of folds: 396, Number of superfamilies: 580, Number of families: 1,387, Class: Multi-domain proteins (alpha and beta), Number of folds: 74, Number of superfamilies: 74, Number of families: 128, Class: Membrane and cell surface proteins and peptides, Number of folds: 69, Number of superfamilies: 131, Number of families: 204, Class: Small proteins, Number of folds: 100, Number of superfamilies: 141, Number of families: 280, Totals: Number of folds: 1,257, Number of superfamilies: 2,067, Number of families: 5,084	FASTA
MINT database	2013	Protein	674 species	_	Interactions: 139,547, Interactors: 27,756	.mitab
BindingDB	2013	Protein, Compounds	_	_	2,903,069 binding data for 9,319 proteins and over 1,253,918 drug-like molecules	.tsv
CARD	2013	Protein, RNA, DNA, compounds, molecules	40 species	_	377 pathogens, 21,079 chromosomes, 2,662 genomic islands, 41,828 plasmids and 155,606 whole-genome shotgun assemblies, resulting in collation of 322,710 unique ARG allele sequences	.tsv, .json, .gz, .tar, .pdf, .txt, tab, FASTA, OBL, OWL
HIPPIE	2013	Protein	Homo sapiens	1	more than 270,000 confidence scored and annotated PPIs	.txt, .tsv, .json
MalaCards	2013	Protein, Genes, Disease	Homo sapiens	Human	22,960 entries, 15,278 with associated genes, Total disorders: 22,960, Gene-related Disorders: 15,278	_
VariBench	2013	Protein, RNA, DNA	_	_	19,335 Pathogenic tolerance affecting variations, 21,170 Neutral human nonsynonymous coding SNPs (neutral tolerance data), 17,525 Clustered pathogenic tolerance affecting variations, 15,745 Clustered neutral tolerance affecting variations, 14,610 Pathogenic tolerance affecting variations, 17,393 Neutral human nonsynonymous coding SNPs (neutral tolerance data), 13,096 Clustered pathogenic tolerance affecting variations, 13,107 Clustered neutral tolerance affecting variations, 1,760 Functional and nonfunctional variants extracted from the Protein Mutant Database (PMD), 1,592 Clustered variants from the Protein Mutant database, 2,156 Variations from ProTherm, 1,784 Missense variations from 80 proteins, 964: 339 Variants in nine proteins and 625 variants from ProTherm database, 19 MLH1 and MSH2 gene variants	.xlsx
ClinVar	2013	DNA, RNA, Protein	Homo sapiens	Animal	4,391,341 records, 92,225 genes	.xml, .tsv, .vcf
BioLip	2012	DNA, RNA, Protein	Homo sapiens	Animal	873,925 Entries, 448,816 regular ligands, 191,485 mental ligands, 37,492 Peptide ligands, 43,448 DNA ligands, 152,684 RNA ligands, 873,925 binding affinity data, 451,485 Protein receptors	FASTA
OGEE	2011	Protein, Genes	91 species		Human cell lines: 931, Human tissues: 27, Human essential genes more than 57,878, Genes: 213,608, Conditional essential genes: 15,440	.txt
PDB	2011	Protein	Homo sapiens, Mus musculus, Arabidopsis thaliana, Saccharomyces cerevisiae	_	~150,000 entries	FASTA
Negatome database	2010	Protein, Domain pairs	_	_	Number of pairs: 30,756	.txt
ChEMBL	2009	Protein, Molecules, Compounds, Drugs	_	_	15,598 targets, 2,431,025 distinct compounds, 20,772,701 activities, 89,892 publications, 262 deposited datasets	.sdf, FASTA
ConSurf-DB	2009	Protein	Homo sapiens, Mus musculus	_	473,197 PDB chains, 108,958 non-redundant PDB chains	FASTA
dbPTM	2009	Protein	Homo sapiens	_	2,235,664 experimental sites, 542,107 putative sites, 2,777,771 sites, 82,444 literatures	FASTA
DUD-E	2009	Protein, Compounds	_	_	22,886 active compounds, 102 targets, 224 ligands	_
CCLE	2008	DNA, RNA, Protein	Homo sapiens	Animal	1,019 RNA cell lines, 954 microRNA expression profiles, 899 Protein lines, 897 Genome-wide histone modifications, 843 DNA methylation, 329 whole Genome Sequencing, 326 whole exome Sequencing	.csv
STITCH	2007	Protein, Chemical	Eukaryote, Prokaryote	2,031 organisms	more than 9,600,000 proteins, 340,000 to 430,000 compounds	.tsv.gz
DUD	2006	Protein, Compounds	_	_	2,950 active compounds, 40 targets	.mol2, .pdb, .sdf
PINA	2006	mRNA, Protein	_	_	Homo sapiens: Binary Interactions: 439,714, Complexes: 15,252, Saccharomyces cerevisiae: Binary Interactions: 128,319, Complexes: 6,302, Caenorhabditis elegans: Binary Interactions: 22,305, Complexes: 105, Drosophila melanogaster: Binary Interactions: 57,578, Complexes: 810, Mus musculus: Binary Interactions: 57,669, Complexes: 1,304, Rattus norvegicus: Binary Interactions: 5,796, Complexes: 307, Arabidopsis thaliana: Binary Interactions: 56,282, Complexes: 431, mRNA expression: Number of patients: 9,870, Number of genes: 608,188, Protein expression: Number of patients: 936, Number of proteins: 73,330	.csv, .excel
TCDB	2005	Protein	_	_	Protein sequences: 23,572, Transporter families: 1,929	FASTA
NCBI	2005	DNA, RNA, Protein	Homo sapiens, Mus musculus	Animal	35,608 CCDS IDs that correspond to 19,107 Genes, with 48,062 Protein Sequences	FASTA
PDBbind database	2004	Protein	_	_	Biomolecular complexes: 23,496, Protein–ligand: 19,443, Protein–protein: 2,852, Protein-nucleic acid: 1,052, Nucleic acid-ligand complexes: 149	.mol2, .sdf
PubChem	2004	Compounds, Genes, Protein, Cell lines	_	_	Compounds: 118,372,533, Substances: 319,659,057, BioAssays: 1,671,253, Bioactivities: 295,155,009, Genes: 113,242, Proteins: 247,869, Taxonomy: 108,194, Pathways: 241,163, Cell Lines: 2,005	.csv, .json, .xml, .sdf, .asnt
GOA	2003	Protein	_	_	68 million GO annotations to almost 54 million proteins in more than 480,000 taxonomic groups	GPAD, GPI
IEDB	2003	T Cell Epitopes, Antibodies, Protein	_	4,505 organisms	Peptidic Epitopes 1,619,619, Non-Peptidic Epitopes 3,188, T Cell Assays 536,844, B Cell Assays 1,405,550, MHC Ligand Assays 4,879,690, Restricting MHC Alleles 1,010, References 24,908	.xlsx, .tsv, .json, .csv
PhosphoSitesPlus	2003	Protein	_	Human, Mouse, Rat	Proteins: Non-redundant: 20,205, Total: 59,514, PTMs, all types: Non-redundant: 485,813, Total: 600,912, PTMs, low-throughput (LTP) methods: Non-redundant: 25,499, Total: 31,609, PTMs, high-throughput (HTP) MS/MS: Non-redundant: 478,249, Total: 588,707, MS peptides: Non-redundant: 640,925, Total: 2,631,035	.txt, .xlsx, FASTA, OWL
CTD	2003	Protein, Chemical, Genes, Phenotypes, Diseases, Chemical–Gene/Protein Interactions, Gene–Disease Associations, Chemical–Disease Associations, Chemical–Phenotype Interactions, Gene–Gene Interactions, Pathways	_	632 organisms	2,915,515 Chemical–gene interactions, 406,571 Phenotype–based interactions, 32,694,093 Gene–disease associations, 3,489,469 Chemical–disease associations, 6,577,078 Chemical–GO associations, 1,570,026 Chemical–pathway associations, 305,622 Disease–pathway associations, 1,358,371 Gene–gene interactions, 39,776,068 Gene–GO annotations, 135,792 Gene–pathway annotations, 3,133,281 GO–disease associations, 17,667 Chemicals with curated data, 7,285 Diseases with curated data, 55,128 Genes with curated data	.csv, .tsv, .xml
STRING	2003	Protein	_	12,535 organisms	59.3 million proteins, 20 billion interactions	.txt, .sql
BioGRID	2003	Protein	74 species	_	2,694,446 protein and genetic interactions, 31,144 chemical interactions, 1,128,339 post translational modifications, non-redundant interactions to 2,091,895, raw interactions to 2,694,446, non-redundant chemical associations to 13,719, raw chemical associations to 31,144, Non-Redundant PTM Sites to 563,757 and Un-Assigned PTMs to 57,396	.mitab, psi, psi25, tab, tab2, tab3
intAct	2002	Protein, Molecules	16 species	3,671 organisms	Binary Interactions 1,572,071, Interactions 844,973, Interactors 143,194, Proteins 124,275, Mutation Features 79,805, Experiments 75,229, Publications 23,417, Nucleic Acids 12,142, Controlled Vocabulary Terms 4,058, Genes 1,289, Interaction Detection Methods 246	.xml, tab, .json, xgmml
interPro	2002	Protein	_	12 organisms	3,510 homologous superfamily, 25,772 family, 14,524 domain, 379 repeat, 133 active sites, 75 binding sites, 741 conserved sites, 17 PTM	.tsv, .json, .txt
Therapeutic Targets Database	2001	Protein, Disease, Pathways, Drugs	Homo sapiens	_	Targets: 3,730, Drugs: 39,863	.xlsx, .txt
GEO	2000	DNA, RNA, Protein	21 species	_	7,209,691 samples	SOFT, MINiML, .txt
DIP	1999	Protein	834 species	_	28,850 proteins, 81,923 interactions	FASTA
Phospho.ELM	1999	Protein	Caenorhabditis, Drosophila, Vertebrate	_	8,718 substrate proteins covering 3,370 tyrosine, 31,754 serine and 7,449 threonine instances	.dump
RCSB PDB	1998	Protein	_	_	Structures from the PDB: 222,036, Computed Structure Models (CSM): 1,068,577	.txt, FASTA, .pdb, .xml, .sdf, .mol2, .cif, API
GeneCards	1997	Genes, Protein, RNA	Homo sapiens	Human	43,839 HGNC approved, 21,601 Protein coding, 291,492 RNA genes including 130,365 lncRNAs, 111,811 piRNAs, and 49,316 other ncRNAs	_
IMGT	1995	Genes, Protein	IMGT/LIGM-DB: 369 species, IMGT/PRIMER-DB: 11 species, IMGT/GENE-DB: 38 species	_	IMGT/LIGM-DB: Nucleotide sequences of IG and TR from 369 species (251,528 entries), IMGT/PRIMER-DB: Oligonucleotides (primers) of IG and TR from 11 species (1,864 entries), IMGT/GENE-DB: International nomenclature for IG and TR genes from 38 species (11,391 genes, 15,659 alleles), IMGT/3Dstructure-DB and IMGT/2Dstructure-DB: 3D structures (IMGT Colliers de Perles) of IG antibodies, TR, MH and RPI (8,751 entries), IMGT/mAb-DB: Monoclonal antibodies (IG, mAb), fusion proteins for immune applications (FPIA), composite proteins for clinical applications (CPCA), and related proteins (RPI) of therapeutic interest (1,489 entries)	FASTA
KEGG	1995	DNA, RNA, Protein	6 species	14 organisms	53,674,741 Genes, 4,181 Addendum Proteins, 6,88,823 Viral Genes, 377 Viral mature Peptides	KGML, FASTA, .txt
SCOP	1994	Protein	_	_	Number of folds: 1,562, Number of IUPR: 24, Number of hyperfamilies: 22, Number of superfamilies: 2,816, Number of families: 5,936, Number of inter-relationships: 60, Non-redundant domains: 72,544, Protein structures: 861,631	.txt, FASTA
EMBL-EBI	1994	DNA, RNA, Protein	_	_	~130 million sequences	.xml, FASTA, .txt, .tsv, .json
GPCRdb	1993	Protein, Drugs	_	_	424 Human proteins, 40,450 Species orthologs, 69,580 Genetic variants, 968 Drugs, 175 Drug targets, 405 Disease indications, 217,578 Ligands, 527 Endogenous ligands, 481,718 Ligand bioactivities, 35,606 Ligand site mutations, 48,039 Ligand interactions, 1,160 GPCRs structures, 842 GPCRs structure models, 2,922 Generic residues, 504 Refined structures	.json
CATH	1990	Protein	_	_	41 architectures, 1,390 topology, 6,631 homologous superfamily, 32,388 S35 superfamily, 45,835 S60 family, 62,915 S95 family, 122,727 S100 family, 500,238 domains	.txt, .gz, FASTA
Prosite	1989	Protein	Mammals	_	1,559 documentation entries, 1,308 patterns, 863 profiles and 869 ProRules	.dat, .doc, .txt
BRENDA	1987	Protein, Enzyme	_	16,018,959 organisms	38,623 active compounds, 32,832,265 sequences	.json, .txt
UniProtKB	1986	Protein	Archaea, Eukaryotes, Viruses	Bacteria	1,1206 Peptides	FASTA, .xml, .dat
OMIM	1960	DNA, RNA, Protein	Homo sapiens	Animal	17,290 Gene descriptions, 18 Gene and Phenotypes combined, 6,859 Phenotype description molecular basis known, 1,502 Phenotype description molecular basis unknown, 1,736 mainly Phenotypes with suspected mendelian basis	.txt

Based on an in-depth analysis of ‘organism’ feature in Table 1, we have categorized these databases into two different classes: 1) Narrow-organisms range, 2) Wide-organisms range databases. In narrow-organisms range databases, 20 or fewer organisms are present and we have included 12 databases to this category. This names of these databases are DisProt (106), MtSSPdb (79), OAS database (85), COSMIC (88), HPIDB (89), DisGeNET (72), HIPPIE (109), MalaCards (90), ClinVar (75), BioLip (76), CCLE (77) and NCBI (78). In contrast, remaining databases, also known as wide-organism range, contain more than 20 databases such as BRENDA (105), intAct (104), STRING (72), and CTD (103) etc.

Since word embeddings and LLMs based predictive pipelines require large amount of raw data for training in an unsupervised fashion, these databases act as facilitators for development of these predictive pipelines. For this, we have categorized these databases based on the volume of data into three different categories: (1) Low sequence facilitator, (2) Medium sequence facilitator, (3) High sequence facilitator. Low sequence facilitator databases provide with up to 100,000 sequence. A total of 26 databases are low sequence facilitator databases which include AmyPro (122), BindingDB (93), ChEMBL (91), DisProt (106), DUD (95), DUD-E (92), HPIDB (89), interPro (120), IPD-MHC (124), MalaCards (90), MtSSPdb (79), Negatome database (101), PDBbind database (118), Phospho.ELM (114), PPT-Ohmnet (108), SAbDab (117), SCOPe (132), STCRDab (129), TCDB (133), Therapeutic Targets Database (113), VDJdb (97), CCLE (77), UniProtKB (116), NCBI (78), OMIM (84) and Prosite (115). Similarly, medium sequence facilitator databases contain data sequences within a range of 100,000 to 1 million. There are 17 medium sequence facilitators databases namely CARD (73), CATH (138), ConSurf-DB (111), DIP (128), GeneCards (86), GLASS (131), GPCRdb (137), IMGT (87), MINT database (126), OGEE (127), PhosphoSitesPlus (119), PHROGs (107), PINA (82), RCSB (135), PDB (135), SCOP (136), GEO (80) and BioLip (76). In this study, 25 databases are identified as high sequence facilitator databases including AlphaFoldDB (121), BRENDA (105), dbPTM (112), GOA (134), IEDB (100), intAct (104), MGnify (99), MobiDB (123), OAS database (85), PubChem (102), KEGG (81), CTD (103), STRING (72), DisGeNET (139), BioGRID (125), STITCH (94), ClinVar (75), COSMIC (88), HIPPIE (109), McPAS-TCR (96), PIRD (98), Uniclust30 (130), VariBench (74), PDB (110) and EMBL-EBI (83).

A closer look on Table 1 ‘data format’ feature revels that in total 41 unique data formate have been used to house data into databases. These formats are, txt, .csv, .json, FASTA, .tsv, GAF, .xlsx, .pdf, MSA, HMM, .gff, .edgelist, .irf, .dat, .xml, RDF, SQL Dump, .sdf, .mitab, .gz, .tar, tab, OBL, OWL, .vcf, .tsv.gz, .mol2, .asnt, psi, psi25, tab2, tab3, xgmml, SOFT, MINiML, .dump, .cif, API, KGML, .dat and, doc. A detailed analysis of 295 studies reveals that, txt and FASTA formats are most commonly used by various protein sequence analysis pipelines. Each entry in these formats consists of at least two lines: first is known as header and includes accession number, species name, or identification details whereas other lines contain amino acid sequences. Second most commonly used formats are, csv and, tsv which are text-based formats and uses commas and tabs to separate values in rows. Specifically, in these two formats, first row signifies header which contain information title such as sequence ID, name, type, function, process and other details and preceding lines contain entries of data. In contrast, .xlsx format is used for complex datasets which contain computed information using various formulas across multiple columns. Additionally, vcf format also specifies headers in the first row and is mostly used to store genetic variation data which encompasses single nucleotide polymorphisms, insertions, deletions, and structural variants.

Furthermore, data related to protein function prediction is available in seven databases namely CARD (73), AlphaFoldDB (121), DisProt (106), GOA (134), MobiDB (123), SCOPe (132) and STCRDab (129). Similarly, data related to structure prediction, bitter peptides identification, domain boundary prediction, variant effects prediction, protein complexes identification, intrinsically disorder protein prediction, G-Protein coupled receptors identification and virus-host protein interaction prediction task is available in seven databases including AlphaFoldDB (121), AmyPro (122), BindingDB (93), CATH (138), ConSurf-DB (111), DIP (128) and DisProt (106), respectively. In addition, data for drug-target interaction and drug-protein interaction prediction is present in six databases namely DUD-E (92), BindingDB (93), ChEMBL (91), BRENDA (105), PubChem (102) and DUD (95) databases. Moreover, data for multiple interaction types prediction and compound-protein binding affinity prediction utilize tasks is extracted from ChEMBL (91) database, MINT database (126), intAct (104), and Therapeutic Targets Database (113). Additionally, data related to virus-host interaction prediction, protein–protein interaction prediction, gene functions prediction, secreted peptides prediction, antibody sequence infilling, phage-host interaction prediction, TRP channels classification and mutation effects prediction is available at 11 databases including GPCRdb (137), HIPPIE (109), HPIDB (89), intAct (104), MGnify (99), MtSSPdb (79), OAS database (85), PHROGs (107), RCSB PDB (135), TCDB (133) and VariBench (74) databases. Similarly, data regarding post-translational modification prediction is sourced from three different databases namely dbPTM (112), Phospho.ELM (114) and PhosphoSitesPlus (119). Moreover, two databases namely DUD (95) and PDBbind database (118) houses data for commercially available inhibitors prediction against SARS-CoV-2. Moreover, DUD (95) database also contains data related to drug-target binding affinity prediction, whereas GLASS (131), BindingDB (93), ChEMBL (91) facilitate with data related to compound-protein interaction prediction. IEDB (100) database is specific for providing sequences for anti-inflammatory peptides identification, Protein Binding Sites Prediction and MHC–peptide class II interaction prediction. Moreover, data related to enzyme substrate prediction and protein function identification is also available at interPro (120). In contrast, data related to disease genes identification is available at MalaCards (90), ChEMBL (91), intAct (104), MINT database (126) and GeneCards (86) database. Specifically, data related to protein–protein interaction prediction is provided by seven databases including DIP (128), HIPPIE (109), intAct (104), PINA (82), PPT-Ohmnet (108), PPT-Ohmnet (108), and MINT database (126). Furthermore, data related to essential genes identification is available at OGEE (127) and DIP (128) but OGEE (127) also facilitates with data related to essential gene identification. Data related to vascular calcification, protein properties prediction, remote homology detection, solubility, fold prediction and subcellular location identification tasks is available in five databases namely PDBbind database (118), BindingDB (93), PubChem (102), SCOP (136) and SCOPe (132) databases. Additionally, data related to nucleic acid binding protein prediction, secondary structure prediction, and binding affinity prediction task is present at 10 databases including Uniclust30 (130), MGnify (99), VDJdb (97), SCOPe (132), PIRD (98), DisProt (106), SCOP (136), BindingDB (93), ChEMBL (91) and PubChem (102) database.

## Protein sequence analysis benchmark datasets

This section presents a summary of 627 benchmark datasets that have been utilized for development of AI-driven applications across 63 diverse protein sequence analysis tasks. A rigorous analysis of 295 AI-driven protein sequence analysis articles reveals that these datasets are either created by authors or taken from existing studies. Among the 627 benchmark datasets, 403 are publicly accessible, while the remaining 224 are in-house. Table 2 illustrates 63 distinct protein sequence analysis tasks related 627 benchmark datasets distribution into public and in-house sources.

**Table 2.** Overview of 403 public and 224 in-house datasets used across 63 different protein sequence analysis tasks

Task name	Public	Private
Protein Family Classification	GLUT Dataset (141), SGLT Dataset (141), SWEET Dataset (141), GPCR Dataset(families, sub-families, sub-subfamilies) (227), COG Dataset (families) (227)	Balamurugan et al Dataset (140), Idhaya et al. Dataset (312), POG (226)
Protein Similarity Prediction	_	STRING-Yeast (229), STRING-Human Dataset (229), KGSIM-ALL-Pfam (229), KGSIM-ALL-PPI (229)
SNARE Proteins Identification	Li et al. Dataset (228), Le et al. Dataset (56), Kha et al. Dataset (56)	_
Electron Transport Protein Identification	Nguyen et al. Dataset (57)	_
G-protein Coupled Receptors Identification	_	Kim et al. Dataset (58)
Essential Proteins Identification	Yue et al. S. Cerevisiae Dataset (232), Zeng et al. Dataset (236), Saha et al. Dataset yeast PPIN (313), S. cerevisiae (BioGrid) (314), S. cerevisiae (DIP) (314), S. cerevisiae (Krogan) (314), H. sapiens (314), M. musculus (314), C. elegans (314), Zeng et al. Dataset (318)	BioGRID Dataset (230), DIP Dataset (230), Lu et al. Dataset (231), Cai et al. S. Cerevisiae Dataset (59), Wang et al. Dataset S. Cerevisiae Dataset (234), Inzamam et al. S. Cerevisiae Dataset (315), Inzamam et al. E. coli Dataset (315), E. coli Dataset (316), Zhang et al. S. Cerevisiae Dataset (317)
Intrinsically disordered Protein (IDP) Identification	TE82 Dataset (60)	_
Glycosylation and Glycation Modification Prediction	Ngly Dataset (13), Kgly Dataset (13), N-GlyDE Dataset (12), N-GlycositeAtlas (12)	_
Protein S-nitrosylation Modification Prediction	DeepNitro Dataset (14)	_
Phosphorylation Modification Prediction	Xu et al. Datasets (S/T (15), Y (15)), Guo et al. Datasets (S/T (17), Y (17)), Song et al. Mouse Phosphorylation Datasets (S (16), T (16), Y (16)), Song et al. PPA Test Datasets (S (16), T (16), Y (16))	Wang et al. P.ELM Datasets (S (18), T (18), Y (18)) Wang et al. PPA Datasets (S (18), T (18), Y (18))
Serine Phosphorylation Modification Prediction	Shrestha et al. Dataset (19)	_
Succinylation sites Modification Prediction	Hasan et al. Dataset (20)	_
Lysine Phosphoglycerylation Modification Prediction	Sohrawordi et al. Dataset (22), Chandra et al. Dataset (22)	_
Non-histone acetylation Modification Prediction	Meng et al. Dataset (143)	_
Protein S-sulfenylation Modification Prediction	Xu et al. Dataset (239)	_
Protein Glutarylation Modification Prediction	Huang et al. Dataset (238), Wang et al. Dataset (144)	_
Protein–Protein Interaction Prediction	Martin et al. Dataset (35), Dang et al. Human Dataset (35), Guo et al. Dataset (35), Ma et al. Dataset: Yeast Dataset (36), Multi-Species Dataset (36), Multi-Class Dataset (36), Zeng et al. Dataset: DeepPPISP Dataset (43), Albu et al. Dataset (240), Jha et al. Dataset: Human Dataset (145), Jha et al. Dataset: E. coli Dataset (145), Jha et al. Dataset: Drosophila Dataset (145), Jha et al. Dataset: C. elegan Dataset (145), Tsukiyama et al. Dataset: host-virus PPI Dataset (147), Guo et al. Dataset: Yeast PPI Dataset (147), Sun et al. Dataset: Human PPI Dataset (147), Ieremie et al. Datasets: S.cerevisiae Dataset (149), Ieremie et al. Datasets: H.sapiens Dataset (149), Chen et al. Dataset: STRING (148), Chen et al. Dataset: SHS27k (38), Chen et al. Dataset: SHS148k (38), Ozger et al. Dataset (39), Zheng et al. Dataset (40), Zhang et al. Dataset (45), Su et al. Dataset (241), Pan et al. Dataset: A. thaliana Dataset (242), Pan et al. Dataset: Zea mays Dataset (242), Pan et al. Dataset: Oryza sativa Dataset (242), Asim et al. Dataset: S.cerevisiae Dataset (243), Martin et al. Dataset: H. pylori Dataset (243), CCSB Dataset (65), HPRD Dataset (65), SARS-CoV2-host Dataset: Dataset 3 (65), SARS-CoV2-host Dataset: Dataset 4 (65), PPI network Dataset (244), GraphSAGE-PPI Dataset (244), E.coli PPI Dataset (CC, BP, MF) (245), Zhang et al. Dataset: SHS27k (37), Zhang et al. Dataset: SHS148k (37)	Nambiar et al. Dataset (42), Human PPI Dataset (46), S. cerevisiae PPI Dataset (46), Kermani et al. Dataset: HPRD Dataset (319), Kermani et al. Dataset: C. elegans Dataset (319), Kermani et al. Dataset: E. coli Dataset (319), Kermani et al. Dataset: M. musculus Dataset (319), Xu et al. Dataset: Yeast Dataset (146), Xu et al. Dataset: Human Dataset (146), HUMAN Dataset (BP, CC, MF) (246), MOUSE Dataset (BP, CC, MF) (246), YEAST Dataset (BP, CC, MF) (246), Murakami et al Dataset: Dset_186 (41), Murakami et al Dataset: Dset_72 (41), Singh et al. Dataset: Dset_164 (41), Zhang et al. Dataset: Dset_448 (41), Li et al. Dataset: Dset_355 (41)
Protein Complexes Identification	Gavin Dataset (248), Krogan core Dataset (248), DIP Dataset (248), MIPS Dataset (248), DIP Dataset (249), BIOGRID Dataset (249)	Krogan14K Dataset (247), Collins et al. Dataset (247), HPRD Dataset (247), Zhu et al. Dataset: Krogan Dataset (250), Zhu et al. Dataset: DIP Dataset (250), Zhu et al. Dataset: BIOGRID Dataset (250), Collins et al. Dataset (251), Gavin Dataset (251), Krogan Dataset (251), Kiemer et al. Dataset: Wiphi Dataset (251)
Residue-Residue Interaction Prediction	Hong et al. Dataset (252)	_
Contact Prediction	ProteinNet Dataset (148), trRosetta Dataset (150), HomoPDB Dataset (151), HetroPDB Dataset (151), DHTest Dataset (151), DB5.5 Dataset (151), SPOT-2018 (153), CASP14-FM (153)	_
Nucleic Acid-Binding Proteins Identification	Protein-DNA Dataset: Test_129 (157), Protein-DNA Dataset: Test_181 (157), Protein-RNA Dataset: Test_117 (157), YK17 Dataset (160), DRNA-1314 Dataset (160), RRM162 Dataset (253), Homeo215 Dataset (253), 690 ChIP-Seq Dataset (158), Patiyal et al. Dataset (156), Xia et al. (Dataset 2) (156), Liu and Tian (Dataset 1, Dataset 2) (159)	_
Protein RNA Interaction Prediction	NPInter2.0 (255), NPInter2.0_lncRNA (255), RPI7317 (255), RPI2241 (255), RPI38317 (255), RPI369 Dataset (320), RPI488 Dataset (320), RPI1446 Dataset (320), RPI1807 Dataset (320), RPI2241 Dataset (320)	_
Virus Host Protein Interaction Prediction	SARS-CoV-2 Interaction Dataset (47), H1N1 Dataset (47), Ebola Dataset (47), Xia et al. Dataset (51), Yang et al. Dataset (48), Barman et al. Dataset (52), Fatma et al. Dataset (52), Yang et al. Dataset (52), TR-TS1 Dataset (52), TR-TS2 Dataset (52), TR-TS1 Dataset (52), TR-TS2 Dataset (52)	Human-HIV Dataset (49), Human-Herpes Dataset (49), Human-Papilloma Dataset (49), Human-Influenza Dataset (49), Human-Hepatitis Dataset (49), Human-Dengue Dataset (49), Human-Zika Dataset (49), Human-SARS-CoV-2 Dataset (49), Chakraborty et al. Dataset: Set-1 Dataset (50), Chakraborty et al. Dataset: Set-2 Dataset (50), Chakraborty et al. Dataset: Set3-3 Dataset (50)
Compound-Protein Interaction Prediction	Liu et al. Dataset: Balanced Human Dataset (161), Liu et al. Dataset: Balanced C. elegans Dataset (161), Tsubaki et al. Datasets: Human Dataset (162), Tsubaki et al. Datasets: C.elegans Dataset (162), Gao et al. Dataset: BindingDB Dataset (162), Palhamkhani et al. Dataset: BindingDB Dataset (260), Chen et al. Dataset (261), Watanabe et al. Dataset (263), BindingDB 3CLpro Dataset (321), Wang et al. Dataset: BindingDB Dataset (453), Wang et al. Dataset: DrugBank Dataset (453), Wang et al. Dataset: GPCR Dataset (453), Davis et al. Dataset (453)	Koyama et al. Dataset: BindingDB Dataset (61), Wang et al. Dataset: Human Dataset (262), Wang et al. Dataset: C.elegans Dataset (262)
Compound-Protein Binding Affinity Prediction	Lin et al. Dataset: KIBA Dataset (63), Tang et al. Dataset: KIBA Dataset (161), Davis et al. Dataset (161)	Koyama et al. Dataset: PDBbind Dataset (61), Zhao et al. Dataset: Metz Dataset (61), Wang et al. Dataset: KIBA Dataset (163), Wang et al. Dataset: BindingDB Dataset (163)
Phage-Host Interaction Prediction	Gonzales et al. Dataset (164)	ESKAPE Dataset (264)
Missing Link Prediction	Balogh et al. Dataset: Homo sapiens Dataset (266), Balogh et al. Dataset: Saccharomyces cerevisiae Dataset (266), Balogh et al. Dataset: Mus musculus Dataset (266), Balogh et al. Dataset: Rattus norvegicus Dataset (266), Balogh et al. Dataset: Sus scrofa Dataset (266)	Kermani et al. Dataset: H. sapiens Dataset (265), Kermani et al. Dataset: M. musculus Dataset (265), Kermani et al. Dataset: S. cerevisiae Dataset (265), Orphanet Dataset (267), Nasiri et al. Dataset: E.coli Dataset (268), Nasiri et al. Dataset: C.elegan Dataset (268), Nasiri et al. Dataset: Drosophila Dataset (268), PPI Dataset (270), Homo Sapiens PPI Network Dataset (270), Feng et al. Dataset (269)
Anti-cancer peptides	ACP_Main (352), ACP_Alternate (353), ACP_344 (354), ACP_mixed_80 (355), Set 1 (356), Set 2 (356), ACP_539 (325), ENNAACT_main (357)	_
Anti-bacterial Peptides	Sharma et al. Dataset (54), Youmans et al. Dataset (337), Youmans et al. Dataset (Old) (358)	Singh et al. Dataset (359), Khaledian et al. Dataset (360)
Antimicrobial peptides	Bournez et al. Dataset (55), Xu et al. Dataset (328), Xiao et al. Dataset (361), Lin et al. Dataset (330), Olcay et al. Dataset (331), Teimouri et al. Dataset (332), Veltri Dataset (165), LMPred Dataset (165), Wang et al. Dataset (333), Jaiswal et al. Dataset (334)	Wani et al. Dataset (362), Söylemez et al. Dataset (363), Sharma et al. Dataset (364), Kavousi et al. Dataset (365), AMP-11053 (366), AMP-2211 (366), Bournez et al. Dataset (55), Chung et al. Dataset (367), Chung et al. Dataset (356), Xiao et al. Dataset (329), Yu et al. Dataset (368), Gull et al. Dataset (369)
Signal Peptides	Teufel et al. Dataset (Sec/SPaseI, Sec/Spase II,Tat/SPaseI) (438), SignalP 5.0 (370)	Petersen et al. Dataset (371), Savojardo et al. Dataset (371), Zhang et al. Dataset (372), SPdb Dataset (372), Choo et al. Dataset (372), Zeng et al. Dataset (373), SP19 (374)
Secreted Peptides	SSPs Dataset (167)	_
Anti-Inflammatory Peptides	Gupta Dataset (340), Manavalan Dataset (375), Deng Dataset (342)	Deng Independent Test Set-2 (168)
Hormone Peptides	Kaur et al. Dataset (376)	_
Peptide-Binding Specificity Prediction	Peptide-MHC Dataset (170)	_
Drug-Protein Interaction Prediction	Zhou et al. Dataset: BindingDB Dataset (171), Zhou et al. Dataset: Davis Dataset (171), Zhou et al. Dataset: Yamanishi et al. Datasets (Enzyme, GPCR, IC, NR) (171), Zhang et al. Datasets: DrugBank Dataset (172), Zhang et al. Datasets: Epigenetic-regulators Dataset (172), DUD-E Dataset (271), Xuan et al. Dataset (272), Sun et al. Dataset (343)	ER Dataset (173), Ion-C Dataset (173), RTK Dataset (173), GPCR Dataset (173)
Drug-Target Binding Affinity Prediction	Xia et al. Dataset: Ki Dataset (174), Davis et al. Dataset (174), Hu et al. Dataset: PDBbind Dataset (271), Wang et al. Dataset: PDBbind Dataset (344), Wang et al. Dataset: CASF2016 Dataset (344), Wang et al. Dataset: Core 2016 Dataset (345), Wang et al. Dataset: Test71 Dataset (345)	Tang et al. Dataset: KIBA Dataset (175)
Gene Phenotype Prediction	Kafkas et al. Dataset (1,2,3) (177), Chen et al., 2016 Dataset (273)	_
Disease Genes Prediction	Li et al. Dataset (178), ClinVar_BRCA1 Dataset (180), ClinVar_PTEN Dataset (180), Wang et al. Dataset (274), Yang et al. Dataset (293), Pancan TCGA Dataset (281), Luo et al. Dataset (291), Ratajczak et al. Dataset (Cardiovascular Disease, Immune Dysregulation, Body Mass Disorder, Diabetes, Insulin Disorder) (277), Jagodnik et al. Dataset (278), Liu et al. Dataset (288), Wang et al. Dataset (346)	Wang et al. Dataset (179), Chu et al. Dataset (275), Vilela et al. Dataset (276), Zhang et al. Dataset (279), Wang et al. Dataset (282), Prabhakar et al. Dataset (283), Wang et al. Dataset (284), Liu et al. Dataset (285), Du et al. Dataset (286), Xu et al. Dataset (287), Madeddu et al. Dataset (289), Peng et al. Dataset (290), Zhu et al. Dataset (292), Ata et al. Dataset (294), Gao et al. Dataset (347)
Essential Genes Identification	Ibrahim et al. Dataset (295)	FIs Dataset (296), InWeb_IM Dataset (296)
Prokaryotic Gene Prediction	Tu et al. Dataset (181)	_
Solubility Prediction	Khurana et al. Dataset (150), S. cerevisiae Dataset (182), Bhandari et al. PSI:biology Dataset (348), Niwa et al. eSOL Dataset (348), Smialowski et al. e-coli Dataset (348), Price et al. Dataset (348)	_
Stability Prediction	Rockline et al. Dataset (150), S2648 (184), Ssym (184), S669 (184), CAGI5 Challenge’s Frataxin (184)	_
Thermophilicity Prediction	Haselbeck et al. Dataset (187)	_
Fluorescence Prediction	Sarkisyan et al. Dataset (185)	_
Domain Boundary Prediction	_	FM Dataset (189), Multi-Domain Dataset (189), DCD Dataset (189)
Protein Function Identification	CAFA3 (191), You et al. Dataset (MF, BP, CC) (192), Zhapa et al. Dataset (MF, BP, CC) (193), Kulmanov et al. Dataset (MF, BP, CC) (194), Kulmanov et al. Dataset neXtProt (MF, BP) (194), Chua et al. Dataset (MF, BP, CC) (196), Zhao et al. Dataset Human (CC, MF, BP) (198), Zhao et al. Dataset Mouse (CC, MF, BP) (198), Zhao et al. Dataset Arabidopsis (CC, MF, BP) (198), Pang et al. Dataset DP93 (Protein Binding, DNA Binding, RNA Binding, Ion Binding, Lipid Binding, Flexible linker functional sites) (199), Pang et al. Dataset DP94 (199), Zhang et al. Dataset (MF, BP, CC) (297), STCRDAB Dataset (298), PDB Bind Dataset (298), CAID Dataset (DisProt, DisProt-PDB, disordered region, disordered proteins functions) (201), TE176 Dataset (Protein binding, DNA binding, RNA binding, Flexible linker disordered functions) (201), Ahmed et al. Dataset Benchmark (202), Ahmed et al. Dataset Balanced Test Set (202), Yuan et al. Dataset (MF, BP, CC) (203), Yeast (BP, MF, CC) Dataset (204), Human (BP, MF, CC) Dataset (204), Arabidopsis (BP, MF, CC) Dataset (204), TDNK Dataset (BP, CC, MF) (205), RS Dataset (BP, CC, MF) (205), TSNK Dataset (BP, CC, MF) (205), 2016 Dataset (206), Hu et al. Dataset (207), Wang et al. Dataset (BP, CC, MF, EC) (185), Wu et al. Dataset Yeast (BP, MF, CC) (299), Wu et al. Human (BP, MF, CC) (299), Mouse Dataset (300), UniProtKB/SwissProt (304), NEW Dataset (305), Zhang et al. Dataset (BP, CC, MF), (303) Gligorijević et al. Dataset (MF, CC, BP) (186)	Antibiotic Resistance CARD Dataset (150), Fluorescence TAPE Dataset (150), Fitness Dataset (150), Tawfiq et al. Dataset (MF, BP, CC) (190), Shaw et al. Dataset Random EC (195), Shaw et al. Dataset Random GO (195), Shaw et al. Dataset Clustered EC (195), Shaw et al. Dataset Clustered GO (195), Shaw et al. Dataset NEW-392 (195), Shaw et al. Dataset Price-149 (195), Shaw et al. Dataset PDB EC (195), Shaw et al. Dataset Clustered Pfam (195), Zhang et al. Dataset (197), AMIE Dataset (200), B3VI55_LIPSTSTABLE Dataset (200), B3VI55_LIPST Dataset (200), BF520 Dataset (200), BG505 Dataset (200), BG_STRSQ Dataset (200), BLAT_2014 Dataset (200), BLAT_2012 Dataset (200), BLAT_2015 Dataset (200), BLAT_2013 Dataset (200), BRCA1_BRCT Dataset (200), BRCA1_RING Dataset (200), CALM1_Roth2017 Dataset (200), DLG4_RAT Dataset (200), GAL4 Dataset (200), HG_FLU Dataset (200), HSP82 Dataset (200), IF1_ECOLI Dataset (200), MK01 Dataset (200), MTH3 Dataset (200), P84126 Dataset (200), PABP Dataset (200), PA_FLU Dataset (200), POLG_HCVJF Dataset (200), PTEN Dataset (200), RASH Dataset (200), RL401_2013 Dataset (200), RL401_2014 Dataset (200), RL401_2016 Dataset (200), SUMO1 Dataset (200), TIM_SULSO Dataset (200), TIM_THEMA Dataset (200), TPK1_2017 Dataset (200), TPMT_2018 Dataset (200), UBC9 Dataset (200), UBE4B Dataset (200), YAP1 Dataset (200), HIV_Tat Dataset (200), Tseng et al. Dataset (CC, MF, BP) (301), CORUM Dataset (BP, MF, CC) (302), Islam et al. Dataset (BP, CC, MF)(349)
Structure Prediction	Cuff et al. TS115 Q8 Dataset (60), Cuff et al. CASP12 Q8 Dataset(60), Cuff et al. CASP14 Q3 Dataset (208), NEW364 Q3(208), Feng et al. Strict_Data (Unbalanced, Balanced) (209), Feng et al. NonStrict_Data (Unbalanced, Balanced)(209), SetTst29 (211), NetSurfP Q3 Dataset (188)	CAMEO (150), CASP15 (150), Chen et al. Dataset (150), CASP12 + CASP 14 Dataset (150), Cuff et al. CASP12 Q3 Dataset (27), Cuff et al. TS115 Q3 Dataset (27), Cuff et al. CB513 Dataset (27), Cuff et al. CASP14 Q8 Dataset (27), Lin et al. CASP14 (210)
Fold Prediction	Hou et al. Dataset (150)	LINDAHL Dataset (214), LINDAHL_1.75 Dataset (214), SCOP_2.06 Dataset (214)
Remote Homology Detection	Top 1773 Superfamilies Dataset (215), Top 50 Superfamilies Dataset (215), Hou et al. Dataset (377), Foldseek Benchmark Dataset (216)	Rives et al. Dataset (213), Routray et al. Dataset (1,2,3,4) (350)
TRP channels Classification	Shah et al. Dataset (217)	_
Protein Subcellular Localization Identification	Luo et al. Swis-Prot Datasets (Cell junction (23), Cell membrane (23), Cell projection (23), Cytoplasm (23), Golgi apparatus (23), Lysosome (23), Mitochondrion (23), Nucleus (23), Secreted (23)) Luo et al. TrEMBL Datasets (Cell junction (23), Cell membrane (23), Cell projection (23), Cytoplasm (23), Golgi apparatus (23), Lysosome (23), Mitochondrion (23), Nucleus (23), Secreted (23), Endoplasmic reticulum (23)), Armenteros et al. Dataset (27), Swiss-Prot CV Dataset (28), HPA Independent Dataset (28), Pan et al. Yeast Dataset (30), Pan et al. Human Dataset (31), Gram-Positive bacteria Dataset (25), Gram-Negative bacteria Dataset (25), Viral Dataset (25), Plant Dataset (25), Human Dataset (25), SARS-CoV-2 Dataset (25), Gillani et al. Datasets (Other (26), Membrane (26), Cytoplasm (26), Golgi Apparatus (26), Mitochondrion (26), Nucleus (26), Plastid (26), Secreted (26))	Subcellular location Dataset (32)
Protein Submitochondrial Localization Identification	Savojardo et al. SM424 Dataset (218), Kumar et al. SM570 Dataset (218), Wang et al. Datasets (Human.Mitocarta3.0 (218), Mouse.Mitocarta3.0 (218))	Hou et al. Datasets (M187 (306), Human.MitoCarta3.0 (306), Mouse.Mitocarta3.0 (306)), M317 Dataset (351), M983 Dataset (351), M495 Dataset (351), M1217 Dataset (351)
Subchloroplast Localization Identification	MSchlo578 Dataset (219), Novel Dataset (219)	_
Mutation Prediction	Tzavella et al. Dataset (TP53, BRAF, AR, CHEK2, PTEN) (220)	_
Mutation Effects Prediction	Yang et al. Dataset (221), Strokach et al. Dataset (223)	Riesselman et al. Dataset (222)
Variant Effects Prediction	_	Marquet et al. Datasets (PMD4k (224), DMS4 (224))
Malaria Parasite Identification	_	Verma et al. Dataset (307)
Tumour Necrosis Factors Identification	_	Nguyen et al. Dataset (308)
COVID-19 Virus Classification	_	Adjuik’s et al. Dataset (309)
Vascular Calcification	Chao et al. Dataset (310)	_
B/T Cell Receptor Sequences Analysis	DS1 (311), DS2 (311), DS3 (311)	_
B-Cell Epitopes Identification	Zeng et al. Dataset (225)	_

A thorough analysis of AI-driven protein sequence analysis literature reveals that:

- **229** public and **100** in-house datasets are used to develop LLMs-based applications for **47** different protein sequence analysis tasks including Protein Family Classification (140, 141), G-protein Coupled Receptors Identification (58), Intrinsically disordered protein (IDP) identification (60), Glycosylation and Glycation modification prediction (12, 13, 142), Protein S-nitrosylation modification prediction (14), Phosphorylation modification prediction (15), Serine phosphorylation modification prediction (19), Succinylation modification prediction (20), Lysine phosphoglycerylation modification prediction (21, 22), Non-histone acetylation modification prediction (143), Protein Glutarylation modification prediction (144), Protein–Protein Interaction Prediction (35, 36, 42–44, 46, 145–149), Contact Prediction (27, 148, 150–155), Nucleic Acid-Binding Proteins Identification (156–160), Virus Host Protein Interaction Prediction (47), Compound-Protein Interaction Prediction (61, 161, 162), Compound-Protein Binding Affinity Prediction (61–64, 161, 163), Phage-Host Interaction Prediction (164), Antimicrobial Peptides Identification (165), Signal Peptides Identification (166), Secreted Peptides Identification (167),Anti-Inflammatory Peptides identification prediction (168), Antibody Sequence Infilling (169), Peptide-Binding Specificity Prediction (170), Drug-Protein Interaction Prediction (171–173), Drug-Target Binding Affinity Prediction (174–176), Gene Phenotype Prediction (177), Disease Genes Prediction (178–180), Prokaryotic Gene Prediction (181), Solubility Prediction (27, 150, 182, 183), Stability Prediction (150, 154, 155, 184–186), Thermophilicity Prediction (187), Fluorescence Prediction (27, 154, 155, 185, 186, 188), Domain Boundary Prediction (189), Protein Function Identification (150, 185, 186, 190–207), Structure Prediction (27, 60, 150, 154, 155, 188, 208–213), Fold Prediction (27, 150, 214), Remote Homology Detection (136, 154, 188, 213, 215, 216), TRP channels Classification (217), Protein Subcellular Localization Identification (23, 27, 28), Protein Submitochondrial Localization Identification (218), Subchloroplast Localization Identification (219), Mutation Prediction (220), Mutation Effects Prediction (221–223), Variant Effects Prediction (224), and B-Cell Epitopes Identification (225).
- **86** public and **79** in-house datasets are used to develop word embeddings based AI applications for **32** protein sequence analysis tasks including Protein Family Classification (226, 227), SNARE proteins Identification (228), Protein Similarity Prediction (229), Electron Transport Protein Identification (57), Essential Proteins Identification (59, 230–237), Phosphorylation modification prediction (16–18, 18), Protein Glutarylation Modification Prediction (238), Protein S-sulfenylation modification prediction (239), Protein–Protein Interaction Prediction (38–40, 45, 65, 240–246), Protein Complexes Identification (247–251), Residue-Residue Interaction Prediction (252), Nucleic Acid-Binding Proteins Identification (253), Protein RNA Interaction Prediction (254–259), Virus Host Protein Interaction Prediction (48, 49, 51), Compound-Protein Interaction Prediction (260–263), Phage-Host Interaction Prediction (264), Missing Link Prediction (265–270), Antibacterial peptides (54), Drug-Protein Interaction Prediction (271, 272), Drug-Target Binding Affinity Prediction (271), Gene Phenotype Prediction (273), Disease Genes Prediction (274–294), Essential Genes Identification (295, 296), Protein Function Identification (297–305), Protein Subcellular Localization Identification (30–33), Protein Submitochondrial Localization Identification (306), Malaria Parasite Identification (307), Tumour Necrosis Factors Identification (308), COVID-19 Virus Classification (309), Vascular Calcification (310), and $B/T$ Cell Receptor Sequences Analysis (311).
- **88** public and **46** in-house datasets are used to develop domain-specific representation learning based AI applications for **21** different tasks namely Protein Family Classification (312), SNARE proteins Identification (56), Essential Proteins Identification (313–318), Protein S-sulfenylation modification prediction (239), Protein–Protein Interaction Prediction (37, 41, 319), Protein RNA Interaction Prediction (320), Virus Host Protein Interaction Prediction (50, 52), Compound-Protein Interaction Prediction (321), Anticancer Peptides identification prediction (53, 322–327), Antimicrobial Peptides identification prediction (55, 328–335), Signal Peptides identification prediction (336), Antibacterial peptides (337), Anti-Inflammatory Peptides identification prediction (338–342), Drug-Protein Interaction Prediction (343), Drug-Target Binding Affinity Prediction (344, 345), Disease Genes Prediction (346, 347), Solubility Prediction (348), Protein Function Identification (349), Remote Homology Detection (350), Protein Subcellular Localization Identification (25, 26), and Protein Submitochondrial Localization Identification (351).

Through a large-scale literature review, we investigate the reuse patterns of common datasets across three distinct types AI applications paradigms: LLMs, word embeddings, and domain-specific representation learning methods. Our conclusions on the overlap between all three paradigms related AI-driven applications and dataset reuse patterns across 63 distinct protein sequence analysis tasks are as follows:

- **245** public and **124** in-house datasets are used to develop both word embedding and LLMs based predictive pipelines for **12** different protein sequence analysis tasks including Protein–Protein Interaction Prediction, Virus Host Protein Interaction Prediction, Compound-Protein Interaction Prediction, Drug-Protein Interaction Prediction, Drug-Target Binding Affinity Prediction, Disease Genes Prediction, Protein Function Identification, Protein Subcellular Localization Identification, Protein Family Classification, Nucleic Acid-Binding Proteins Identification, Phage-Host Interaction Prediction, and Gene Phenotype Prediction.
- **253** public and **138** in-house datasets are leveraged for development of word embedding and domain-specific representation learning approaches based predictive pipelines for **13** tasks namely Protein–Protein Interaction Prediction, Virus Host Protein Interaction Prediction, Compound-Protein Interaction Prediction, Drug-Protein Interaction Prediction, Drug-Target Binding Affinity Prediction, Disease Genes Prediction, Protein Function Identification, Protein Subcellular Localization Identification, Essential Proteins Identification, Protein S-sulfenylation modification prediction, Protein RNA Interaction Prediction, Antibacterial peptides identification, and Protein Submitochondrial Localization Identification.
- **259** public and **149** in-house datasets are utilized for the development of both LLMs and domain-specific representation learning approaches based predictive pipelines for **14** protein sequence analysis tasks including Protein–Protein Interaction Prediction, Virus Host Protein Interaction Prediction, Compound-Protein Interaction Prediction, Drug-Protein Interaction Prediction, Drug-Target Binding Affinity Prediction, Disease Genes Prediction, Protein Function Identification, Protein Subcellular Localization Identification, SNARE proteins Identification, Phosphorylation modification prediction, Antimicrobial Peptides identification, Signal Peptides identification, Solubility Prediction, and Remote Homology Detection.

A rigorous analysis of existing studies reveal that only two public datasets are commonly employed by both word embedding and language models based predictive pipelines for Protein–Protein Interaction Prediction, six public datasets are commonly employed by both word embedding and domain-specific representation learning approaches based predictive pipelines for Protein Function Identification, and four public datasets are commonly employed by both language models and domain-specific representation learning approaches based predictive pipelines for protein–RNA interaction prediction. Overall for all three kinds of predictive pipelines, **221** public and **120** in-house datasets are used to develop predictive pipelines for **8** different protein sequence analysis tasks namely Protein–Protein Interaction Prediction, Virus Host Protein Interaction Prediction, Compound-Protein Interaction Prediction, Drug-Protein Interaction Prediction, Drug-Target Binding Affinity Prediction, Disease Genes Prediction, Protein Function Identification, and Protein Subcellular Localization Identification. Surprisingly, not a single dataset is commonly used by all three kind of predictive pipelines. This trend highlights a tendency among researchers to create new datasets for each predictive pipeline instead of reusing existing ones. Consequently, protein sequence analysis domain lacks robust comparisons of predictive pipeline performance.

A holistic view of Table 2 reveals that seven distinct tasks related public datasets are not available because existing application are developed on in-house datasets only. These tasks include protein similarity prediction, G-protein coupled receptors identification. The highest number of public datasets are available for two tasks namely Protein Function Prediction, Protein–Protein Interaction prediction, and Protein Subcellular Localization Prediction with 88, 40, 38 datasets. It is difficult to perform experimentation on such a large number of datasets, to stream line development of applications. Hence, there is need to analyse all these tasks related public datasets and develop new species specific datasets and benchmark performance on these datasets.

## Word embeddings based AI-driven protein sequence analysis predictive pipelines

**Figure 5.**
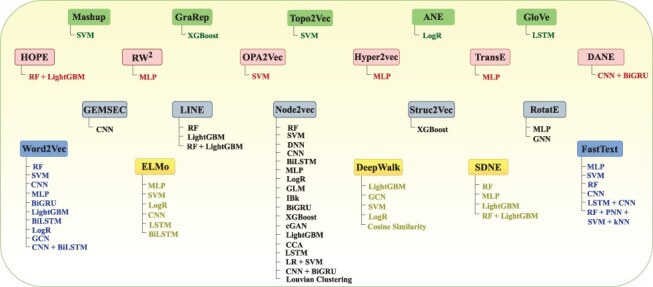
Utilization of 22 different word embedding methods in diverse protein sequence analysis pipelines based on a variety of machine and deep learning predictors

This section outlines 22 distinct word embedding methods that are utilized in AI-driven predictive pipeline development for 63 different protein sequence analysis tasks. These methods include Mashup (299, 304), Word2Vec (32, 309, 311), Node2Vec (231, 271), FastText (239), RotatE (283), ANE (265), ELMo (297), DANE (230), GEMSEC (59), LINE (40), Struc2Vec (275), TransE (276), DeepWalk (280), Hyper2Vec (282), Opa2Vec (284), Random Watcher-Walker (RW2) (289), HOPE (274), RandomWalk (265), SDNE (274), GloVe (238), GraRep (261), and Topo2Vec (270). Figure 5 provides a comprehensive overview of these 22 word embedding methods along with 21 different predictors namely SVM (281), RF (302), BiLSTM (306), CNN (306), MLP (229), BiGRU (230), GCN (247), LogR (252), LightGBM (48), k-means (251), IBk (273), GLM (294), Ensemble (LR + BR + DTR + SVM) (248), XGBoost (261), cGAN (266), CCA (51), Louvain clustering (65), LSTM (245), ET (296), and GAT (255).

A high-level analysis of Figure 5 reveals that Node2Vec emerges as the most extensively explored method, followed by Word2Vec, FastText, and ELMo. Specifically, Node2Vec (231, 271) is employed with 18 different predictors, while Word2Vec (32) based predictive pipelines have used 10 predictors. On the other hand, FastText (239) and ELMo (297) based predictive pipelines have reaped benefits of six predictors each. DeepWalk based predictive pipelines (280) has employed five predictors and SDNE based predictive pipelines (274) have used four predictors. Additionally, LINE (40) and RotatE (283) based predictive pipelines have leveraged 3 and 2 predictors, respectively. However, potential of remaining 14 word embedding methods have not been fully explored as they are evaluated with only one predictor. This highlights the need of further investigation to fully explore their potential.

In the realm of protein sequence analysis, word embedding methods are utilized in two primary ways to generate sequence embeddings. First approach segregates protein sequences into k-mers and generates embeddings for each individual k-mers. In contrast, second approach generates embeddings for entire protein sequences, which can be subsequently used for homogeneous and heterogeneous networks. Homogeneous networks deal with a single type of biomolecule, such as protein–protein interaction networks. On the other hand„ heterogeneous networks involve multiple types of biomolecules, including proteins, RNA (e.g. lncRNA, miRNA, circRNA (378, 379)), drugs, compounds, and diseases. In heterogeneous networks, nodes represent biomolecules, while their interactions or associations form the edges. Heterogeneous networks include disease-gene association networks, compound-protein interaction networks, RNA-protein interaction prediction, and virus-host interaction prediction. Compared to homogeneous networks, heterogeneous networks are more complex and enable the extraction of richer and more comprehensive relationships through graph-based embedding methods.

Among 22 unique word embedding methods, 10 method (Word2Vec, FastText, DANE, ANE, ELMo, GloVe, Node2Vec, LINE, GraRep, and SDNE) have been employed to generate k-mer based embeddings of sequences across 8 different predictive pipelines (38, 255, 258, 262, 271, 281, 287, 300). An extensive review of existing studies reveals that Word2Vec is most widely explored in the k-mer approach, followed by FastText and Node2Vec. Specifically, 13 predictive pipelines have employed Word2Vec using k-mer approach with eight different predictors for protein family classification (226), protein–protein interaction prediction (40), and anti-bacterial peptides identification (54). These predictors include CNN (226, 227), SVM (32), LogR (311), MLP (242), GNN (254), RF (259), BiLSTM (49, 54), and BiLSTM + MCNN (303). Additionally, nine predictive pipelines (57, 228, 239, 243, 253, 302, 305, 307, 308) have utilized FastText in k-mer approach for eight different tasks and leverages 7 predictors including CNN (228), SVM (57), MLP (239), LSTM + CNN (243), affinity regression (253), RF (302), and an ensemble (RF + PNN + SVM + KNN) (307). Moreover, 5 predictive pipelines has explored the potential of Node2Vec in k-mer approach. Node2Vec based predictive pipelines have leveraged 4 predictors (GNN (38, 258), MLP (271), GAT (255), BiGRU (271)) for three different protein sequence analysis tasks namely protein–protein interaction prediction (38), RNA-protein interaction prediction (255), and disease gene interaction (271).

Moreover, 18 distinct predictive pipelines have used 12 unique word embedding methods by generating embedding for entire bio-molecule (Homogeneous network) for eight distinct protein sequence analysis task including protein similarity prediction (229), essential genes identification (59, 231–235), compound-protein interaction prediction (260, 263), missing link identification (270), residue-residue interaction prediction (252), protein complex identification (248–251), protein–protein interaction prediction (45, 244–246, 319), and virus-host interaction prediction (51). These 12 unique word embedding methods includes Node2Vec (277), GEMSEC (59), DeepWalk (244), Topo2Vec (270), LINE (274), SDNE (274), HOPE (274), Struc2Vec (275), Hyper2Vec (282), Opa2Vec (284), Random Watcher-Walker (RW2) (289), and Mashup (299). Among all these word embedding methods, Node2Vec and DeepWalk are explored for entire sequence embedding generation across 10 distinct predictive pipelines (45, 231–237, 244, 245).

Apart from this, 14 predictive pipelines (65, 247, 256, 267, 272, 276, 278–280, 291–294, 310) have leveraged seven unique word embedding methods for heterogeneous network for six distinct protein sequence analysis task including protein–protein interaction prediction, protein complex identification, protein–RNA interaction prediction, drug-protein interaction prediction, disease gene prediction and vascular calcification. These methods include Node2Vec (65, 267, 272, 278–280, 291, 294, 310), HIN2Vec (256), TransE (276), DeepWalk (280), LINE (280), and SDNE (280). Surprisingly, 11 predictive pipelines have explored Node2Vec for heterogeneous network along with seven predictor including Louvain clustering (65), LightGBM (267, 280), GNN (272), MLP (279, 291), LogR (278), GLM (294), and RF (310). An in-depth analysis of existing studies reveals that four embedding approaches (DeepWalk, Node2vec, LINE, SDNE) are used for generating k-mer embeddings as well as entire sequence embeddings in both homogeneous and heterogeneous networks.

## Language models based AI-driven protein sequence analysis predictive pipelines

This section highlights 15 unique language models that are used to develop 137 AI-driven predictive pipelines for 63 distinct protein sequence analysis tasks. These language models include BERT (140), ALBERT (175), RoBERTa (183), T5 (17, 20), GPT (177), ESM-1 (181, 200), ESM-2 (150, 190), AlphaFold (207), AlphaFold2 (161), IgFold (169), MolFormer (61), Graph Transformer Network (178), XLNet (212), Vision Transformer (46) and Transformer (15, 19).

**Figure 6.**
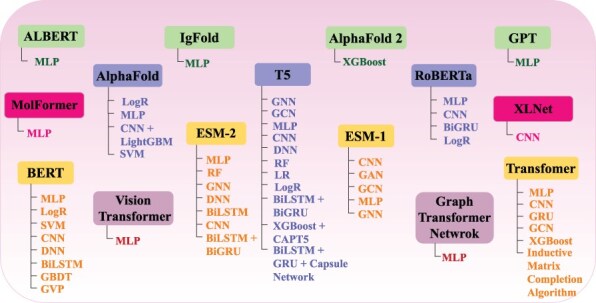
Utilization of 15 different LLMs in diverse protein sequence analysis pipelines based on a variety of machine and deep learning algorithms

The training paradigm of language models involves two stages: pre-training and fine tuning. Pre-training involves training model on a large amount of unlabeled data to learn underlying patterns or structures of data in an unsupervised fashion. Specifically, language models learn contextual patterns by using masked language modelling and next sentence prediction approaches. In masked language modelling approach, some k-mers in a sequence are replaced with a mask token, and model learns to predict the masked k-mers whereas next sentence prediction approach focus on predicting next sequence based on preceding sequence. By learning contextual patterns, model acquires a deep understanding of linguistic features like syntax, semantics, and context without need of labelled data. Fine tuning stage adapts pre-trained model to perform downstream tasks including protein family classification, protein function identification, or protein–protein interaction prediction. During fine-tuning, model is further trained on a task-specific labelled dataset. Specifically for this process, model can be used in two ways: 1) By using its self-classifier, 2) Integrating external classifiers to optimize task performance. Figure 6 graphically illustrates the utilization of 15 different language models, either paired with their self-classifiers or used in combination with 18 external classifiers. The external classifiers include SVM (12), LogR (202), RF (164), GBDT (223), XGBoost (35), MLP (161, 196), Hybrid (XGBoost + CAPT5) (35), CNN (142), GRU (167), BiGRU (62), BiLSTM (189), GNN (206), GCN (182), GAT (44), GAN (198), GVP (186), BiLSTM + BiGRU (60), and Inductive Matrix Completion Algorithm (179).

In protein sequence analysis landscape, among 137 predictive pipelines based on 15 LLMs, 72 predictive pipelines have leveraged self-classifier, whereas 65 pipelines make use of an external classifier to perform downstream tasks. Among 72 predictive pipelines based on language models leveraging self-classifier, BERT stands out the most extensively used language model, followed by T5 and ESM-2. Specifically, 14 predictive pipelines (21, 58, 140, 147, 154, 158, 174, 180, 186, 186, 188, 188, 188, 218) have explored the potential of BERT language model with a self-classifier for eight different tasks, eight predictive pipelines (17, 20, 27, 28, 187, 195, 203, 214) has employed T5 with a self-classifier for 11 different tasks and seven predictive pipelines (23, 60, 150, 190, 193, 194, 210) has leveraged ESM-2 for seven different tasks. In addition, 12 transformer based predictive pipelines (15, 19, 47, 149, 171, 173, 185, 185, 185, 213, 213) and 3 ESM-1 based predictive pipelines (181, 200, 222) utilize self-classifier for seven and two different protein sequence analysis tasks, respectively. On the other hand, four predictive pipelines have employed GPT (177), AlphaFold (207), RoBERTa (183), and graph transformer network (178) with a self-classifier for four different tasks. Beyond exploring standalone potential of language models, 10 predictive pipelines reap combined benefits of multiple language models or in combination with other approaches as: (Transformer + GNN) (63), (T5 + OHE) (143), (RoBERTa + BERT + ALBERT) (175), (ESM-2 + PSSM) (157), (ESM-2 + MolFormer) (61), (ESM-1 + BERT) (146), (ELMo + Vision Transformer) (46), (BERT + T5) (201), and (BERT + AlphaFold + IgFold) (169).

Furthermore, among 65 predictive pipelines integrating external classifier, BERT, ESM-1 and T5 based predictive pipelines are most widely explored, followed by transformer, ESM-2 and AlphaFold. Specifically, 11 BERT based predictive pipelines have incorporated seven distinct classifiers (BiLSTM (136, 189), CNN (142, 156, 159, 209), GVP (186), CNN+ BiLSTM + MLP (144), LogR (202), SVM (141), Transformer + GAT-CNN (43)), 11 T5 language model based predictive pipelines have employed six unique classifiers (CNN (14, 165, 208, 211), GCN (199), GMM (220), XGBoost+CAPT5 (35), LogR (215, 224), RF (164)), and 11 ESM-1 based predictive pipelines have incorporated six distinct classifiers (CNN (13, 22, 152, 153), BiLSTM (166), GAN (198), GCN (44, 204), GNN (206), GVPConv (151)). In addition, seven predictive pipelines have leveraged transformer with four classifiers (CNN (18), GRU (167), Inductive Matrix Completion Algorithm (179), ResNet (155)) and four ESM-2 based predictive pipelines have employed four classifiers (BiLSTM (160), CNN (184), GAT (36), RF (197)). Moreover, two predictive pipelines have utilized AlphaFold with GCN (221), AlphaFold2 with MLP (161) and RoBERTa with LogR (42) classifiers, respectively. Apart from this, 17 predictive pipelines reap benefits of two or more language models or other embedding or sequence encoding approaches with external classifiers such as: (AlphaFold + BERT) with SVM classifier (217), (ESM-1 + ESM-2 + T5) with BiLSTM + BiGRU classifier (60), (ESM-1b + AlphaFold) with SVM classifier (12), (BERT + TransE) with GNN classifier (148), (Transformer + BERT) with CNN + MLP classifier (163), (OHE + BERT) with CNN + MLP classifier (64), (Word2Vec + BERT) with SnTCN classifier (168), (BERT + RoBERTa) with GCN + MLP classifier (176), (ESM-1 + Prottrans) with CNN classifier (191), (ESM-2 + T5) with GNNs classifier (192), (ESM-2 + BERT) with DNNS classifier (196), (Transformer-XL + XLNet + BERT + ALBERT + ELECTRA + T5) with CNN classifier (212), (BERT + PSSM) with CNN classifier (219), (BERT + GNN) with GBDT classifier (223), (ESM-2 + AlphaFold) with BiLSTM + GNN classifier (225), and (Integer Encoding + RoBERTa) with BiGRU classifier (62).

Table 3 presents 15 distinct language models and their variants used for the development of AI-driven predictive pipelines for 63 different protein sequence analysis tasks. Additionally, it provides details on number of layers in language model architecture, including number of encoders or decoders and their respective layers. These language models are categorized into five groups based on their architectures. These categories include encoder-decoder, encoder-only, decoder-only, special transformer variants and Vision Transformer Models (380).

**Table 3.** A summary of 15 contemporary language models utilized in protein sequence analysis tasks.

Architecture Type	Language model, Release Year	Language Model Variants	Number of Layers in Encoders	Number of Layers in Decoders
Encoder-Decoder	T5, (381), 2020	Small	6	6
		Base	12	12
		Large	24	24
		T5-3B	24	24
		T5-11B	24	24
	Transformer, (382), 2017	Base	6	6
		Big	6	6
Encoder-Only	ALBERT, (383), 2020	Base	12	_
		Large	24	_
		xLarge	24	_
		xxLarge	12	_
	BERT, (384), 2019	Base	12	_
		Large	24	_
	RoBERTa, (385), 2019	Base	12	_
		Large	24	_
	XL-Net, (386), 2019	Base	12	_
		Large	24	_
Decoder-Only	GPT, 2018	GPT-1 (387)	_	12
		GPT-2 small (388)	_	12
		GPT-2 medium (388)	_	24
		GPT-2 Large (388)	_	36
		GPT-3 (389)	_	96
		GPT-4 (390)	_	120
Special Transformer Variants	IgFold, (391), 2023	_	Graph Transformer Layers=4	
	ESM-2 (392), 2022	ESM-2 (8M)	6	_
		ESM-2 (35M)	12	_
		ESM-2 (150M)	30	_
		ESM-2 (650M)	33	_
		ESM-2 (3B)	36	_
		ESM-2 (15B)	48	_
		ESMFold	48	_
	AlphaFold, (393), 2021	AlphaFold v2	220 Residual convolutional blocks (each block: 6 layers)	
	ESM-1, 2021	ESM-1b (213)	33	_
		ESM-1v (222)	33	_
		ESM-MSA/ MSA Transformer (394)	12	_
	AlphaFold 2, 2021	_	48 Evoformer Blocks, 8 Structure Blocks	
	MolFormer, 2022 (395)	_	Transformer trained on SMILES sequences of 1.1 billion unlabelled molecules	
	Graph Transformer Network, 2022 (396)	_	5 Graph Transformer Layers	
Vision Transformer Models	Vision Transformer, (380), 2021	Base	12	_
		Large	24	_
		Huge	32	_

## Experimental settings and evaluation measures

Evaluation of AI-driven protein sequence analysis of predictive pipelines undergoes through two distinct experimental settings: 1) k-fold cross-validation (28, 30) and 2) Train-test split (15, 143). k-fold cross-validation is an iterative process that divides dataset into k equal sized folds. Among k folds, *k* − 1 folds are used for training and remaining one fold is reserved for testing. For deep learning predictive pipelines, an additional set, known as validation set is developed which is usually 10% of training data. This validation set helps in tuning hyperparameters. On the other hand, train-test split setting splits dataset into two sets namely, a) train set and b) test set. Typically, train set contains majority portion of dataset (usually 70-80%) whereas, test set contains remaining portion of dataset (20-30%). Similar to k-fold cross-validation, train-test split also uses validation set for deep learning predictive pipelines.

Depending on task type, evaluation measures are categorized into four classes: (1) Binary (280)/Multi-class classification (397), (2) Multi-label classification (28), (3) Regression (184), and (4) Clustering (184). Following subsections provide an in-depth insight for all four types of evaluation measures.

### Evaluation measures for binary/multi-class classification

**Figure 7.**
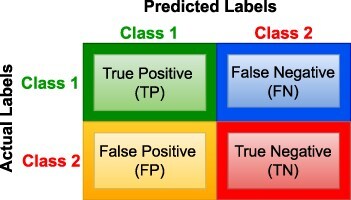
Illustration of confusion matrix.

Predictive pipelines for binary/multi-class classification categorizes instances into pre-defined classes. For evaluation of these predictive pipelines, most commonly utilized evaluation measures are accuracy(247), precision (247), recall (247), F1-score (247), specificity (264), and Matthews correlation coefficient (321). To compute performance values of predictive pipelines through these measures, confusion matrix is used which consists of four entities: true positive (TP), true negative (TN), false positive (FP), and false negative (FN). Figure 7 illustrates confusion matrix encompassing all four entities graphical representation.

Figure 7 depicts that TP and TN signify correct positive and negative predictions, whereas FP and FN indicate incorrect positive and negative predictions, respectively. Accuracy (Acc) (247) is ratio of correctly predicted instances out of total instances, whereas precision (Pr) (247) is proportion of true positive predictions out of all positive predictions. Recall (R) (247) calculates true positive predictions out of all actual positives and F1-score (247) is harmonic mean of precision and recall. Specificity (Sp) (264) measures true negative predictions out of all actual negatives. Matthews correlation coefficient (MCC) (321) is a ratio of difference between correct predicted class and incorrect predicted class to square root of product of all four entities of confusion matrix. Equation 6 embodies mathematical expressions used to calculate aforementioned measures.


(6) $$ f(x)_{\text{balanced}}= \left\{\begin{matrix} Acc = \frac{TP+TN}{TP+FP+TN+FN} & \\[4pt] Pr = \frac{TP}{TP+FP} & \\[4pt] R = \frac{TP}{TP+FN} & \\[4pt] F1-score =\frac{2 \times Pr \times R}{Pr + R} & \\[4pt] Sp = \frac{TN}{TN+FP} & \\[4pt] MCC = \frac{(TP \times TN) - (FP \times FN)}{\sqrt{(TP+FP)(TP+FN)(TN+FP)(TN+FN)}} & \\[4pt] \end{matrix}\right. $$

An extensive study of existing protein sequence analysis predictors reveals that most widely used evaluation measures for balanced datasets are accuracy, precision, recall, specificity, F1-score and Matthews correlation coefficient (MCC). For highly imbalanced datasets, micro, macro, and weighted variants of these measures are employed. To overcome class imbalance issue, weighted score computes weighted average of these measures for each class such as weighted precision (267), weighted recall (267) and weighted F1-score (267). Similarly, Macro score (398) computes average sum of these measures namely precision, recall, all F1-score across all classes. Beyond weighted and macro scores, micro precision aggregates true positives and false positive, Micro recall (38) is a proportion of true positives and false negatives and micro F1-score (38) aggregates F1-score across all classes, respectively. Equation 7 depicts these measures mathematical expressions in terms of micro, macro and weighted scores.


(7) $$ f(x)_{\text{imbalanced}}= \left\{\begin{matrix} \text{Weighted Pr} = \frac{\sum_{j=1}^{m} Pr^j \times w^j}{\sum_{j=1}^{m} w^j} & \\[4pt] \text{Weighted R}= \frac{\sum_{j=1}^{m} R^j \times w^j}{\sum_{j=1}^{m} w^j} & \\[4pt] \text{Weighted F1} = \frac{\sum_{j=1}^{m} F1^j \times w^j}{\sum_{j=1}^{m} w^j} & \\[4pt] \text{Macro Pr} = \frac{1}{m} \sum_{j=1}^{m} Pr^j & \\[4pt] \text{Macro R} = \frac{1}{m} \sum_{j=1}^{m} R^j & \\[4pt] \text{Macro F1} = \frac{1}{m} \sum_{j=1}^{m} F1^j & \\[4pt] \text{Micro Pr}= \frac{\sum_{j=1}^{m} {TP}^j}{\sum_{j=1}^{m}({TP}^j+{FP}^j)} & \\[4pt] \text{Micro R} = \frac{\sum_{j=1}^{m} {TP}^j}{\sum_{j=1}^{m}({TP}^j+{FN}^j)} & \\[4pt] \text{Micro F1} = \frac{\sum_{j=1}^{m} 2 \times {TP}^j}{\sum_{j=1}^{m}(2 \times {TP}^j+{FP}^j+{FN}^j)} & \\[4pt] \end{matrix}\right. $$

Here, *TP^j^*, *FP^j^* and *FN^j^* denote true positives, false positives and false negatives in class *j*, respectively. Similarly, *Pr^j^*, *R^j^* and $F1^j $ indicate precision, recall and F1-score of class *j*. Here, *w^j^* signifies weight of class *j* where *j* refers to *j*^th^ class among *m* classes.

### Evaluation measures for multi-label classification

Contrary to binary or multi-class classification, predictive pipelines for multi-label classification predict multiple labels of an instance at a time. Hence, some of predicted labels can be either correct, incorrect, all correct or all incorrect. This partial correctness or incorrectness in prediction generates complexity. To overcome these complexities, researchers have proposed diverse evaluation measures namely: precision (Pr) (219), recall (R) (219), accuracy (Acc) (28), F1-score (F1) (219) and hamming loss (HL) (273). Precision measures average of all correctly predicted true positive labels out of all positive predicted labels, whereas recall calculates average of all correctly predicted true positive labels out of actual true labels. Accuracy calculates average of correctly predicted labels to total number of labels, whereas F1-score measures average of precision and recall. Hamming loss quantifies fraction of incorrect labels to total number of labels. Equation 8 embodies mathematical expressions for evaluation measures for multi-label classification.


(8) $$ f(x)_{\text{multi-label}}=\left\{\begin{matrix} Pr =\frac{1}{M}\sum_{j=1}^{M} \frac{\left|A^{j}\wedge P^{j}\right|}{\left|P^{j}\right|}& \\[4pt] R = \frac{1}{M}\sum_{j=1}^{M} \frac{\left|A^{j}\wedge P^{j}\right|}{\left|A^{j}\right|}& \\[4pt] Acc = \frac{1}{M}\sum_{j=1}^{M}\left| \frac{A^{j}\wedge P^{j}}{A^{j}\vee P^{j}}\right| & \\[4pt] F1 = \frac{1}{M}\sum_{j=1}^{M} \frac{2\times\left|Pr(m^j)\times R(m^j)\right|}{\left|Pr(m^j)+ R(m^j)\right|}& \\[4pt] HL = \frac{1}{ML} \sum_{j=1}^{M}\sum_{k=1}^{L}\left [ 1({A_k}^j \neq {P_k}^j ) \right ]& \end{matrix}\right. $$

In Equation 8, *M* represents total number of samples, *m^j^* denotes *j*^th^ sample out of *M* samples. *A^j^* is actual class label and *P^j^* is predicted class label for *m^j^* sample. *L* represents sample length, *k* denotes class index, $\lor$ signifies logical OR operator and $\land$ denotes logical AND operator. $A_k^j $ represents true label of *k*^th^ instance for *j*^th^ label and $P_k^j $ represents predicted label of *k*^th^ instance for *j*^th^ label.

### Evaluation measures of regression

Predictive pipelines for regression tasks predict continuous numerical values instead of discrete labels. Most commonly used evaluation measures for regression based tasks are mean absolute error (MAE) (344), mean squared error (MSE) (63), root mean square error (RMSE) (344), mean bias error (MBE), mean absolute percentage error (MAPE), *R*^2^ (63), relative mean absolute error (rMAE), relative mean square error (rMSE), relative mean bias error (rMBE) and relative root mean square error (rRMSE).

MAE measures average of absolute difference between predicted and actual values, whereas MSE calculates average of squares of errors between predicted and actual values. In contrast, MBE calculates average bias present in predictive pipelines by measuring average difference between predicted and actual value and MAPE determines average of absolute percentage errors between predicted and actual values. RMSE computes standard deviation by averaging squared differences of actual and predicted value which illustrates close clustering of data points around regression line. Lower values of MAE, MSE, MBE, and MAPE indicate better performance of predictive pipelines. *R*^2^ calculates ratio of squared difference between actual and predicted values to squared difference between actual values with its mean. Equation 4 embodies mathematical expressions for evaluation measures for regression.


(9) $$ f(x)_{\text{Regression}}=\left\{\begin{matrix} MAE = \frac{1}{N} \sum_{j=1}^{N} \left | P^j - A^j \right | & \\[6pt] MSE = \frac{1}{N} \sum_{j=1}^{N} \left ( A^j - P^j \right )^2 & \\[6pt] RMSE = \sqrt{\frac{1}{N} \sum_{j=1}^{N} \left ( A^j - P^j \right )^2} & \\[6pt] MBE = \frac{1}{N} \sum_{j=1}^{N} \left (P^j - A^j\right ) & \\[6pt] MAPE = \frac{1}{N} \sum_{j=1}^{N} \left | \frac{P^j - A^j}{A^j} \right | \times 100 & \\[6pt] R^{2} =1- \frac{\sum_{j=1}^{N}\left (P-A \right)^{2}}{\sum_{j=1}^{N} (A-\bar{A} )^{2}} & \\[6pt] \end{matrix}\right. $$

In above-mentioned Equation 4, *N* signifies total number of samples, *A^j^* represents actual value and *P^j^* denotes predicted values. Evaluating relative performance of predictive pipelines can enhance quality of performance by minimizing noise from data. Since data continuously changes and yields different predicted values at different times, relative error of all data points is calculated as an overall percentage. For rMAE, rMSE, rMBE, and rRMSE, percentage error of each matrix is computed relative to average of actual values. Equation 10 depicts mathematical expressions for relative measures.


(10) $$ f(x)_{\text{relative}}=\left\{\begin{matrix} rMAE = \frac{MAE}{\bar{A}} \times 100 & \\[6pt] rMSE = \frac{MSE}{\bar{A}} \times 100 & \\[6pt] rMBE = \frac{MBE}{\bar{A}} \times 100 & \\[6pt] rRMSE = \frac{RMSE}{\bar{A}} \times 100 & \\[6pt] \end{matrix}\right. $$

Here, *j* denotes sample number and $\bar{A}$ is average of overall actual values.

### Evaluation measures of clustering

Clustering tasks related predictive pipelines aim to organize instances into clusters based on their similarity with each other. Higher value of similarity for a cluster signifies instances belongs to that specific cluster. The most commonly used evaluation measures for clustering based predictive pipelines are accuracy (140), normalized mutual information (140), silhouette score (399), Davies-Bouldin index (DBI) (400) and Dunn index(400). Accuracy (Acc) measures average of maximum instances correctly assigned to actual cluster for optimal cluster-label mapping. Normalized mutual information (NMI) determines mutual information between predicted and actual clusters. Higher NMI score indicates that clustering algorithm has successfully uncovered distinct and informative clusters within data. Silhouette score (SS) calculates similarity of instances to its own cluster and other clusters. Its score ranges from −1 to 1, where a higher score indicates better-defined clusters. DBI evaluates sum of distance of instance within-cluster to between-clusters. Dunn index (DI) quantifies ratio of minimum inter-cluster distance to maximum intra-cluster distance. Equation 11 depicts mathematical expressions for these evaluation measures.


(11) $$ f(x)_{\text{clustering}}=\left\{\begin{matrix} Acc=\underset{max}{m} \frac{\sum_{j=1}^{n}1\left\{y_j = m(c_j)\right\}}{n} & \\[6pt] NMI = \frac{I(y_j,c_j)}{\frac{1}{2}\left [ E(y_j)+E(c_j) \right ]} & \\[6pt] SS = \frac{min \left\{d(y_j) \right\} -a(y_j)}{max\left\{min \left\{d(y_j) \right\},a (y_j)\right\}} & \\[6pt] DBI = \frac{1}{n}\sum_{j=1}^{n} \underset{k\neq j}{max}(\frac{\bar{S_j}+\bar{S_k}}{d(c_j,c_k)}) & \\[6pt] DI = \frac{min_{1\leq j< k\leq n}d(c_j,c_k)}{max_{1\leq m\leq n}{d^{\prime}(c)}}& \\[6pt] \end{matrix}\right. $$

In Equation 11, *y_j_* signifies predicted cluster, *c_j_* and *c_k_* indicates *j*^th^ and *k*^th^ clusters among *n* clusters, whereas *m* denotes mapping of cluster-label. Furthermore, *I*(*y_j_*,*c_j_*) indicates mutual information, *E*(*y_j_*) and *E*(*c_j_*) shows entropy of predicted and actual clusters, respectively. *d*(*y_j_*) is average distance from *y_j_* to all points in other cluster and *a*(*y_j_*) is average distance of *y_j_* to all points in other clusters. *d*(*c_j_*,*c_k_*) represents inter-cluster distance between *j* and *k* clusters, $\bar{S}_j$ denotes mean distance from cluster mean for all observations in cluster *j*, whereas $\bar{S}_k$ denotes mean distance from cluster median for all observations in cluster *k*. An in-depth analysis of existing literature for protein sequence analysis demonstrates that most commonly employed evaluation metrics in domain of clustering are accuracy and normalized mutual information.

## Open-source protein sequence analysis predictive models

Publicly accessible source codes for AI-driven protein sequence analysis applications allow researchers to avoid reinventing the wheel. They can leverage existing codes to develop applications for other similar tasks and can incorporate new ideas to refine and elevate the performance of established AI-driven protein sequence analysis applications. This section delves into open-source code availability within the landscape of AI-driven protein sequence analysis. To identify open source codes, we thoroughly analysed 295 research articles, specifically focusing on development of AI-driven protein sequence analysis applications by using two different paradigms: word embeddings and LLMs. Our analysis reveals that, for word embeddings-based AI applications, 52 out of 98 studies made their source codes publicly available. Similarly, for large language models (LLMs)-based AI applications, 102 out of 137 studies made their source codes publicly available. Tables 4 and 5 illustrate details about open-source codes of word embeddings and LLMs based protein sequence analysis applications respectively. These tables also summarises details of representation learning methods, machine/deep learning predictors employed, and link of respective source code.

**Table 4.** Overview of open-source word embedding based protein sequence analysis models in existing studies

Author, Year [ref]	Task Name	Embedding approach	Classifier	Source Code
Akbar et al., 2022 (404)	Anticancer Peptides Prediction	FastText	MLP	https://github.com/shahidakbarcs/cACP-DeepGram
Raza et al., 2023 (168)	Anti-Inflammatory Peptides Prediction	FastText + BERT	TCN	https://github.com/shahidawkum/AIPs-SnTCN
Hamid et al., 2019 (405)	Antimicrobial peptides Prediction	Word2Vec	BiGRU	https://github.com/nafizh/NeuBI
Sarker et al., 2019 (305)	Protein Function Identification	FastText	MLP	https://github.com/facebookresearch/fasttext
Yusuf et al., 2021 (227)	Protein Family Classification	Word2Vec	CNN	https://github.com/CSUBioGroup/DeepPPF
Li et al., 2023 (230)	Essential Proteins Identification	DANE, CNN + BiGRU	MLP	https://github.com/yxinshidai/pro.git
Yue et al., 2022 (232)	Essential Proteins Identification	Node2Vec, DC + BC + CC + EC +SC + SoECC + ClusterC + MNC + LAC + LID	CNN	https://github.com/LionKingAHAU/MBIEP
Zeng et al., 2019 (236)	Essential Proteins Identification	Node2Vec, CNN	MLP	https://github.com/CSUBioGroup/DeepEP
Ho et al., 2020 (57)	Electron Transport Protein Prediction	FastText	SVM	https://github.com/khucnam/FastET
Le et al., 2019 (228)	SNAREs Identification	FastText	CNN	https://github.com/khanhlee/fastSNARE
Hu et al., 2022 (271)	Drug Protein Interaction Prediction	Node2Vec, Word2Vec	BiGRU	https://github.com/AI-bio/multi-task-for-cov
Pan et al., 2021 (30)	Protein Subcellular Locations Identification	Node2Vec	LSTM	https://github.com/xypan1232/node2loc
Wu et al., 2023 (299)	Protein Function Identification	Mashup	SVM	https://github.com/XiaozheHu/melissa
Zhang et al., 2020 (303)	Protein Function Identification	Word2Vec	Bi-LSTM + MCNN	https://github.com/CSUBioGroup/DeepGOA
Wan et al., 2019 (304)	Protein Function Identification	Mashup + Node2Vec	SVM	https://github.com/psipred/STRING2GO
Hou et al., 2021 (306)	Protein submitochondrial localization identification	ELMo	CNN + BiLSTM	https://github.com/houzl3416/iDeepSubMito
Yang et al., 2020 (253)	Nucleic acid-binding Proteins Identification	FastText	Affinity Regression	https://github.com/syang11/ProbeRating
Do et al., 2021 (239)	Protein S-sulfenylation sites detection	FastText	MLP	https://github.com/khanhlee/fastSulf-DNN
Hong et al., 2021 (252)	Residue-Residue Interaction Prediction	Node2Vec, PCP	LogR	https://github.com/liujlg/trimer
Palhamkhani et al., 2023 (260)	Compound-Protein Interaction Prediction	Node2Vec, OHE	CNN	https://github.com/farnazkhani/Deep_Compound_Net
Chen et al., 2023 (261)	Compound-Protein Interaction Prediction	Drug Morgan fingerprint (RDkit), GraRep	XGBoost	https://github.com/gitlearning518/GraphCPIs
Watanabe et al., 2021 (263)	Compound-Protein Interaction Prediction	CNN, ECFP, Node2Vec	MLP	https://github.com/Njk-901aru/multi_DTI.git
Amiri et al., 2023 (401)	Multiple Interaction Types Prediction	Node2Vec	XGBoost	https://github.com/elmira-amiri/DT2VecPlus
Xia et al., 2022 (51)	Virus-Host Protein Interaction Prediction	Node2Vec	CCA	https://github.com/LittleBird120/DiseaseGenePredicition
Kang et al., 2023 (38)	Protein–Protein Interaction Prediction	Node2Vec	GNN + MLP	https://github.com/Xinchaow/BBLN
Ozger et al., 2023 (39)	Protein–Protein Interaction Prediction	TFIDF	SVM	http://github.com/ZBaOz/Sars-CoV-2-Protein-Interaction-Prediction
Zheng et al., 2023 (40)	Protein–Protein Interaction Prediction	Word2Vec + Node2Vec	LogR	https://github.com/zjy1125/DeepAraPPI
Su et al., 2022 (241)	Protein–Protein Interaction Prediction	LINE	RF	https://github.com/rasmusbergpalm/DeepLearnToolbox, https://github.com/hashemifar/DPPI, https://github.com/Blair1213/LPPI, https://github.com/muhao chen/seq_ppi.git
Ieremie et al., 2022 (149)	Protein–Protein Interaction Prediction	Node2Vec	Transformer	https://github.com/Ieremie/TransformerGO
Su et al., 2021 (244)	Protein–Protein Interaction Prediction	DeepWalk + Node2Vec	LogR	https://github.com/Blair1213/LPPI
Zhang et al., 2020 (245)	Protein–Protein Interaction Prediction	Node2Vec	LSTM	https://github.com/ZhuMan94/protein2vec
Gavali et al., 2022 (407)	Kinase–substrate Interaction Prediction	DeepWalk	RF	https://github.com/udel-cbcb/ikg_v2_public, https://github.com/udel-cbcb/triple_walk.git
Huang et al., 2020 (402)	Molecular Interaction Prediction	Node2Vec	GNN	https://github.com/kexinhuang12345/SkipGNN
Balogh et al., 2022 (266)	Missing Link Prediction	Node2Vec	cGAN	https://github.com/semmelweis-pharmacology/ppi_pred
Mallick et al., 2019 (270)	Missing Link Prediction	Topo2Vec	RF	https://github.com/th3-buNNy-gUy/Topo2vec/tree/master
Wang et al., 2022 (248)	Protein Complexes Identification	Node2Vec	Ensemble (LR +BR+ DTR+SVM)	https://github.com/RongquanWang/ELF-DPC
Meng et al., 2021 (249)	Protein Complexes Identification	DeepWalk	Core Attachments based Clustering Algorithm	https://github.com/XiangmaoMeng/DPCMNE
Ostrovsky et al., 2021 (311)	B/T Cell Receptor Sequences analysis	Word2Vec	LogR	https://bitbucket.org/yaarilab/immune2vec_model/src/master
Qiu et al., 2021 (406)	G-protein coupled receptors identification	Word2Vec	XGBoost	https://github.com/454 170 054/EMCBOW-GPCR
Xu et al., 2021 (287)	Disease Gene Prediction	Word2Vec + Node2Vec	CNN	https://github.com/apple/turicreate
Vilela et al., 2023 (276)	Disease Genes Prediction	ComplEx + DistMult + TransE	_	https://github.com/jrderuiter/pybiomart
Chu et al., 2023 (275)	Disease Genes Prediction	Struc2Vec	XGBoost	https://github.com/FengLi12/Our-code
Lu et al., 2022 (281)	Disease Genes Prediction	Word2Vec, Node2Vec	SVM	https://github.com/scikit-learn-contrib/boruta_py
Luo et al., 2019 (291)	Disease Genes Prediction	Node2Vec	DBN	https://github.com/luoping1004/dgMDL
Yang et al., 2018 (293)	Disease Genes Prediction	Node2Vec, LINE	Cosine Similarity	https://github.com/yangkuoone/HerGePred
Nguyen et al., 2020 (308)	Tumour Necrosis Factors Identification	FastText	SVM	https://github.com/khucnam/TNFPred
Guo et al., 2020 (403)	Protein miRNA Interaction	Node2Vec	RF	https://github.com/CocoGzh/MAN-1.0
Heinzinger et al., 2019 (33)	Protein Subcellular Localization Identification, Structure Prediction	ELMo	CNN	https://github.com/Rostlab/SeqVec
Albu et al., 2023 (240)	Protein–Protein Interaction Prediction	ELMo	LogR	https://github.com/alexandraalbu/MM-StackEns
Jha et al., 2023 (145)	Protein–Protein Interaction Prediction	ELMo	BERT	https://github.com/jwzhanggy/Graph-Bert, https://github.com/JhaKanchan15/PPI_GBERT
Zhang et al., 2023 (297)	Protein Function Identification	ELMo	_	https://github.com/BIOGOHITSZ/HNetGO
Ali et al., 2023 (298)	Protein Function Identification	ELMo	SVM+LR	https://github.com/pchourasia1/PDB_Plus_LLM_Contact_Map
Chen et al., 2020 (162)	Compound-Protein Interaction Prediction	Word2Vec, RDkit, GCN	Transformer Decoder + MLP	https://github.com/lifanchen-simm/transformerCPI

**Table 5.** Overview of open-source language models based protein sequence analysis models in existing studies

Author, Year [ref]	Task Name	Language Model	Classifier	Pre-trained/Self-train	Source Code
Roche et al., 2024 (157)	Nucleic Acid Binding Protein Identification	ESM-2,PSSM	_	Pre-train	https://github.com/Bhattacharya-Lab/EquiPNAS
Shin et al., 2023 (412)	Protein–Protein Interaction Prediction	Transformer	CNN	Self-train	https://github.com/pnumlb/AptaTrans
Zeng et al., 2023 (160)	Nucleic Acid-Binding Proteins Identification	ESM-2	BiLSTM	Self-train	https://github.com/wwzll123/ESM-NBR
Wang et al., 2022 (419)	Nucleic Acid-Binding Proteins Identification	RoBERTa	_	Self-train	https://github.com/FuxuWang/MHCRoBERTa
Yamaguchi et al., 2022 (423)	Nucleic Acid-Binding Proteins Identification	AlphaFold	CNN + LightGBM	Self-train	https://github.com/google-deepmind/AlphaFold
Liu et al., 2024 (156)	Nucleic Acid-Binding Proteins Identification	BERT	CNN	Pre-train	https://github.com/YAndrewL/clape
Naim et al., 2023 (408)	Nucleic Acid-Binding Proteins Identification	Hybrid (T5 + ESM-1 + ESM-2)	CapsNet	Pre-train	https://github.com/agemagician/ProtTrans/
Yuan et al., 2022 (424)	Nucleic Acid-Binding Proteins Identification	AlphaFold	_	Pre-train	https://github.com/biomed-AI/GraphSite
Liu et al., 2022 (425)	Protein–Protein Binding Site Prediction	AlphaFold	_	Pre-train	https://github.com/Liuzhe30/space-hhblits
Abdin et al., 2022 (413)	Peptide Binding Sites Prediction	Transformer	GNN	Pre-train	https://gitlab.com/oabdin/pepnn
Leem et al., 2022 (427)	B Cell Receptor Sequences Analysis	BERT	_	Self-train	https://github.com/alchemab/antiberta
Yuan et al., 2022 (414)	Compound-Protein Interaction Prediction	Transformer	_	Pre-train	https://github.com/biomed-AI/LMetalSite
Littmann et al., 2021 (440)	Compound-Protein Interaction Prediction	T5	CNN	Pre-train	https://github.com/Rostlab/bindPredict
Weber et al., 2022 (420)	Compound-Protein Interaction Prediction	RoBERTa	_	Pre-train	https://github.com/leonweber/drugprot
Sun et al., 2020 (428)	Compound-Protein Interaction Prediction	BERT	_	Pre-train	https://github.com/CongSun-dlut/CPI_extraction
Filipavicius et al., 2020 (183)	Structure Prediction, Remote Homology Detection, Protein–Protein Binding Site Prediction	RoBERTa	_	Self-train	https://github.com/PaccMann/paccmann_proteomics, https://ibm.ent.box.com/v/paccmann-proteomics-data
Duong et al., 2019 (429)	Protein Function Identification, Protein–Protein Interaction Prediction	BERT	_	Self-train	https://github.com/datduong/EncodeGeneOntology
Zhang et al., 2022 (148)	Protein–Protein Interaction Prediction, Structure Prediction, Contact Prediction	BERT, TransE	GNN	Self-train	https://github.com/zjunlp/OntoProtein
Lanchantin et al., 2021 (47)	Virus Host Protein Interaction Prediction	Transformer	_	Self-train	https://github.com/QData/DeepVHPPI
Liu et al., 2023 (397)	Virus Host Protein Interaction Prediction	ESM-1	_	Pre-train	https://github.com/AMLab-Amsterdam/AttentionDeepMIL/
Luo et al., 2024 (23)	Protein Subcellular Localization Identification	ESM-2	_	Self-train	https://github.com/yujuan-zhang/feature-representation-for-LLMs
Li et al., 2023 (415)	Secreted Peptides Prediction	Transformer	BiGRU	Self-train	https://github.com/Johnsunnn/ExamPle
Melnyk et al., 2023 (169)	Structure Prediction	BERT, AlphaFold, IgFold	_	Self-train	https://github.com/IBM/ReprogBERT
Heinzinger et al., 2023 (216)	Structure Prediction	T5	CNN	Self-train	https://github.com/mheinzinger/ProstT5
Ferruz et al., 2022 (445)	Structure Prediction	GPT	_	Self-train	https://huggingface.co/docs/transformers/main_classes/trainer
Brandes et al., 2022 (188)	Structure Prediction	BERT	_	Self-train	https://github.com/nadavbra/protein_bert
Weissenow et al., 2022 (211)	Structure Prediction	T5	CNN	Pre-train	https://github.com/kWeissenow/EMBER2
Feng et al., 2022 (209)	Structure Prediction	BERT	CNN	Self-train	https://github.com/Cambridge-F/BERT-PPII.git
Villegas et al., 2022 (214)	Structure Prediction	T5	_	Pre-train	https://github.com/amelvim/FoldEmbeddings
Verkuil et al., 2022 (409)	Structure Prediction	ESM-2	_	Pre-train	https://github.com/facebookresearch/esm
Elnaggar et al., 2021 (212)	Structure Prediction	Transformer-XL, XLNet, BERT, ALBERT, ELECTRA, T5	CNN	Self-train	https://github.com/agemagician/ProtTrans/
Meier et al., 2021 (222)	Mutation Effects Prediction	ESM-1v, MSA Transformer	_	Self-train	https://github.com/facebookresearch/esm
Wang et al., 2024 (221)	Mutation Effects Prediction	AlphaFold	GCN	Pre-train	https://github.com/biomed-AI/DeepMutSol
Tzavella et al., 2023 (220)	Mutation Prediction	T5	GMM	Pre-train	https://github.com/KonstantinaT/D2Deep/
Ma et al., 2024 (36)	Protein–Protein Interaction Prediction	ESM-2	GAT	Pre-train	https://github.com/Wenjian-Ma/CollaPPI
Dang et al., 2024 (35)	Protein–Protein Interaction Prediction	T5	XGBoost + CAPT5	Self-train	https://github.com/aidantee/xCAPT5
Zhang et al., 2024 (430)	Protein–Protein Interaction Prediction	BERT	_	Self-train	https://github.com/Freshwind-Bioinformatics/TABR-BERT
Kang et al., 2023 (44)	Protein–Protein Interaction Prediction	ESM-1	GAT	Pre-train	https://github.com/1 075 793 472/AFTGAN
Nambiar et al., 2023 (42)	Protein–Protein Interaction Prediction	RoBERTa	LogR	Self-train	https://github.com/annambiar/PRoBERTa
Mou et al., 2023 (43)	Protein–Protein Interaction Prediction	BERT	Transformer + GAT-CNN	Pre-train	https://github.com/idrblab/EnsemPPIS
Dang et al., 2023 (441)	Protein–Protein Interaction Prediction	T5	XGBoost	Pre-train	https://github.com/anhvt00/MCAPS
Strokach et al., 2021 (223)	Protein–Protein Interaction Prediction	BERT, GNN	GBDT	Pre-train	https://gitlab.com/elaspic/elaspic2
Wang et al., 2020 (431)	Protein–Protein Interaction Prediction	BERT	BiLSTM	Pre-train	https://github.com/dlutwy/ppim
Zhou et al., 2019 (416)	Protein–Protein Interaction Prediction	Transformer	_	Self-train	https://github.com/thunlp/Fast-TransX
Gong et al., 2023 (184)	Stability Prediction	ESM-2	CNN	Pre-train	https://github.com/FPPGroup/THPLM
Rives et al., 2021 (213)	Remote Homology Detection, Structure Prediction, Contact Prediction	Transformer	_	Self-train	https://github.com/facebookresearch/esm
Xiao et al., 2021 (154)	Contact Prediction, Remote Homology Detection, Structure Prediction, Fluorescence Prediction, Stability Prediction	BERT	_	Self-train	https://github.com/THUDM/ProteinLM
Rao et al., 2019 (155)	Structure Prediction, Contact Prediction, Fluorescence Prediction, Stability Prediction, Remote Homology Detection	Transformer	ResNet	Self-train	https://github.com/songlab-cal/tape
Elnaggar et al., 2023 (27)	Structure Prediction, Contact Prediction, Fold Prediction, Fluorescence Prediction, Solubility Prediction, Protein Subcellular Localization Identification	T5	_	Self-train	https://github.com/agemagician/Ankh
Xu et al., 2023 (60)	Structure Prediction, Intrinsically disordered protein (IDP) identification	ESM-2	_	Pre-train	https://github.com/xu-shi-jie/idp-elm
Cheng et al., 2021 (432)	Protein–Protein Interaction Prediction	BERT	_	Self-train	https://github.com/s6juncheng/BERTMHC
Huang et al., 2023 (433)	Drug-Target Interaction Prediction	BERT, MPNN	_	Pre-train	https://github.com/huangyixian666/CapBM-DTI
Chen et al., 2023 (442)	Drug-Target Interaction Prediction	ESM-1	GCN	Pre-train	https://github.com/Chenjxjx/drug-target-prediction
Zhang et al., 2023 (417)	Drug-Target Interaction Prediction	Transformer	_	Self-train	https://github.com/ranzhran/MHTAN-DTI
Kang et al., 2022 (421)	Drug-Target Interaction Prediction	BERT + RoBERTa	_	Pre-train	https://github.com/hskang0906/DTI-Prediction.git
Wang et al., 2022 (185)	Drug-Target Interaction Prediction	Transformer	MLP	Self-train	https://github.com/nick1997a/model
Zheng et al., 2022 (434)	Drug-Target Interaction Prediction	BERT	CNN	Pre-train	https://github.com/agemagician/ProtTrans, https://github.com/Jane4747/DTI-BERT
Kalakoti et al., 2022 (426)	Drug-Target Interaction Prediction	AlphaFold	_	Pre-train	https://github.com/TeamSundar/transDTI
Prihoda et al., 2022 (422)	Drug-Target Interaction Prediction	RoBERTa	_	Self-train	https://github.com/Merck/BioPhi
Chen et al., 2021 (273)	Drug-Target Interaction Prediction	BERT	BiLSTM	Pre-train	https://github.com/Fitnessnlp/DeepEmbedding-DTI
Hwang et al., 2024 (410)	Protein Function Identification	ESM-2 + RoBERTa	LogR	Pre-train	https://github.com/y-hwang/gLM
Pang et al., 2024 (199)	Protein Function Identification	T5	GCN	Pre-train	https://github.com/YihePang/DisoFLAG
Abdin et al., 2024 (411)	Protein Function Identification	ESM-2 + GPT-2	GNN	Pre-train	https://github.com/hadi-abdine/Prot2Text
Pang et al., 2023 (201)	Protein Function Identification	BERT+T5	_	Pre-train	https://github.com/YihePang/IDP-LM
Raza et al., 2023 (168)	Protein Function Identification	Hybrid (Word2Vec, BERT, CTF)	TCN	Pre-train	https://github.com/shahidawkum/AIPs-SnTCN
Pei et al., 2023 (202)	Protein Function Identification	BERT	LogR	Pre-train	https://github.com/zhibinlv/BertThermo
Sun et al., 2023(435)	Anticancer Peptide Identification	BiLSTM, BERT, DPC + BPF + AAC + PAAC	BERT	Pre-train	https://github.com/shunmengfan/ACP-BC/tree/master
Yuan et al., 2023 (203)	Protein Function Identification	T5	_	Pre-train	https://github.com/biomed-AI/SPROF-GO
Zhao et al., 2023 (204)	Protein Function Identification	ESM-1	GCN	Pre-train	https://github.com/CandyPerfect/Master
Hu et al., 2022 (207)	Protein Function Identification	AlphaFold	_	Pre-train	https://github.com/elttaes/Revisiting-PLMs
Rao et al., 2020 (443)	Protein Function Identification	ESM-1	LR	Pre-train	https://github.com/facebookresearch/esm
Singh et al., 2022 (153)	Contact Prediction	ESM-1	CNN	Pre-train	https://github.com/jas-preet/SPOT-Contact-LM
Si et al., 2023 (152)	Contact Prediction	ESM-1	CNN	Pre-train	https://github.com/ChengfeiYan/DRN-1D2D_Inter
Zhang et al., 2022 (418)	Gene Phenotype Prediction	Transformer	_	Self-train	https://github.com/TingheZhang/T-GEM
Wang et al., 2023 (144)	Lysine Glutarylation Modification Prediction	BERT, PCP, AAindex, BE, BLOSUM62, DDE	CNN + BiLSTM	Pre-train	https://github.com/xwanggroup/Deepro-Glu
Kim et al., 2024 (58)	G-Protein coupled receptors Identification	BERT	_	Pre-train	https://github.com/Andrewkimmm/GPCR-BERT
Shrestha et al., 2024 (19)	Phosphorylation Modification Prediction	Transformer	_	Self-train	https://github.com/pallucs/PhosSer
Xu et al., 2024 (15)	Phosphorylation Modification Prediction	Transformer	_	Pre-train	https://github.com/StatXzy7/PTransIPs
Pakhrin et al., 2023 (17)	Phosphorylation Modification Prediction	T5	_	Pre-train	https://github.com/KCLabMTU/LMPhosSite
Meng et al., 2024 (143)	Non-histone acetylation Modification Prediction	T5	_	Pre-train	https://github.com/TransPTM/TransPTM
Pratyush et al., 2023 (14)	Protein S-nitrosylation Modification Prediction	T5	CNN	Pre-train	https://github.com/KCLabMTU/pLMSNOSite
Alkuhlani et al., 2022 (13)	Glycosylation and Glycation Modification Prediction	ESM-1	CNN	Pre-train	https://github.com/Alhasanalkuhlani/PTG-PLM
Qiao et al., 2022 (436)	Lysine Crotonylation Modification Prediction	BERT	BiLSTM	Pre-train	http://zhulab.org.cn/BERT-Kcr_models/
Pokharel et al., 2022 (20)	Succinylation Modification Prediction	T5	_	Pre-train	https://github.com/KCLabMTU/LMSuccSite
Liu et al., 2022 (142)	Lysine Glycation Modification Prediction	BERT	CNN	Pre-train	https://github.com/yinboliu-git/Gly-ML-BERT-DL
Motmaen et al., 2023 (170)	Peptide-Binding Specificity Prediction	AlphaFold	LogR	Pre-train	https://github.com/phbradley/AlphaFold_finetune
Nallapareddy et al., 2023 (215)	Protein Family Classification	T5	LogR	Pre-train	https://github.com/vam-sin/ CATHe
Wang et at., 2022 (186)	Protein Function Identification, Stability Prediction, Fluorescence Prediction	BERT	_	Pre-train	https://github.com/aws-samples/lm-gvp
Xu et al., 2022 (146)	Protein Function Identification, Stability Prediction, Fluorescence Prediction, Solubility Prediction, Protein Subcellular Localization Identification, Structure Prediction, Contact Prediction, Fold Prediction, Protein–Protein Interaction Prediction	ESM-1, BERT	_	Pre-train	https://github.com/DeepGraphLearning/PEER_Benchmark
Pourreza et al., 2021 (437)	Protein-Phenotype Interaction Prediction	BERT	RNN + CNN	Pre-train	https://github.com/mpourreza/DeepPPPred
Tu et al., 2023 (181)	Prokaryotic Gene Prediction	ESM-1	_	Pre-train	https://github.com/tonytu16/protigeno
Teufel et al., 2022 (438)	Signal Peptides (SPs) Prediction	BERT	_	Pre-train	https://github.com/fteufel/signalp-6.0
Wang et al., 2023 (219)	Subchloroplast Localization Identification	BERT, PSSM	CNN, Transformer	Pre-train	https://github.com/xwanggroup/DaDL-SChlo
Wang et al., 2023 (218)	Protein Submitochondrial Localization Identification	BERT	_	Self-train	https://github.com/Wangbiub/GO-Submito
Shah et al., 2021 (439)	TRP channels Classification	BERT	SVM	Pre-train	https://github.com/Muazzam-Kazmi/TRP-BERT
Haselbeck et al., 2023 (187)	Thermophilicity Prediction	T5	_	Pre-train	https://github.com/grimmlab/ProLaTherm
Haseeb et al., 2023 (189)	Domain Boundary Prediction	BERT	BiLSTM	Self-train	https://github.com/maryam988/BERTDom-Code
Li et al., 2020 (180)	Disease Gene Prediction	BERT	_	Self-train	https://github.com/xzenglab/BertVS
Brandes et al., 2023 (444)	Variant Effects Prediction	ESM-1	_	Pre-train	https://github.com/ntranoslab/esm-variants
Marquet et al., 2022 (224)	Variant Effects Prediction	T5	LogR	Pre-train	https://github.com/Rostlab/VESPA
Gonzales et al., 2023 (164)	Phage-Host Interaction Prediction	T5	RF	Pre-train	https://github.com/bioinfodlsu/phage-host-prediction

An extensive analysis of Table 4 reveals that in 52 word embedding methods based studies which have follow the open science rules to make their source codes public, a total of 14 unique word embedding methods have been employed. These methods include Node2Vec (30, 38, 51, 149, 232, 236, 245, 248, 252, 260, 266, 271, 281, 291, 293, 401–403), FastText (57, 228, 239, 253, 305, 308, 404), Word2Vec (227, 271, 281, 303, 311, 405, 406), DeepWalk (249, 407), DANE (230), Mashup (299), ELMo (33, 145, 240, 297, 298, 306), GraRep (261), LINE (241), Topo2Vec (270), Struc2Vec (275), ComplEx (276), DistMult (276), and TransE (276). Among all these methods, the highest number of predictive pipelines are developed by utilizing Node2Vec embedding generation method. A total of 19 open-source predictive pipelines utilize Node2vec word embedding method along with a diverse range of machine learning and deep learning predictors. Specifically, two predictive pipelines have used Node2vec with CNN classifier for two different tasks (232, 260), two predictive pipelines have explored Node2vec potential with LSTM classifier (30, 245) and other 15 studies have used Node2vec embedding method along with MLP (236, 263), GNN+MLP (38), SVM (281), DBN (291), Cosine Similarity (293), BiGRU (271), LogR (252), XGBoost (401), CCA (51), GNN (402), cGAN (266), RF (403), Transformer (149) and ensemble (LR+BR+DTR+SVM) (248) classifiers. Similarly, seven predictive pipelines have utilized FastText word embedding method with four unique classifiers (SVM, MLP, CNN, Affinity Regression) for seven different tasks and seven predictive pipelines have explored Word2vec method potential with six unique classifiers (LogR classifier (40, 311), BiGRU (405), CNN (227), Bi-LSTM+MCNN (303), SVM (39), and XGBoost (406)) for six different protein analysis tasks. Furthermore, six predictive pipelines have used ELMo word embedding along with six classifiers namely CNN (33), LogR (240), BERT (145), MLP (297), SVM+LR (298), CNN+BiLSTM (303) for four different tasks. In addition, two predictive pipelines have used DeepWalk word embedding along with RF classifier and clustering algorithm for two tasks. In contrast, remaining predictive pipeline have used 6 unique word embedding approaches namely DANE (230), Mashup (299), GraRep (261), LINE (241), Topo2Vec (270), and Struc2Vec (275) with six different classifiers (MLP, SVM, XGBoost, RF, RF, and XGBoost) for six different tasks.

A holistic view of Table 4 shows that six predictive pipelines have reaped combine benefit of multiple word embedding methods or word embedding method along with language model. Specifically, predictive pipelines have made use of following different combinations of methods: (FastText, BERT) (168), (Mashup, Node2vec) (304), (Word2vec, Node2vec) (40, 287), (ComplEx, DistMult, TransE) (276), and (Node2vec, DeepWalk) (244) is explored for four different tasks

A holistic view of Table 5 shows that in 102 language models based studies, a total of 10 unique language models have been employed. These models include ESM-2 (23, 36, 60, 157, 160, 184, 408–411), Transformer (15, 19, 47, 155, 172, 185, 213, 412–418), RoBERTa (42, 183, 410, 419–422), AlphaFold (169, 170, 207, 221, 423–426), BERT (43, 58, 142, 144, 146, 148, 154, 156, 168, 169, 180, 186, 188, 189, 201, 202, 209, 212, 218, 219, 223, 273, 421, 427–439), T5 (14, 17, 20, 27, 35, 143, 164, 187, 199, 201, 203, 211, 212, 214–216, 220, 224, 408, 440, 441), ESM-1 (13, 44, 146, 152, 153, 181, 204, 222, 397, 408, 442–444), GPT/GPT-2 (411, 445), Transformer-XL (212) and MSA Transformer (222).

In language models driven protein sequence analysis applications, these models have been utilised in two different scenarios: (1) Training from scratch followed by fine-tuning, and (2) Fine-tuning only. In the first scenario, researchers train these models on large protein sequence datasets in an unsupervised manner and further fine-tune them on downstream tasks. In the second scenario, researchers use existing pre-trained models and just fine-tune them for particular downstream tasks.

Moreover, apart from these two scenarios, researchers either use these models directly to develop an end-to-end applications or extract representations from these models to feed into machine learning or deep learning predictors. Overall, 12 distinct pre-trained BERT language models have been utilized in 34 different predictive pipelines. These pipelines have been developed for 12 different tasks namely $B/T$ Cell Receptor Sequences Analysis, Protein Functions Prediction, Protein–Protein Interaction Prediction, Structure Prediction, Contact Prediction, Remote Homology Detection, Fluorescence Prediction, Drug-Protein Interaction Prediction, Protein Submitochondrial localization prediction, Domain Boundary Prediction, Disease Gene Prediction, and Stability Prediction. BERT language model based 12 different pretrained variants are generated by performing unsupervised training of model on different types of data. Table 6 illustrates details of protein data on which BERT and other 35 language models are trained to produce different pretrained versions.

**Table 6.** Summary of uniquely pre-trained language models along with pre-training data for protein sequence analysis tasks

Language Model	Pre-training Data	Language Model	Pre-training Data	Language Model	Pre-training Data	Language Model	Pre-training Data
Leem et al., BERT (427)	57M Human BCR Sequences (42M heavy chains and 15M light chains)	Elnagger et al. BERT (446)	200M Protein Sequences from BDF and UniRef Database	Li et al. Transformer (415)	Peptide sequence from plant SSPs dataset and peptide secondary structure from PHAT web interface	Jumper et al. AlphaFold (393)	90K structures and MSAs from PDB and UniRef100
Duong et al., BERT (429)	Gene Ontology Context	Elnagger et al. BERT (212)	216M Protein Sequences from UniRef 50 and 100 dataset	Zhou et al. Transformer (416)	PPI relations from BioCreative	Yamaguchi et al. AlphaFold (423)	Chen (NUC5tr, NUC5tst, and nonNUC) Dataset
Zhang et al., BERT (148)	ProteinKG25 Dataset (612,483 entities, 4,990,097 triples)	Lee et al. BERT (447)	Biomedical Domain Corpus	Rao et al. Transformer (155)	Pfam Dataset	Wang et al. RoBERTa (419)	565 254 Protein Sequences from Swiss-Prot
Melnyk et al., BERT (169)	Protein and BooksCorpus Data	Chithrananda et al., BERT (448)	SMILES string of molecules and protein sequences	Wang et al. Transformer (185)	Amino acid sequences of proteins and SMILES and drugs from BindingDB	Liu et al. RoBERTa (385)	PubMed + MIMIC-III + BioMed
Brandes et al., BERT (188)	106M Protein derived from UniProtKB/UniRef90	Zhang et al., BERT (449)	556,603 Protein Sequences from UniProt	Zhang et al. Transformer (418)	TCGA Data, and PBMC Data	Prihoda et al. RoBERTa (422)	160GB of text
Zhang et al., BERT (430)	13 529 384 unique TCRCDR3*β* Sequences from TCRdb	Heinzinger et al., T5 (216)	17 million sequences	Shrestha et al. Transformer (19)	Protein Sequences from UniProt	Heinzinge et al. ELMo (33)	UniRef50 Dataset
Xiao et al., BERT (154)	Protein sequences from Pfam	Elnaggar et al., T5 (27)	UniRef50 Dataset, UniRef90 Dataset, UniRef100 Dataset	Zhang et al. Transformer (417)	Metapath dataset	Albu et al. ELMo (240)	Yeast and Human Dataset, Human-2021, Yeast-2017 from PINA, BioGRID, DIP, UniRef50 databases
Feng et al., BERT (209)	PPII helix structure sequences	Deng et al., T5 (35)	2.3M Protein Sequences from BFD100 and Uniref50 Dataset	Ingraham et al. Transformer (450)	717,932 fragment complexes from Scan Protein–Protein interfaces	Peter et al., ELMo (451)	20M words Dataset sampled from Wikipedia and Common Crawl
Cheng et al., BERT (432)	134 281 sequences from Immune Epitope Database	Elnaggar et al., T5 (212)	Sequences from UniRef50 Dataset, BSD Dataset	Ahmed et al. Transformer (446)	6000 Sequences, and S/T and Y Phosphorylation sites from DeepIPS model	Ferruz et al. GPT (445)	4.99M protein sequences
Raza et al., BERT (168)	200M Protein Sequences from BDF and UniRef Database	Elnaggar et al., T5 (446)	Protein Sequences from Uniref50 Dataset, UniRef100 Dataset, and BFD Dataset	Meier et al. ESM 1 (222)	UniRef90 Dataset	Achiam et al. GPT (390)	Uniref50, Uniclust30, and PDB70
Wang et al., BERT (218)	SM424-18 dataset, SubMitoPred dataset, and Mitocarta3.0 dataset	Elnaggar et al., T5 (212)	2.3 million protein sequences from Uniref50 Dataset	Rives et al. ESM 1 (213)	250M Protein Sequences from UniRef50	Jha et al. ELMo + BERT (145)	ELMo on 0.5 million sequences from SwissProt and BERT on 33 million sequences from UniRef 50
Haseeb et al., BERT (189)	UniRef50 Dataset (185 000 Protein Sequences)	Shin et al. Transformer (412)	166,136 Protein Sequences from PDB and 79,890 RNA Sequences from bpRNA	Lin et al., ESM 2 (392)	UniRef and UniProt data	Filipavicius et al., RoBERTa + LongFormer (183)	31M Pfam, 10M STRING, 5M STRING2Seq, 9.53M STRINGLF, 4.76M STRINGLF2Seq, 504K Swiss-Prot Dataset
Li et al., BERT (180)	16,382 sequences from Pfam database	Chen et al. Transformer (162)	Label reversal Dataset: GPCR Dataset, Kinase Dataset	Zeng et al. ESM 2 (160)	43M Protein Sequences from UniRef50	_	_
Devlin et al. BERT (384)	BooksCorpus (800M words) and English Wikipedia (2,500M words)	Lanchantin et al. Transformer (47)	562,253 Protein Sequences	Luo et al. ESM 2 (23)	UniProt Dataset	_	_

## Protein sequence analysis predictive pipelines performance analysis

In AI-driven protein sequence analysis landscape, applications are being developed through three primary approaches: (1) leveraging LLMs, (2) utilizing word embedding methods combined with machine or deep learning predictors, and (3) harnessing domain-specific sequence encoding methods along with machine or deep learning predictors. This review primarily focuses on an in-depth exploration of LLMs and word embeddings-based applications across 63 different protein sequence analysis tasks. However, these approaches may not always achieve state-of-the-art performance for every task. In some cases, domain-specific sequence encoding methods might offer superior performance. To facilitate the development of new predictors, one objective of this paper is to provide the current state-of-the-art performance of predictor for each task. To achieve this, we performed a large-scale literature review on domain-specific encoding methods based applications and included those with the current state-of-the-art performance in the results tables. Tables 7 to 16, contain performance values of all word embedding and LLMs based applications. These tables also include domain specific methods based applications which have state-of-the-art performance values for each task. Moreover, each table represents AI-driven applications performance for a particular goal which contain several protein sequence analysis tasks.

**Table 7.** Protein identification related 7 distinct protein sequence analysis tasks predictive pipelines performance

Task Type	Task Name	Author, Year [ref]	Dataset	Representation learning	Classifier	Performance Evaluation
Multi-class Classification	Protein Family Classification	Idhaya et al., 2023 (312)	Idhaya et al. Dataset	AAC + DPC + Autocorr + CTD + SOCN + QSOC + Pseudo AAC (Amino acid composition and correlation approaches)	Stack (RF, NB, DT)	Acc = 98.49, Precision =96.78, Recall = 95.76, F1-score = 94.79, AUROC = 97.90
		Balamurugan et al., 2023 (140)	Balamurugan et. al Dataset	BERT	_	Acc=0.9902, Weighted F1-score=0.9902, macro F1-score=0.9900, NMI score=0.9845
		Zhou et al., 2022 (226)	POG	Word2Vec	CNN	POG Dataset: Acc = 81.42, Precision = 77.57, Recall = 80.67, F1-score = 77.32, MCC = 81.38
		S**hah et al., 2021 (141)**	**GLUT Dataset, SGLT Dataset, SWEET Dataset**	**BERT**	**SVM**	**1. GLUT: Sn=91.18, Sp=93.98, Acc=92.43, MCC=0.85; 2. SGLT: Sn=88.89, Sp=100, Acc=97.30, MCC=0.93; 3. SWEET: Sn=84.21, Sp=95.24, Acc=92.97, MCC=0.79**
		**Yusuf et al., 2021 (227)**	**GPCR Dataset(families, sub-families, sub-subfamilies), COG Dataset (families)**	**Word2Vec**	**CNN**	**1. GPCR Dataset: Family Acc = 98.89, MCC = 97.62, Sub-family Acc = 90.31, MCC = 88.45, Sub-subfamily Acc = 84.38, MCC =83.09; 2. COG Dataset: Family Acc = 91.83, MCC = 90.31**
Regression	Protein Similarity Prediction	Wang et al., 2023 (229)	STRING-Yeast, STRING-Human Dataset, KGSIM-ALL-Pfam, KGSIM-ALL-PPI	RotatE	MLP	1: AUC-ROC = 0.918, 2: AUC-ROC = 0.912, 3: Pearson Correlation = 0.955, 4: Pearson Correlation = 0.717
Binary Classification	SNARE proteins Identification	**Kha et al., 2022 (56)**	**Le et al. Dataset, Kha et al. Dataset**	**PSSM**	**CNN**	**1. Cross-Validation: Sn=0.845, Sp=0.955, Acc=0.930, MCC=0.800; Independent 1: Sn=0.842, Sp=0.968, Acc=0.955, MCC=0.767; 2. Independent 2: Sn=0.8, Sp=0.952, Acc=0.936, MCC=0.7**
		**Le et al., 2019 (228)**	**Li et al. Dataset**	**FastText**	**CNN**	**Cross-Validation: Sn=96.6, Sp=98.4, Acc=97.5, MCC=0.95; Independent: Sn=88.5, Sp=97, Acc=92.8, MCC=0.86**
Binary Classification	Electron Transport Protein Prediction	**Ho et al., 2020 (57)**	**Nguyen et al. Dataset**	**FastText**	SVM	**Acc > 0.95, Sp > 0.95, Sn > 0.95, MCC=0.96, AUROC=0.986**
Multi-class Classification	G-protein Coupled Receptors Identification	Kim et al., 2024 (58)	Kim et al. Dataset ( NPxxY, CWxP, E/DRY)	BERT	_	E/DRY: Acc=100, NPxxY: Acc=98.05 ± 0.479, CWxP: Acc=86.295 ± 1.010
Binary Classification	Essential Proteins Identification	Saha et al., 2024 (313)	**Saha et al. Dataset yeast PPIN**	**PAAC+PCP+AAC (Amino acid composition and physico-chemical approach)**	**RF**	**Precision = 0.703, Recall = 0.720, F1-score = 0.711, AUC = 0.745**
		**Ye et al., 2024 (314)**	**S. cerevisiae (BioGrid), S. cerevisiae (DIP), S. cerevisiae (Krogan), H. sapiens, M. musculus, C. elegans**	**OHE**	**SVM**	**1. S. cerevisiae (BioGrid): Acc = 0.91, Precision = 0.78, Recall = 0.71; 2. S. cerevisiae (DIP): Acc = 0.90, Precision = 0.81, Recall = 0.74; 3. S. cerevisiae (Krogan): Acc = 0.88, Precision = 0.78, Recall = 0.81; 4. H. sapiens: Acc = 0.88, Precision = 0.78, Recall = 0.81; 5. M. musculus: Acc = 0.78, Precision = 0.60, Recall = 0.69; 6. C. elegans: Acc = 0.91, Precision = 0.46, Recall = 0.57**
		Inzamam et al., 2023 (315)	Inzamam et al. S. Cerevisiae Dataset, Inzamam et al. E. coli Dataset	BC + CC + DC + EC + LAC + NC + SC + IC + PC1 + PC2 + P3, and three different P values of gene expression (Topological and biological features)	RF	1: Acc = 93.43, Recall = 0.9604, Specificity = 0.9195, Precision = 0.8612, NPV = 0.8920, FPR = 0.0840, MCC = 0.8809, F1-Score = 0.9081; 2: Acc = 87.40, Recall = 0.9700, Specificity = 0.8900, PPV = 0.7293, F1-score = 0.8326
		Li et al., 2023 (230)	BioGRID Dataset, DIP Dataset	DANE	CNN+BiGRU	1: Acc=0.901, Precision=0.841, Recall=0.743, F1-score= 0.783, AUC= 0.842; 2: Acc=0.910, Precision=0.847, Recall=0.754, F1-score=0.792, AUC=0.851
		Lu et al., 2023 (231)	Lu et al. Dataset	Node2Vec, TCN	MLP	Precision = 0.72, Recall = 0.74, F -measure = 0.73, Acc = 0.88, AUC = 0.921, AUPR = 0.755
		Hossain et al., 2023 (316)	E. coli Dataset	SG + DG + EV + IC + LAC + BC + CC + NC + three different Log Fold changes and three different P values of gene expression (Topological and biological features)	RF	E.coli Dataset: Acc = 87.65, Recall = 0.9822, Sp = 0.7250, Precision = 0.8366, NPV = 96.61, FPR =27.50, MCC = 75.35, F1-score = 0.9036
		**Yue et al., 2022 (232)**	**Yue et al. S. Cerevisiae Dataset**	**Node2Vec, , DC + BC + CC + EC +SC + SoECC + ClusterC + MNC + LAC + LID (Topological and biological features)**	**CNN**	**Acc = 0.9048, Precision=0.7306, Recall= 0.7885, F1 score=0.7585, Specifcity=0.9320, NPV=0.9496**
		Cai et al., 2022 (59)	Cai et al. S. Cerevisiae Dataset	GEMSEC	CNN	Acc = 0.818, Precision = 0.680, Recall = 0.433, F-measure=0.529, AUC=0.802
		Zhang et al., 2022 (317)	Zhang et al. S. Cerevisiae Dataset	OHE	GCN	Acc = 0.8420, Precision = 0.6667, Recall = 0.6180, F-measure = 0.6414, AUC = 0.7680
		**Zeng et al., 2021 (318)**	**Zeng et al. Dataset**	**PseAAC**	**Ensemble GBDT**	**Acc = 0.727, Precision = 0.704, Recall = 0.784, F-measure = 0.742, AUC = 0.816, AUPR = 0.814**
		Wang et al., 2020 (233)	Wang et al. S. Cerevisiae Dataset	Node2Vec	XGBoost	Acc = 0.82, Precision = 0.60, Recall = 0.58, F-score = 0.59, AUC = 0.82
		Wang et al., 2020 (234)	Wang et al. S. Cerevisiae Dataset	Node2Vec	XGBoost	Acc=0.82, Precision=0.60, Recall=0.58, F-score=0.59, AUC=0.82
		Zeng et al., 2019 (235)	Zeng et al. Dataset	Node2Vec	BiLSTM	Acc = 0.85, Precision = 0.68, Recall = 0.50, F-measure = 0.58, AUC = 0.83
		Zeng et al., 2019 (236)	Zeng et al. Dataset	Node2Vec, CNN	MLP	Acc = 0.826, Precision = 0.58, Recall = 0.52, F-measure=0.55, AUC=0.82
		Zeng et al., 2018 (237)	Zeng et al. Dataset	Node2Vec, CNN	MLP	Acc = 0.823, Precision = 0.58, Recall = 0.52, F-measure = 0.55, AUC = 0.81
Binary Classification	Intrinsically disordered protein (IDP) identification	**Xu et al., 2024 (60)**	**TE82 Dataset**	**ESM-1 + ESM-2 + T5**	**BiLSTM + BiGRU**	**AUROC=0.8469, F1-score=0.6325, MCC=0.4814, Recall=0.8344, Precision=0.5092**

Table 7 provides performance metrics for 25 AI-driven (language models, word embeddings, domain specific) applications that are designed for protein identification goal related tasks. This goal oriented binary/multi class classification and similarity prediction applications are developed by utilizing 18 unique representation learning approaches and 12 unique classifiers. The representation learning methods include BERT, Word2vec, RotatE, PSSM, FastText, DANE, Node2vec, OHE, PseAA, ESM-1, ESM-2, T5, TCN, GEMSEC, CNN, amino acid composition and correlation approaches, amino acid composition and physico-chemical properties based approaches and topological and biological features based approaches. The classifiers include MLP, NB, DT, BERT with self-classifier, CNN, SVM, RF, GCN, BiGRU, ensemble GBDT, XGBoost, and BiLSTM.

Among all representation learning approaches, Node2vec is the most commonly used representation followed by BERT, Word2Vec, and FastText. Specifically, potential of Node2vec is explored with XGBoost (233, 234), and BiLSTM (235) classifiers for essential protein identification. Also combined representation from Node2Vec with CNN (236, 237) and Node2Vec with TCN (231) are used with MLP classifier for for essential protein identification. Despite being used in seven predictive pipelines for same task, not a single Node2vec based predictive pipeline have achieved state-of-the-art performance. Furthermore, BERT is used with SVM (141) and a self-classifiers (140) for protein family classification, whereas potential of BERT is explored with a self-classifier for G-protein coupled receptors identification and have achieved state-of-the-art performance (58). Beyond Node2vec and BERT, Word2vec representation is used with CNN classifier for protein family classification (226, 227). In addition, RotatE representation learning method is used with MLP classifier for protein similarity prediction and have achieved state-of-the-art performance (229). Apart from Node2vec, BERT and Word2vec approaches, FastText is used with CNN (228) and SVM (57) classifiers for SNARE and electron transport protein identification whereas combined representation from ESM 1, ESM 2 and T5 language model is utilized with hybrid (BiLSTM+BiGRU) classifier for intrinsically disordered protein identification (60). Additionally, GEMSEC with CNN classifier (59), DANE with hybrid (CNN+BiGRU) classifier (230), PseAAC with ensemble GBDT classifier (318), and OHE with GCN (317) and SVM (314) classifiers are used for essential protein identification. In contrast, amino acid composition and physico-chemical properties based approaches (313) and topological and biological features based approached (315, 316) are used with RF classifier for essential protein identification. In addition, Yue et al. (232) predictor is developed by using topolgical and biological features based approaches with a CNN classifier. Moreover, potential of amino acid composition and correlation based approaches is explored with stacked (RF+NB+DT) classifier for protein family classification and have achieved state-of-the-art performance (312). Similarly, PSSM representation is employed with CNN classifier for SNARE protein identification and achieved state-of-the-art performance (56). From all tasks in this goal, three tasks namely protein similarity prediction, SNARE protein, and intrinsically disordered protein identification offers some room for improvement. An in-depth analysis of these studies reveals that utilizing physico-chemical properties and occurrence frequencies based representation learning approaches combined with ensemble classifiers, can improve performance of under-performing tasks.

Table 8 presents performance metrics for 17 AI-driven applications including language models, word embeddings, and domain-specific based predictive pipelines which are designed for tasks related to protein modification prediction. This goal is oriented to binary classification applications that are developed by utilizing 11 unique representation learning approaches, and nine unique classifiers. The representation learning methods include BERT (21, 142, 144), ESM-1 (12, 13, 22), AlphaFold (12), T5 (14, 17, 20, 143), Transformer (15, 18, 19), integer encoding (16), Amino acid occurrence frequency based approaches (452), FastText (239), amino acid based approaches (144), ELMo (238), and GloVe (238). The classifiers include CNN, SVM, MLP, BiGRU, BiLSTM, Stacked LSTM, and self-classifiers of Transformer, T5, and BERT

**Table 8.** Protein modification prediction related nine distinct protein sequence analysis tasks predictive pipelines performance

Task Type	Task Name	Author, Year [ref]	Dataset	Representation learning	Classifier	Performance Evaluation
Binary Classification	Glycosylation and Glycation Modification Prediction	**Hou et al., 2023 (12)**	**N-GlyDE Dataset, N-GlycositeAtlas**	**ESM-1 + AlphaFold**	**SVM**	**1. MCC=0.736, Sp=0.70, Sn=0.975, Acc=0.884, AUROC=0.946; 2. MCC=0.804, Sp=0.928, Sn=0.875, Acc=0.902, AUROC=0.976**
		Liu et al., 2022 (142)	Kgly Dataset	BERT	CNN	Sn=0.595, Sp=0.595, Precision=0.598, MCC=0.192, Acc=0.595, AUROC=0.640
		**Alkuhlani et al., 2022 (13)**	**Ngly Dataset, Kgly Dataset**	**ESM-1**	**CNN**	**Ngly Dataset: Cross-Validation: Acc=0.922 ± 0.01, Recall=0.984 ± 0.009, Precision=0.875 ± 0.018, F1-score=0.927 ± 0.011, AUROC=0.922 ± 0.008, MCC=0.851 ± 0.017; Independent Test: Acc=0.965, Recall=1, Precision=0.852, F1-score=0.920, AUROC=0.978, MCC=0.902; Kgly Dataset: Cross-Validation: Acc=0.599 ± 0.009, Recall=0.563 ± 0.035, Precision=0.604 ± 0.009, F1-score=0.583 ± 0.018, AUROC=0.599 ± 0.008, MCC=0.199 ± 0.017; Independent Test: Acc=0.64, Recall=0.67, Precision=0.64, F1-score=0.65, AUROC=0.64, MCC=0.28**
Binary Classification	Protein S-nitrosylation Modification Prediction	**Pratyush et al., 2023 (14)**	**DeepNitro Dataset**	**T5**	**CNN**	**Cross-Validation: Acc=0.727 ± 0.017, Sn=0.769 ± 0.016, Sp=0.685 ± 0.033, MCC=0.4573 ± 0.032; Independent Test: Acc=0.769, Sn=0.735, Sp=0.773, MCC=0.340, AUROC=0.754**
Binary Classification	Phosphorylation Modification Prediction	**Xu et al., 2024 (15)**	**Xu et al. Datasets (S/T, Y)**	**Transformer**	_	**S/T: Acc=0.8438, Sn=0.8554, Sp=0.8323, MCC=0.6879, AUROC=0.9232; Y: Acc=0.9286, Sn=0.9524, Sp=0.9048, MCC=0.8581, AUROC=0.9683**
		**Song et al., 2024 (16)**	**Song et al. Mouse Phosphorylation Datasets (S, T, Y), Song et al. PPA Test Datasets (S, T, Y)**	**Integer Encoding**	**CNN**	**1. S: Sp=84.01, Sn=66.98, AUROC=0.831, MCC=0.511, F1-score=0.672; T: Sp=87.79, Sn=50.91, AUROC=0.775, MCC=0.416, F1-score=0.566; Y: Sp=75.68, Sn=50.00, AUROC=0.658, MCC=0.262, F1-score=0.524; 2. S: Sn=61.15, Sp=81.36, AUROC=0.788, MCC=0.443, F1-score=0.688; T: Sn=48.93, Sp=75.68, AUROC=0.691, MCC=0.254, F1-score=0.565; Y: Sn=0.5956, Sp=60.59, AUROC=0.625, MCC=0.201, F1-score=0.599**
		**Pakhrin et al., 2023 (17)**	**Guo et al. Datasets (S/T, Y)**	**T5**	_	**Cross-Validation: S/T: MCC=0.502 ± 0.004, Precision=0.766 ± 0.006, Recall=0.721 ± 0.007, F1-score=0.743 ± 0.002; Independent Test: S/T: MCC=0.3905, Precision=0.3878, Recall=0.6712, F1-score=0.4915; Y: MCC=0.2984, Precision=0.3490, Recall=0.6203, F1-score=0.4467**
		Wang et al., 2022 (18)	Wang et al. P.ELM Datasets (S, T, Y), Wang et al. PPA Datasets (S, T, Y)	Transformer	CNN	1. Cross-Validation: S: Sn=80.56, Sp=75.80, MCC=0.564, AUROC=0.858; T: Sn=76.54, Sp=74.70, MCC=0.512, AUROC=0.834; Y: Sn=61.99, Sp=65.11, MCC=0.271, AUROC=0.695; 2. Independent Test: S: Sn=67.16, Sp=75.89, MCC=0.432, AUROC=0.787; T: Sn=47.32, Sp=76.22, MCC=0.246, AUROC=0.672; Y: Sn=38.52, Sp=72.30, MCC=0.115, AUROC=0.601
Binary Classification	Serine phosphorylation Modification Prediction	**Shrestha et al., 2024 (19)**	**Shrestha et al. Dataset**	**Transformer**	_	**Cross-Validation: AUPRC=0.9560, AUROC=0.9459, MCC=0.8488; Independent Test: AUROC=0.93, MCC=0.70, AUPRC=0.94**
Binary Classification	Succinylation Modification Prediction	**Pokharel et al., 2022 (20)**	H**asan et al. Dataset**	**T5**	_	**Cross-Validation: Acc=0.77 ± 0.01, MCC=0.56 ± 0.02, Sn=0.80 ± 0.01, Sp=0.76 ± 0.02; Independent Test: Acc=0.79, MCC=0.36, Sn=0.79, Sp=0.79**
Binary Classification	Lysine phosphoglycerylation Modification Prediction	**Lai et al., 2023 (21)**	**Sohrawordi et al. Dataset**	**BERT**	_	**Acc=99.53, MCC=99.07, Sn=99.21, Sp=99.85**
		**Chandra et al., 2023 (22)**	**Chandra et al. Dataset**	**ESM-1**	**CNN**	**AUROC=0.839**
Binary Classification	Non-histone Acetylation Modification Prediction	**Meng et al., 2023 (143)**	**Meng et al. Dataset**	**T5**	_	**Acc=0.88, F1-score=0.51, MCC=0.45, AUROC=0.83, AUPRC=0.51**
Binary Classification	Protein S-sulfenylation Modification Prediction	Zhang et al., 2023 (452)	Bui et al. Dataset (Train, IND), Zhang et al. Dataset (Train, IND)	AAC + EAAC + GAAC + PCP + BLOSUM62 (Amino acid occurrence frequency based approach)	BiGRU + MLP	1. Train: Acc=96.66%, Sn=99.69%, Sp=93.63%, MCC=0.9350, AUROC=0.9965; IND: Acc=95.91, Sn=99.65%, Sp=92.18%, MCC=0.9211, AUROC=0.9934; 2. Train: Acc=94.75%, Sn=98.71%, Sp=90.79%, MCC=0.8979, AUROC=0.9916; IND: Acc=95.26%, Sn=98.67%, Sp=91.86%, MCC=0.9079, AUROC=0.9896
		**Do et al., 2021 (239)**	**Xu et al. Dataset**	**FastText**	**MLP**	**Sn=85.71%, Sp=69.47%, Acc=77.09%, MCC=0.5554, AUROC=0.833**
Binary Classification	Protein Glutarylation Modification Prediction	**Wang et al., 2023 (144)**	**Wang et al. Dataset**	BERT + (**PCP + AAindex + BE + BLOSUM62 + DDE (amino acid based approaches)**)	**CNN + BiLSTM**	**Acc=0.963, MCC=0.923, Sn=0.954, Sp=0.970, Precision=0.954, AUROC=0.988**
		**Liu et al., 2022 (238)**	**Huang et al. Dataset**	**ELMo + GloVe**	**Stacked LSTM + MLP**	**Acc=0.79, Sp=0.89, Sn=0.59, MCC=0.51**

For this goal, T5 is the most commonly used representation learning approach followed by transformer, BERT and ESM-1. Specifically, T5 language model based representation learning is used with CNN classifier for protein S-nitrosylation modification prediction (14), whereas potential of T5 language model is explored with a self-classifier for phosphorylation modification prediction, succinylation modification prediction and non-histone acetylation modification prediction (17, 20, 143). Among all T5 language model based predictive pipelines, three predictive pipelines have achieved state-of-the-art performance such as T5 representation with CNN classifier for protein S-nitrosylation modification prediction and T5 representation with a self-classifier for succinylation modification prediction and non-histone acetylation modification prediction. Apart from this, transformer is used with CNN for phosphorylation modification prediction (18), while transformer with self-classifier is used for phosphorylation modification prediction and serine phosphorylation modification prediction (15, 19). Amid all predictive pipelines based on transformer language model, transformer based predictive pipelines with self-classifier have attained state-of-the-art results across two tasks including phosphorylation modification prediction and serine phosphorylation modification prediction.

Furthermore, BERT representation learning approach is employed with CNN and a self-classifier for glycosylation, glycation and lysine phosphoglycerylation modification prediction (21, 142), respectively. Among all BERT based predictive pipelines, BERT with self-classifier have achieved state-of-the-art performance for lysine phosphoglycerylation modification prediction. Similarly, ESM-1 language model based representation learning is employed with CNN classifier for glycosylation, glycation and lysine phosphoglycerylation modification prediction (13, 22). ESM-1 language model based predictive pipelines have achieved state-of-the-art mode for glycosylation and glycation modification prediction. Additionally, combined representation from ESM-1 and AlphaFold language models is used with SVM classifier for glycosylation and glycation modification prediction (12). In addition, integer encoding is employed with CNN classifier for phosphorylation modification prediction (16). FastText is used with MLP classifier for protein S-sulfenylation modification prediction (239).

In addition, potential of combined representation from ELMo and GloVe is explored with stacked (LSTM + MLP) classifier for protein glutarylation modification prediction (238). Apart from this, amino acid occurrence frequency based representation learning approaches are explored with BiGRU + MLP classifier for protein S-sulfenylation modification prediction (452). Among all amino acid occurrence frequency based predictive pipelines, amino acid occurrence frequency based approaches with BiGRU classifier have achieved state-of-the-art performance for protein S-sulfenylation modification prediction task. Combined potential of BERT with amino acid based representation learning approaches is used with CNN + BiLSTM classifier for protein glutarylation modification prediction (144) and has achieved state-of-the-art results across protein glutarylation modification prediction task. An extensive analysis of this goal reveals that three tasks namely succinylation, S-nitrosylation, glycosylation and glycation modification prediction offer room for improvement. Considering performance trend of across different goal, potential of shallow neural network based word embeddings namely Word2vec, FastText, GloVe or graph neural network based embedding such as Node2vec, DeepWalk, LINE or HOPE can enhance the performance of under-performing tasks.

Table 9 presents performance metrics for 11 AI-driven applications that are developed using three different approaches namely (1) language models, (2) word embeddings, (3) domain-specific approaches, designed for tasks related to protein interaction prediction. This goal is oriented to interaction applications are developed by utilizing 37 unique representation learning approaches, and 37 unique classifiers. These unique representation learning approaches are T5, ESM-2, BERT, GAT, Node2Vec, TFIDF, Word2Vec, Score Matrix and Physico-chemical properties based approaches, RoBERTa, ESM-1, ELMO, Vision Transformer, PSSM, LINE, FastText, TransE, Transformer, DeepWalk, Physico-Chemical properties based approaches based representations, AlphaFold2, Amino Acid Occurance based representation learning approach, HIN2Vec, VGAEs, RDKit, GCN, CNN, MolFormer, OHE, ResNet, ECFP, Integer Encoding, GNN, SDNE, ANE, RandomWalk, Topo2Vec, and GraRep. In contrast, unique classifiers include XGBoost, CAPT5, GAT, GNN, MLP, SVM, LogR, CNN, Transformer, RF, LSTM, Louvain clustering, GCN, LR, BR, DTR, Core Attachments based clustering method, k-means, GVPConv, ResNet, BiLSTM, GAE, LightGBM, AdaBoost, CCA, MPNN, GRU, Transformer Decoder, BiGRU, cGAN, Affinity Regression and self-classifiers of ESM-1b, BERT, Transformer, ESM-2, T5, and MolFormer.

**Table 9.** Protein interaction prediction related 11 distinct protein sequence analysis tasks predictive pipelines performance

Task Type	Task Name	Author, Year [ref]	Dataset	Representation learning	Classifier	Performance Evaluation
Interaction	Protein–Protein Interaction Prediction	**Dang et al., 2024 (35)**	**1. Martin et al. Dataset 2. Dang et al. Human Dataset 3. Guo et al. Dataset**	**T5**	**Hybrid (XGBoost +CAPT5)**	**1. Acc=97.27+0.12, Precision=97.30+0.24, Recall=97.07+0.2, Sp=97.44 + 0.11, F1-score=97.18+0.25, MCC=94.82 + 0.2 2. Acc=99.77+0.02, Precision=99.75+0.03, Recall=99.75+0.02, Sp=99.80+0.02, F1-score=99.62+0.06, MCC=99.55+0.03 3. Acc=99.76+0.05, Precision=99.76+0.04, Recall=99.75+0.07, Sp=99.77+0.04, F1-score=99.37+0.27, MCC=99.52+0.1**
		**Ma et al., 2024 (36)**	**1. Ma et al. Dataset: Yeast Dataset 2. Multi-Species Dataset 3. Multi-Class Dataset**	**ESM-2**	**GAT**	**1. Acc=98.53, Precision=98.92, Sn=98.12, Sp=98.93, F1-score=98.52, MCC=97.05, AUROC=99.66 2. Acc=99.31, Precision=99.89, Sn=98.73, Sp=99.89, F1-score=99.30, MCC=98.62 3. Acc=57.39, Precision=60.09, F1-score=57.32**
		**Zhang et al., 2024 (37)**	**Zhang et al. Datasets: 1. SHS27k, 2. SHS148k**	**BERT + GAT**	**GNN**	**1. Recall=0.863, Precision=0.896, F1-score=0.879 2. Recall=0.909, Precision=0.936, F1-score=0.921**
		**Kang et al., 2023 (38)**	**Chen et al. Datasets: 1. SHS27k 2. SHS148k**	**Node2Vec**	**GNN + MLP**	**1. micro-F1-score=88.78% 2. micro-F1-score=92.40%**
		**Ozger et al., 2023 (39)**	**Ozger et al. Dataset**	**TFIDF**	**SVM**	**Acc=98.6%**
		**Zheng et al., 2023 (40)**	**Zheng et al. Dataset**	**Word2Vec + Node2Vec**	**LogR**	**AUPRC=0.965**
		Hu et al., 2023 (41)	Murakami et al Datasets: 1. Dset_186 2. Dset_72 Singh et al. Dataset: 3. Dset_164 Zhang et al. Dataset: 4. Dset_448 Li et al. Dataset: 5. Dset_355	PSSM + PRSA + PI + PCP (Score matrix and physico-chemical properties based approaches)	CNN	Dset_448: Acc=0.859, Precision=0.480, Sp=0.919, Sn=0.481, MCC=0.399, F1-score=0.480, AUROC=0.824, AUPRC=0.479 Dset_355: Acc=0.871, Precision=0.460, Sp=0.927, Sn=0.460, MCC=0.387, F1-score=0.460, AUROC=0.822, AUPRC=0.448 Dset_72: Acc=0.851, Precision=0.299, Sp=0.917, Sn=0.299, MCC=0.216, F1-score=0.299, AUROC=0.740, AUPRC=0.254 Dset_164: Acc=0.778, Precision=0.386, Sp=0.864, Sn=0.386, MCC=0.250, F1-score=0.386, AUROC=0.710, AUPRC=0.364 Dset_186: Acc=0.809, Precision=0.373, Sp=0.887, Sn=0.37, MCC=0.260, F1-score=0.373, AUROC=0.732, AUPRC=0.357
		Nambiar et al., 2023 (42)	Nambiar et al. Dataset	RoBERTa	LogR	Acc=0.98, Precision=0.98, Recall=0.99
		**Mou et al., 2023 (43)**	**Zeng et al. Dataset: DeepPPISP Dataset**	**BERT**	Transformer + GAT-CNN	**Acc=0.732, Precision=0.375, Recall=0.532, F1-score=0.440, AUROC=0.719, AUPRC=0.405, MCC=0.277**
		Kang et al., 2023 (44)	Chen et al. Datasets: 1. SHS27k 2. SHS148k	ESM-1	_	1. micro FI-score=0.867 2. micro FI-score=0.920
		**Zhang et al., 2023 (45)**	**Zhang et al. Dataset**	**Node2Vec**	**CNN**	**Acc=0.9836, Sn=0.9837, Sp=0.9815, Precision=0.9816, MCC=0.9651**
		Jha et al., 2023 (46)	1. Human PPI Dataset 2. S. cerevisiae PPI Dataset	ELMo + Vision Transformer	MLP	1. Acc=98, Sn=98.57, Sp=96.74, Precision=98.51, F1-score=98.54, MCC=95.34, AUROC=99.22, AUPRC=99.39 2. Acc=98.75, Sn=98.81, Sp=98.69, Precision=98.69, F1-score=98.75, MCC=97.49, AUROC=99.64, AUPRC=99.67
		**Albu et al., 2023 (240)**	**Albu et al. Dataset**	**ELMo**	**LogR**	**Acc=0.828 ± 0.013, Precision=0.830 ± 0.022, Recall=0.826 ± 0.024, AUROC=0.902 ± 0.011, AUPRC=0.915 ± 0.011**
		**Jha et al., 2023 (145)**	**Jha et al. Datasets: 1. Human Dataset 2. E. coli Dataset 3. Drosophila Dataset 4. C. elegan Dataset**	**BERT**	_	**1. Acc=99.10, Sn=97.92, Sp=100, Precision=100, F1-score=98.94, MCC=98.19 2. Acc=99.74, Sn=99.62, Sp=99.82, Precision=99.75, F1-score=99.68, MCC=99.46 3. Acc=99.98, Sn=99.96, Sp=100, Precision=100, F1-score=99.98, MCC=99.96 4. Acc=99.44, Sn=99.83, Sp=98.78, Precision=99.30, F1-score=99.56, MCC=98.80**
		Kermani et al., 2022 (319)	Kermani et al. Datasets: 1. HPRD Dataset 2. C. elegans Dataset 3. E. coli Dataset 4. M. musculus Dataset	PSSM + Node2vec	LogR	1. Acc=0.752 2. Acc=0.732 3. Acc=0.706 4. Acc=0.798
		**Su et al., 2022 (241)**	**Su et al. Dataset**	**LINE**	**RF**	**Acc= 86.55%, Sn= 82.49%, Precision= 89.79%, AUROC= 0.9301, AUPRC= 0.9308**
		**Pan et al., 2022 (242)**	**Pan et al. Datasets: 1. A. thaliana Dataset 2. Zea mays Dataset 3. Oryza sativa Dataset**	**Word2Vec**	**MLP**	**1. Acc=89.47 ± 0.32, Sn=91.47 ± 0.27, Sp=87.48 ± 0.88, Precision=87.97 ± 0.72, MCC=79.02 ± 0.61, AUROC=0.9548 ± 0.0034 2. Acc=95.00 ± 0.38, Sn=96.30 ± 0.38, Sp=93.69 ± 0.70, Precision=93.85 ± 0.63, MCC=90.02 ± 0.75, AUROC=0.9867 ± 0.0025 3. Acc=85.63 ± 0.17, Sn=86.38 ± 0.13, Sp=84.89 ± 0.23, Precision=85.11 ± 0.21, MCC=71.28 ± 0.35, AUROC=0.9213 ± 0.0019**
		**Asim et al., 2022 (243)**	**1. Asim et al. Dataset: S.cerevisiae Dataset, 2. Martin et al. Dataset: H. pylori Dataset**	**FastText**	**LSTM + CNN**	**1. Accurcay=0.9573, Precision=0.9575, Recall=0.9394, MCC=0.9144 2. Accurcay=0.9263, Precision=0.9284, Recall=0.9609, MCC=0.8547**
		Ray et al., 2022 (65)	1. CCSB Dataset 2. HPRD Dataset SARS-CoV2-host Datasets: 3. Dataset 3–4. Dataset 4	Node2Vec	Louvain clustering	_
		Xu et al., 2022 (146)	Xu et al. Datasets: 1. Yeast Dataset 2. Human Dataset	ESM-1b + BERT	–	1. Acc=66.07 2. Acc=88.06
		**Madan et al., 2022 (147)**	**Tsukiyama et al. Dataset: 1. host-virus PPI Dataset Guo et al. Dataset: 2. Yeast PPI Dataset Sun et al. Dataset: 3. Human PPI Dataset**	**BERT**	_	**1. AUROC=98.50, AUPRC=94.50, F1-score=89.69, MCC=88.76 2. AUROC=99.61, AUPRC=99.58, F1-score=97.37, MCC=94.77 3. AUROC=99.74, AUPRC=99.66, F1-score=98.84, MCC=97.67**
		**Zhang et al., 2022 (148)**	**Chen et al. Datasets: 1. SHS27k 2. SHS148k 3. STRING**	**BERT + TransE**	GNN	**1. BFS = 72.26, DFS = 78.89 2. BFS = 75.23, DFS = 77.52 3. BFS = 76.71, DFS = 91.45**
		**Ieremie et al., 2022 (149)**	**Ieremie et al. Datasets: 1. S.cerevisiae Dataset, 2. H.sapiens Dataset**	**Transformer**	_	**1. AUROC=0.961 2. AUROC=0.974**
		**Su et al., 2021 (244)**	**1. PPI network Dataset, 2. GraphSAGE-PPI Dataset**	**DeepWalk+ Node2Vec**	**LogR**	**1. Acc=0.99997, Precision=1.0, Sn=0.99993, MCC=0.99993, AUROC=0.99996 2. Acc=0.9979, Precision=1.0, Sn=0.9958, MCC=0.9958, AUROC=0.9979**
		**Zhang et al., 2020 (245)**	**E.coli PPI Dataset (CC, BP, MF**)	**Node2Vec**	**LSTM**	**CC: Acc=0.81, AUROC=0.91 BP: Acc=0.83, AUROC=0.93 MF: Acc=0.81, AUROC=0.91**
		Zhong et al., 2020 (246)	1. HUMAN Dataset (BP, CC, MF) 2. MOUSE Dataset (BP, CC, MF) 3. YEAST Dataset (BP, CC, MF)	Node2Vec	SVM	1. BP: AUROC=0.8814, CC: AUROC=0.8396, MF: AUROC=0.8397 2. BP: AUROC=0.8728, CC: AUROC=0.8517, MF: AUROC=0.8608 3. BP: AUROC=0.8889, CC: AUROC=0.8358, MF: AUROC=0.8411
Binary Classification	Protein Complexes Identification	Zhou et al., 2023 (247)	1. Krogan14K Dataset 2. Collins et al. Dataset 3. HPRD Dataset	Word2Vec	GCN	1. Precision=0.537, Recall=0.437, F1-score=0.482, Acc=0.505 2. Precision=0.609, Recall=0.571, F1-score=0.590, Acc=0.619 3. Precision=0.5468, Recall= 0.6474, F1-score=0.5928
		**Wang et al., 2022 (248)**	**1. Gavin Dataset 2. Krogan core Dataset 3. DIP Dataset 4. MIPS Dataset**	**Node2Vec**	**Ensemble (LR +BR+ DTR+SVM)**	**1. F1-score=0.6674, CR=0.4792, Acc=0.3391, MMR=0.2516, Jaccard=0.4330 2. F1-score=0.6287, CR=0.4239, Acc=0.2984, MMR=0.2687, Jaccard=0.4302 3. F1-score=0.6200, CR=0.4922, Acc=0.2768, MMR=0.2273, Jaccard=0.3454 4. F1-score=0.4811, CR=0.2914, Acc=0.2237, MMR=0.1678, Jaccard=0.2599 (using Standard Protein Complexes 2) 1. F1-score=0.4546, CR=0.3838, Acc=0.3259, MMR=0.1745, Jaccard=0.3619 2. F1-score=0.5336, CR=0.3768, Acc=0.2827, MMR=0.1750, Jaccard=0.3785 3. F1-score=0.5126, CR=0.3998, Acc=0.2607, MMR=0.1386, Jaccard=0.3020 4. F1-score=0.4026, CR=0.2599, Acc=0.1937, MMR=0.1011, Jaccard=0.2249**
		**Meng et al., 2021 (249)**	**1. DIP Dataset, 2. BIOGRID Dataset**	**DeepWalk**	**Core Attachments based Clustering Method**	**1. CYC2008: Precision=0.469, Recall=0.648, F1-score=0.544, Acc=0.585, F1-score+Acc=1.129; NewMIPS: Precision=0.510, Recall=0.643, F1-score=0.569, Acc=0.338, F1-score+Acc=0.907; 2. CYC2008: Precision=0.405, Recall=0.797, F1-score=0.537, Acc=0.671, F1-score+Acc=1.207 NewMIPS: Precision=0.411, Recall=0.684, F1-score=0.514, Acc=0.384, F1-score+Acc=0.897**
		Zhu et al., 2019 (250)	Zhu et al. Datasets: 1. Krogan Dataset 2. DIP Dataset 3. BIOGRID Dataset	DeepWalk	GCN	1. Frac=0.61, Acc=0.68, MMR=0.5 2. Frac=0.81, Acc=0.68, MMR=0.75 3. Frac=0.35, Acc=0.69, MMR=0.28
		Yao et al., 2019 (251)	1. Collins et al. Dataset 2. Gavin Dataset 3. Krogan Dataset 4. Kiemer et al. Dataset: Wiphi Dataset	Node2Vec	k-means	1. F1-score=0.6060 2. F1-score=0.5293 3. F1-score=0.5808 4. F1-score=0.5236 (MIPS) 1. F1-score=0.5000 2. F1-score=0.4663 3. F1-score=0.4775 4. F1-score=0.4334
Interaction	Residue-Residue Interaction Prediction	**Hong et al., 2021 (252)**	**Hong et al. Dataset**	**Node2Vec + physico-chemical properties based approaches based representation learning**	**LogR**	**Acc=54.5%**
Interaction	Contact Prediction	**Chen et al., 2024 (150)**	**trRosetta Dataset**	**ESM-2**	–	**Acc=93.32**
		**Si et al., 2024 (151)**	**1. HomoPDB Dataset 2. HetroPDB Dataset 3. DHTest Dataset 4. DB5.5 Dataset**	**ESM-1**	GVPConv	**1. Precision=65.25 2. Precision=45.0 3. Precision=75.4 4. Precision=40.0**
		Si et al., 2023 (152)	1. HomoPDB Dataset 2. HetroPDB Dataset 3. DHTest Dataset 4. DB5.5 Dataset	ESM-1	CNN	1. Precision=57.6 2. Precision=38.5 3. Precision=56 4. Precision=27.5
		Elnaggar et al., 2023 (27)	ProteinNet Dataset	T5	–	Precision=73.2 ± 11
		**Singh et al., 2022 (153)**	**1. SPOT-2018 2. CASP14-FM**	**ESM-1**	**CNN**	**1. Medium range: Precision=42.43, Long range: Precision=39.60 2. Medium range: Precision=41.44, Long range: Precision=25.23**
		Zhang et al., 2022 (148)	ProteinNet Dataset	BERT + TransE	GNN	6<seq<12 L/5: Average Precision=0.57 12<seq<24 L/5: Average Precision=0.50 24<seq L/5: Average Precision=0.39
		**Xiao et al., 2021 (154)**	**ProteinNet Dataset**	**BERT**	–	**Precision=0.75**
		Rao et al., 2019 (155)	ProteinNet Dataset	Transformer	ResNet	Precision=0.36
Interaction	Nucleic Acid-Binding Proteins Identification	Liu et al. 2024 (156)	1. Patiyal et al. Dataset (Dataset 1) 2. Xia et al. Dataset (Dataset 2)	BERT	CNN	1. Sp=0.835, Recall=0.747, Precision=0.306, F1-score=0.434, MCC=0.401, AUROC=0.871 2. Sp=0.955, Recall=0.464, Precision=0.396, F1-score=0.427, MCC=0.389, AUROC=0.881
		**Roche et al., 2024 (157)**	**1. Protein-DNA Datasets: Test_129, Test_181 2. Protein-RNA Dataset: Test_117**	**ESM-2 + AlphaFold2 + PSSM**	_	**1. Protein-DNA: Test_129: AUROC=0.940, AUPRC=0.569 Test_181: AUROC=0.918, AUPRC=0.384 2. Protein-RNA: Test_117: AUROC=0.886, AUPRC=0.320**
		Luo et al. 2023 (158)	690 ChIP-Seq Dataset	BERT	–	AUROC=0.947 ± 0.041, Acc=0.880 ± 0.062, Precision=0.882 ± 0.061, Recall=0.880 ± 0.062, F1-score=0.880 ± 0.062, MCC=0.762 ± 0.122
		Murad et al. 2023 (159)	Liu and Tian 2023 Datasets	BERT	CNN	Dataset 1: Sp=0.529, Precision=0.106, Recall=0.574, F1-score=0.179, AUROC=0.551, MCC=0.025 Dataset 2: Sp=0.724, Precision=0.119, Recall=0.536, F1-score=0.194, AUROC= 0.630, MCC=0.067
		**Zeng et al., 2023 (160)**	**1. YK17 Dataset, 2. DRNA-1314 Dataset**	**ESM-2**	**BiLSTM**	**1. DNA-Binding residue: MCC=0.427, Average Precision=0.405, AUROC=0.121 RNA-Binding residue: MCC=0.218, Average Precision=0.148, AUROC=0.462 2. DNA-Binding residue: MCC=0.391, Average Precision=0.350, AUROC=0.195 RNA-Binding residue: MCC=0.276, Average Precision=0.232, AUROC=0.462**
		**Yang et al., 2020 (253)**	**1. RRM162 Dataset, 2. Homeo215 Dataset**	**FastText**	**Affinity Regression**	**1. SRCC=0.864 2. SRCC=0.772**
Interaction	Protein–RNA Interaction Prediction	**Wang et al., 2024 (320)**	**RPI369 Dataset, RPI488 Dataset, RPI1446 Dataset, RPI1807 Dataset, RPI2241 Dataset**	**k-mer + DCC + KGap Descriptors + PseTNC + Conjoint Triad + GDPC + QSOrder Descriptors + DDE + ACC (Amino Acid Occurrence based representation learning approach)**	**GCN**	**1. Acc=97.27 2. Acc=97.32 3. Acc=96.54 4. Acc=95.76 5. Acc=94.98**
		Li et al., 2024 (254)	Li et al. Dataset	Word2Vec	GNN	DB1: AUROC=95.51 ± 0.36, AUPRC=94.24 ± 0.61, Acc=89.95 ± 0.67, Precision=87.44 ± 1.00, Recall=93.31 ± 0.64, F1-score=90.28 ± 0.61 DB2: AUROC=97.31 ± 0.31, AUPRC=96.80 ± 0.47, Acc=92.30 ± 0.47, Precision=92.12 ± 0.44, Recall=92.51 ± 0.94, F1-score=92.31 ± 0.49 DB3: AUROC=95.47 ± 0.32, AUPRC=93.87 ± 0.74, Acc=91.02 ± 0.24, Precision=87.67 ± 0.66, Recall=95.49 ± 0.83, F1-score=91.41 ± 0.23 DB4: AUROC=96.46 ± 0.34, AUPRC=94.91 ± 0.76, Acc=92.83 ± 0.28, Precision=90.10 ± 0.59, Recall=96.23 ± 0.38, F1-score=93.06 ± 0.25
		**Han et al., 2023 (255)**	**NPInter2.0 Dataset, RPI7317 Dataset, RPI38317 Dataset**	**Node2Vec**	**GNN**	**NPInter2.0: Sn=98.2 ± 0.2, Sp=95.0 ± 0.2, Precision=95.1 ± 0.2, Acc=96.6 ± 0.1, MCC=0.932 ± 0.002; RPI7317: Sn=94.5 ± 0.4, Sp=91.3 ± 0.8, Precision=92.0 ± 0.3, Acc=93.1 ± 0.1, MCC=0.863 ± 0.002**
		Wei et al., 2023 (256)	Wei et al. Dataset	HIN2Vec	SVM	AUROC=0.97, Acc=0.95, Precision=0.932, Recall=0.981, Sp= 0.928, MCC=0.9102, F1-score= 0.956
		Zhao et al., 2023 (257)	Zhao et al. Dataset 1, Zhao et al. Dataset 2	VGAEs + Word2Vec	GAE	1. AUROC=0.974, AUPRC=0.7688, Acc=0.9851, F1-score=0.6397, Precision=0.4238 2. AUROC=0.9734, AUPRC=0.9421, Acc=0.9305, F1-score=0.8534, Precision=0.7871
		Shen et al., 2021 (258)	NPInter2.0 Dataset, RPI7317 Dataset, RPI2241 Dataset, RPI369 Dataset	Node2Vec	GNN	1. Acc=93.3, Sn=95.6, Sp=91.1, Precision=91.5, MCC=0.868 2. Acc=91.5, Sn=92.7, Sp=90.7, Precision=90.7, MCC=0.830 3. Acc=62.6, Sn=49.8, Sp=74.8, Precision=67.2, MCC=0.270 4. Acc=60.2, Sn=61.5, Sp=58.9, Precision=60.0, MCC=0.212
		Yi et al., 2020 (259)	RPI369 Dataset, RPI1807 Dataset, RPI488 Dataset	Word2Vec	RF	RPI369 Dataset: Acc=73.06, Sn=75.32, Sp=71.14, Precision=72.64, MCC=46.67; RPI488 Dataset: Acc=89.92, Sn=82.75, Sp=96.72, Precision=96.32, MCC=80.59; RPI1807 Dataset: Acc=97.10, Sn=97.89, Sp=96.14, Precision=96.91, MCC=94.13;
Interaction	Virus Host Protein Interaction Prediction	**Yang et al., 2024 (48)**	**Yang et al. Dataset**	**Word2Vec**	**LightGBM**	**AUROC=0.919, AUPRC=0.408, Precision=0.395, Recall=0.688, Acc=0.881, F1-score=0.502**
		Xie et al., 2023 (49)	1. Human-HIV Dataset 2. Human-Herpes Dataset 3. Human-Papilloma Dataset 4. Human-Influenza Dataset 5. Human-Hepatitis Dataset 6. Human-Dengue Dataset 7. Human-Zika Dataset 8. Human-SARS-CoV-2 Dataset	Word2Vec	BiLSTM	1. Sn=88.65, Sp=87.72, Acc=88.18, Precision=90.29, F1-score=87.74, AUROC=97.95, AUPRC=97.90 2. Sn=74.44, Sp=77.07, Acc=75.76, Precision=77.79, F1-score=75.68, AUROC=86.38, AUPRC=86.94 3. Sn=53.42, Sp=83.56, Acc=68.48, Precision=70.20, F1-score=72.66, AUROC=84.08, AUPRC=82.50 4. Sn=69.75, Sp=82.96, Acc=76.35, Precision=77.28, F1-score=78.31, AUROC=88.84, AUPRC=89.12 5. Sn=67.85, Sp=69.23, Acc=68.54, Precision=74.63, F1-score=66.27, AUROC=83.16, AUPRC=81.58 6. Sn=58.92, Sp=75.57, Acc=67.22, Precision=66.55, F1-score=69.28, AUROC=77.51, AUPRC=76.16 7. Sn=39.09, Sp=84.37, Acc=61.65, Precision=60.47, F1-score=68.52, AUROC=74.94, AUPRC=75.22 8. Sn=30.61, Sp=80.88, Acc=55.64, Precision=56.24, F1-score=62.68, AUROC=68.01, AUPRC=67.16
		Chakraborty et al., 2023 (50)	Chakraborty et al. Datasets: 1. Set-1 2. Set-2 3. Set3-3	AAC + PseAAC + CT + DC + Normalized-AC (Amino Acid Occurrence based representation learning approach)	AdaBoost + SVM + RF	1. Acc=63.636, RMSE=0.4867, Precision=0.639, Sn=0.636, F1-score=0.638, MCC=0.260, MAE=0.4041 2. Acc=68.391, RMSE=0.5622, Precision=0.685, Sn=0.684, F1-score=0.683, MCC=0.368, MAE=0.316 3. Acc=67.275, RMSE=0.466, Precision=0.673, Sn=0.673, F1-score=0.673, MCC=0.345
		**Xia et al., 2022 (51)**	**Xia et al. Dataset**	**Node2Vec**	**CCA**	**Score=0.1998, Seed=726, Precision=0.9399, Recall=0.6078, F1-score=0.7382**
		**Asim et al., 2022 (52)**	**1. Barman et al. Dataset 2. Fatma et al. Dataset 3. Yang et al. Dataset 4. TR-TS1 Dataset 5. TR-TS2 Dataset 6.TR-TS1 Dataset 7. TR-TS2 Dataset**	**APAAC + QS order (Amino Acid Occurrence based representation learning approach)**	**SVM**	**1. Acc=82.90, Sn=90.87, Sp=82.90, Precision=84.08, F1-score=82.74, MCC=66.96, AUROC=88.17 2. Acc=94.59, Sn=97.23, Sp=94.59, Precision=94.73, F1-score=94.58, MCC=89.32, AUROC=98.16 3. Acc=91.18, Sn=95.58, Sp=51.74, Precision=86.01, F1-score=87.27, MCC=10.08,AUPRC=47.07, AUROC=82.95 4. Acc=90.26, Sn=95.06, Sp=90.26, Precision=91.44, F1-score=90.19, MCC=81.69, AUUROC=96.70 5. Acc=94.30, Sn=97.07, Sp=94.30, Precision=94.39, F1-score=94.29, MCC=88.69, AUROC=97.77 6. Acc=90.53, Sn=95.06, Sp=90.53, Precision=90.78, F1-score=90.51, MCC=81.31, AUROC=95.98 7. Acc=93.62, Sn=96.71, Sp=93.62, Precision=93.64, F1-score= 93.62, MCC=87.27, AUROC=98.14**
		**Lanchantin et al., 2021 (47)**	**1. SARS-CoV-2 Interaction Dataset 2. H1N1 Dataset 3. Ebola Dataset**	**Transformer**	–	**1. AUROC=0.753, AUPRC=0.076, F1-score=0.114, Precision=0.151 2. AUROC=0.945, AUPRC=0.948, F1-score=86.5 3. AUROC=0.968, AUPRC=0.974, F1-score=89.6**
Interaction	Compound-Protein Interaction Prediction	**Du et al., 2024 (321)**	**BindingDB 3CLpro Dataset**	**RDKit**	**MPNN + BiLSTM**	**AUROC=0.958, Acc=0.938, Sn=0.957, Sp=0.912, Recall=0.957, MCC=0.870**
		**Wang et al., 2024 (453)**	**1. Wang et al. Datasets: BindingDB Dataset, DrugBank Dataset, GPCR Dataset, 2. Davis et al. Dataset**	**GCN + CNN**	**GRU + MLP**	**BindingDB Dataset: AUROC=0.9778 ± 0.0008, Acc=0.9291 ± 0.0028 DrugBank Dataset: AUROC=0.8507 ± 0.0047 GPCR Dataset: AUROC=0.8687 ± 0.0031, AUPRC=0.9660 ± 0.0007 Davis Dataset: AUROC=0.8688 ± 0.0100, AUPRC=0.7807 ± 0.0148**
		**Ma et al., 2024 (161)**	**Liu et al. Datasets: 1. Balanced Human Dataset, 2. Balanced C. elegans Dataset**	**AlphaFold2 + RDkit**	**Transformer + MLP**	**1: AUROC=0.990 ± 0.002, Precision=0.952 ± 0.004, Recall=0.979 ± 0.004 2: AUROC=0.989 ± 0.003, Precision=0.935 ± 0.005, Recall=0.959 ± 0.004**
		Koyama et al., 2024 (61)	Koyama et al. Dataset: BindingDB Dataset	ESM-2 + MolFormer	–	AUROC=0.828(0.018), AUPRC=0.827(0.049), Acc=0.755(0.019), F1-score=0.756(0.036)
		**Palhamkhani et al., 2023 (260)**	**Palhamkhani et al. Dataset: BindingDB Dataset**	**Node2Vec + OHE**	**CNN**	**CI=0.843 ± 0.01, R=0.862, MSE=0.806, R2=0.650 ± 0.01, AUPRC=0.519 ± 0.01**
		**Chen et al., 2023 (261)**	**Chen et al. Dataset**	**RDkit + GraRep**	**XGBoost**	**Acc=90.09 ± 0.41, Sn=86.16 ± 0.90, Sp=94.03 ± 0.64, Precision=93.52 ± 0.63, MCC=0.8044 ± 0.0080**
		Wang et al., 2022 (262)	Wang et al. Datasets: 1. Human Dataset 2. C.elegans Dataset	Node2Vec, Word2Vec + CNN, RDkit + ResNet	MLP	1: AUROC=0.978 ± 0.002, Precision=0.960 ± 0.004, Recall=0.958 ± 0.005; 2: AUROC=0.990 ± 0.002, Precision=0.955 ± 0.005, Recall=0.954 ± 0.004
		**Watanabe et al., 2021 (263)**	**Watanabe et al. Dataset**	**CNN, ECFP, Node2Vec**	**MLP**	**AUROC=0.972 ± 0.004, AUPRC=0.954 ± 0.005, F1-score=0.900 ± 0.006, Acc=0.933 ± 0.004**
		**Chen et al, 2020 (162)**	**Tsubaki et al. Datasets: 1. Human Dataset 2. C.elegans Dataset 3. Gao et al. Dataset: BindingDB Dataset**	**Word2Vec, RDkit, GCN**	**Transformer Decoder + MLP**	**1. AUROC=0.973+0.002, Precison=0.916+0.006, Recall=0.925+0.006 2. AUROC=0.988+0.002, Precision=0.952+0.006, Recall=0.953+0.005 3. AUROC=0.951, Precision=0.949**
Regression	Compound-Protein Binding Affinity Prediction	Koyama et al., 2024 (61)	1. Koyama et al. Dataset: PDBbind Dataset 2. Zhao et al. Dataset: Metz Dataset	ESM-2, MolFormer	–	1. RMSE=1.530(0.069), PCC=0.598(0.039), SRCC=0.598(0.042), MAE=1.201(0.045) 2. RMSE=0.851(0.043), PCC=0.549(0.035), SRCC=0.491(0.044), MAE=0.689(0.040)
		**Ma et al., 2024 (161)**	**1. Tang et al. Dataset: KIBA Dataset 2. Davis et al. Dataset**	**AlphaFold2, RDkit**	**Transformer, MLP**	**1. MSE=0.212, PCC=0.802, CI=0.908 2. MSE=0.141, PCC=0.895, CI=0.913**
		Xu et al., 2023 (62)	1. Davis et al. Dataset 2. Xu et al. Dataset: KIBA Dataset	Integer Encoding, RoBERTa	BiGRU	1. CI=0.895, MSE=0.213 2. CI=0.902, MSE=0.135
		**Lin et al., 2022 (63)**	**Lin et al. Dataset: KIBA Dataset**	**Transformer, GNN**	–	KIBA Dataset: MSE=0.4582, R***{}2=0.3906
		Wang et al., 2021 (163)	Wang et al. Datasets: 1. KIBA Dataset 2. BindingDB Dataset	Transformer, BERT	CNN	1. CI=0.888, MSE=0.151, PCC=0.885, R2=0.780 2. CI=0.815, MSE=0.798, PCC=0.821, R2=0.640
		Zhao et al., 2020 (64)	1. Davis et al. Dataset 2. Tang et al. Dataset: KIBA Dataset	OHE, BERT	CNN	1. CI=0.891, MSE=0.229, R2=0.654, PCC=0.850, AUPRC=0.717 2. CI=0.876, MSE=0.179, R2=0.706, PCC=0.868, AUPRC=0.782
Interaction	Phage-Host Interaction Prediction	**Gonzales et al., 2023 (164)**	**Gonzales et al. Dataset**	**T5**	**RF**	**k=100%: Weighted Precision=77.23% k=60%: Weighted Recall=59.15% k=60%: Weighted Sp=94.44%**
		Pan et al., 2023 (264)	ESKAPE Dataset	SDNE + Word2Vec	MLP	Acc=86.65 ± 1.55, Sn=88.40 ± 1.81, Sp=84.91 ± 1.96, Precision=85.43 ± 1.74, F1-score=86.88 ± 1.53, AUC=0.9208 ± 0.0119
Binary Classification	Missing Link Prediction	Kermani et al., 2023 (265)	Kermani et al. Datasets: 1. H. sapiens Dataset 2. M. musculus Dataset 3. S. cerevisiae Dataset	ANE + PSSM + RandomWalk	LogR	1: F1-score=0.87, MCC=0.78 2: F1-score=0.89, MCC=0.73 3: F1-score=0.87, MCC=0.75
		**Balogh et al., 2022 (266)**	**Balogh et al. Datasets: 1. Homo sapiens Dataset 2. S. cerevisiae Dataset 3. Mus musculus Dataset 4. Rattus norvegicus Dataset 5. Sus scrofa Dataset**	**Node2Vec**	**cGAN**	**1: AUROC=0.913, AUPRC=0.169, NDCG=0.761 2: AUROC=0.931, AUPRC=0.202, NDCG=0.781 3: AUROC=0.909, AUPRC=0.137, NDCG=0.742 4: AUROC=0.925, AUPRC=0.252, NDCG=0.809 5: AUROC=0.898, AUPRC=0.120, NDCG=0.721**
		Patel et al., 2022 (267)	Orphanet Dataset	Node2Vec	LightGBM	weighted Recall=0.84, weighted Precision=0.93, weighted F1-score=0.87, AUROC=0.90, AUPRC=0.78
		Nasiri et al., 2021 (268)	Nasiri et al. Datasets: 1. E.coli Dataset 2. C.elegan Dataset 3. Drosophila Dataset	DeepWalk	LogR	1. AUROC=0.7937, F1-score=0.6996, RMSE=0.2060, PCC=0.4727 2. AUROC=0.6586, F1-score=0.5278, RMSE=0.3135, PCC=0.2018 3. AUROC=0.6024, F1-score=0.5240, RMSE=0.3095, PCC=0.1273
		Feng et al., 2020 (269)	Feng et al. Dataset	GAT	CNN	Acc=0.888, AUROC=0.954, Recall=0.876, Precision=0.898, Sp=0.900
		Mallick et al., 2019 (270)	1. PPI Dataset, 2. Homo Sapiens PPI Network Dataset	Topo2Vec	RF	1. AUROC=0.8162 2. AUROC=0.8978

Among all representation approaches, Node2Vec emerges as most commonly used representation learning approach followed by BERT and Transformer. Specifically, Node2Vec is used with CNN, Louvain clustering, LSTM, SVM and Ensemble (GNN + MLP) classifiers for protein–protein interaction prediction (38, 45, 65, 245, 246) and potential of Node2Vec is also explored with GNN classifier Protein-RNA Interaction Prediction (255, 258). Moreover, Node2Vec is employed with ensemble (LR+BR+DTR+SVM) classifier and k-means clustering algorithm for protein complexes interaction prediction (248, 251). Similarly, Node2Vec is also employed with CCA, cGAN, and LightGBM classifiers for virus-host interaction prediction (51) and missing link prediction (266, 267), respectively. Despite being most commonly used representation learning, not a single Node2Vec based predictive pipelines manages to achieve state-of-the-art performance. In addition, combined potential of Node2Vec with four different representation learning approaches namely Word2Vec, PSSM, DeepWalk and physico-chemical properties based approaches is explored with LogR classifier for protein–protein interaction prediction (45, 244, 319) and residue-residue interaction prediction (252). Similarly, combined representation from Node2Vec and OHE is utilized with CNN classifier (260), and combined potential of Node2Vec with (Word2Vec+CNN, RDkit+ResNet) (262) and CNN+ECFP approaches (263) is explored with MLP classifier for compound-protein interaction prediction. Among these combined approaches, Node2vec and physico-chemical properties based representation learning approaches with LogR classifier has achieved state-of-the-art performance for residue-residue interaction prediction (252). Furthermore, BERT is the second most commonly used representation learning, which is employed with four distinct classifiers for four different tasks. Particularly, BERT is used with CNN and a self-classifier for nucleic acid-binding proteins identification (156, 158, 159). In addition, potential of BERT and a self-classifier is also explored for two other tasks namely protein–protein interaction prediction (145, 147) and contact prediction (154). Moreover, combined potential of BERT is explored with several other approaches as: BERT + Transformer + OHE along with CNN classifier for compound-protein binding affinity prediction (64, 163), BERT + TransE is used with GNN for contact prediction (185) and protein–protein interaction prediction (148), BERT + ESM-1 with a self-classifier (146), and BERT with Transformer + GAT-CNN classifier is used for protein–protein interaction prediction (43).

Beyond Node2Vec and BERT representation learning approaches, transformer is employed with a self-classifier and ResNet for three tasks namely protein–protein interaction prediction (149), contact prediction (155), and virus-host interaction prediction (47). Moreover, combined representation from transformer and GNN is used with a self-classifier for compound-protein interaction prediction (162). Among all transformer based predictive pipelines, transformer with a self-classifier has achieved state-of-the-art performance for contact prediction (150). Apart from this, Word2Vec is used with MLP classifier for protein–protein interaction prediction (242). Furthermore, Word2Vec is also used with LightGBM and BiLSTM classifiers for virus–host interaction prediction (48, 49). Word2Vec is also employed with GNN and RF classifiers for protein complexes identification (247) and protein–protein interaction prediction (254, 259), respectively. Among all Word2Vec based predictive pipelines, Word2Vec with BiLSTM manages to achieve top performing values for virus host interaction prediction (49). Apart from standalone potential of Word2Vec representation learning, combined potential of Word2Vec and VGAEs with a GAE classifier is explored for protein–RNA interaction prediction (257). Similarly, Word2Vec and SNDE combined used as representation learning approach with MLP classifier for phage-host interaction prediction (264), whereas combined representation learning from Word2Vec, RDkit, and GCN is used with transformer-decoder and MLP classifier for compound-protein interaction prediction (162). Among all Word2Vec based combined representation learning approaches, Word2Vec and SDNE representation with MLP classifier has achieved state-of-the-art performance for phage-host interaction prediction (264).

ESM-1 with a self-classifier is used for protein–protein interaction prediction (44). In addition, potential of ESM-1 is also explored with CNN and and GVPConv classifiers for contact prediction (151–153). In contrast, ESM-2 with GAT and BiLSTM classifier is used for protein–protein interaction prediction (36) and nucleic acid binding protein identification (160), whereas potential of ESM-2, AlphaFold, PSSM and MolFormer is explored with a self-classifier and BiLSTM for nucleic acid-binding proteins identification (157, 160), Compound-Protein Interaction (61) and Compound-Protein Binding Affinity Prediction (61). Similarly, potential of T5 is explored with RF, hybrid (XGBoost+CAPT5) and self-classifier for phage-host interaction prediction (164), protein–protein interaction prediction (35) and contact prediction (27) respectively. Among all ESM-1, ESM-2 and T5 language model based predictive pipelines, T5 with a hybrid (XGBoost+CAPT5) classifier has achieved state-of-the-art performance for protein–protein interaction prediction (35). In addition, RoBERTa is used with LogR classifier for protein–protein interaction prediction (42) and combined potential of RoBERTa with Integer Encoding is explored with BiGRU classifier for compound-protein binding affinity prediction (62), whereas AlphaFold2 and RDkit is used with a self-classifier for compound-protein interaction prediction (161) and compound-protein binding affinity prediction (161). Among both RoBERTa and AlphaFold2 language models based predictive pipelines, AlphaFold2 with a self-classifier has achieved state-of-the-art performance for compound-protein binding affinity prediction (161).

Furthermore, FastText is explored with Affinity Regression and hybrid (LSTM+CNN) classifiers for nucleic acid-binding proteins identification (253) and protein–protein interaction prediction (243). DeepWalk is used with clustering algorithm, GCN, and LogR classifier for protein complexes identification (249, 250) and Missing Link Prediction (268). In addition, ELMo is used with LogR classifier and LINE method is employed with RF classifier and combined potential of ELMo and vision transformer is explored with a self-classifier for protein–protein interaction prediction (46, 240, 241). Furthermore, HIN2Vec is used with SVM for protein–RNA interaction prediction (256), GraRep and RDkit representation is explored with XGBoost classifier for compound-protein interaction prediction (261), potential of Topo2Vec with SVM classifier is explored for missing link prediction (270). Beyond word embedding and language model based representation learning approaches, amino acid occurrence-based representation learning is also employed with GCN, SVM and hybrid (AdaBoost+SVM+RF) classifiers for protein–RNA interaction prediction (320) and virus host protein interaction prediction (50, 52). Similarly, score matrix and physico-chemical properties based approaches are utilized with CNN classifier for protein–protein interaction prediction (41). Among both, amino acid occurrence based representation learning approaches with GCN have achieved state-of-the-art performance for protein–RNA interaction prediction (320). In addition, combined potential of ANE + PSSM + RandomWalk representation learning is explored with a LogR classifier and TFIDF with SVM classifier (39), GAT with CNN classifier for Missing Link Prediction (269) and RDkit with hybrid MPNN and BiLSTM are used for Compound-Protein Interaction (321) for protein–protein interaction prediction. Moreover, ASNE+PSSM+Random Walk with LogR classifier is used for missing link prediction (265).

Detailed analysis of all these predictive pipelines indicates that there is a significant room of improvement in residue-residue interaction prediction, protein complexes identification and phage-host interaction prediction. Considering the performance trend for this goal, potential of LLMs such as T5, BERT, transformer with standalone or hybrid deep learning predictors such as CNN, BiLSTM, CNN+LSTM, CNN+BiGRU, and XGBoost+CAPT5 classifiers can enhance the performance of under-performing tasks.

Furthermore, Table 10 provides a high level overview of performance of 30 predictive pipelines that are developed using three different approaches namely (1) language models, (2) word embeddings, (3) domain-specific approaches, under the hood of peptide analysis goal. A detailed review of existing studies have identified 21 unique representation learning approaches namely Word2Vec, OHE, T5, ESM-1, Transformer, AlphaFold, BERT, IgFold, CTF, random embedding, TPC, motif features, LPE, matrix reduction based methods, amino acid occurrence based approaches, structural amino acid composition based approaches, amino acid composition based approaches, ProtDCal software based approaches, physico-chemical based representation approaches, k-Gap based approaches and amino acid structural and occurrence frequency based approaches. Moreover, these predictive pipelines have utilized 24 unique classifiers including LogR, SVM, SnTCN, GRU, CNN, LSTM, LightGBM, BiLSTM, RF, k-means clustering algorithm, scoring card method, BR, CC, MLkNN, GRNN, kNN, PNN, LR, ETC, XGB, CatBoost and self-classifiers of BERT, AlphaFold, and IgFold.

**Table 10.** Peptide analysis related eight distinct protein sequence analysis tasks predictive pipelines performance

Task Type	Task Name	Author, Year [ref]	Dataset	Representation learning	Classifier	Performance Evaluation
Binary Classification	Anti-cancer peptides Identification	**Deng et al., 2023 (324)**	**ACP_mixed_80: 303 ACPs, 303 non-ACPs**	**AAC+ AAIN+ BPF+ CTDD+ DDE (amino acid occurrence-based representation approach)**	**RF+ BR+ CC+ MLkNN**	**Acc=0.89, Sn=0.79, Sp=0.8, MCC=0.78, P=0.77, AUROC=0.57**
		**Garai et al., 2023 (326)**	**ENNAACT_main: 659 ACPs, 5257 non-ACPs**	**OHE+ secondary structure features (structural amino acid composition based representation approach)**	**CNN+ LSTM+ MAM**	**Acc=0.84, Sn=0.77, Sp=0.88, MCC=0.66, P=0.78, AUROC=0.92**
		**Yao et al., 2023 (327)**	**a) Set 1: 793 ACPs, 799 non-ACPs b) Set 2: 902 ACPs, 847 non-ACPs**	**FEGS+ BLOSUM62+ BPF (matrix reduction based representation approach)**	**RF**	**Dataset: Acc Sn Sp MCC Set 1: 77.1 76.8 78.3 77.5 Set 2: 94.1 97.7 90.9 94.2**
		**Han et al., 2022 (322)**	**ACP_Alternate: 970 ACPs, 970 non-ACPs**	**BPF+ QuanPAA+ QualPAA (Occurrence based representation approach)**	**BiLSTM**	**Acc=93.6, Sn=92.3, Sp=94.9, MCC=87.1**
		**Charoenkwan et al., 2021 (53)**	**ACP_Main: 861 ACPs, 861 non-ACPs**	**AAC+ DPC+ CTR (amino acid composition based representation approach)**	**Scoring Card method**	**Acc=82.5 Sn=72.6 Sp=90.3 MCC=0.64**
		**Wang et al., 2021 (325)**	**ACP_539: 189 ACPs, 350 non-ACPs**	**2BPF (Occurrence based representation approach)**	**LightGBM**	**Acc=0.97, Sn=0.8, Sp=0.97, MCC=0.97, AUROC= 0.98**
		**Akbar et al., 2017 (323)**	**ACP_344: 138 ACPs, 206 non-ACPs**	**PAAC+ PGDC+ RAAAC (amino acid composition based representation approach)**	**GRNN+ KNN+ PNN+ RF+ SVM**	**Acc=0.96, Sn=0.95, Sp=0.97, MCC=0.91**
Binary Classification	Anti-bacterial Peptides Identification	**Sharma et al., 2021 (54)**	**Sharma et al. Dataset: 1635 ABPs, 1485 non-ABPs**	**Word2Vec**	**BiLSTM**	**Dataset (CV or IND): Acc Sn Sp MCC P Sharma et al. Dataset (CV): 97.19 97.31 97.47 94.38 97.68 Sharma et al. Dataset (IND): 95.80 94.94 95.49 91.38 93.67**
		**Youmans et al., 2019 (337)**	**a) Original Dataset: 2609 ABPs, 3170 non-ABPs b) Reduced Dataset: 565 ABPs, 1910 non-ABPS**	**ProtDCal software (software based representation approach)**	**LSTM**	**Dataset: Acc MCC Original Dataset: 94.98 89.90 Reduced Dataset: 93.04 82.70**
Binary Classification	Antimicrobial peptides Identification	**Olcay et al., 2024 (331)**	**199 synergistic effect, 208 non-synergistic effect (Train, Valid)**	**OHE**	**LightGBM**	**Train: Acc=99.75, AUC=1 Valid: Acc=75.75, AUC=0.82, P=77.4, R=75.65, F1=75.30**
		**Bournez et al., 2023 (55)**	**a) Gram Positive Dataset: 2849 Non-AMP, 2942 AMP b) Gram Negative Dataset: 3163 Non-AMP, 2924 AMP c) Fungi Dataset: 1475 Non-AMP, 1069 AMP**	**AAC+ CTD+ DPC+ PAAC+ GPC (amino acid composition based representation approach)**	**RF**	**Dataset: Acc Sn Sp MCC AUROC Gram Positive Dataset: 0.79 0.79 0.79 0.58 0.86 Gram Negative Dataset: 0.8 0.78 0.82 0.61 0.87 Fungi Dataset: 0.77 0.63 0.9 0.55 0.86**
		**Xu et al., 2023 (328)**	**Dataset: 49 115 AMPs, 195 525 non-AMPs**	**OHE+ BLOSUM62+ AAI+ PAAC (matrix reduction based representation approach)**	**CNN**	**Dataset: Acc Sn Sp MCC AUROC P F1 Dataset 1: 0.9994, 1, 0.9988, 0.9979, 0.9982, 0.9969, 0.9985**
		**Teimouri et al., 2023 (332)**	**a) E.coli: 183 AMPs, 214 non-AMPs b) A. baumannii: 87 AMPs, 35 non-AMPs**	**PCP (physicochemical based representation approach)**	**LR+ SVM**	**E.coli: Acc=98, MCC=95, R=98 A. baumannii: Acc=100, MCC=100, R=100**
		**Wang et al., 2023 (333)**	**Wang et al. Dataset: 379 AMPs, 4115 non-AMPs**	**AAC+ GDPC+ QSOrder+ PAAC, GTDC, CTD (amino acid composition based representation approach)**	**k-means Clustering**	**Acc=0.8948 ± 0.0066, MCC=0.5789 ± 0.0115, F1=0.5858 ± 0.0108**
		**Jaiswal et al., 2023 (334)**	**Dataset 1 (Train): 1362 AMPs, 1217 non-AMPs Dataset 1 (IND): 453 AMPs, 405 non-AMPs Dataset 2 (Train): 1362 AMPs, 1362 non-AMPs Dataset 2 (IND): 453 AMPs, 453 non-AMPs Dataset 3 (Train): 1362 AMPs, 1362 non-AMPs Dataset 3 (IND): 453 AMPs, 453 non-AMPs**	**AAC+ DPC+ PCP (amino acid composition based representation approach)**	**SVM+ RF**	**Dataset: Acc Sn Sp MCC AUROC Dataset 1 (Train): 90.38 91.34 89.32 0.81 0.93 Dataset 1 (IND): 87.88 84.77 91.36 0.76 0.93 Dataset 2 (Train): 92.8 91.2 94.4 0.85 0.97 Dataset 2 (IND): 91.9 90.3 93.6 0.83 0.97 Dataset 3 (Train): 89.1 86.7 91.6 0.78 0.94 Dataset 3 (IND): 88.9 85 92.7 0.77 0.94**
		**Dee et al., 2022 (165)**	**Veltri Dataset: 1778 AMPs, 1778 non-AMPs LMPred Dataset: 3758 AMPs, 3758 non-AMPs**	**T5**	**CNN**	**Dataset: Acc Sn Sp MCC AUROC Veltri Dataset: 93.33 92.28 94.38 0.8668 97.89 LMPred Dataset: 88.26 88.89 87.63 0.7653 94.66**
		**Xiao et al., 2021 (329)**	**a) Dataset 1: 3594 AMPs, 3925 non-AMPs b) Dataset 2: ABPs=220, AVPs=190, AFPs=931, AHIV peptides=110 ACPs=245, ABFPs=51, APPs=124, CPs=62, AMRSAPs=173, AEPs=56**	**Random embedding**	**Hybrid (CNN+ BiLSTM+ SVM)**	**Dataset 1: Acc=0.9413, Sn=0.9547, Sp=0.9277, MCC=0.8829, P=0.9310, F1=0.0427 Dataset 2: Subset Acc=0.5585**
		**Lin et al., 2019 (330)**	**Dataset 1: 2618 AMPs, 4371 non-AMPs Dataset 2: 278 AMPs, 1382 non-AMPs Dataset 3: 18 Wound Healing, 13 Spermicidal, 28 Insecticidal, 57 Chemotactic, 593 Antifungal, 4 Anti-protist, 22 Antioxidant, 1297 Antibacterial, 32 Antibiotic, 25 Antimalarial, 101 Antiparasital, 125 Antiviral, 125 Anticancer, 109 Anti-HIV, 26 Proteinase inhibitor, 43 Surface immobilized**	**PCP+ AAC+ PAAC (amino acid composition based representation approach)**	**RF**	**Dataset 1: - Dataset 2: Acc=93.91, Sn= 92.83, Sp=94.99, MCC=0.878 Dataset 3: Acc=0.86, P=0.92, R=0.93**
		**Lin et al., 2016 (335)**	**Stage 1: A) Xiao et al. Dataset (Train): 879 AMPs, 2405 non-AMPs b) Xiao et al. Dataset (IND): 920 AMPs, 920 non-AMPs Stage 2: Xiao et al. Dataset: 770 ABPs, 140 ACPs, 336 AFPs, 84 anti-HIV Peptides, 124 AVPs**	**PAAC (amino acid composition based representation approach)**	**RF**	**Stage 1 Dataset: Acc Sn Sp MCC Xiao et al. Dataset (Train): 89.90 77.00 94.60 0.737 Xiao et al. Dataset (IND): 94.70 97.30 94.60 0.895 Stage 2 Subset Acc=0.4846, Acc=0.6864, P=0.8338, R=0.7631**
Binary Classification	Signal Peptides Identification	**Dumitrescu et al., 2023 (336)**	**a) Teufel et al. Dataset Sec/SPaseI (Label): eukaryotes (1995 SPs and 14 095 non-SPs), gram-negative bacteria (1274 SPs and 898 non-SPs), gram-positive bacteria (496 SPs and 223 non-SPs), archaea (84 SPs and 109 non-SPs) b) Teufel et al. Dataset Sec/Spase II (Label): eukaryotes (1995 SPs and 14 095 non-SPs), gram-negative bacteria (1274 SPs and 898 non-SPs), gram-positive bacteria (496 SPs and 223 non-SPs), archaea (84 SPs and 109 non-SPs) c) Teufel et al. DatasetTat/SPaseI (Label): eukaryotes (1995 SPs and 14 095 non-SPs), gram-negative bacteria (1274 SPs and 898 non-SPs), gram-positive bacteria (496 SPs and 223 non-SPs), archaea (84 SPs and 109 non-SPs)**	**LPE+ OHE**	**BERT encoder+ multi-head attention-based transformer decoder**	**Dataset: MCC a) Teufel et al. Dataset Sec/SPaseI (Label): 0.874 ± 0.009 0.851 ± 0.016 0.936 ± 0.032 0.741 ± 0.044 b) Teufel et al. Dataset Sec/Spase II (Label): -0.816 ± 0.005 0.883 ± 0.022 0.802 ± 0.044 c) Teufel et al. DatasetTat/SPaseI (Label): -0.957 ± 0.010 0.846 ± 0.01 0.869 ± 0.072**
		**Chen et al., 2021 (166)**	**a) SignalP 5.0 Sec/SPI (Label): 17 270 Eukaryotes: 2614 SP, 0 T, 0L, 14 656 N/C 923 Gram-positive: 189 SP, 95 T, 449 L, 190 N/C 2328 Gram-negative: 509 SP, 334 T, 1063 L, 422 N/C 237 Archaea: 60 SP, 27 T, 28 L, 122 N/C b) SignalP 5.0 Sec/SPII (Label): 17 270 Eukaryotes: 2614 SP, 0 T, 0L, 14 656 N/C 923 Gram-positive: 189 SP, 95 T, 449 L, 190 N/C 2328 Gram-negative: 509 SP, 334 T, 1063 L, 422 N/C 237 Archaea: 60 SP, 27 T, 28 L, 122 N/C c) SignalP 5.0 Tat/SPI (Label): 17 270 Eukaryotes: 2614 SP, 0 T, 0L, 14 656 N/C 923 Gram-positive: 189 SP, 95 T, 449 L, 190 N/C 2328 Gram-negative: 509 SP, 334 T, 1063 L, 422 N/C 237 Archaea: 60 SP, 27 T, 28 L, 122 N/C**	**ESM**	**BiLSTM**	**Dataset: MCC a) SignalP 5.0 Sec/SPI (Label): 0.901 0.975 0.876 0.922 b) SignalP 5.0 Sec/SPII (Label): - 0.946 0.945 0.936 c) SignalP 5.0 Tat/SPI (Label): - 0.916 0.983 0.972**
Binary Classification	Secreted peptides Identification	**Wang et al., 2023 (167)**	**SSPs Dataset**	**Transformer**	**GRU**	**Acc=0.9886, Sn=0.9889, Sp=0.9866, AUROC=0.9981, MCC=0.9755**
Binary Classification	Anti-Inflammatory Peptides Identification	**Gaffar et al., 2024 (338)**	**Gupta Dataset: 173 AIPs, 253 AIPs**	**AAC+ DPC+ PAAC+ APAAC+ QSOrder+ SOCN+ GTPC (amino acid composition based approach)**	**Voting (RF+ ETC+ XGB+ LightGBM+ CatBoost)**	**Acc=77.7**, **Sn=80.3, Sp=74.2, AUROC=53.6, MCC=87.1**
		**Raza et al., 2023 (168)**	**Manavalan Dataset: 1679 AIPs, 1679 non-AIP**	**Hybrid (Word2Vec+ BERT+ CTF)**	**SnTCN**	**Acc=90.2, Sn=87.17, Sp=93.57, AUROC=0.95, MCC=0.8**
		**Deng et al., 2022 (342)**	**Deng Dataset: 2642 AIPs, 3704 non-AIPs**	**DDE+ CKSAAP (K-Gap based representation approach)**	**Ensemble (RF+ ET)**	**Acc=0.701, Sn=0.658, Sp=0.743, AUROC=0.797, P=0.719**
		**Zhang et al., 2020 (339)**	**Gupta Dataset: 173 AIPs, 253 non-AIPs Manavalan Dataset: 1679 AIPs, 1679 non-AIP**	**AAC+ PSSM+ PP (amino acid composition based representation approach)**	**RF**	**Gupta Dataset: Acc=74.8, Sn=52.8, Sp=88.3, MCC=45.3 Manavalan dataset: Acc=76.2, Sn=55.5, Sp=89.9, AUROC=76.7, MCC=49.7**
		**Khatun et al., 2019 (341)**	**Manavalan Dataset: 1679 AIPs, 1679 non-AIP**	**AAI+ KSAAP+ structural features+ pKSAAP (amino acid structural and occurrence frequency based representation approach)**	**RF**	**Acc=77, Sn=61.8, Sp=87.1, AUROC=84, MCC=51.2**
		**Gupta et al., 2017 (340)**	**Gupta Dataset: 173 AIPs, 253 non-AIPs**	**TPC+ motif features**	**SVM**	**Acc=72, Sn=78.6, Sp=67.4, MCC=45**
Regression	Peptide-Binding Specificity Prediction	**Motmaen et al., 2023 (170)**	**Peptide-MHC Dataset**	**AlphaFold**	**LogR**	**Class I: AUROC=0.97**
Regression	Antibody Sequence Infilling	**Melnyk et al., 2023 (169)**	**1.n CoV-AbDab Dataset 2. SabDab Dataset**	**BERT+AlphaFold+IgFold**	**_**	**1. CoV-AbDab Training: Amino Acid Recovery=39.3, Diversity=60.2, Perplexity=5.7 2. CoV-AbDab + SabDab: Training: Amino Acid Recovery=37.3, Diversity=64.1, Perplexity=4.9 2. SabDab: Amino Acid Recovery=42.4, Diversity=57.4, Perplexity=3.9**

Among all representation learning approaches for this goal, amino acid composition approaches are most commonly used representation, followed by amino acid occurrence approaches. Amino acid composition approaches are used with RF, hybrid (SVM+RF) and k-means clustering algorithm for anti-microbial peptides identification (55, 330, 333–335) whereas, potential of amino acid composition approaches are also explored with BiLSTM, LightGBM, ensemble (GRNN+kNN+PNN+RF+SVM) classifiers and scoring card method for anti-cancer peptides identification (53, 322, 323, 325). Similarly, amino acid composition approaches are employed with RF and Voting (RF+ETC+XGB+LightGBM+CatBoost) classifiers for anti-inflammatory peptides identification (338, 339). Among all these predictive pipelines, amino acid composition approaches with RF classifier has achieved state-of-the-art performance for anti-inflammatory peptides identification (339). In addition, structural amino acid composition approaches with hybrid (CNN+LSTM+MAM) classifier and matrix reduction based approaches with RF classifier are used for anti-cancer peptides identification (326, 327). Similarly, amino acid occurrence approaches are used with ensemble (RF+BR+CC+MLkNN) classifier for anti-cancer peptides identification (324) and has achieved state-of-the-art performance. Moreover, amino acid structural and occurrence frequency based approaches with RF classifier and k-Gap based approaches with RF+ET classifier are employed for anti-inflammatory peptides identification (341, 342). In addition, potential of physico-chemical properties based approaches are explored with hybrid (LR+SVM) classifier and ProtDCal software based representation learning approaches are used with LSTM classifier for anti-microbial peptides identification (332) and anti-bacterial peptides identification (337), respectively. Moreover, combined potential of TPC and motif features is explored with SVM classifier for anti-inflammatory peptide identification (340).

Furthermore, standalone potential of OHE with LightGBM classifier and combined potential of OHE, BLOOSUM62, AAI, and PPC representation with a CNN classifier are explored for anti-microbial peptides identification (328, 331). Moreover, combined representation from OHE and LPE is used with ProtBERT classifier for signal peptides identification (336). Among all standalone and combined representation approaches, OHE with LightGBM classifier has achieved state-of-the-art performance for anti-microbial peptides identification (331). Besides this, Word2Vec is used with BiLSTM classifier for anti-bacterial peptides identification (54) and have achieved state-of-the-art performance. Moreover, potential of random embedding is explored with hybrid (CNN+BiLSTM+SVM) classifier for anti-microbial peptides identification (329) whereas potential of T5 representation with CNN classifier and ESM representation with BiLSTM classifier are explored for anti-microbial peptides identification (165) and signal peptides identification (166), respectively. Moreover, representation from transformer is used with GRU classifier and AlphaFold is employed with LogR classifier for secreted peptides prediction (167) and peptide-binding specificity prediction (170), respectively. Among all LLMs based predictive pipelines, transformer with GRU classifier and AlphaFold with LogR classifier have achieved state-of-the-art performance for secreted peptides prediction (167) and peptide-binding specificity prediction (170), respectively. Similarly, combined representation from AlphaFold, IgFold and BERT is used with self-classifier for antibody sequence infilling (169) and has achieved state-of-the-art performance. Moreover, combined potential of BERT language model, Word2Vec and CTF representation is explored with SnTCN classifier for anti-inflammatory peptides identification (168).

From all tasks in this goal, two tasks namely antibody sequence in-filling and anti-inflammatory peptides offer room for improvement. Based on current performance trends, potential of shallow neural network based word embeddings such as Word2Vec, FastText, or GloVe or graph embeddings such as LINE and HOPE can be explored with standalone deep neural networks or hybrid frameworks to raise the performance of these under-performing tasks.

Table 11 provides performance metrics for 12 AI-driven (language models, word embeddings) applications that are designed for drug analysis related tasks. This goal is oriented to binary classification and regression prediction applications that are developed by utilizing 13 unique representation learning approaches including transformer, BERT, ESM-2, BiLSTM, RoBERTa, ALBERT, Node2Vec, Word2Vec, Random Walk, PSSM, RDkiT, Mol2Vec, and algebraic graph features. Moreover, these predictive pipelines make use of 10 unique classifiers namely GCN, BiGRU, MLP, CNN, GRU and self-classifiers of BERT, Transformer, ESM-2, RoBERTa, and ALBERT.

**Table 11.** Drug analysis related two distinct protein sequence analysis tasks predictive pipelines performance

Task Type	Task Name	Author, Year [ref]	Dataset	Representation learning	Classifier	Performance Evaluation
Binary Classification	Drug-Protein Interaction Prediction	**Zhou et al., 2024 (171)**	**Zhou et al. Datasets: 1. BindingDB Dataset 2. Davis Dataset 3. Yamanishi et al. Datasets (Enzyme, GPCR, IC, NR)**	**Transformer**	_	**1. AUROC=0.966 ± 0.001, AURPC=0.963 ± 0.002 2. AUROC=0.984 ± 0.001, AUPRC=0.978 ± 0.003 3. AUROC=0.951 ± 0.001, AUPRC=0.953 ± 0.001 4. AUROC=0.947 ± 0.009, AUPRC=0.943 ± 0.007 5. AUROC=0.978 ± 0.005, AUPRC=0.978 ± 0.006 6. AUROC=0.935 ± 0.008, AUPRC=0.934 ± 0.008**
		**Zhang et al., 2024 (172)**	**Zhang et al. Datasets: 1. DrugBank Dataset 2. Epigenetic-regulators Dataset**	**ESM-2+Transformer**	_	**1. Acc=83.3, AUROC=91.1, Recall=83.0, AUPRC=90.8 2. Acc=59.6, AUROC=66.1, Recall=60.9, AUPRC=64.5**
		**Sun et al., 2023 (343)**	**Sun et al. Dataset**	**BiLSTM**	**CNN**	**AUROC=94.4%, AUPRC=49.4%**
		Yang et al., 2023 (173)	1. ER Dataset 2. Ion-C Dataset 3. RTK Dataset 4. GPCR Dataset	Transformer	_	1. RMSE=1.42, PCC=0.26 2. RMSE=1.47, PCC=0.02 3. RMSE=1.51, PCC=0.18 4. RMSE=1.30, PCC=0.39
		**Hu et al., 2022 (271)**	**DUD-E Dataset**	**Node2Vec+Word2Vec**	**BiGRU**	**AUROC=100%, Acc=99.2%, Precision=99.5%, Recall=98.5%, F1-score=99%, Sp=99.7%**
		**Xuan et al., 2022 (272)**	**Xuan et al. Dataset**	**Node2Vec+RandomWalk**	**MLP**	**(10-fold CV) AUROC=0.981, AUPRC=0.451**
Regression	Drug-Target Binding Affinity Prediction	**Xia et al., 2023 (174)**	**1. Xia et al. Dataset: Ki Dataset 2. Davis et al. Dataset**	**BERT**	_	**1. MSE=0.421, CI=0.891 2. MSE=0.203, CI=0.900**
		**Wang et al., 2023 (344)**	**Wang et al. Datasets: 1. PDBbind Dataset 2. CASF2016 Dataset**	**PSSM, RDkit, Mol2vec**	**CNN, GRU**	**1. PCC=0.774, CI=0.791, RMSE=0.110, MAE=0.887, SD=1.098 2. PCC=0.824, CI=0.811, RMSE=1.223, MAE=0.996, SD=1.159**
		**Zhu et al., 2023 (345)**	**Wang et al. Datasets: 1. Core 2016 Datasetset 2. Test71 Dataset**	**Algebraic graph features**	**MLP**	**1. RMSE=1.274, MSE=1.012, PCC=0.814, SD1.265, CI=0.806 2. RMSE=1.220, MSE=0.949, PCC=0.538, SD=1.146, CI=0.688**
		**Hu et al., 2022 (271)**	**Hu et al. Dataset: PDBbind Dataset**	**Node2Vec+Word2Vec**	**BiGRU**	**RMSE=1.538, PCC=0.71**
		Saadat et al., 2022 (175)	Tang et al. Dataset: KIBA Dataset	RoBERTa+BERT+ALBERT	_	CI=0.911, MSE=0.110, RMSE=0.333
		Lennox et al., 2021 (176)	1. Tang et al. Dataset: KIBA Dataset 2. Davis et al. Dataset	BERT + RoBERTa	GCN	1. MSE=0.149, CI=0.888, R2=0.761, AUPRC=0.838 2. MSE=0.199, CI=0.896, R2=0.741, AUPRC=0.806

Among all representation learning approaches, BERT and Transformer are most commonly used, followed by Node2Vec, RoBERTa and Word2Vec. Specifically, BERT is used with a self-classifier for drug-target binding affinity prediction (174). Moreover, combined potential of BERT and RoBERTa representation with GCN classifier and representation from all three BERT, RoBERTa, and ALBERT is used with a self classifier for a single task namely drug-target binding affinity prediction (175, 176). Among all BERT based predictive pipelines, BERT with a self-classifier has achieved state-of-the-art performance for drug-target binding affinity prediction (174). Furthermore, potential of transformer is explored with a self-classifier for drug-protein interaction prediction (171, 173) and has achieved state-of-the-art performance. Apart from this, combined representation from transformer and ESM-2 language model is utilized with a self-classifier for drug-protein interaction prediction (172). Moreover, combined potential of Node2Vec and Word2Vec with BiGRU classifier is explored for drug-protein interaction prediction (271) and drug-target binding affinity prediction (271). In addition, combined representation learning approach of Node2Vec and Random Walk with MLP classifier is used for drug-protein interaction prediction (272).

Furthermore, potential of BiLSTM is explored with a CNN classifier for drug-protein interaction prediction (343). Besides these representation learning approaches, combined potential of PSSM, RDkit and Mol2Vec are employed with hybrid (CNN+GRU) classifier for drug-target binding affinity prediction (344). Moreover, algebraic graph features based representation is used with MLP classifier for drug-target binding affinity prediction (345).

An in-depth analysis of existing predictive pipelines indicates that there is a significant room of improvement for drug-target binding affinity prediction. By observing performance trends across different goals, potential of heterogeneous graph transformers along with deep learning classifiers such as CNN or BiLSTM can enhance the performance of under-performing task.

Table 12 provides performance metrics for 31 AI-driven (language models, word embeddings, and domain specific) applications that are designed for gene analysis related tasks. This goal is oriented to binary, multi-class, and multi-label classification and regression prediction applications are developed by utilizing 21 unique representation learning and 20 unique classifiers. These representation learning approaches include GPT (177), Node2Vec (273, 277–282, 284–288, 290, 291, 293, 294), CP-N3 (346), LINE (185, 274, 293), SDNE (185, 274), HOPE (274), Struc2Vec (275), TransE (276), ComplEx (276), DistMult (276), Graph Transformer Network (178), DeepWalk (274, 280, 292, 295), Word2Vec (281, 287), Hyper2Vec (282), Transformer (179), RotateE (283), Opa2Vec (284), Random Watcher Walker (RW2) (289), BERT (180), Bias Random Walk (296) and ESM-1 (181). Unique classifiers involve IBK (273), LSTM (346), RF (274), LightGBM (274, 280), XGBoost (275), MLP (277, 279, 282, 289), LogR (278), SVM (281, 284–286, 288, 290, 295), Inductive Matrix Completion Algorithm (179), GraphSAGE (283), CNN (287), GCN (292, 347), GLM (294), ET (296), DBN (291), Cosine Similarity (293) and self classifier of GPT (177), Graph Transformer Network (178), BERT (180) and ESM-1 (181).

**Table 12.** Gene analysis related four distinct protein sequence analysis tasks predictive pipelines performance

Task Type	Task Name	Author, Year [ref]	Dataset	Representation learning	Classifier	Performance Evaluation
Multi-label/Multi-class Classification	Gene Phenotype Prediction	**Kafkas et al., 2023 (177)**	**1. Kafkas et al. Dataset 1–2. Kafkas et al. Dataset 2-3. Kafkas et al. Dataset 3**	**GPT**	_	**1. Gene set size=5: (0-shot ) AUPRC=0.985, (1-shot) AUROC=0.990 Gene set size=25: (0-shot) AUPRC = 0.770, (1-shot) AUROC=0.964 Gene set size=50: (0-shot) AUPRC = 0.723, (1-shot) AUROC=0.962 Gene set size=75: (0-shot) AUPRC = 0.681 (1-shot) AUROC=0.981 Gene set size=100: (0-shot) AUPRC = 0.559 (1-shot) AUROC=0.928 2. Gene set size=5: (1-shot) AUPRC=0.972, (1-shot) AUROC=0.991 Gene set size=25: (1-shot) AUPRC=0.856, (1-shot) AUROC=0.982 Gene set size=50: (0-shot) AUPRC = 0.806, (1-shot) AUROC=0.977 Gene set size=75: (1-shot) AUC=0.980 3. Gene set size=5: (1-shot ) AURPC=0.956, (1-shot) AUROC=0.991 Gene set size=25, (1-shot) AUPRC = 0.784, (1-shot) AUROC=0.979 Gene set size=50, (1-shot) AUPRC = 0.677, (1-shot) AUROC=0.973 Gene set size=75, (1-shot) , AUPRC = 0.595, (0-shot) AUROC=0.992 Gene set size=100, (1-shot) AUPRC = 0.539 (1-shot) AUROC=0.937**
		**Chen et al., 2021 (273)**	**Chen et al., 2016 Dataset**	**Node2Vec**	**IBk**	**Acc=0.5195, Hamming loss=0.1077, Exact Match=0.3646**
Binary Classification	Disease Genes Prediction	**Wang et al., 2024 (346)**	**Wang et al. Dataset 1**	**CP-N3**	**LSTM**	**Mean Average Precision 10=0.361, Mean Average Precision@50=0.370**
		**Wang et al., 2023 (274)**	**Wang et al. Dataset 2**	**LINE + SDNE + HOPE**	**Ensembl (RF + LightGBM)**	**AUROC=0.924 ± 0.001, AUPRC=0.934 ± 0.001, F1-score=0.857 ± 0.001, Acc=0.865 ± 0.001, Recall=0.811 ± 0.007, Sp=0.919 ± 0.007, Precision=0.909 ± 0.006, MCC=0.734 ± 0.002**
		Chu et al., 2023 (275)	Chu et al. Datasset	Struc2Vec	XGBoost	Recall=0.746, F1-score=0.679, Precision=0.781, AUPRC=0.740
		Vilela et al., 2023 (276)	Vilela et al. Dataset	ComplEx + DistMult + TransE	_	Mean Rank=0.13, Mean Reciprocal Rank=0.96
		**Ratajczak et al., 2023 (277)**	**Ratajczak et al. Dataset (Cardiovascular Disease, Immune Dysregulation, Body Mass Disorder, Diabetes, Insulin Disorder)**	**Node2Vec**	**MLP**	**Cardiovascular Disease: AUROC=0.75, Immune Dysregulation: AUROC=0.73, Body Mass Disorders: AUROC=0.71, Diabetes: AUROC=0.77, Insulin Disorders: AUROC=0.72**
		**Jagodnik et al., 2023 (278)**	**Jagodnik et al. Dataset**	**Node2Vec**	**LogR**	**Recall=0.93, Precision=0.97, F1-score=0.95**
		Zhang et al., 2023 (279)	Zhang et al. Dataset	Node2Vec	MLP	AUROC=93.84, Acc=90.64, F1-score=90.84, Precision=86.48, Recall=95.66, AUPRC=92.86
		**Li et al., 2023 (178)**	**Li et al. Dataset**	**Graph Transformer Network**	_	**AUROC=0.9750, AUPRC=0.9649**
		**Wang et al., 2022 (280)**	**Yang et al. Dataset**	**Node2Vec + DeepWalk + LINE + SDNE**	**LightGBM**	**AUROC=0.9853, Acc=0.9349, F1-score=0.9350, Precision=0.9347, Recall=0.9352**
		Gao et al., 2022 (347)	Gao et al. Dataset	_	GCN	AUROC=0.978
		**Lu et al., 2022 (281)**	**Pancan TCGA Dataset**	**Word2Vec + Node2Vec**	**SVM**	**Weighted F1-score=0.6200**
		Wang et al., 2022 (282)	Wang et al. Dataset 3	Node2Vec + Hyper2Vec	MLP	AUROC=0.85459, AUPRC=0.79411, F1-score=0.75033, Acc=0.75661
		Wang et al., 2022 (179)	Wang et al. Dataset 4	Transformer	Inductive Matrix Completion Algorithm	AUROC= 0.9616 ± 0.0003, AUPRC=0.9564 ± 0.0002, Acc=0.8955 ± 0.0007, Precision=0.8737 ± 0.0005, Recall=0.9248 ± 0.0009, F1-score=0.8985 ± 0.0014
		Prabhakar et al., 2022 (283)	Prabhakar et al. Dataset	RotatE	GraphSAGE	Mean Rank=346.81
		Wang et al., 2021 (284)	Wang et al. Dataset 5	Node2Vec + Opa2Vec	SVM	AUROC=0.94707(0.01904), AUPRC=0.88969(0.01775), F1-score=0.94707(0.01904), Acc=0.86667(0.05666)
		Liu et al., 2021 (285)	Liu et al. Dataset 1	Node2Vec	SVM	AUROC=0.731
		Du et al., 2021 (286)	Du et al. Dataset	Node2Vec	SVM	AUROC=0.74, AUPRC=0.72
		Xu et al., 2021 (287)	Xu et al. Dataset	Word2Vec + Node2Vec	CNN	Hamming loss = 0.23, Jaccard similarity = 0.34, micro AUROC = 0.78, macro AUROC =0.78, micro Average Precision = 0.47, macro Average Precision = 0.48, micro F1-score = 0.48, and macro F1-score = 0.39
		**Liu et al., 2020 (288)**	**Liu et al. Dataset 2**	**Node2Vec**	**SVM**	**Acc= 0.7011 ± 0.0212, F1-score=0.6944 ± 0.0138, AUROC=0.7647 ± 0.0186, AUPRC=0.7472 ± 0.0283**
		Madeddu et al., 2020 (289)	Madeddu et al. Dataset	Random Watcher-Walker (RW2 )	MLP	Recall 1=36%, STD=0.008
		**Li et al., 2020 (180)**	**1. ClinVar_BRCA1 Dataset 2. ClinVar_PTEN Dataset**	**BERT**	_	**1. Acc=0.890, AUROC=0.920, AUPRC=0.717, Recall=0.861, Precision=0.778, F1-score=0.815 2. Acc=0.853, AUROC=0.909, AUPRC=0.958, Recall=0.875, Precision=0.884, F1-score=0.879**
		Peng et al., 2019 (290)	Peng et al. Dataset	Node2Vec	SVM	AUROC varies between 0.69 and 0.73
		**Luo et al., 2019 (291)**	**Luo et al. Dataset**	**Node2Vec**	**DBN**	**AUROC = 0.969**
		Zhu et al., 2019 (292)	Zhu et al. Dataset	DeepWalk	GCN	Average Precision=0.411; Top 3: (Precision=0.283, Recall=0.361, F1-score=0.266); Top 10: (Precision=0.147, Recall=0.494, F1-score=0.188)
		Yang et al., 2018 (293)	Yang et al. Dataset	Node2Vec + LINE	Cosine Similarity	AP=0.294 ± 0.005; Top@3: Precision=0.243 ± 0.003, Recall=0.325 ± 0.004, F1-score=0.233 ± 0.003; Top 10: Precision=0.124 ± 0.002, Recall=0.477 ± 0.008, F1-score=0.167 ± 0.003;
		Ata et al., 2018 (294)	Ata et al. Dataset	Node2Vec	GLM	In form of graph
Binary Classification	Essential Genes Identification	**Kuru et al., 2022 (295)**	**Kuru et al. Dataset**	**DeepWalk**	**SVM**	**Acc=0.885, AUROC=0.884 F1-score=0.687, Average Precision=0.514**
		Dai et al., 2020 (296)	1. FIs Dataset 2. InWeb_IM Dataset	Bias Random Walk	ET	1. Essential non-essential gene ratio (1:4): F1-score=0.692, MCC=0.641, Acc=0.893, AUROC=0.913, Avg Precision=0.769 Essential non-essential gene ratio (1:6): F1-score=0.847, MCC=0.699, Acc=0.849, AUROC=0.914, Avg Precision=0.902 2. Essential non-essential gene ratio (1:6): F1-score=0.665, MCC=0.641, Acc=0.921, AUROC=0.915, Avg Precision=0.762 Essential non-essential gene ratio (1:1): F1-score=0.857, MCC=0.713, Acc=0.856, AUROC=0.928, Avg Precision=0.921
Binary Classification	Prokaryotic Gene Prediction	**Tu et al., 2023 (181)**	**Tu et al. Dataset**	**ESM-1**	_	**Acc=0.933+0.009, Precision=0.930+0.019, Recall=0.928 + 0.012, F1-score=0.929 + 0.009**

A comprehensive analysis reveals that Node2Vec is most commonly used representation learning approach followed by LINE and DeepWalk. Specifically, Node2Vec is used with IBK classifier for gene phenotype prediction (273) whereas potential of Node2Vec is also explored with MLP, LogR, SVM, DBN and GLM classifiers for disease genes prediction (277–279, 285, 286, 288, 290, 291, 294). In addition, combined potential of Node2Vec, DeepWalk, LINE and SDNE is explored with LightGBM classifier for disease genes prediction (280). Moreover, combined representation from Node2Vec and Word2Vec is used with SVM and CNN classifiers for disease genes prediction (281, 287). Whereas, combined potential of Node2Vec and Hyper2Vec is employed with MLP classifier and combined potential of Node2Vec and Opa2Vec is explored with SVM classifier for disease genes prediction (282, 284). Among all Node2Vec based predictive pipelines, not a single pipeline has achieved state-of-the-art performance. In addition, DeepWalk with GCN and SVM classifiers is employed for disease genes prediction (292), and essential genes identification (295). Among both, Deepwalk with SVM classifier has achieved state-of-the-art performance for essential genes identification (295).

Moreover, Stru2Vec is used with XGBoost classifier, TransE, ComplEx, and DistMult are employed with MLP classifier and RotatE is utilized with GraphSAGE classifier for disease genes prediction (275, 276, 276, 283). Furthermore, Random Watcher Walker (*RW*^2^) is employed with GCN and MLP classifier and potential of Bias Random Walk is explored with ET classifier for disease genes prediction (289) and essential genes identification (296), respectively. In addition, combined potential of LINE, SDNE and HOPE is also explored with ensemble (RF+LGBM) classifier for disease genes prediction (274). Apart from this, CP-N3 representation is used with LSTM classifier for disease genes prediction and has achieved state-of-the-art performance (346). Furthermore, potential of Transformer based representation is explored with Inductive Matrix Completion Algorithm whereas Graph Transformer Network and BERT is employed with a self-classifier for disease genes prediction (178–180). Moreover, GPT is used with a self-classifier for gene phenotype prediction and has achieved state-of-the-art performance (177). ESM-1 is also used with a self-classifier for prokaryotic gene prediction (181) and has achieved state-of-the-art performance.

From all these tasks, essential genes identification has significant room for improvement. Taking into account the performance trends of different tasks in this goal, potential of LLMs such as ESM-1 and GPT with a self-classifier can enhance the performance of under-performing tasks.

Table 13 provides performance metrics for 19 AI-driven (language models, word embeddings, and domain specific) applications that are designed for protein properties prediction related tasks. This goal is oriented to binary, multi-class, and multi-label classification and regression prediction applications are developed by utilizing seven unique representation learning approaches namely Transformer (155, 185), CTAPAAC (348), ESM-1 (182), T5(27, 187), RoBERTa (183), ESM-2 (150, 184) and BERT (154, 186, 188, 189). Moreover, these predictive pipelines have used 10 unique classifiers including RF (348), GCN (182), CNN (184), BiLSTM (189), ResNet (155) and self-classifiers of ESM-2 (150), T5 (27, 187), RoBERTa (445), Transformer (185) and BERT (154, 186, 188).

**Table 13.** Protein properties prediction related five distinct protein sequence analysis tasks predictive pipelines performance

Task Type	Task Name	Author, Year [ref]	Dataset	Representation learning	Classifier	Performance Evaluation
Binary Classification	Solubility Prediction	Li et al., 2024 (150)	Khurana et al. Dataset	ESM-2	_	Acc=79.45
		**Mehmood et al., 2023 (348)**	**1. Bhandari et al. PSI: biology Dataset 2. Niwa et al. Dataset 3. Smialowski et al. e-coli Dataset 4. Price et al. Dataset**	**CTAPAAC**	**RF**	**1. Acc=77, AUROC=80, MCC=44.8 2. Acc=88.6, AUROC=92.4, Precision=88.7, Recall=88.6, F1-score=88.6 3. Acc=85.9, Sn=85.8, Sp=85.8, MCC=74.2 4. Acc=98.6, Precision=98.6, MCC=96.9, AUROC=99.9**
		**Chen et al., 2023 (182)**	**S. cerevisiae Dataset**	**ESM-1**	**GCN**	**R2= 0.390**
		Elnaggar et al., 2023 (27)	Khurana et al. Dataset	T5	_	Acc=76.4 ± 2
		Filipavicius et al., 2020 (183)	Khurana et al. Dataset	RoBERTa	_	Acc=0.583
Regression	Stability Prediction	**Li et al., 2024 (150)**	**Rockline et al. Dataset**	**ESM-2**	_	**SRCC=84.21**
		**Gong et al., 2023 (184)**	**1. S2648 2. Ssym 3. S669 4. CAGI5 Challenge’s Frataxin**	**ESM-2**	**CNN**	**1. Direct: PCC=0.76, Acc=0.86, RMSE=0.88 Reverse: PCC=0.77, Acc=0.87, RMSE=0.87 Direct+Reverse: PCC=0.86, Acc=0.87, RMSE=0.88 2. Direct: PCC=0.76, Acc=0.84, RMSE=1.24 Reverse: PCC=0.76, Acc=0.82, RMSE=1.25 Direct+Reverse: PCC=0.86, Acc=0.83, RMSE=1.25 3. Direct: PCC=0.39, Acc=0.74, RMSE=1.60 Reverse: PCC=0.35, Acc=0.73, RMSE=1.66 Direct+Reverse: PCC=0.53, Acc=0.74, RMSE=1.63 4. Direct: PCC=0.67, Acc=0.62, RMSE=3.55 Reverse: PCC=0.72, Acc=0.75, RMSE=3.55 Direct+Reverse: PCC=0.80, Acc=0.69, RMSE=3.55**
		Wang et al., 2023 (185)	Rockline et al. Dataset	Transformer	_	SRCC=0.767
		Wang et al., 2022 (186)	Rockline et al. Dataset	BERT	_	SRCC=0.730 ± 0.005
		Xiao et al., 2021 (154)	Rockline et al. Dataset	BERT	_	SRCC=0.79
		Rao et al., 2019 (155)	Rockline et al. Dataset	Transformer	ResNet	SPCC=0.73
Binary Classification	Thermophilicity Prediction	**Haselbeck et al., 2023 (187)**	**Haselbeck et al. Dataset**	**T5**	_	**Acc=0.970 ± 0.004, F1-score=0.955 ± 0.005, Precision=0.963 ± 0.015, Recall=0.947 ± 0.005, Sp=0.982 ± 0.008, MCC=0.933 ± 0.008**
Regression	Fluorescence Prediction	**Wang et al., 2023 (185)**	**Sarkisyan et al. Dataset**	**Transformer**	_	**SRCC=0.683**
		Elnaggar et al., 2023 (27)	Sarkisyan et al. Dataset	T5	_	SRCC=0.62 ± 0.4
		Wang et al., 2022 (186)	Sarkisyan et al. Dataset	BERT	_	SRCC=0.680 ± 0.003
		Brandes et al., 2022 (188)	Sarkisyan et al. Dataset	BERT	_	SRCC=0.66
		Xiao et al., 2021 (154)	Sarkisyan et al. Dataset	BERT	_	SRCC=0.68
		Rao et al., 2019 (155)	Sarkisyan et al. Dataset	Transformer	ResNet	SRCC=0.68
Multi-class Classification	Domain Boundary Prediction	Haseeb et al., 2023 (189)	1. FM Dataset 2. Multi-Domain Dataset 3. DCD Dataset	BERT	BiLSTM	FM Dataset: Acc=0.74, Precision=0.74, Recall=0.47, F1-score=0.58 Multi-Domain Dataset: Acc=0.76, Precision=0.82, Recall=0.45, F1-score=0.58 DCD Dataset: Acc=0.70, Precision=0.82, Recall=0.33, F1-score=0.47

Among all representation learning approaches, BERT is the most commonly used followed by transformer. Specifically, BERT is employed with a self-classifier for stability prediction (154, 186), and fluorescence prediction (154, 186, 188), and potential of BERT representation is also explored with BiLSTM classifier for domain boundary prediction (189). Among all BERT based predictive pipelines, BERT representations with BiLSTM have achieved state-of-the-art performance. Similarly, Transformer is used with a self-classifier for solubility prediction (150), stability prediction (150, 155, 185) and fluorescence prediction (155, 185). For all three tasks, transformer based predictive pipeline with a self classifier manages to achieve top performing values. Moreover, T5 is employed with a self-classifier for solubility prediction (27), thermophilicity prediction (187) and fluorescence prediction (27), and has achieved state-of-the-art performance for thermophilicity prediction (187). Beyond BERT and T5 language model, potential of ESM-2 representation is utilized with CNN for stability prediction (184), and also employed with self-classifier for both stability prediction (150) and solubility prediction (150). Additionally, ESM-1 representation is utilized with GCN classifier for solubility prediction (182). In addition, RoBERTa is used with a self-classifier for solubility prediction (183), and potential of CTAPAAC representation is also explored with RF classifier for solubility prediction (348).

From all tasks in this goal, fluorescence prediction offers a significant potential for improvement. Considering the performance trends across different goals, potential of shallow and graph neural network based word embedding such as FastText, Word2Vec, DeepWalk, and Node2Vec with deep learning classifiers including CNN, BiLSTM and BiGRU can improve the performance of under-performing task.

Table 14 summarizes 54 predictive pipelines related to protein function and structure prediction goal. For this goal, predictive pipelines have used 19 unique representation learning approaches namely transformer, ESM-1, ESM-2, BERT, T5, AlphaFold, KG, ELMo, FastText, Word2Vec, Node2Vec, Mashup, GRU, OHE, XLNet, ALBERT, ELECTRA, CNN, and Transformer-XL. Moreover, these predictive pipelines have employed 23 unique classifiers including ANN, CNN, GNN, BiLSTM, DNN, RF, GAN, GCN, MLP, SVM, GVP, LogR, MCNN and self-classifier of transformer, BERT, ESM-1, ESM-2, T5, AlphaFold, XLNet, ALBERT, ELECTRA, and Transformer-XL.

**Table 14.** Protein function and structure related five distinct protein sequence analysis tasks predictive pipelines performance

Task Type	Task Name	Author, Year [ref]	Dataset	Representation learning	Classifier	Performance Evaluation
Multi-label Classification	Protein Function Identification	Chen et al., 2024 (150)	1. Antibiotic Resistance CARD Dataset, 2. Fluorescence TAPE Dataset, 3. Fitness Dataset	ESM-2	–	1: 19-cls Acc = 98.38, 2: SRCC = 66.00, 3: SRCC = 96.10
		Tawfiq et al., 2024 (190)	Tawfiq et al. Dataset (MF, BP, CC)	ESM-2	–	MF: F1-score = 0.468, Smin = 12.230, AUPRC = 0.449, AUROC = 0.874, BP: F1-score = 0.476, Smin = 19.394, AUPRC = 0.462, AUROC = 0.847, CC: F1-score = 0.739, Smin = 3.394, AUPRC = 0.759, AUROC = 0.903
		Islam et al., 2024 (349)	Islam et al. Dataset (BP, CC, MF)	KG	ANN	BP; Precision =1, Recall = 1, F1-score = 1, Acc = 96, CC; Precision =1, Recall = 1, F1-score = 1, Acc = 97, MF; Precision =1, Recall = 1, F1-score = 1, Acc = 98
		**Song et al., 2024 (191)**	**CAFA3**	**ESM-1 + Transformer**	**CNN**	**MF: F1-score = 0.601, AUPRC = 0.559, Smin = 6.709, CC: F1-score = 0.643, AUPRC = 0.634, Smin = 7.037, BP: F1-score = 0.518, AUPRC = 0.441, Smin = 18.753**
		**Yuan et al., 2024 (192)**	**You et al. Dataset (MF, BP, CC)**	**ESM-2 + T5**	**GNN**	**MF: F1-score = 0.641, AUPRC = 0.623, BP: F1-score= 0.336, AUPRC = 0.243, CC: F1-score= 0.726, AUPRC = 0.767**
		**Zhapa et al., 2024 (193)**	**Zhapa et al. Dataset (MF, BP, CC)**	**ESM-2**	–	**similarity-based split setting MF: F1-score = 0.5317, Smin = 11.6490, AUPRC = 0.5026, AUROC = 0.8413, BP: F1-score = 0.4365, Smin = 36.7640, AUPRC = 0.3928, AUROC = 0.8674, CC: F1-score = 0.7210, Smin = 9.1010, AUPRC = 0.7696, AUROC = 0.9240, time-based split setting MF: F1-score = 0.533, Smin = 9.232, AUROC = 0.943, BP: F1-score = 0.559, Smin = 22.730, AUROC = 0.900, CC: F1-score = 0.750, Smin = 6.323, AUROC = 0.952**
		**Kulmanov et al., 2024 (194)**	**1. Kulmanov et al. Dataset (MF, BP, CC), 2. Kulmanov et al. Dataset neXtProt (MF BP)**	**ESM-2**	–	**1: MF: F1-score = 0.554, Smin = 11.681, AUPRC = 0.552, AUROC = 0.874, BP: F1-score = 0.432, Smin = 39.419, AUPRC = 0.401, AUROC = 0.864, CC: F1-score = 0.721, Smin = 9.499, AUPRC = 0.730, AUROC = 0.914, 2: neXtProt MF: F1-score = 0.386, Smin = 10.093, AUPRC = 0.324, AUROC = 0.744, neXtProt BP: F1-score = 0.349, Smin = 30.170, AUPRC = 0.312, AUROC = 0.683**
		Shaw et al., 2024 (195)	1. Shaw et al. Dataset Random EC, 2. Shaw et al. Dataset Random GO, 3. Shaw et al. Dataset Clustered EC, 4. Shaw et al. Dataset Clustered GO, 5. Shaw et al. Dataset NEW-392, 6. Shaw et al. Dataset Price-149, 7. Shaw et al. Dataset PDB EC, 8. Shaw et al. Dataset Clustered Pfam	T5	–	1: F1-score = 0.987, 2: F1-score = 0.917, 3: F1-score = 0.958, 4: F1-score = 0.854, 5: NEW-392: Weighted AUROC for EC = 0.932, 6: Price-149: Weighted AUROC for EC = 0.842, 7: PDB Protein-centric F1-score for EC = 0.909, 8: Clustered Pfam Family Acc = 92.6, Lifted Clan Acc = 93.3, Average Per-Family Acc = 91.7
		Chua et al., 2024 (196)	Chua et al. Dataset (BP, CC, MF)	OHE + ESM-2 + T5 + BERT	DNN	–
		Zhang et al., 2024 (197)	Zhang et al. Dataset (CC, BP, MF)	ESM-2	RF	Average Weighted F1-score = 0.604, CC: Weighted F1-score = 0.729, BP: Weighted F1-score = 0.445, MF: Weighted F1-score = 0.639
		**Zhao et al., 2024 (198)**	**1. Zhao et al. Dataset Human (CC, MF, BP), 2. Zhao et al. Mouse (CC, MF, BP), 3. Zhao et al. Arabidopsis (CC, MF, BP)**	**ESM-1**	**GAN**	**1: CC: micro Average F1-score = 86.81 ± 0.05, macro Average F1-score = 85.01 ± 0.06, AUROC = 89.29 ± 0.15, AUPRC = 90.32 ± 0.17, F1-score = 83.83 ± 0.04, MF: micro Average F1-score = 83.60 ± 0.07, macro Average F1-score = 85.89 ± 0.02, AUROC = 87.07 ± 0.25, AUPRC = 87.84 ± 0.18, F1-score = 80.58 ± 0.11, BP: micro Average F1-score = 81.74 ± 0.41, macro Average F1-score = 85.90 ± 0.10, AUROC = 88.39 ± 0.29, AUPRC = 85.17 ± 0.10, F1-score = 79.28 ± 0.11, 2: CC: micro Average F1-score =84.98 ± 0.02, macro Average F1-score =90.80 ± 0.01, AUROC = 88.64 ± 0.04, AUPRC = 88.80 ± 0.18, F1-score = 83.74 ± 0.13, MF: micro Average F1-score = 77.44 ± 0.27, macro Average F1-score = 86.05 ± 0.04, AUROC = 81.07 ± 0.12, AUPRC = 82.01 ± 0.71, F1-score = 77.16 ± 0.15, BP: micro Average F1-score = 82.86 ± 0.65, macro Average F1-score = 87.82 ± 0.14, AUROC = 87.52 ± 0.03, AUPRC = 88.22 ± 1.47, F1-score = 81.57 ± 0.33, 3: CC: micro Average F1-score = 81.05 ± 0.29, macro Average F1-score = 89.86 ± 0.33, AUROC = 94.58 ± 0.16, AUPRC = 95.53 ± 0.28, F1-score = 90.67 ± 0.26, MF: micro Average F1-score = 86.36 ± 0.36, macro Average F1-score = 89.66 ± 0.05, AUROC = 90.87 ± 0.54, AUPRC = 92.22 ± 0.28, F1-score = 87.30 ± 0.06, BP: micro Average F1-score = 84.77 ± 0.15, macro Average F1-score = 87.47 ± 0.30, AUROC = 90.42 ± 0.08, AUPRC = 91.21 ± 0.43, F1-score = 83.37 ± 0.08**
		**Pang et al., 2024 (199)**	**1. Pang et al. Dataset DP93 (Protein Binding, DNA Binding, RNA Binding, Ion Binding, Lipid Binding, Flexible linker functional sites), 2. Pang et al. Dataset DP94 (Protein Binding, DNA Binding, RNA Binding, Ion Binding, Lipid Binding, Flexible linker functional sites)**	**T5**	**GCN**	**1: Protein Binding: AUROC=0.839, AUPRC=0.768, F1-score=0.434, MCC=0.370, Acc=0.768, DNA Binding: AUROC=0.896, AUPRC=0.821, F1-score=0.152, MCC=0.181, Acc=0.821, RNA Binding: AUROC=0.908, AUPRC=0.850, F1-score=0.207, MCC=0.222, Acc=0.850, Ion Binding: AUROC=0.700, AUPRC=0.695, F1-score=0.028, MCC=0.069, Acc=0.695, Lipid Binding: AUROC=0.861, AUPRC=0.771, F1-score=0.304, MCC=0.328, Acc=0.771, Flexible linker: AUROC=0.897, AUPRC=0.833, F1-score=0.403, MCC=0.389, Acc=0.833, 2: Protein Binding: AUROC=0.846, AUPRC=0.285, F1-score=0.357, MCC=0.327, Balanced Acc = 0.784, DNA Binding: AUROC=0.716 , AUPRC=0.004, F1-score=0.016, MCC=0.035, Balanced Acc = 0.685, RNA Binding: AUROC=0.833 , AUPRC=0.006 , F1-score=0.016, MCC=0.064, Balanced Acc = 0.801, Ion Binding: AUROC=0.930, AUPRC=0.706, F1-score=0.817, MCC=0.829, Balanced Acc = 0.849, Lipid Binding: AUROC=0.800, AUPRC=0.021, F1-score=0.067, MCC=0.085, Balanced Acc = 0.701, Flexible linker: AUC= 0.762, AUPR= 0.110, Fmax=0.184, MCC= 0.167, BAcc = 0.685**
		**Wang et al., 2023 (185)**	**Wang et al. Dataset (BP, CC, MF, EC)**	**Transformer**	–	**BP: AUPRC = 0.363, F1-score = 0.495, CC: AUPRC = 0.457, F1-score = 0.551, MF: AUPRC = 0.665, F1-score = 0.677, EC: AUPRC = 0.915, F1-score = 0.888**
		Derbel et al., 2023 (200)	1. AMIE Dataset, 2. B3VI55_LIPSTSTABLE Dataset, 3. B3VI55_LIPST Dataset, 4. BF520 Dataset, 5. BG505 Dataset, 6. BG_STRSQ Dataset, 7. BLAT_2014 Dataset, 8. BLAT_2012 Dataset, 9. BLAT_2015 Dataset, 10. BLAT_2013 Dataset, 11. BRCA1_BRCT Dataset, 12. BRCA1_RING Dataset, 13. CALM1_Roth2017 Dataset, 14. DLG4_RAT Dataset, 15. GAL4 Dataset, 16. HG_FLU Dataset, 17. HSP82 Dataset, 18. IF1_ECOLI Dataset, 19. MK01 Dataset, 20. MTH3 Dataset, 21. P84126 Dataset, 22. PABP Dataset, 23. PA_FLU Dataset, 24. POLG_HCVJF Dataset, 25. PTEN Dataset, 26. RASH Dataset, 27. RL401_2013 Dataset, 28. RL401_2014 Dataset, 29. RL401_2016 Dataset, 30. SUMO1 Dataset, 31. TIM_SULSO Dataset, 32. TIM_THEMA Dataset, 33. TPK1_2017 Dataset, 34. TPMT_2018 Dataset, 35. UBC9 Dataset, 36. UBE4B Dataset, 37. YAP1 Dataset, 38. HIV_Tat Dataset	ESM-2	–	1. AMIE Dataset SRCC = 0.806, 2. B3VI55_LIPSTSTABLE Dataset SRCC = 0.73, 3. B3VI55_LIPST Dataset SRCC = 0.491, 4. BF520 Dataset SRCC = 0.803, 5. BG505 Dataset SRCC = 0.829, 6. BG_STRSQ Dataset SRCC = 0.66, 7. BLAT_2014 Dataset SRCC = 0.843, 8. BLAT_2012 Dataset SRCC = 0.813, 9. BLAT_2015 Dataset SRCC = 0.882, 10. BLAT_2013 Dataset SRCC = 0.743, 11. BRCA1_BRCT Dataset SRCC = 0.588, 12. BRCA1_RING Dataset SRCC = 0.647, 13. CALM1_Roth2017 Dataset SRCC = 0.316, 14. DLG4_RAT Dataset SRCC = 0.755, 15. GAL4 Dataset SRCC = 0.716, 16. HG_FLU Dataset SRCC = 0.714, 17. HSP82 Dataset SRCC = 0.719, 18. IF1_ECOLI Dataset SRCC = 0.74, 19. MK01 Dataset SRCC = 0.584, 20. MTH3 Dataset SRCC = 0.701, 21. P84126 Dataset SRCC = 0.832, 22. PABP Dataset SRCC = 0.804, 23. PA_FLU Dataset SRCC = 0.452, 24. POLG_HCVJF Dataset SRCC = 0.78, 25. PTEN Dataset SRCC = 0.706, 26. RASH Dataset SRCC = 0.825, 27. RL401_2013 Dataset SRCC = 0.821, 28. RL401_2014 Dataset SRCC = 0.726, 29. RL401_2016 Dataset SRCC = 0.75, 30. SUMO1 Dataset SRCC = 0.633, 31. TIM_SULSO Dataset SRCC = 0.768, 32. TIM_THEMA Dataset SRCC = 0.758, 33. TPK1_2017 Dataset SRCC = 0.373, 34. TPMT_2018 Dataset SRCC = 0.572, 35. UBC9 Dataset SRCC = 0.712, 36. UBE4B Dataset SRCC = 0.523, 37. YAP1 Dataset SRCC = 0.744, 38. HIV_Tat Dataset SRCC = 0.89
		**Zhang et al., 2023 (297)**	**Zhang et al. Dataset (MF, BP, CC)**	**ELMo**	**MLP**	**BP: F1-score=0.561, AUROC=0.909, AUPRC=0.625, CC: F1-score=0.748, AUROC=0.971, AUPRC=0.812, MF: F1-score=0.697, AUROC=0.959, AUPRC=0.771**
		**Ali et al., 2023 (298)**	**1. STCRDAB Dataset, 2. PDB Bind Dataset**	**ELMo**	**SVM+LogR**	**1: Acc=0.991, Precision=0.990, Recall=0.991, Weighted F1-score=0.990, macro F1-score=0.988, AUROC=0.985, 2: Acc=0.968, Precision=0.972, Recall=0.968, Weighted F1-score=0.969, macro F1-score=0.966, AUROC=0.980**
		**Pang et al., 2023 (201)**	**1. CAID Dataset (DisProt, DisProt-PDB, disordered region, disordered proteins functions), 2. TE176 Dataset (Protein binding, DNA binding, RNA binding, Flexible linker disordered functions)**	**BERT + T5**	–	**1: disordered region prediction on the CAID DisProt Dataset: AUROC=0.833, F1-score=0.516, MCC=0.415, Balanced Acc=0.762, disordered region on the CAID DisProt-PDB Dataset: AUROC=0.910, F1-score=0.766, MCC=0.662, Balanced Acc=0.836, disordered proteins on the CAID DisProt Dataset: F1-score = 0.680, MCC = 0.657, TNR = 0.969, TPR = 0.733, PPV = 0.635, Balanced Acc = 0.851, disordered Binding Sites: AUROC=0.792, F1-score=0.260, MCC=0.239, Acc=0.730, 2: disordered Protein Binding: AUROC=0.824, F1-score=0.473, MCC=0.403, disordered DNA Binding: AUROC=0.897, F1-score=0.176, MCC=0.208, disordered RNA Binding: AUROC=0.883, F1-score=0.262, MCC=0.259, disordered flexible linker: AUROC=0.748, F1-score=0.263, MCC=0.250**
		**Pei et al., 2023 (202)**	**1. Ahmed et al. Dataset Benchmark, 2. Ahmed et al. Dataset Balanced Test Set**	**BERT**	**LogR**	**1: Acc=0.9751, Sn=0.9853, Sp=0.9654, MCC=0.9504, AUROC=0.9935, 2: Acc=0.913, Sn=0.826, Sp=0.916, MCC=0.910, AUROC=0.972**
		**Yuan et al., 2023 (203)**	**Yuan et al. Dataset (MF, BP, CC)**	**T5**	–	**MF : F1-score=0.647, AUPRC=0.622, BP: F1-score=0.335, AUPRC=0.247, CC: F1-score=0.725, AUPRC=0.765**
		**Zhao et al., 2023 (204)**	**1. Yeast (BP, MF, CC) Dataset, 2. Human (BP, MF, CC) Dataset, 3. Arabidopsis (BP, MF, CC) Dataset**	**ESM-1**	**GCN**	**1: BP: Smin=0.75637, F1-score=0.5406, AUROC=0.7727, AUPRC=0.5595, MF: Smin=0.39593, F1-score=0.6182, AUROC=0.8464, AUPRC=0.6094, CC: Smin=0.53249, F1-score=0.7238, AUROC=0.8154, AUPRC=0.7452, 2: BP: Smin=0.186452, F1-score=0.4895, AUROC=0.7483, AUPRC=0.4733, MF: Smin=0.69805, F1-score=0.6900, AUROC=0.8534, AUPRC=0.6679, CC: Smin=0.72587, F1-score=0.6837, AUROC=0.8154, AUPRC=0.6917, 3: BP: Smin=0.93192, F1-score=0.5309, AUROC=0.7663, AUPRC=0.5342, MF: Smin=0.34015, F1-score=0.7195, AUROC=0.9036, AUPRC=0.53427544, CC: Smin=0.37739, F1-score=0.7944, AUROC=0.8560, AUPRC=0.8137**
		**Wu et al., 2023 (299)**	1. **Wu et al., Dataset Yeast (BP, MF, CC), 2. Wu et al. Dataset Human (BP, MF, CC)**	**Mashup**	**SVM**	**1: BP: Acc=67.34, F1-Score=46.47, AUPRC=65.76, MF: Acc=61.13, F1-Score=43.94, AUPRC=61.57, CC: Acc=76.27, F1-Score=47.83, AUPRC=81.29, 2: BP: Acc=43.81, F1-Score=32.27, AUPRC=34.23, MF: Acc=48.75, F1-Score=37.60, AUPRC=43.31, CC: Acc=52.82, F1-Score=36.24, AUPRC=47.93**
		Wang et al., 2022 (186)	1. Gligorijevic et al. Dataset (MF, CC, BP), 2. Fluorescence stability Dataset, 3. Protease stability Dataset	BERT	GVP	1: GO-CC: AUROC=0.430 ± 0.010, F1-score=0.525 ± 0.003, GO-BP: AUROC=0.300 ± 0.006, F1-score=0.415 ± 0.005, GO-MF: AUROC=0.577 ± 0.004, F1-score=0.547 ± 0.002, Fluorescence: SRCC=0.680 ± 0.003, Protease Stability: SRCC=0.730 ± 0.005
		Kabir et al., 2022 (205)	1. TDNK Dataset (BP, CC, MF), 2. RS Dataset (BP, CC, MF), 3. TSNK Dataset (BP, CC, MF)	ESM-1 + Transformer	–	1: BP: F1-score(Validation=0.591, Test=0.389), AUPRC(Validation=0.623, Test=0.338), CC: F1-score(Validation=0.750, Test=0.537), AUPRC(Validation=0.782, Test=0.506), MF: F1-score(Validation=0.624, Test=0.528), AUPRC(Validation=0.643, Test=0.468), 2: BP: F1-score(Validation=0.589, Test=0.577), AUPRC(Validation=0.629, Test=0.627), CC: F1-score(Validation=0.736, Test=0.751), AUPRC(Validation=0.751, Test=0.765), MF: F1-score(Validation=0.607, Test=0.614), AUPRC(Validation=0.617, Test=0.619), 3: BP: F1-score(Validation=0.526, Test=0.557), AUPRC(Validation=0.526, Test=0.557), CC: F1-score(Validation=0.739, Test=0.729), AUPRC(Validation=0.724, Test=0.693), MF: F1-score(Validation=0.580, Test=0.623), AUPRC(Validation=0.564, Test=0.584)
		**Zhao et al., 2022 (206)**	**2016 Dataset**	**ESM-1**	**GNN**	**MF: F1-score = 0.598, Smin = 9.670, AUPRC = 0.564, BP: F1-score = 0.478, Smin = 40.229 AUPRC = 0.436, CC: F1-score = 0.709, Smin = 9.558 AUPRC = 0.744**
		**Hu et al., 2022 (207)**	**Hu et al. Dataset**	**AlphaFold**	–	**Metal Ion Binding (MIB) Acc = 0.794, Antibiotic Resistance (ABR) Acc = 0.979, Fluorescence SRCC= 0.67, Stability SRCC= 0.81**
		**Li et al., 2022 (300)**	**Mouse Dataset**	**Word2Vec + Node2Vec**	**RF**	**Exact match: 0.182, Acc: 0.542**
		Tseng et al., 2021 (301)	Tseng et al. Dataset (CC, MF, BP)	ELMo	MLP	BP: F1-score=0.8019, CC: F1-score=0.7066, MF: F1-score=0.8832
		Sharma et al., 2021 (302)	CORUM Dataset (BP, MF, CC)	FastText	RF	BP: AUROC = 0.895, MF: AUROC = 0.927, CC: AUROC = 0.957
		**Zhang et al., 2020 (303)**	**Zhang et al. Dataset (BP, CC, MF)**	**Word2Vec**	**BiLSTM + MCNN**	**BP: F1-score=0.369, Average Precision=0.376, Average Recall=0.366, MCC=0.373, AUROC=0.904, CC: F1-score=0.538, Average Precision=0.582, Average Recall=0.496, MCC=0.502, AUROC=0.953, MF: F1-score=0.570, Average Precision=0.637, Average Recall=0.521, MCC=0.465, AUROC=0.954**
		**Wang et al., 2019 (304)**	**UniProtKB/SwissProt**	**Mashup + Node2Vec**	**SVM**	**F1-score=0.497**
		**Sarker et al., 2019 (305)**	**NEW Dataset**	**FastText**	**MLP**	**Acc: 94.3%**
Multi-class Classification	Structure Prediction	Chen et al., 2024 (150)	1. CAMEO, 2. CASP15, 3. Chen et al. Dataset, 4. CASP12 + CASP 14 Dataset	ESM-2	–	1: TM-score = 0.86, 2: TM-score = 0.70, 3: RMSD=0.9823 ± 0.007, TM-score=0.961 ± 0.001, 4: 3-cls ACC = 75.33
		**Xu et al., 2023 (60)**	**1. Cuff et al. TS115 Q8 Dataset, 2. Cuff et al. CASP12 Q8 Dataset**	**ESM-2**	–	**1: Q8 Acc = 0.7291, 2: Q8 Acc = 0.7902**
		Elnaggar et al., 2023 (27)	1. Cuff et al. CASP12 Q3 Dataset, 2. Cuff et al. TS115 Q3 Dataset, 3. Cuff et al. CB513 Dataset, 4. Cuff et al. CASP14 Q8 Dataset	Transformer-XL, XLNet, BERT, ALBERT, ELECTRA, T5	–	1: Q3 Acc=83.8+3, 2: Q3 Acc=88.2+1, 3: Q8 Acc=77.4+1, Q3 Acc=88.6+0.6, 4: Q8 Acc=63.2+3
		**Heinzinger et al., 2023 (208)**	1. **Cuff et al. CASP14 Q3 Dataset, 2. Elnaggar et al. NEW364 Q3 Dataset**	**T5**	**CNN**	**1: Q3 Acc = 89.4, 2: Q3 Acc = 82.2**
		**Feng et al., 2022 (209)**	**1. Feng et al. Strict_Data (Unbalanced, Balanced), 2. Feng et al. NonStrict_Data (Unbalanced, Balanced)**	**BERT**	**CNN**	**1: Unbalanced Strict_Data: Sn=0.30, Sp=0.99, MCC=0.44, Acc=0.980, Balanced Strict_Data: Sn=0.661, Sp=0.838, MCC=0.198, Acc=0.834, AUROC=0.826, 2: Unbalanced NonStrict_Data: Sn=0.30, Sp=0.99, MCC=0.43, Acc=0.966, Balanced NonStrict_Data: Sn=0.559, Sp=0.833, MCC=0.219, Acc=0.824, AUROC=0.826**
		Lin et al., 2022 (210)	Lin et al. CASP14 Dataset	ESM-2	–	CASP14: TM-score=67.8
		**Weissenow et al., 2022 (211)**	**SetTst29**	**T5**	**CNN**	**TM-score=0.50 ± 0.06**
		**Brandes et al., 2022 (188)**	**NetSurfP Q3 Dataset**	**BERT**	–	**Q3 Acc=0.74**
		Xiao et al., 2021 (154)	Cuff et al. CB513 Dataset	BERT	–	CB513: Q3 Acc = 0.79, Q8 Acc = 0.654
		Elnaggar et al., 2021 (212)	1. Cuff et al. CASP12 Q3 Dataset, 2. Cuff et al. TS115 Q8 Dataset, 3. Cuff et al. CB513 Q8 Dataset, 4. Elnaggar et al. NEW364 Q3 Dataset	T5	CNN	CASP12: Q3 Acc=70.5, TS115: Q8 Acc=77.1, CB513: Q8 Acc=74.5, NEW364: Q3 Acc=74.5
		Rives et al., 2020 (213)	1. Cuff et al. CB513 Q8 Dataset, 2. Cuff et al. CASP13 Q8 Dataset	Transformer	–	CB513: Q8 Acc=71.6 ± 0.1, CASP13: Q8 Acc=72.5 ± 0.2
		Rao et al., 2019 (155)	1. Cuff et al. CB513 Dataset, 2. Cuff et al. CASP12 Dataset, 3. Cuff et al. TS115 Dataset	Transformer	–	CB513: Q3 Acc=0.8, Q8 Acc = 0.63, CASP12: Q3 Acc = 0.76, Q8 Acc = 0.61, TS115: Q3 Acc = 0.81, Q8 Acc = 0.68
Multi-class Classification	Fold Prediction	**Chen et al., 2024 (150)**	**Hou et al. Dataset**	**ESM-2**	–	**Acc=75.61**
		Elnaggar et al., 2023 (27)	Hou et al. Dataset	T5	–	Acc=61.1
		Morcillo et al., 2022 (214)	1. LINDAHL Dataset, 2. LINDAHL_1.75 Dataset, 3. SCOP_2.06 Dataset	T5	–	1: Family: Acc=94.6, SuperFamily: Acc=90.8, Fold: Acc=93.1, 2: Full set: Acc=97.5, Family: Acc=99.3, SuperFamily: Acc=97.6, Fold: Acc=81.4, 3: Full set: 99.6, Family: Acc=99.6, SuperFamily: Acc=99.9, Fold: Acc=86.5
Multi-class Classification	Remote Homology Detection	**Nallapareddy et al., 2023 (215)**	**1. Top 1773 Superfamilies Dataset, 2. Top 50 Superfamilies Dataset**	**T5**	**LogR**	**1: Acc=85.6+0.4, F1-score=72.4+0.7, 2: Acc=98.2+0.3, F1-score=95.5+0.9**
		**Heinzinger et al., 2023 (216)**	**Foldseek Benchmark Dataset**	**T5**	**CNN**	**SF: AUROC=0.45**
		Routray et al., 2022 (350)	1. Routray et al. Dataset 1, 2. Routray et al. Dataset 2, 3. Routray et al. Dataset 3, 4. Routray et al. Dataset 4	CNN+ GRU	MLP	1: Routray et al. Dataset 1: AUROC=0.98, AUROC50=0.96, 2: Routray et al. Dataset 2: AUROC=99.81, AUROC50=99.55, 3: Routray et al. Dataset 3: AUROC=97.74, AUROC50=97.53, 4: Routray et al. Dataset 4: AUROC=98.79, AUROC50=98.88
		**An et al., 2022 (136)**	**Hou et al. Dataset**	**BERT**	**BiLSTM**	**Acc=0.42**
		Brandes et al., 2022 (188)	Hou et al. Dataset	BERT	–	Acc=0.22
		Rives et al., 2021 (213)	Rives et al. Dataset	Transformer	–	Fold: AUROC=0.770, SF: AUROC=0.880
		Xiao et al., 2021 (154)	Hou et al. Dataset	BERT	–	Acc=0.30
Binary Classification	TRP channels Classification	**Shah et al., 2023 (217)**	**Shah et al. Dataset**	**AlphaFold + BERT**	**SVM**	**Cross-Validation Sn=87**, **Sp=93.61**, **Acc=93.39**, **MCC=0.52**, **Independent Test Sn=100**, **Sp=95.54**, **Acc=95.73**, **MCC=0.69**

For this goal, T5 is the most commonly used followed by BERT and ESM-2. Specifically, T5 is used with a self-classifier for protein function identification (195, 203), and fold prediction (27, 214). Moreover, potential of T5 is explored with GCN and LogR classifiers for protein function identification (199) and remote homology detection (215). Similarly, T5 is used with CNN classifier for structure prediction (208, 211, 212) and remote homology detection (216). Among all T5 based predictive pipelines, T5 with CNN classifier has achieved state-of-the-art performance for remote homology detection (216). In addition, combined potential of T5 and ESM-2 is explored with GNN classifier for protein function identification (192). Furthermore, BERT is used with a self-classifier for structure prediction (154, 188) and remote homology detection (154, 188). Moreover, potential of BERT is also explored with LogR and GVP classifiers for protein function identification (186, 202) whereas, BERT is also employed with CNN and BiLSTM classifiers for structure prediction (209) and remote homology detection (136), respectively. In addition, combined potential of T5 and BERT representation with self-classifier is explored for protein function identification (201). Beyond BERT and T5 language models, ESM-2 is employed with self-classifier for protein function identification (150, 190, 193, 194, 200), structure prediction (60, 150, 210), and fold prediction (150) whereas, potential of ESM-2 is explored with RF classifier for protein function identification (197). Moreover, combined potential of T5, BERT, ESM-2, and OHE is explored with DNN classifier for protein function identification (196). In addition, potential of ESM-1 is explored with GAN, GCN and GNN classifiers for protein function identification (198, 204, 206). Apart from this, transformer based representation learning is used with a self-classifier for protein function identification (185), structure prediction (155, 213) and remote homology detection (213). Besides these, combined potential of Transformer-XL, XLNet, BERT, ALBERT, ELECTRA and T5 is explored with a self-classifier for structure prediction (27). Among ESM-1, ESM-2 and transformer based representation approaches, ESM-2 with a self classifier has achieved state of the art performance for all three tasks protein function identification (150), structure prediction (150) and fold prediction (150). Besides this, combined potential of transformer and ESM-1 representation is used with CNN and self-classifier for protein function identification (191, 205). Moreover, AlphaFold is employed with a self-classifier for protein function identification (207). whereas combined potential of AlphaFold and BERT is explored with SVM classifier for TRP channels classification (217) and has achieved state-of-the-art performance.

Furthermore, FastText representation with RF and MLP classifiers while ELMo representation is used with MLP and hybrid (SVM+LogR) classifiers are employed for protein function identification (297, 298, 301, 302, 305). Moreover, Word2Vec is employed with hybrid (BiLSTM+MCNN) classifier for protein function identification (303). Apart from this, combined potential of Word2Vec and Node2Vec with RF classifier is also explored for protein function identification (300). Beyond these representation learning approaches, Mashup is employed with SVM classifier for protein function identification (299). Similarly, combined potential of Mashup and Node2Vec is also explored with SVM classifier, KG representation is used with ANN classifier for protein function identification (304, 349). Besides these, combined representation from CNN and GRU is used with MLP classifier for remote homology detection (350).

A comprehensive analysis of existing studies for this goal indicates that there is a significant room for improvement in fold prediction, remote homology detection and TRP channels classification. By observing performance trends for this goal, potential of shallow neural network based word embedding such as Word2Vec and FastText with graph based deep learning classifiers such as GNN can raise the performance of under-performing tasks.

Table 15 summarizes the performance of 13 predictive pipelines for protein sub-cellular localization prediction goal. This goal predictive pipelines have used 10 unique representation learning approaches namely, ESM-2 (23), amino acid properties based representation approaches (25), OHE (26), T5 (27, 28), Node2Vec (30, 31), Word2Vec (32), ELMo (33, 306), amino acid properties and composition based representation approaches (351), BERT (218, 219), and PSSM (219). In addition, nine unique classifiers are identified including GAT (25), CNN (26, 33, 219, 306), LSTM (30, 31), SVM (32, 351), BiLSTM (306) and self-classifier of BERT (218), Transformer (219), ESM-2 (23) and T5 (27, 28).

**Table 15.** Protein sub-cellular localization prediction related three distinct protein sequence analysis tasks predictive pipelines performance

Task Type	Task Name	Author, Year [ref]	Dataset	Representation learning	Classifier	Performance Evaluation
Multi-label, Multi-class Classification	Protein Subcellular Localization Identification	**Luo et al., 2024 (23)**	**Luo et al. Swis-Prot Datasets** (**Cell junction**, **Cell membrane**, **Cell projection**, **Cytoplasm**, **Golgi apparatus: Lysosome**, **Mitochondrion**, **Nucleus**, **Secreted**) **Luo et al. TrEMBL Datasets** (**Cell junction**, **Cell membrane**, **Cell projection**, **Cytoplasm**, **Golgi apparatus**, **Lysosome**, **Mitochondrion**, **Nucleus**, **Secreted**, **Endoplasmic reticulum**)	**ESM-2**	**_**	**Cell junction: MCC=0.377 Cell membrane: MCC=0.780 Cell projection: MCC=0.069 Cytoplasm: MCC=0.558 Golgi apparatus: MCC=0.605 Lysosome: MCC=0.380 Mitochondrion: MCC=0.878 Nucleus: MCC= 0.730 Secreted: MCC=0.866 Cell junction: MCC=0.06** ± **0.14 Cell membrane: MCC=0.62** ± **0.04 Cell projection: MCC=0.21** ± **0.07 Cytoplasm: MCC=0.40** ± **0.03 Golgi apparatus: MCC=0.65** ± **0.03 Lysosome: MCC=0.51** ± **0.06 Mitochondrion: MCC=0.68** ± **0.03 Nucleus: MCC=0.73** ± **0.04 Secreted: MCC=0.61** ± **0.02 Endoplasmic reticulum: MCC=0.69** ± **0.04**
		**Wang et al., 2024 (25)**	**1. Gram-Positive bacteria Dataset 2. Gram-Negative bacteria Dataset 3. Viral Dataset 4. Plant Dataset 5. Human Dataset 6. SARS-CoV-2 Dataset**	**CT + DC + PsePSSM + DDE + EBGW + CTD (amino acid properties based representation approaches)**	**GAT**	**Average Acc=0.9895, Average Precision=0.9873, F1-score=0.9933, Hamming Loss=0.0045, Ranking Loss=0.0082, One Error=0.0105 Average Acc=0.9582, Average Precision=0.9539, F1-score=0.9497, Hamming Loss=0.0107, Ranking Loss=0.0463, One Error=0.0617 Average Acc=0.9470, Average Precision=0.9889, F1-score=0.9762, Hamming Loss=0.0145, Ranking Loss=0.0179, One Error=0.0538 Average Acc=0.9371, Average Precision=0.9780, F1-score=0.9660, Hamming Loss=0.0239, Ranking Loss=0.0329, One Error=0.0440 Average Acc=0.907, Average Precision=0.9177, F1-score=0.9371, Hamming Loss=0.0387, Ranking Loss=0.0594, One Error=0.0742 Average Acc=78.76**
		**Gillani et al., 2024 (26)**	**Gillani et al. Datasets (Other, Membrane, Cytoplasm, Golgi Apparatus, Mitochondrion, Nucleus, Plastid, Secreted)**	**OHE**	**N-to-1-CNN**	**Other: MCC=0.10, Acc=26.74, Sp=86.99, Sn=26.74, F1-score=17.89 Membrane: MCC=0.72, Acc=71.67, Sp=96.02, Sn=71.67, F1-score=77.89 Cytoplasm: MCC=0.40, Acc=44.21, Sp=93.34, Sn=44.21, F1-score=47.59 Golgi Apparatus: MCC=0.19, Acc=1.35, Sp=92.31, Sn=26.67, F1-score=27.00 Mitochondrion: MCC=0.48, Acc=53.33, Sp=97.43, Sn=53.33, F1-score=50.45 Nucleus: MCC=0.58, Acc=55.68, Sp=95.66, Sn=55.68, F1-score=65.19 Plastid: MCC=0.50, Acc=57.50, Sp=97.12, Sn=57.50, F1-score=52.47 Secreted: MCC=0.80, Acc=78.98, Sp=97.53, Sn=78.98, F1-score=84.05**
		**Elnaggar et al., 2023 (27)**	**Armenteros et al. Dataset**	**T5**	–	**Acc=83.2 ± 2**
		**Thumuluri et al., 2022 (28)**	**1. Swiss-Prot CV Dataset 2. HPA Independent Dataset**	**T5**	–	**Swiss-Prot CV Dataset Acc=0.55+0.02, Jaccard=0.69+0.01, Micro F1-score=0.73+0.01, Macro F1-score=0.66+0.01 HPA Independent Test Acc=0.39, Jaccard=0.53, Micro F1-score=0.60, Macro F1-score=0.46**
		**Pan et al., 2022 (30)**	**Pan et al. Yeast Dataset**	**Node2Vec**	**LSTM**	**Acc=0.795, MCC=0.741**
		**Pan et al., 2019 (31)**	**Pan et al. Human Dataset**	**Node2Vec**	**LSTM**	**Acc=0.843, MCC=0.812**
		Asgari et al., 2019 (32)	Subcellular location Dataset	Word2Vec	SVM	Macro Precision=0.68, Macro Recall=0.60, Macro F1-score=0.62
		Michael Heinzinge, 2019 (33)	Armenteros et al. Dataset	ELMo	CNN	Localization: Acc=68 ± 1, MCC=0.61 ± 0.01 membrane/globular: Acc=86.8 ± 1.0, MCC=0.725 ± 0.021
Multi-class Classification	Protein Submitochondrial Localization Identification	**Ruan et al., 2024 (351)**	**1. M317 Dataset 2. M983 Dataset 3. M495 Dataset 4. M1217 Dataset**	**RS + DDE + P-PSSM-EnCom + Im-Psepssm + PseAAC + AD (amino acid properties and composition based representation approaches)**	**SVM**	**Average F1-score=98.77, Average MCC=98.15, Acc=98.7 Average F1-score=98.89, Average MCC=98.36, Acc=98.9 Average F1-score=96.05, Average MCC=93.93, Acc=95.8 Average F1-score=90.22, Average MCC=87.37, Acc=90.1**
		**Wang et al., 2023 (218)**	**1. Savojardo et al. SM424 Dataset 2. Kumar et al. SM570 Dataset Wang et al. Datasets (3. Human.Mitocarta3.0, 4. Mouse.Mitocarta3.0)**	**BERT**	–	**Inner membrane: MCC=0.92, Inter membrane space: MCC=0.87, Matrix: MCC=0.94, Outer membrane: MCC=0.96, GCC=0.92 Inner membrane: MCC=0.87, Inter membrane space: MCC=0.77, Matrix: MCC=0.91, Outer membrane: MCC==0.95, GCC=0.88 Inner membrane: MCC=0.80, Inter membrane space: MCC=0.54, Matrix: MCC=0.82, Outer membrane: MCC=0.77, GCC=0.73 Inner membrane: MCC=0.78, Inter membrane space: MCC=0.62, Matrix: MCC=0.82, Outer membrane: MCC=0.71, GCC=0.73**
		Hou et al., 2021 (306)	Hou et al. Datasets (1. M187, 2. Human.MitoCarta3.0, 3. Mouse.Mitocarta3.0)	ELMo	CNN + BiLSTM	Matrix: MCC=0.5799, Inter membrane: MCC=0.7864, Inner membrane: MCC=0.5280, Outer membrane: MCC=0.7012, GCC=0.6829 Matrix: MCC=0.5617, Inter membrane: MCC=0.3124, Inner membrane: MCC=0.4746, Outer membrane: MCC=0.5052, GCC=0.4623 Matrix: MCC=0.5969, Inter membrane: MCC=0.3949, Inner membrane: MCC=0.4974, Outer membrane: MCC=0.5570, GCC=0.5151
Multi-label Classification	Subchloroplast Localization Identification	**Wang et al., 2023 (219)**	**1. MSchlo578 Dataset 2. Novel Dataset**	**BERT, PSSM**	**CNN, Transformer**	**Acc=0.943, Precision=0.951, Recall=0.943, F1-score=0.945, Grand Mean=0.923 Acc=0.862, Precision=0.877, Recall=0.86, F1-score=0.864, Grand Mean=0.842**

Among all representation learning approaches, Node2Vec is used with LSTM classifier for protein subcellular localization identification (30, 31), and ELMo is employed with CNN and hybrid (CNN + BiLSTM) classifiers for protein subcellular localization identification (33, 306). In addition, potential of T5 representation is also explored with a self-classifier for protein subcellular localization identification (27, 28). Whereas, BERT with its a self-classifier is used for protein submitochondrial localization identification (218) and combined potential of BERT and PSSM is explored with CNN and Transformer self-classifier for subchloroplast localization identification (219). Among all four representation approaches, BERT has achieved state-of-the-art performance for subchloroplast localization identification (219). Furthermore, Word2Vec with SVM classifier and OHE with CNN classifier are employed for protein subcellular localization identification (26, 32). In addition, potential of ESM-2 representation is explored with self-classifier for protein subcellular localization identification (23) and has achieved state-of-the-art performance. Apart from word embedding and LLMs based predictive pipeline, amino acid properties based representation approaches are used with GAT classifier for protein subcellular localization identification (25). Whereas, amino acid properties and composition based representation approaches are employed with SVM classifier for protein submitochondrial localization identification (351). Among both, amino acid properties and composition based representation approaches with SVM classifier manages to achieve top performing values.

From all task of this goal, subchloroplast localization identification offers significant room for improvement. Analysing the performance trends of various tasks in this goal, amino acid properties and composition based representation approaches with machine learning classifiers such as SVM and GAT classifiers can improve the performance of under-performing task.

Table 16 provides a high level overview of 11 predictive pipelines related to two goals namely mutation analysis and disease analysis.

**Table 16.** Mutation and disease analysis related nine distinct protein sequence analysis tasks predictive pipelines performance

Task Type	Task Name	Author, Year [ref]	Dataset	Representation learning	Classifier	Performance Evaluation
**Goal: Mutation Analysis**						
Multi-class Classification	Mutation Prediction	**Tzavella et al., 2023 (220)**	**Tzavella et al. Dataset (TP53, BRAF, AR, CHEK2, PTEN)**	**T5**	**GMM**	**Gene TP53: Acc=0.85, Weighted Acc=0.915 BRAF: Acc=0.89, Weighted Acc=0.969 AR: Acc=0.862, Weighted Acc=0.935 CHEK2: Acc=0.90, Weighted Acc=0.942 PTEN: Acc=0.817, Weighted Acc=0.961**
Multi-class Classification	Mutation Effects Prediction	**Wang et al., 2024 (221)**	**Yang et al. Dataset**	**AlphaFold**	**GCN**	**Cross-Validation Acc=0.654, GCC=0.289 Independent Test Acc=0.618, GCC=0.242**
		Meier et al., 2021 (222)	Riesselman et al. Dataset	ESM-1	_	zero shot: Full: SRCC=0.509 Test: SRCC=0.482 +further Train Full: SRCC=0.538 Test: SRCC=0.519
		**Strokach et al., 2021 (223)**	**Strokach et al. Dataset**	**BERT, GNN**	**GBDT**	**EL2interface: SRCC=0.62**
Binary Classification	Variant Effects Prediction	Marquet et al., 2021 (224)	Marquet et al. Datasets (1. PMD4k 2. DMS4 )	T5	LR	1. Effect: F1-score=55.93 ± 1.23, Neutral: F1-score=80.11 ± 0.64, Q2=72.59 ± 0.72, MCC=0.405 ± 0.016 2. Effect: F1-score=81.49 ± 0.15, Neutral: F1-score=38.24 ± 0.4, Q2=71.51 ± 0.39, MCC=0.206 ± 0.010
**Goal: Disease Analysis**						
Binary Classification	Malaria Parasite Identification	Hayat et al., 2022 (307)	Verma et al. Dataset	FastText	Ensemble (RF+PNN+SVM + KNN)	Acc=97.81, Sn=97.51, Sp=98.10, MCC=0.95
Binary Classification	Tumour Necrosis Factors Identification	Nguyen et al., 2020 (308)	Nguyen et al. Dataset	FastText	SVM	Acc=95.82 ± 1.67, Sp=97.59 ± 2.15, Sn=83.67 ± 7.45, MCC=0.83 ± 0.06
Binary Classification	COVID-19 Virus Classification	Adjuik et al., 2022 (309)	Adjuik’s et al. Dataset	Word2Vec	RF	Train Acc=0.990, Test Acc=0.995
Binary Classification	Vascular Calcification	**Chao et al., 2022 (309)**	**Chao et al. Dataset**	**Node2Vec, GNN**	**RF**	**F1-score=0.724**
Binary Classification	B/T Cell Receptor Sequences Analysis	**Ostrovsky et al., 2021 (311)**	**1. DS1 2. DS2 3. DS3**	**Word2Vec**	**LogR**	**DS1 F1-score = 0.67, DS2 F1-score = 0.51, DS3 F1-score = 0.69**
Binary Classification	B-Cell Epitopes Identification	**Zeng et al., 2023 (225)**	**Zeng et al. Dataset**	**ESM-2 + AlphaFold**	**BiLSTM, GNN**	**AUROC=0.751, AUPRC=0.261, F1-score=0.310, MCC=0.232, Recall=0.393, Precision=0.255**

For mutation analysis goal, five predictive pipelines for three protein sequence analysis tasks have used five unique representation namely T5, AlphaFold, ESM-1, GNN and BERT. Moreover, these predictive pipelines have utilized five different classifiers namely GBDT, LR, GCN, GMM and MLP. T5 emerges as the most frequently used representation learning approach for this goal. Specifically, T5 is used with LR and GMM classifiers for variant effect prediction (224) and mutation prediction (220), respectively. Among both, T5 with GMM classifier has achieved state-of-the-art performance for mutation prediction (220). Furthermore, AlphaFold is used with GCN classifier, combined representation from ESM-1 and MSA transformer is employed with self-classifier and combined potential of BERT and GNN is also explored with GBDT classifier for mutation effects prediction (221–223). An in-depth analysis of these predictive pipelines indicates that there is a significant room for improvement in mutation effects prediction. Taking into account the performance trends across different goals, potential of shallow neural network based word embeddings such as Word2Vec, Node2Vec and FastText can be used with deep learning classifiers such as BiLSTM and CNN to raise the performance of under-performing task.

For disease analysis goal, six predictive pipelines for different tasks have employed six unique representation learning approaches namely FastText, Word2Vec, Node2Vec, AlphaFold, GNN and ESM-2. Overall, these predictive pipelines have utilized seven different classifiers including SVM, RF, PNN, KNN, BiLSTM, GNN and LogR.. Among all representation learning approaches, FastText and Word2Vec are most commonly used for this goal. FastText is explored with SVM and ensemble $(RF+SVM+PNN+KNN)$ classifiers for tumour necrosis factors identification (308) and malaria parasite identification (307), respectively. Moreover, Word2Vec is used with RF classifier for COVID-19 virus classification (309) and $B/T$ Cell receptor sequences analysis (311). Furthermore, combined potential of Node2Vec and GNN is used with RF classifier for vascular calcification (310). Beyond word embedding based representation learning approaches, combined potential of ESM-2+AlphaFold representation is also explored with BiLSTM and GNN for B-Cell epitopes identification (225). From all these tasks, vascular calcification, B-Cell epitopes identification and $B/T$ Cell receptor sequences analysis indicate a significant room for improvement. By analysing the performance patterns across different goals, potential of amino acid composition approaches and BERT with deep learning classifiers such as BiLSTM and LightGBM can raise the performance of under-performing tasks.

To sum it up, a comprehensive analysis of advanced predictive pipelines based on word embeddings, language models, and domain-specific representation learning methods reveals intriguing trends. Among 63 protein sequence analysis tasks classified into 11 main biological goals, 34 tasks involve binary classification, nine involve interaction prediction, nine involve multi-class classification, two involve multi-label classification, and seven involve regression. Two protein sequence analysis tasks belong to more than one task type such as gene phenotype prediction and protein subcellular localization prediction involve multi-class and multi-label classification applications. In total, 84 distinct representation learning methods and 67 predictive algorithms are employed to develop robust predictive pipelines for these tasks. Language model-based representation learning strategies and deep learning classifiers consistently achieve superior performance across the majority of tasks within these 11 biological goals. Researchers are encouraged to investigate capabilities of cutting-edge transformer based language models such as hierarchical and heterogeneous Graph transformers, GPT-4, and hybrid representation learning approaches. Furthermore, integrating these models with advanced ensemble machine learning or deep learning classifiers may enhance performance for various classification, regression, and clustering tasks.

## Publisher and journal-wise distribution of research articles

This section provides comprehensive overview of publication venue distribution for 295 protein sequence analysis studies across different publishers, journals, and conferences. Selection of suitable publication venue for interdisciplinary AI-driven protein sequence analysis research is important. There exists three primary categories of publication venues namely (1) Core AI based publication venues emphasizes mathematical foundations and technical advancements in AI algorithms, (2) Biological publication venues focuses on biological significance and novelty of presented research findings, (3) Hybrid publication venues bridges the gap by integrating both AI and biological approaches. Researchers often face desk rejections when targeting core AI or biology venues due to their narrow disciplinary focus. This analysis emphasizes strategic selection of hybrid publication venues catering to interdisciplinary nature of research. Although various tools have been developed for venues identification, but this study provides in-depth analysis to target resource for researchers by identifying diverse venues which have published applications of word embeddings and LLMs for protein sequence analysis.

Table 17 presents comprehensive overview of 295 protein sequence analysis studies disseminated across various academic platforms including 87 journals, 11 conferences, six transactions, and three pre-print repositories. Within journals, highest publication frequency is observed in Briefings in Bioinformatics, followed by Bioinformatics, Computers in Biology and Medicine, and BMC Bioinformatics. Among 11 conferences, specifically IEEE International Conference on Bioinformatics and Biomedicine (BIBM) leads in publications and have published 11 articles whereas each of remaining conferences have only published one article. These conferences include IEEE International Conference on Bioinformatics and Biomedicine (BIBM), IEEE Asia-Pacific Conference on Computer Science and Data Engineering (CSDE), Asia Conference on Advanced Robotics, Automation, and Control Engineering (ARACE), International Joint Conference on Neural Networks (IJCNN), IEEE International Conference on Tools with Artificial Intelligence (ICTAI), Annual International Conference of the IEEE Engineering in Medicine & Biology Society (EMBC), International Conference on Electrical Engineering and Information & Communication Technology (ICEEICT), and Bioinformatics Research and Applications International Symposium (ISBRA). Among all transactions publications, ACM Transactions on Computational Biology and Bioinformatics is predominant, followed by IEEE Transactions on Emerging Topics in Computational Intelligence, IEEE Transactions on Neural Networks and Learning Systems, IEEE Transactions on NanoBioscience, IEEE Transactions on Pattern Analysis and Machine Intelligence, and IEEE Transactions on Computational Social Systems. Taking into account the fast-paced nature of research, researchers have also considered rapid dissemination platforms and published 40 studies on these platforms namely BioRxiv, medRxiv, and arXiv.

**Table 17.** Publication distribution of protein sequence analysis literature across diverse journals and conferences

Journal Name	Papers Count	Journal Name	Papers Count	Journal Name	Papers Count	Journal Name	Papers Count	Journal Name	Papers Count	Journal Name	Papers Count	Journal Name	Papers Count	Conference	Papers Count
Briefings in Bioinformatics	26	Molecular Therapy- Nucleic Acids	1	BioMed Research International	1	International Journal of Computational Intelligence Systems	1	Nature Communications	1	Axioms	1	Frontiers in Oncology	1	BIBM	11
Bioinformatics Advances	1	Medicine in Novel Technology and Devices	1	Mathematical Problems in Engineering	1	Frontiers of Computer Science	1	Nature Machine Intelligence	1	International Journal of Molecular Sciences	4	Frontiers in Genetics	7	CSDE	1
Bioinformatics	17	Information Fusion	1	Expert Systems	1	Amino Acids	1	Plos one	4	Biomolecules	3	Frontiers in Genetics	2	ARACE	1
Nucleic Acids Research	3	Neurocomputing	1	Oxidative Medicine and Cellular Longevity	1	Journal of translational medicine	1	PLOS Computational Biology	1	Genes	2	Frontiers in immunology	1	IJCNN	1
NAR Genomics & Bioinformatics	2	Structure	1	BMC bioinformatics	14	BMC systems biology	1	IEEE/ACM Transactions on Computational Biology and Bioinformatics	15	Applied Sciences	1	Journal of Biomolecular Structure and Dynamics	1	ICTAI	1
Journal of Computational Biology	3	Gene	1	Interdisciplinary Sciences: Computational Life Sciences	3	Journal of Cheminformatics	1	IEEE Transactions on Emerging Topics in Computational Intelligence	1	Antibiotics	1	Elife	1	EMBC	1
Computers in Biology & Medicine	17	Analytical Biochemistry	1	BMC genomics	3	BMC biology	1	IEEE Transactions on Neural Networks and Learning Systems	1	bioRxiv	16	Advances in Neural Information Processing Systems	1	ICEEICT	1
Computational & Structural Biotechnology Journal	4	Journal of Computational Science	1	SN Computer Science	1	Human genetics	1	IEEE Transactions on NanoBioscience	1	medRxiv	2	Research	1	CBCBHI	1
Computational Biology & Chemistry	3	Patterns	1	Journal of Shanghai Jiaotong University (Science)	1	BMC Medical Genomics	1	IEEE Transactions on pattern analysis and machine intelligence	1	arXiv	22	PeerJ	1	ICML	1
Computational & Structural Biotechnology Journal	1	Biophysical Chemistry	1	Journal of Cheminformatics	1	International Journal of Information Technology	1	IEEE Transactions on Computational Social Systems	1	Frontiers in Bioengineering and Biotechnology	1	Chinese Journal of Electronics	1	ICKDIR	1
Iscience	2	Knowledge-Based Systems	1	Molecular Diversity	1	Journal of Chemical Information and Modeling	6	IEEE Journal of Biomedical and Health Informatics	five	Frontiers in Bioinformatics	1	Biosafety and Health	1	ICLR	1
Artificial Intelligence in Medicine	2	The Plant Journal	1	BMC Biomedical Engineering	1	Journal of proteome research	1	IEEE Access	1	Frontiers in Physiology	1	_	_	ISBRA	1
Journal of molecular biology	1	Journal of Computational Chemistry	1	Molecular Genetics and Genomics	1	Scientific Reports	9	Life	1	Frontiers in Medicine	1	_	_	AMIA Annual Symposium Proceeding	1
Journal of Algorithms and Computation	1	Duzce Universitesi Bilim ve Teknoloji Dergisi	1	Advances in Neural Information Processing Systems	6	CURRENT SCIENCE	1	Computing and Informatics	1	Proceedings of the National Academy of Sciences	3	_	_	_	_

**Figure 8.**
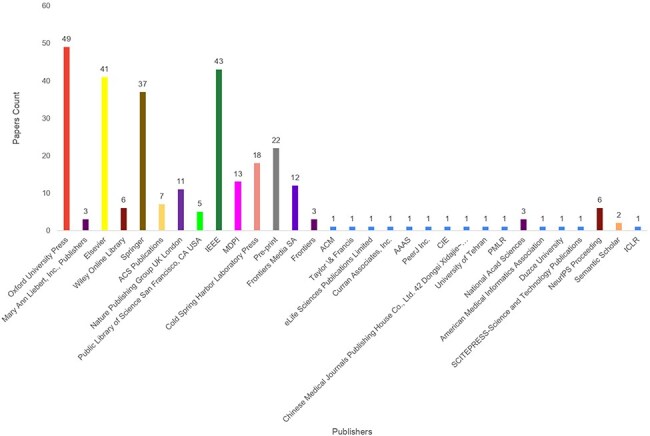
Distribution of publishers involved in the publication of protein sequence analysis literature.

Figure 8 further elaborates distribution of these studies across 31 publishers including Oxford University Press (https://academic.oup.com/), Mary Ann Liebert, Inc. (https://www.liebertpub.com/), Elsevier (https://www.elsevier.com/), Wiley Online Library, Springer (https://www.springer.com/in), ACS Publications (https://pubs.acs.org/), Nature Publishing Group UK London (https://www.iabuk.com/member-directory/nature-publishing-group), Public Library of Science San Francisco, CA USA (https://plos.org/), IEEE (https://www.ieee.org/), MDPI (https://www.mdpi.com/), Cold Spring Harbor Laboratory Press (https://www.cshlpress.com/), Pre- print (https://arxiv.org/), Frontiers Media SA (https://research.monash.edu/en/activities/frontiers-media-sa-publisher), Frontiers (https://www.frontiersin.org/), ACM (https://www.acm.org/publications), Taylor & Francis (https://taylorandfrancis.com/), eLife Sciences Publications Limited (https://elifesciences.org/), Curran Associates Inc. (https://www.proceedings.com/), AAAS (https://www.aaas.org/journals), PeerJ Inc. (https://peerj.com/), CIE (https://cie.co.at/publications), Chinese Medical Journals Publishing House Co. Ltd (https://journals.lww.com/cmj/pages/default.aspx), University of Tehran (https://www.nhbs.com/shop/publisher/university-of-tehran), PMLR (https://proceedings.mlr.press/), National Academy of Sciences (https://www.nationalacademies.org/publications), American Medical Informatics Association (https://amia.org/news-publications/journals), Duzce University (https://doaj.org/toc/2148-2446), SCITEPRESS-Science and Technology Publications (https://www.scitepress.org/HomePage.aspx), NeurIPS Proceedings (https://papers.nips.cc/), Semantic Scholar (https://www.semanticscholar.org/about/publishers), and ICLR (https://iclr.cc/).

It is worth noting that 170 of the 295 studies are published by Oxford University Press, Springer, Elsevier, and IEEE, whereas Nature Publishing Group UK London, MDPI, Cold Spring Harbor Laboratory Press, Pre-print, and Frontiers Media SA have collectively published 76 studies. Remaining 49 studies are published by Mary Ann Liebert, Inc., Wiley Online Library, ACS Publications, Public Library of Science San Francisco, CA USA, Frontiers, ACM, Taylor & Francis, eLife Sciences Publications Limited, Curran Associates Inc., AAAS, PeerJ Inc., CIE, Chinese Medical Journals Publishing House Co., Ltd, University of Tehran, PMLR, National Academy of Sciences, American Medical Informatics Association, Duzce University, SCITEPRESS-Science and Technology Publications, NeurIPS Proceedings, Semantic Scholar, and ICLR. In summary, among 295 protein sequence analysis studies, 213 are journal articles, 22 are conference papers, 20 are transaction papers, and 40 are pre-print studies, published by 31 different publishers. This detailed analysis highlights extensive and diverse research landscape in field of protein sequence analysis and spans multiple publication platforms and venues.

## Discussion

This study sets a stage for AI-driven protein sequence analysis by performing large scale literature of 22 distinct word embeddings methods and 15 LLMs based 295 distinct scientific studies. In total 22 distinct word embeddings and 15 language models based predictive pipelines are designed by developing datasets from 100 distinct databases. Among these databases, 32 databases do not exist anymore while 68 databases are publicly available. These databases are always updated with new sequences information on daily, weekly or monthly bases and can be utilized to develop new datasets. Although for distinct types of tasks many datasets are publicly available but those datasets may have less number of sequences and deep learning models produce better performance when they are trained on large sequences data. However, development of new datasets leads toward inconsistency in predictive pipelines performance comparison. As an example in total 22 distinct word embeddings based predictive pipelines are evaluated on 165 distinct datasets of 32 different protein sequence analysis tasks, while 13 language models based predictive pipelines are evaluated on 328 datasets of 47 different tasks. Both types of predictive pipelines are evaluated only on two common datasets. This analysis reveals that predictive pipelines are not evaluated on same benchmark datasets and their is need of comparative studies that benchmark performance values of these predictive pipelines across same benchmark datasets.

In addition to comparative study, there is a need to standardize dataset utilization to streamline the development of new predictors. Researchers should develop new datasets but they must report their predictors performance on existing datasets as well. An other solution is to benchmark existing predictors performance on newly developed dataset and compare proposed predictor performance with them as well.

However, the majority of researchers do not make their predictors source codes publicly available, which makes challenging to benchmark the predictors performance on newly developed datasets. A comprehensive analysis of AI-driven protein sequence analysis studies reveals that while developing predictive pipelines researchers have primarily focused on two key components: representation learning methods and predictors (classifiers, regressors, similarity computation methods). Within representation learning landscape, 22 distinct word embedding methods and 15 language models have been utilized. At the predictor level, researchers have employed eight different machine learning algorithms and 15 deep learning techniques to build the pipelines. In AI-driven protein sequence analysis predictive pipelines, researchers have predominantly focused on leveraging either word embedding methods or language models. However, the performance of these pipelines could be significantly improved by harnessing the combined strengths of both word embedding techniques and language models simultaneously. Moreover, only a limited number of word embeddings and language models have been investigated for specific tasks. For instance, in protein–protein interaction prediction task, only six word embedding methods and six language models have been explored. Beyond these methods, potential of an additional nine word embedding techniques and five LLMs is investigated within DNA and RNA sequence analysis. However, these word embeddings and language models remain unexplored in predictive pipelines for protein sequence analysis. The unexplored word embeddings are MetaGraph2Vec (454), HAKE (229), HOPE (274, 455), Laplacian eigen maps (456), Locally linear embedding (456), RWR (457), SocDim (458, 459), SVD (460, 461), and Graph2vec (462). Moreover, unexplored language models are BigBird (463), ELECTRA (464, 465), Heterogeneous Graph Transformer (466), LongFormer (463), Transformer-XL (467), and ULMFiT (468, 469). By leveraging additional word embedding methods and LLMs can provide new insights and enhance accuracy in AI-driven protein sequence analysis tasks. One of the primary objectives of this study is to provide a strong foundation for researchers to further investigate the potential of various word embedding methods and language models across tasks where their applicability has yet to be fully explored.

## Declaration of generative AI and AI-assisted technologies in the writing process

Authors have utilized Grammarly to deal with language and grammar issues, and ChatGPT to assist with outlining, understanding various studies, and expanding concepts during preparation of this work. After these tools utilization, authors have reviewed and edited content as necessary, and take full responsibility for final publication.

## References

[1] Xie X-L, Zheng L-F, Yu Y et al. (2012) Protein sequence analysis based on hydropathy profile of amino acids. *Journal of Zhejiang University Science B*, 13, 152–158. doi: 10.1631/jzus.B1100052 22302429 PMC3274743

[2] Edelstein C, Gordon JI, Toscas K et al. (1983) In vitro conversion of proapoprotein A-I to apoprotein A-I. Partial characterization of an extracellular enzyme activity. *Journal of Biological Chemistry*, 258, 11430–11433. doi: 10.1016/S0021-9258(17)44242-6 6311811

[3] Mitra A, Herren CD, Patel IR et al. (2016) Integration of ai-2 based cell-cell signaling with metabolic cues in Escherichia coli. *PLoS One*, 11, e0157532. doi: 10.1371/journal.pone.0157532 PMC4928848 27362507

[4] Murphy BR, Clements ML. The systemic and mucosal immune response of humans to influenza a virus. In *New Strategies for Oral Immunization: International Symposium at the University of Alabama at Birmingham and Molecular Engineering Associates, Inc*. *Birmingham, AL, USA*, *March 21–22*, 1988, 107–116. Springer, 1989. 10.1007/978-3-642-74529-4_12 2659262

[5] Vilhekar RS, Rawekar A Artificial intelligence in genetics. *Cureus*, 16, 2024. doi: 10.7759/cureus.52035 PMC10856672 38344556

[6] Chen H, Zhu Z, Zhu Y et al. (2015) Pathway mapping and development of disease-specific biomarkers: protein-based network biomarkers. *Journal of Cellular and Molecular medicine*, 19, 297–314. doi: 10.1111/jcmm.12447 25560835 PMC4407592

[7] Laub V, Devraj K, Elias L, et al. (2023) Bioinformatics for wet-lab scientists: practical application in sequencing analysis. *BMC genomics*, 24, 382. doi: 10.1186/s12864-023-09454-7 PMC10326960 37420172

[8] Satam H, Joshi K, Mangrolia U, et al. (2023) Next-generation sequencing technology: current trends and advancements. *Biology*, 12, 997. doi: 10.3390/biology12070997 PMC10376292 37508427

[9] Krishnaji Kulkarni C (2021) *Automating the Experimental Laboratory.* The Ohio State University.

[10] Yongjun X, Liu X, Cao X, et al. Artificial intelligence: a powerful paradigm for scientific research. *The Innovation*, 2, 2021. doi: 10.1016/j.xinn.2021.100179 PMC8633405 34877560

[11] Mardikoraem M, Wang Z, Pascual N, et al. (2023) Generative models for protein sequence modeling: recent advances and future directions. *Briefings in Bioinformatics*, 24, bbad358. doi: 10.1093/bib/bbad358 PMC10589401 37864295

[12] Hou X, Wang Y, Bu D et al. (2023) Emngly: predicting n-linked glycosylation sites using the language models for feature extraction. *Bioinformatics*, 39, btad650. doi: 10.1093/bioinformatics/btad650 PMC10627407 37930896

[13] Alkuhlani A, Gad W, Roushdy M et al. (2022) Ptg-plm: predicting post-translational glycosylation and glycation sites using protein language models and deep learning. *Axioms*, 11, 469. doi: 10.3390/axioms11090469

[14] Pratyush P, Pokharel S, Saigo H, et al. (2023) plmsnosite: an ensemble-based approach for predicting protein s-nitrosylation sites by integrating supervised word embedding and embedding from pre-trained protein language model. *BMC bioinformatics*, 24, 41. doi: 10.1186/s12859-023-05164-9 PMC9909867 36755242

[15] Ziyang X, Zhong H, Bingrui H et al. (2024) Ptransips: identification of phosphorylation sites enhanced by protein plm embeddings. *IEEE Journal of Biomedical and Health Informatics*. 10.1109/JBHI.2024.3377362 38483806

[16] Song T, Yang Q, Qu P et al. (2024) Attenphos: general phosphorylation site prediction model based on attention mechanism. *International Journal of Molecular Sciences*, 25, 1526. doi: 10.3390/ijms25031526 PMC10855885 38338804

[17] Pakhrin SC, Pokharel S, Pratyush P et al. (2023) Lmphossite: a deep learning-based approach for general protein phosphorylation site prediction using embeddings from the local window sequence and pretrained protein language model. *Journal of Proteome research*, 22, 2548–2557. doi: 10.1021/acs.jproteome.2c00667 37459437

[18] Wang X, Zhang Z, Zhang C et al. (2022) Transphos: a deep-learning model for general phosphorylation site prediction based on transformer-encoder architecture. *International Journal of Molecular Sciences*, 23, 4263. doi: 10.3390/ijms23084263 PMC9029334 35457080

[19] Shrestha P, Kandel J, Tayara H, et al. (2024) Dl-sphos: prediction of serine phosphorylation sites using transformer language model. *Computers in Biology and Medicine*, 169, 107925. doi: 10.1016/j.compbiomed.2024.107925 38183701

[20] Pokharel S, Pratyush P, Heinzinger M et al. (2022) Improving protein succinylation sites prediction using embeddings from protein language model. *Scientific reports*, 12, 16933. doi: 10.1038/s41598-022-21366-2 PMC9547369 36209286

[21] Lai S, Cao Y, Wang P, Lan Y, Liu Z, Bert_plps: a bert-based model for predicting lysine phosphoglycerylation sites, (2023).

[22] Chandra A, Sharma A, Dehzangi I et al. (2023) Predicting phosphoglycerylation with transformer features and deep learning. In *2023 IEEE Asia-Pacific Conference on Computer Science and Data Engineering (CSDE)*. IEEE, pp1–6.

[23] Luo Z, Wang R, Sun Y et al. (2024) Interpretable feature extraction and dimensionality reduction in esm2 for protein localization prediction. *Briefings in Bioinformatics*, 25, bbad534. doi: 10.1093/bib/bbad534 PMC10818170 38279650

[24] Nabeel Asim MN, Ali Ibrahim MA, Imran Malik MI. et al. (2022) El-rmlocnet: an explainable lstm network for rna-associated multi-compartment localization prediction. *Computational and Structural Biotechnology Journal*, 20, 3986–4002. doi: 10.1016/j.csbj.2022.07.031 35983235 PMC9356161

[25] Wang C, Wang Y, Ding P et al. (2024) Ml-fgat: identification of multi-label protein subcellular localization by interpretable graph attention networks and feature-generative adversarial networks. *Computers in Biology and Medicine*, 170, 107944. doi: 10.1016/j.compbiomed.2024.107944 38215617

[26] Gillani M, Pollastri G (2024) Sclpred-ecl: subcellular localization prediction by deep n-to-1 convolutional neural networks. *International Journal of Molecular Sciences*, 25, 5440. doi: 10.3390/ijms25105440 PMC11121631 38791479

[27] Elnaggar A, Essam H, Salah-Eldin W et al. (2023) Ankh: optimized protein language model unlocks general-purpose modelling. arxiv doi: 10.48550. *arXiv preprint arXiv.2301.06568*.

[28] Thumuluri V, Juan Almagro Armenteros JJ, Rosenberg Johansen A et al. (2022) Deeploc 2.0: multi-label subcellular localization prediction using protein language models. *Nucleic Acids research*, 50, W228–W234. doi: 10.1093/nar/gkac278 35489069 PMC9252801

[29] Nabeel Asim M, Ali Ibrahim M, Zehe C et al. (2021) L2s-mirloc: a lightweight two stage miRNA sub-cellular localization prediction framework. In *2021 International Joint Conference on Neural Networks (IJCNN)*. IEEE, pp1–8.

[30] Pan X, Chen L, Liu M et al. (2022) Identifying protein subcellular locations with embeddings-based node2loc. *IEEE/ACM Transactions on Computational Biology and Bioinformatics*, 19, 666–675. doi: 10.1109/TCBB.2021.3080386 33989156

[31] Pan X, Chen L, Liu M et al. (2019) Predicting protein subcellular location using learned distributed representations from a protein–protein network. *BioRxiv*, 768739.

[32] Asgari E, McHardy AC, Mofrad MRK (2019) Probabilistic variable-length segmentation of protein sequences for discriminative motif discovery (dimotif) and sequence embedding (protvecx). *Scientific reports*, 9, 3577. doi: 10.1038/s41598-019-38746-w PMC6401088 30837494

[33] Heinzinger M, Elnaggar A, Wang Y et al. (2019) Modeling aspects of the language of life through transfer-learning protein sequences. *BMC bioinformatics*, 20, 1–17. doi: 10.1186/s12859-019-3220-8 31847804 PMC6918593

[34] Nabeel Asim MN, Ali Ibrahim MA, Imran Malik M et al. (2021) Advances in computational methodologies for classification and sub-cellular locality prediction of non-coding RNAs. *International Journal of Molecular Sciences*, 22, 8719. doi: 10.3390/ijms22168719 PMC8395733 34445436

[35] Dang TH, Vu TA (2024) xCAPT5: protein–protein interaction prediction using deep and wide multi-kernel pooling convolutional neural networks with protein language model. *BMC bioinformatics*, 25, 106. doi: 10.1186/s12859-024-05725-6 PMC10924985 38461247

[36] Wenjian M, Xiangpeng B, Jiang H et al. (2024) Collappi: a collaborative learning framework for predicting protein–protein interactions. *IEEE Journal of Biomedical and Health Informatics*. 10.1109/JBHI.2024.3375621 38466584

[37] Zhang F, Chang S, Wang B, et al. (2024) DSSGNN-PPI: a protein–protein interactions prediction model based on Double Structure and Sequence graph neural networks. *Computers in Biology and Medicine*, 177, 108669. doi: 10.1016/j.compbiomed.2024.108669 38833802

[38] Kang Y, Wang X, Xie C et al. (2023) BBLN: a bilateral-branch learning network for unknown protein–protein interaction prediction. *Computers in Biology and Medicine*, 167, 107588. doi: 10.1016/j.compbiomed.2023.107588 37918265

[39] Banu Ozger ZB (2023) A robust protein language model for SARS-CoV-2 protein–protein interaction network prediction. *Artificial Intelligence in Medicine*, 142, 1–14. doi: 10.1016/j.artmed.2023.102574 PMC10162945 37316102

[40] Zheng J, Yang X, Huang Y et al. (2023) Deep learning-assisted prediction of protein–protein interactions in Arabidopsis thaliana. *The Plant Journal*, 114, 984–994. doi: 10.1111/tpj.16188 36919205

[41] Hu J, Dong M, Tang Y-X, et al. (2023) Improving protein–protein interaction site prediction using deep residual neural network. *Analytical Biochemistry*, 670, 1–9. doi: 10.1016/j.ab.2023.115132 36997014

[42] Nambiar A, Liu S, Heflin M et al. (2023) Transformer neural networks for protein family and interaction prediction tasks. *Journal of Computational Biology*, 30, 95–111. doi: 10.1089/cmb.2022.0132 35950958

[43] Mou M, Pan Z, Zhou Z et al. (2023) A transformer-based ensemble framework for the prediction of protein–protein interaction sites. *Research*, 6, 1–16. doi: 10.34133/research.0240 PMC10528219 37771850

[44] Kang Y, Elofsson A, Jiang Y et al. (2023) Aftgan: prediction of multi-type ppi based on attention free transformer and graph attention network. *Bioinformatics*, 39, btad052. doi: 10.1093/bioinformatics/btad052 PMC9897180 36692145

[45] Zhang F, Zhang Y, Zhu X et al. (2023) Deepsg2ppi: a protein–protein interaction prediction method based on deep learning. *IEEE/ACM Transactions on Computational Biology and Bioinformatics*, 20, 2907–2919. doi: 10.1109/TCBB.2023.3268661 37079417

[46] Jha K, Saha S, Karmakar S (2023) Prediction of protein–protein interactions using vision transformer and language model. *IEEE/ACM Transactions on Computational Biology and Bioinformatics*, 20, 3215–3225. doi: 10.1109/TCBB.2023.3248797 37027644

[47] Lanchantin J, Weingarten T, Sekhon A et al. (2021) Transfer learning for predicting virus-host protein interactions for novel virus sequences. In *Proceedings of the 12th ACM Conference on Bioinformatics, Computational Biology, and Health Informatics*. pp1–10.

[48] Yang X, Wuchty S, Liang Z et al. (2024) Multi-modal features-based human-herpesvirus protein–protein interaction prediction by using LightGBM. *Briefings in Bioinformatics*, 25, bbae005. doi: 10.1093/bib/bbae005 PMC10818167 38279649

[49] Xie P, Zhuang J, Tian G, et al. (2023) Emvirus: an embedding-based neural framework for human-virus protein–protein interactions prediction. *Biosafety and Health*, 5, 152–158. doi: 10.1016/j.bsheal.2023.04.003 37362223 PMC10166638

[50] Chakraborty A, Mitra S, Bhattacharjee M et al. (2023) Determining human-coronavirus protein–protein interaction using machine intelligence. *Medicine in Novel Technology and Devices*, 18, 100228. doi: 10.1016/j.medntd.2023.100228 PMC10077817 37056696

[51] Xia S, Xia Y, Xiang C et al. (2022) A virus–target host proteins recognition method based on integrated complexes data and seed extension. *BMC bioinformatics*, 23, 256. doi: 10.1186/s12859-022-04792-x PMC9238269 35764916

[52] Nabeel Asim MN, Fazeel A, Ali Ibrahim MA et al. (2022) MP-VHPPI: meta predictor for viral host protein–protein interaction prediction in multiple hosts and viruses. *Frontiers in Medicine*, 9, 1–20. doi: 10.3389/fmed.2022.1025887 PMC9709337 36465911

[53] Charoenkwan P, Chiangjong W, Sanghiran Lee VS et al. (2021) Improved prediction and characterization of anticancer activities of peptides using a novel flexible scoring card method. *Scientific reports*, 11, 3017. doi: 10.1038/s41598-021-82513-9 PMC7862624 33542286

[54] Sharma R, Shrivastava S, Kumar Singh S et al. (2021) Deep-abppred: identifying antibacterial peptides in protein sequences using bidirectional lstm with word2vec. *Briefings in Bioinformatics*, 22, bbab065. doi: 10.1093/bib/bbab065 33784381

[55] Bournez C, Riool M, de Boer L et al. (2023) Calcamp: a new machine learning model for the accurate prediction of antimicrobial activity of peptides. *Antibiotics*, 12, 725. doi: 10.3390/antibiotics12040725 PMC10135148 37107088

[56] Kha Q-H, Ho Q-T, Quoc Khanh Le NQK (2022) Identifying snare proteins using an alignment-free method based on multiscan convolutional neural network and pssm profiles. *Journal of Chemical Information and Modeling*, 62, 4820–4826. doi: 10.1021/acs.jcim.2c01034 36166351 PMC9554904

[57] Quang-Thai H, Phan V-D, Yu-Yen O et al. (2020) Use chou’s 5-steps rule with different word embedding types to boost performance of electron transport protein prediction model. *IEEE/ACM Transactions on Computational Biology and Bioinformatics*, 19, 1235–1244. doi: 10.1109/TCBB.2020.3010975 32750894

[58] Kim S, Mollaei P, Antony A et al. (2024) Gpcr-bert: interpreting sequential design of G protein-coupled receptors using protein language models. *Journal of Chemical Information and Modeling*, 64, 1134–1144. doi: 10.1021/acs.jcim.3c01706 38340054 PMC10900288

[59] Cai K, Zhu Y. (2022) A method for identifying essential proteins based on deep convolutional neural network architecture with particle swarm optimization. In *2022 Asia Conference on Advanced Robotics, Automation, and Control Engineering (ARACE)*. IEEE, pp7–12.

[60] Xu S, Onoda A (2024) Accurate and fast prediction of intrinsically disordered protein by multiple protein language models and ensemble learning. *Journal of Chemical Information and Modeling*, 64, 2901–2911. doi: 10.1021/acs.jcim.3c01202 37883249

[61] Koyama T, Tsumura H, Matsumoto S et al. (2024) Chemglam: chemical genomics language models for compound-protein interaction prediction. *bioRxiv*, 2024–02.

[62] Shuting X, Wang R. (2023) Odindta: combining mutual attention and pre-training for drug-target affinity prediction. In *2023 IEEE 35th International Conference on Tools with Artificial Intelligence (ICTAI)*. IEEE, pp680–687.

[63] Lin S, Shi C, Chen J (2022) Generalizeddta: combining pre-training and multi-task learning to predict drug-target binding affinity for unknown drug discovery. *BMC bioinformatics*, 23, 367. doi: 10.1186/s12859-022-04905-6 PMC9449940 36071406

[64] Zhao L, Xie P, Hao L et al. (2020) Gene ontology aided compound protein binding affinity prediction using bert encoding. In *2020 IEEE International Conference on Bioinformatics and Biomedicine (BIBM)*. IEEE, pp1231–1236.

[65] Ray S, Lall S, Bandyopadhyay S (2022) A deep integrated framework for predicting SARS-CoV2-human protein–protein interaction. *IEEE Transactions on Emerging Topics in Computational Intelligence*, 6, 1463–1472. doi: 10.1109/TETCI.2022.3182354

[66] Ezziane Z (2006) Applications of artificial intelligence in bioinformatics: a review. *Expert Systems With Applications*, 30, 2–10. doi: 10.1016/j.eswa.2005.09.042

[67] Whitfield EJ, Pruess M, Apweiler R (2006) Bioinformatics database infrastructure for biotechnology research. *Journal of biotechnology*, 124, 629–639. doi: 10.1016/j.jbiotec.2006.04.006 16757051

[68] Sung Park J, Bernstein MS, Brewer RN et al. (2021) Understanding the representation and representativeness of age in ai data sets. In *Proceedings of the 2021 AAAI/ACM Conference on AI, Ethics, and Society*. pp834–842.

[69] Busuioc M (2021) Accountable artificial intelligence: holding algorithms to account. *Public Administration review*, 81, 825–836. doi: 10.1111/puar.13293 34690372 PMC8518786

[70] Nabeel Asim M, Ali Ibrahim M, Fazeel A et al. (2023) Dna-mp: a generalized dna modifications predictor for multiple species based on powerful sequence encoding method. *Briefings in Bioinformatics*, 24, bbac546. doi: 10.1093/bib/bbac546 36528802

[71] Nabeel Asim MN, Ali Ibrahim MA, Zaib A, et al. (2025) DNA sequence analysis landscape: a comprehensive review of DNA sequence analysis task types, databases, datasets, word embedding methods, and language models. *Heliyon*, 12, 1–64. doi: 10.3389/fmed.2025.1503229 PMC12011883 40265190

[72] Szklarczyk D, Kirsch R, Koutrouli M, et al. (2023) The STRING database in 2023: protein–protein association networks and functional enrichment analyses for any sequenced genome of interest. *Nucleic Acids research*, 51, D638–D646. doi: 10.1093/nar/gkac1000 36370105 PMC9825434

[73] McArthur AG, Waglechner N, Nizam F, et al. (2013) The comprehensive antibiotic resistance database. *Antimicrobial Agents and chemotherapy*, 57, 3348–3357. doi: 10.1128/AAC.00419-13 23650175 PMC3697360

[74] Sasidharan Nair PS, Vihinen M (2013) VariBench: a benchmark database for variations. *Human mutation*, 34, 42–49. doi: 10.1002/humu.22204 22903802

[75] Landrum MJ, Chitipiralla S, Brown GR, Chen C, Baoshan G, Hart J, Hoffman D, Jang W, Kaur K, Liu C, Clinvar: improvements to accessing data, (2020), D835–D844 et al. *Nucleic acids research*, 48(D1. 31777943 10.1093/nar/gkz972 PMC6943040

[76] Zhang C, Zhang X, Freddolino PL, et al. (2024) BioLiP2: An updated structure database for biologically relevant ligand-protein interactions. *Nucleic Acids Research*, 52, D404–D412. doi: 10.1093/nar/gkad630 37522378 PMC10767969

[77] Nusinow DP, Szpyt J, Ghandi M, et al. (2020) Quantitative proteomics of the cancer cell line encyclopedia. *Cell*, 180, 387–402. doi: 10.1016/j.cell.2019.12.023 31978347 PMC7339254

[78] Jenuth JP (1999) The ncbi: publicly available tools and resources on the web. *Bioinformatics Methods and protocols*, 132, 301–312. doi: 10.1385/1-59259-192-2:301 10547843

[79] Boschiero C, Dai X, Knut Lundquist PK et al. (2020) MtSSPdb: the Medicago truncatula Small Secreted Peptide Database. *Plant Physiology*, 183, 399–413. doi: 10.1104/pp.19.01088 32079733 PMC7210635

[80] Edgar R (2002) Gene expression omnibus: Ncbi gene expression and hybridization array data repository. *Nucleic Acids research*, 30, 207–210. doi: 10.1093/nar/30.1.207 11752295 PMC99122

[81] Kanehisa M . The kegg database. In *‘In silico’ simulation of biological processes: Novartis Foundation Symposium*, 247, 91–103. Wiley Online Library, 2002. 12539951

[82] Wu J, Vallenius T, Ovaska K et al. (2009) Integrated network analysis platform for protein–protein interactions. *Nature methods*, 6, 75–77. doi: 10.1038/nmeth.1282 19079255

[83] Madeira F, Pearce M, Tivey ARN et al. (2022) Search and sequence analysis tools services from embl-ebi in 2022. *Nucleic Acids research*, 50, W276–W279. doi: 10.1093/nar/gkac240 35412617 PMC9252731

[84] Amberger JS, Bocchini CA, Scott AF, et al. (2019) OMIM.org: leveraging knowledge across phenotype–gene relationships. *Nucleic Acids research*, 47, D1038–D1043. doi: 10.1093/nar/gky1151 30445645 PMC6323937

[85] Kovaltsuk A, Leem J, Kelm S et al. (2018) Observed antibody space: A resource for data mining next-generation sequencing of antibody repertoires. *The Journal of Immunology*, 201, 2502–2509. doi: 10.4049/jimmunol.1800708 30217829

[86] Safran M, Dalah I, Alexander J, et al. (2010) GeneCards Version 3: the human gene integrator. *Database*, 2010, baq020. doi: 10.1093/database/baq020 PMC2938269 20689021

[87] Manso T, Folch G, Giudicelli V, et al. (2022) IMGT$\circledR$ databases, related tools and web resources through three main axes of research and development. *Nucleic Acids research*, 50, D1262–D1272. doi: 10.1093/nar/gkab1136 34875068 PMC8728119

[88] Sondka Z, Bindal Dhir NB, Carvalho-Silva D, et al. (2024) COSMIC: A curated database of somatic variants and clinical data for cancer. *Nucleic Acids Research*, 52, D1210–D1217. doi: 10.1093/nar/gkad986 38183204 PMC10767972

[89] Ammari MG, Gresham CR, McCarthy FM, et al. (2016) HPIDB 2.0: a curated database for host–pathogen interactions. *Database*, 2016, baw103. doi: 10.1093/database/baw103 PMC4930832 27374121

[90] Espe S (2018) Malacards: the human disease database. *Journal of the Medical Library Association: JMLA*, 106, 140. doi: 10.5195/jmla.2018.253

[91] Zdrazil B, Felix E, Hunter F, et al. (2024) The chembl database in 2023: a drug discovery platform spanning multiple bioactivity data types and time periods. *Nucleic Acids research*, 52, D1180–D1192. doi: 10.1093/nar/gkad1004 37933841 PMC10767899

[92] Mysinger MM, Carchia M, Irwin JJ, et al. (2012) Directory of useful decoys, enhanced (dud-e): better ligands and decoys for better benchmarking. *Journal of Medicinal chemistry*, 55, 6582–6594. doi: 10.1021/jm300687e 22716043 PMC3405771

[93] Liu T, Lin Y, Wen X et al. (2007) BindingDB: a web-accessible database of experimentally determined protein–ligand binding affinities. *Nucleic Acids research*, 35, D198–D201. doi: 10.1093/nar/gkl999 17145705 PMC1751547

[94] Szklarczyk D, Santos A, Von Mering C et al. (2016) STITCH 5: augmenting protein–chemical interaction networks with tissue and affinity data. *Nucleic Acids research*, 44, D380–D384. doi: 10.1093/nar/gkv1277 26590256 PMC4702904

[95] Huang N, Shoichet BK, Irwin JJ (2006) Benchmarking sets for molecular docking. *Journal of Medicinal chemistry*, 49, 6789–6801. doi: 10.1021/jm0608356 17154509 PMC3383317

[96] Tickotsky N, Sagiv T, Prilusky J et al. (2017) Mcpas-tcr: a manually curated catalogue of pathology-associated T cell receptor sequences. *Bioinformatics*, 33, 2924–2929. doi: 10.1093/bioinformatics/btx286 28481982

[97] Bagaev DV, Vroomans RMA, Samir J, Stervbo U, Rius C, Dolton G, Greenshields-Watson A, Attaf M, Egorov ES, Zvyagin IV, Vdjdb in 2019: database extension, new analysis infrastructure and a T-cell receptor motif compendium, D1057–D1062 et al. *Nucleic acids research*, 48(D1):2020. 10.1093/nar/gkz874 PMC6943061 31588507

[98] Zhang W, Wang L, Liu K et al. (2020) Pird: pan immune repertoire database. *Bioinformatics*, 36, 897–903. doi: 10.1093/bioinformatics/btz614 31373607

[99] Richardson L, Allen B, Baldi G, et al. (2023) Mgnify: the microbiome sequence data analysis resource in 2023. *Nucleic Acids Research*, 51, D753–D759. doi: 10.1093/nar/gkac1080 36477304 PMC9825492

[100] Vita R, Mahajan S, Overton JA et al. (2019) The immune epitope database (iedb): 2018 update. *Nucleic Acids research*, 47, D339–D343. doi: 10.1093/nar/gky1006 30357391 PMC6324067

[101] Blohm P, Frishman G, Smialowski P et al. (2014) Negatome 2.0: a database of non-interacting proteins derived by literature mining, manual annotation and protein structure analysis. *Nucleic Acids research*, 42, D396–D400. doi: 10.1093/nar/gkt1079 24214996 PMC3965096

[102] Kim S, Chen J, Cheng T, et al. (2023) Pubchem 2023 update. *Nucleic Acids research*, 51, D1373–D1380. doi: 10.1093/nar/gkac956 36305812 PMC9825602

[103] Peter Davis AP, Wiegers TC, Johnson RJ et al. (2023) Comparative toxicogenomics database (ctd): update 2023. *Nucleic Acids research*, 51, D1257–D1262. doi: 10.1093/nar/gkac833 36169237 PMC9825590

[104] Hermjakob H et al. (2004) Intact: an open source molecular interaction database. *Nucleic Acids research*, 32, 452D–455. doi: 10.1093/nar/gkh052 PMC308786 14681455

[105] Chang A, Jeske L, Ulbrich S et al. (2021) Brenda, the elixir core data resource in 2021: new developments and updates. *Nucleic Acids research*, 49, D498–D508. doi: 10.1093/nar/gkaa1025 33211880 PMC7779020

[106] Quaglia F, Mészáros B, Salladini E, et al. (2022) Disprot in 2022: improved quality and accessibility of protein intrinsic disorder annotation. *Nucleic Acids research*, 50, D480–D487. doi: 10.1093/nar/gkab1082 34850135 PMC8728214

[107] Terzian P, Olo Ndela E, Galiez C et al. (2021) Phrog: families of prokaryotic virus proteins clustered using remote homology. *NAR Genomics and Bioinformatics*, 3, lqab067. doi: 10.1093/nargab/lqab067 PMC8341000 34377978

[108] Zitnik M, Sosic R, Leskovec J (2018) Biosnap datasets: Stanford biomedical network dataset collection. 5, http://snap.stanford.edu/Biodata Cited by.

[109] Alanis-Lobato G, Andrade-Navarro MA, Schaefer MH (2017) HIPPIE v2.0: enhancing meaningfulness and reliability of protein–protein interaction networks. *Nucleic Acids research*, 45, kw985. doi: 10.1093/nar/gkw985 g. PMC5210659 27794551

[110] Burley SK, Berman HM, Kleywegt GJ et al. (2017) Protein data bank (pdb): the single global macromolecular structure archive. *Protein crystallography: methods and protocols*, 1607, 627–641. doi: 10.1093/nar/28.1.235 PMC5823500 28573592

[111] Ben Chorin A, Masrati G, Kessel A et al. (2020) Consurf-db: an accessible repository for the evolutionary conservation patterns of the majority of pdb proteins. *Protein Science*, 29, 258–267. doi: 10.1002/pro.3779 31702846 PMC6933843

[112] Li Z, Li S, Luo M, et al. (2022) dbptm in 2022: an updated database for exploring regulatory networks and functional associations of protein post-translational modifications. *Nucleic Acids research*, 50, D471–D479. doi: 10.1093/nar/gkab1017 34788852 PMC8728263

[113] Wang Y, Zhang S, Li F, et al. (2020) Therapeutic target database 2020: enriched resource for facilitating research and early development of targeted therapeutics. *Nucleic Acids research*, 48, D1031–D1041. doi: 10.1093/nar/gkz981 31691823 PMC7145558

[114] Diella F, Cameron S, Gemünd C et al. (2004) *BMC bioinformatics*, 5, 1–5. doi: 10.1186/1471-2105-5-79 Phospho.ELM: a database of experimentally verified phosphorylation sites in eukaryotic proteins. 15212693 PMC449700

[115] Sigrist CJA, Cerutti L, De Castro E et al. (2010) Prosite, a protein domain database for functional characterization and annotation. *Nucleic Acids research*, 38, D161–D166. doi: 10.1093/nar/gkp885 19858104 PMC2808866

[116] Bateman A, Martin M-J, Orchard S et al. (2023) Uniprot: the universal protein knowledgebase in 2023. *Nucleic Acids research*, 51, D523–D531. doi: 10.1093/nar/gkac1052 36408920 PMC9825514

[117] Dunbar J, Krawczyk K, Leem J et al. (2014) Sabdab: the structural antibody database. *Nucleic Acids research*, 42, D1140–D1146. doi: 10.1093/nar/gkt1043 24214988 PMC3965125

[118] Wang R, Fang X, Lu Y, et al. (2004) The PDBbind database: collection of binding affinities for protein–ligand complexes with known three-dimensional structures. *Journal of Medicinal chemistry*, 47, 2977–2980. doi: 10.1021/jm030580l 15163179

[119] Hornbeck PV, Kornhauser JM, Tkachev S et al. (2012) Phosphositeplus: a comprehensive resource for investigating the structure and function of experimentally determined post-translational modifications in man and mouse. *Nucleic Acids research*, 40, D261–D270. doi: 10.1093/nar/gkr1122 22135298 PMC3245126

[120] Blum M, Chang H-Y, Chuguransky S, et al. (2021) The interpro protein families and domains database: 20 years on. *Nucleic Acids research*, 49, D344–D354. doi: 10.1093/nar/gkaa977 33156333 PMC7778928

[121] Varadi M, Anyango S, Deshpande M, et al. (2022) Alphafold protein structure database: massively expanding the structural coverage of protein-sequence space with high-accuracy models. *Nucleic Acids research*, 50, D439–D444. doi: 10.1093/nar/gkab1061 34791371 PMC8728224

[122] Varadi M, De Baets G, Vranken WF et al. (2018) Amypro: a database of proteins with validated amyloidogenic regions. *Nucleic Acids research*, 46, D387–D392. doi: 10.1093/nar/gkx950 29040693 PMC5753394

[123] Piovesan D, Del Conte A, Clementel D et al. (2023) MobiDB: 10 years of intrinsically disordered proteins. *Nucleic Acids research*, 51, D438–D444. doi: 10.1093/nar/gkac1065 36416266 PMC9825420

[124] Maccari G, Robinson J, Ballingall K, et al. (2017) Ipd-mhc 2.0: an improved inter-species database for the study of the major histocompatibility complex. *Nucleic Acids research*, 45, D860–D864. doi: 10.1093/nar/gkw1050 27899604 PMC5210539

[125] Oughtred R, Rust J, Chang C, et al. (2021) The BioGRID database: a comprehensive biomedical resource of curated protein, genetic, and chemical interactions. *Protein Science*, 30, 187–200. doi: 10.1002/pro.3978 33070389 PMC7737760

[126] Licata L, Briganti L, Peluso D, et al. (2012) Mint, the molecular interaction database: 2012 update. *Nucleic Acids research*, 40, D857–D861. doi: 10.1093/nar/gkr930 22096227 PMC3244991

[127] Gurumayum S, Jiang P, Hao X, et al. (2021) Ogee v3: online gene essentiality database with increased coverage of organisms and human cell lines. *Nucleic Acids research*, 49, D998–D1003. doi: 10.1093/nar/gkaa884 33084874 PMC7779042

[128] Xenarios I (2002) Dip, the database of interacting proteins: a research tool for studying cellular networks of protein interactions. *Nucleic Acids research*, 30, 303–305. doi: 10.1093/nar/30.1.303 11752321 PMC99070

[129] Leem J, OSH, Krawczyk K, et al. (2018) Stcrdab: the structural t-cell receptor database. *Nucleic Acids research*, 46, D406–D412. doi: 10.1093/nar/gkx971 29087479 PMC5753249

[130] Mirdita M, VonDenDriesch L, Galiez C et al. (2017) Uniclust databases of clustered and deeply annotated protein sequences and alignments. *Nucleic Acids research*, 45, D170–D176. doi: 10.1093/nar/gkw1081 27899574 PMC5614098

[131] Chan WKB, Zhang H, Yang J et al. (2015) Glass: a comprehensive database for experimentally validated gpcr-ligand associations. *Bioinformatics*, 31, 3035–3042. doi: 10.1093/bioinformatics/btv302 25971743 PMC4668776

[132] Fox NK, Brenner SE, Chandonia J-M (2014) SCOPe: structural classification of proteins–extended, integrating SCOP and astral data and classification of new structures. *Nucleic Acids research*, 42, D304–D309. doi: 10.1093/nar/gkt1240 24304899 PMC3965108

[133] Saier MH, Reddy VS, Moreno-Hagelsieb G, et al. (2021) The transporter classification database (TCDB): 2021 update. *Nucleic Acids research*, 49, D461–D467. doi: 10.1093/nar/gkaa1004 33170213 PMC7778945

[134] Huntley RP, Sawford T, Mutowo-Meullenet P et al. (2015) The goa database: gene ontology annotation updates for 2015. *Nucleic Acids Research*, 43, D1057–D1063. doi: 10.1093/nar/gku1113 25378336 PMC4383930

[135] Berman HM (2000) The protein data bank. *Nucleic Acids Research*, 28, 235–242. doi: 10.1093/nar/28.1.235 10592235 PMC102472

[136] An J, Weng X (2022) Collectively encoding protein properties enriches protein language models. *BMC Bioinformatics*, 23, 467. doi: 10.1186/s12859-022-05031-z PMC9641823 36348281

[137] Pándy-Szekeres G, Caroli J, Mamyrbekov A et al. (2023) Gpcrdb in 2023: state-specific structure models using alphafold2 and new ligand resources. *Nucleic Acids research*, 51, D395–D402. doi: 10.1093/nar/gkac1013 36395823 PMC9825476

[138] Sillitoe I, Bordin N, Dawson N, et al. (2021) Cath: increased structural coverage of functional space. *Nucleic Acids research*, 49, D266–D273. doi: 10.1093/nar/gkaa1079 33237325 PMC7778904

[139] Pi nero J, Queralt-Rosinach N, Bravo A et al. (2015) Disgenet: a discovery platform for the dynamical exploration of human diseases and their genes. *Database*, 2015, bav028. doi: 10.1093/database/bav028 PMC4397996 25877637

[140] Balamurugan R, Mohite S, Raja SP (2023) Protein sequence classification using bidirectional encoder representations from transformers (bert) approach. *SN Computer Science*, 4, 481. doi: 10.1007/s42979-023-01980-1

[141] Muazzam Ali Shah SM, Wellem Taju SW, Ho Q-T et al. (2021) GT-Finder: classify the family of glucose transporters with pre-trained BERT language models. *Computers in Biology and medicine*, 131, 1–11. doi: 10.1016/j.compbiomed.2021.104259 33581474

[142] Liu Y, Liu Y, Wang G-A et al. (2022) Bert-kgly: a bidirectional encoder representations from transformers (BERT)-based model for predicting lysine glycation site for homo sapiens. *Frontiers in Bioinformatics*, 2, 1–12. doi: 10.3389/fbinf.2022.834153 PMC9580886 36304324

[143] Meng L, Chen X, Cheng K et al. (2024) Transptm: a transformer-based model for non-histone acetylation site prediction. *Briefings in Bioinformatics*, 25, bbae219. doi: 10.1093/bib/bbae219 PMC11082075 38725156

[144] Wang X, Ding Z, Wang R, et al. (2023) Deepro-glu: combination of convolutional neural network and bi-lstm models using protbert and handcrafted features to identify lysine glutarylation sites. *Briefings in Bioinformatics*, 24, bbac631. doi: 10.1093/bib/bbac631 36653898

[145] Jha K, Karmakar S, Saha S (2023) Graph-BERT and language model-based framework for protein–protein interaction identification. *Scientific Reports*, 13, 5663. doi: 10.1038/s41598-023-31612-w PMC10079975 37024543

[146] Minghao X, Zhang Z, Jiarui L et al. (2022) Peer: a comprehensive and multi-task benchmark for protein sequence understanding. *Advances in Neural Information Processing Systems*, 35, 35156–35173. doi: 10.5555/3600270.3602818

[147] Madan S, Demina V, Stapf M et al. (2022) Accurate prediction of virus-host protein–protein interactions via a Siamese neural network using deep protein sequence embeddings. *Patterns*, 3, 100551. doi: 10.1016/j.patter.2022.100551 PMC9481957 36124304

[148] Zhang N, Zhen B, Liang X et al. (2022) Ontoprotein: protein pretraining with gene ontology embedding. *ArXiv Preprint arXiv:2201.11147*.

[149] Ieremie I, Ewing RM, Niranjan M et al. (2022) TransformerGO: predicting protein–protein interactions by modelling the attention between sets of gene ontology terms. *Bioinformatics*, 38, 2269–2277. doi: 10.1093/bioinformatics/btac104 35176146 PMC9363134

[150] Chen B, Cheng X, Pan L, et al. (2024) xtrimopglm: unified 100b-scale pre-trained transformer for deciphering the language of protein. *ArXiv Preprint arXiv:2401.06199*.

[151] Si Y, Yan C (2024) Protein language model-embedded geometric graphs power inter-protein contact prediction. *Elife*, 12, RP92184. doi: 10.7554/eLife.92184 PMC10987090 38564241

[152] Si Y, Yan C (2023) Improved inter-protein contact prediction using dimensional hybrid residual networks and protein language models. *Briefings in Bioinformatics*, 24, bbad039. doi: 10.1093/bib/bbad039 36759333

[153] Singh J, Litfin T, Singh J et al. (2022) Spot-contact-lm: improving single-sequence-based prediction of protein contact map using a transformer language model. *Bioinformatics*, 38, 1888–1894. doi: 10.1093/bioinformatics/btac053 35104320 PMC9113311

[154] Xiao Y, Qiu J, Ziang L et al. (2021) Modeling protein using large-scale pretrain language model. *ArXiv Preprint arXiv:2108.07435*.

[155] Rao R, Bhattacharya N, Thomas N et al. (2019) Evaluating protein transfer learning with tape. *Advances in Neural Information Processing systems*, 32. PMC7774645 33390682

[156] Liu Y, Tian B (2023) Protein–DNA binding sites prediction based on pre-trained protein language model and contrastive learning. *Briefings in Bioinformatics*, 25, bbad488. doi: 10.1093/bib/bbad488 PMC10782905 38171929

[157] Roche R, Moussad B, Hossain Shuvo MH et al. (2024) EquiPNAS: improved protein–nucleic acid binding site prediction using protein-language-model-informed equivariant deep graph neural networks. *Nucleic Acids Research*, 52, e27–e27. doi: 10.1093/nar/gkae039 38281252 PMC10954458

[158] Luo H, Shan W, Chen C et al. (2023) Improving language model of human genome for DNA-protein binding prediction based on task-specific pre-training. *Interdisciplinary sciences, Computational Life sciences*, 15, 32–43. doi: 10.1007/s12539-022-00537-9 36136096

[159] Murad T, Ali S, Chourasia P, et al. (2023) Advancing protein–DNA binding site prediction: integrating sequence models and machine learning classifiers. *bioRxiv*, 2023–08.

[160] Zeng W, Dafeng L, Liu X et al. (2023) Esm-nbr: fast and accurate nucleic acid-binding residue prediction via protein language model feature representation and multi-task learning. In *2023 IEEE International Conference on Bioinformatics and Biomedicine (BIBM)*. IEEE, pp76–81.

[161] Jun M, Zhao Z, Tongfeng L et al. (2024) Graphsformercpi: graph transformer for compound–protein interaction prediction. *Interdisciplinary Sciences: computational Life Sciences*, 16, 1–17. doi: 10.1007/s12539-024-00609-y 38457109

[162] Chen L, Tan X, Wang D et al. (2020) TransformerCPI: improving compound–protein interaction prediction by sequence-based deep learning with self-attention mechanism and label reversal experiments. *Bioinformatics*, 36, 4406–4414. doi: 10.1093/bioinformatics/btaa524 32428219

[163] Wang C, Zhu Y, Wen N et al. (2021) Seqgo-cpa: improving compound-protein binding affinity prediction with sequence information and gene ontology knowledge. In *2021 IEEE International Conference on Bioinformatics and Biomedicine (BIBM)*. IEEE, pp354–359.

[164] EdwardMGonzales MEM, Ureta JC, Shrestha AMS et al. (2023) Protein embeddings improve phage-host interaction prediction. *PloS One*, 18, e0289030. doi: 10.1371/journal.pone.0289030 PMC10365317 37486915

[165] Dee W, Gromiha M (2022) Lmpred: predicting antimicrobial peptides using pre-trained language models and deep learning. *Bioinformatics Advances*, 2, vbac021. doi: 10.1093/bioadv/vbac021 PMC9710646 36699381

[166] Chen S, Tan Q, Jingchen L, et al. (2021) Uspnet: unbiased organism-agnostic signal peptide predictor with deep protein language model. *bioRxiv*, 2021–11. 10.1038/s43588-023-00576-2 38177492

[167] Wang R, Zhou Z, Wu X et al. (2024) An effective plant small secretory peptide recognition model based on feature correction strategy. *Journal of Chemical Information and Modeling*, 64, 2798–2806. doi: 10.1021/acs.jcim.3c00868 37643082

[168] Raza A, Uddin J, Almuhaimeed A et al. (2023) Aips-sntcn: predicting anti-inflammatory peptides using fasttext and transformer encoder-based hybrid word embedding with self-normalized temporal convolutional networks. *Journal of Chemical Information and modeling*, 63, 6537–6554. doi: 10.1021/acs.jcim.3c01563 37905969

[169] Melnyk I, Chenthamarakshan V, Chen P-Y. (2023) Payel Das, Amit Dhurandhar, Inkit Padhi, and Devleena Das. Reprogramming pretrained language models for antibody sequence infilling. In *International Conference on Machine Learning*. PMLR, pp24398–24419.

[170] Motmaen A, Dauparas J, Baek M et al. (2023) Peptide-binding specificity prediction using fine-tuned protein structure prediction networks. *Proceedings of the National Academy of Sciences*, 120, e2216697120. doi: 10.1073/pnas.2216697120 PMC9992841 36802421

[171] Zhou Z, Liao Q, Wei J et al. (2024) Revisiting drug–protein interaction prediction: a novel global–local perspective. *Bioinformatics*, 40, btae271. doi: 10.1093/bioinformatics/btae271 PMC11087820 38648052

[172] Zhang Y-L, Wang W-T, Guan J-H et al. (2024) MocFormer: a two-stage pre-training-driven transformer for drug–target interactions prediction. *International Journal of Computational Intelligence Systems*, 17, 165. doi: 10.1007/s44196-024-00561-1

[173] Yang Z, Liu J, Zhu X et al. (2023) Fragdpi: a novel drug-protein interaction prediction model based on fragment understanding and unified coding. *Frontiers of Computer Science*, 17, 175903. doi: 10.1007/s11704-022-2163-9 PMC9745276 36532946

[174] Xia L, Xu L, Pan S et al. (2023) Drug-target binding affinity prediction using message passing neural network and self supervised learning. *BMC genomics*, 24, 557. doi: 10.1186/s12864-023-09664-z PMC10510145 37730555

[175] Saadat M, Behjati A, Zare-Mirakabad F, et al. (2021) Drug-target binding affinity prediction using transformers.

[176] Lennox M, Robertson N, Devereux B. (2021) Modelling drug-target binding affinity using a bert based graph neural network. In *2021 43rd Annual International Conference of the IEEE Engineering in Medicine & Biology Society (EMBC)*. IEEE, pp4348–4353. 10.1109/EMBC46164.2021.9629695 34892183

[177] Kafkas S, Abdelhakim M, Althagafi A et al. (2023) The application of large language models to the phenotype-based prioritization of causative genes in rare disease patients. *medRxiv*, 2023–11. 10.1038/s41598-025-99539-y PMC12041562 40301638

[178] Li Y, Guo Z, Wang K et al. (2023) End-to-end interpretable disease–gene association prediction. *Briefings in bioinformatics*, 24, bbad118. doi: 10.1093/bib/bbad118 36987781

[179] Wang H, Wang X, Liu W et al. (2022) deepdga: biomedical heterogeneous network-based deep learning framework for disease-gene association predictions. In *2022 IEEE International Conference on Bioinformatics and Biomedicine (BIBM)*. IEEE, pp601–606.

[180] Li K, Zhong Y, Lin X, et al. (2020) Predicting the disease risk of protein mutation sequences with pre-training model. *Frontiers in Genetics*, 11, 1–10. doi: 10.3389/fgene.2020.605620 33408741 PMC7780924

[181] Tony T, Krishna G, Aghazadeh A (2023) Protigeno: a prokaryotic short gene finder using protein language models. *ArXiv Preprint arXiv:2307.10343*.

[182] Chen L, Wu R, Zhou F et al. (2023) Hybridgcn for protein solubility prediction with adaptive weighting of multiple features. *Journal of Cheminformatics*, 15, 118. doi: 10.1186/s13321-023-00788-8 PMC10704697 38066570

[183] Filipavicius M, Manica M, Cadow J, et al. (2020) Pre-training protein language models with label-agnostic binding pairs enhances performance in downstream tasks. *ArXiv Preprint arXiv:2012.03084*.

[184] Gong J, Jiang L, Chen Y, et al. (2023) Thplm: a sequence-based deep learning framework for protein stability changes prediction upon point variations using pretrained protein language model. *Bioinformatics*, 39, btad646. doi: 10.1093/bioinformatics/btad646 PMC10627365 37874953

[185] Wang G, Zhang X, Pan Z et al. (2022) Multi-TransDTI: transformer for drug–target interaction prediction based on simple universal dictionaries with multi-view strategy. *Biomolecules*, 12, 644. doi: 10.3390/biom12050644 PMC9138327 35625572

[186] Wang Z, Combs SA, Brand R, et al. (2022) Lm-gvp: an extensible sequence and structure informed deep learning framework for protein property prediction. *Scientific reports*, 12, 6832. doi: 10.1038/s41598-022-10775-y PMC9046255 35477726

[187] Haselbeck F, John M, Zhang Y et al. (2023) Superior protein thermophilicity prediction with protein language model embeddings. *NAR Genomics and Bioinformatics*, 5, lqad087. doi: 10.1093/nargab/lqad087 PMC10566323 37829176

[188] Brandes N, Ofer D, Peleg Y et al. (2022) Proteinbert: a universal deep-learning model of protein sequence and function. *Bioinformatics*, 38, 2102–2110. doi: 10.1093/bioinformatics/btac020 35020807 PMC9386727

[189] Haseeb A, Bashir M, Wali A (2023) Bertdom: protein domain boundary prediction using bert. *Computing and Informatics*, 42, 667–689. doi: 10.31577/cai_2023_3_667

[190] Tawfiq R, Niu K, Hoehndorf R, et al. (2024) Deepgometa: predicting functions for microbes. *bioRxiv*, 2024–01.

[191] Song FV, Su J, Huang S et al. (2024) Deepss2go: protein function prediction from secondary structure. *Briefings in Bioinformatics*, 25, bbae196. doi: 10.1093/bib/bbae196 PMC11066904 38701416

[192] Yuan Q, Tian C, Song Y et al. (2024) Gpsfun: geometry-aware protein sequence function predictions with language models. *Nucleic Acids Research*, 52, gkae381. doi: 10.1093/nar/gkae381 PMC11223820 38738636

[193] Zhapa-Camacho F, Tang Z, Kulmanov M, et al. (2024) Predicting protein functions using positive-unlabeled ranking with ontology-based priors. *bioRxiv*, 2024–01. 10.1093/bioinformatics/btae237 PMC11211813 38940168

[194] Kulmanov M, Guzmán-Vega FJ, Duek Roggli P et al. (2024) Protein function prediction as approximate semantic entailment. *Nature Machine Intelligence*, 6, 1–9. doi: 10.1038/s42256-024-00795-w

[195] Shaw P, Gurram B, Belanger D et al. (2024) Protex: a retrieval-augmented approach for protein function prediction. *bioRxiv*, 2024–05.

[196] Ming Chua Z, Rajesh A, Sinha S, et al. (2024) Protgoat: improved automated protein function predictions using protein language models. *bioRxiv*, 2024–04.

[197] Zhang C, Liu Q, Freddolino L (2024) Starfunc: fusing template-based and deep learning approaches for accurate protein function prediction. *bioRxiv*, 2024–05. 10.1093/gpbjnl/qzag018 41942113

[198] Zhao Y, Yang Z, Wang L et al. (2024) Predicting protein functions based on heterogeneous graph attention technique. *IEEE Journal of Biomedical and Health Informatics*. 10.1109/JBHI.2024.3357834 38319781

[199] Pang Y, Liu B (2024) Disoflag: accurate prediction of protein intrinsic disorder and its functions using graph-based interaction protein language model. *BMC biology*, 22, 3. doi: 10.1186/s12915-023-01803-y PMC10762911 38166858

[200] Derbel H, Zhao Z, Liu Q (2023) Accurate prediction of functional effect of single amino acid variants with deep learning. *Computational and Structural Biotechnology Journal*, 21, 5776–5784. doi: 10.1016/j.csbj.2023.11.017 38074467 PMC10709104

[201] Pang Y, Liu B, Ben-Tal N (2023) Idp-lm: prediction of protein intrinsic disorder and disorder functions based on language models. *PLOS Computational Biology*, 19, e1011657. doi: 10.1371/journal.pcbi.1011657 PMC10699601 37992088

[202] Pei H, Li J, Ma S et al. (2023) Identification of thermophilic proteins based on sequence-based bidirectional representations from transformer-embedding features. *Applied Sciences*, 13, 2858. doi: 10.3390/app13052858

[203] Yuan Q, Xie J, Xie J et al. (2023) Fast and accurate protein function prediction from sequence through pretrained language model and homology-based label diffusion. *Briefings in bioinformatics*, 24, bbad117. doi: 10.1093/bib/bbad117 36964722

[204] Zhao Y, Yang Z, Hong Y et al. (2023) Protein function prediction with functional and topological knowledge of gene ontology. *IEEE Transactions on NanoBioscience*, 22, 755–762. doi: 10.1109/TNB.2023.3278033 37204950

[205] Kabir A, Shehu A (2022) Goproformer: a multi-modal transformer method for gene ontology protein function prediction. *Biomolecules*, 12, 1709. doi: 10.3390/biom12111709 PMC9687818 36421723

[206] Zhao C, Liu T, Wang Z (2022) Panda2: protein function prediction using graph neural networks. *NAR Genomics and bioinformatics*, 4, lqac004. doi: 10.1093/nargab/lqac004 PMC8808544 35118378

[207] Mingyang H, Yuan F, Yang K et al. (2022) Exploring evolution-aware &-free protein language models as protein function predictors. *Advances in Neural Information Processing Systems*, 35, 38873–38884. doi: 10.5555/3600270.3603087

[208] Heinzinger M, Weissenow K, Gomez Sanchez J et al. (2023) Prostt5: bilingual language model for protein sequence and structure. *biorxiv.*. 10.1093/nargab/lqae150 PMC11616678 39633723

[209] Feng C, Wang Z, Guokun L et al. (2022) Bert-ppii: the polyproline type ii helix structure prediction model based on bert and multichannel cnn. *BioMed Research International*, 2022, 9015123. doi: 10.1155/2022/9015123 PMC9433275 36060139

[210] Lin Z, Akin H, Rao R, et al. Language models of protein sequences at the scale of evolution enable accurate structure prediction. *BioRxiv*, 2022, 2022.

[211] Weissenow K, Heinzinger M, Rost B (2022) Protein language-model embeddings for fast, accurate, and alignment-free protein structure prediction. *Structure*, 30, 1169–1177. doi: 10.1016/j.str.2022.05.001 35609601

[212] Elnaggar A, Heinzinger M, Dallago C, et al. (2021) Prottrans: toward understanding the language of life through self-supervised learning. *IEEE Transactions on Pattern Analysis and Machine intelligence*, 44, 7112–7127. doi: 10.1109/TPAMI.2021.3095381 34232869

[213] Rives A, Meier J, Sercu T, et al. (2021) Biological structure and function emerge from scaling unsupervised learning to 250 million protein sequences. *Proceedings of the National Academy of Sciences*, 118, e2016239118. doi: 10.1073/pnas.2016239118 PMC8053943 33876751

[214] Villegas-Morcillo A, Gomez AM, Sanchez V (2022) An analysis of protein language model embeddings for fold prediction. *Briefings in Bioinformatics*, 23, bbac142. doi: 10.1093/bib/bbac142 35443054

[215] Nallapareddy V, Bordin N, Sillitoe I et al. (2023) Cathe: detection of remote homologues for cath superfamilies using embeddings from protein language models. *Bioinformatics*, 39, btad029. doi: 10.1093/bioinformatics/btad029 PMC9887088 36648327

[216] Heinzinger M, Weissenow K, Gomez Sanchez J et al. (2023) Bilingual language model for protein sequence and structure. *bioRxiv*, 2023–07. 10.1093/nargab/lqae150 PMC11616678 39633723

[217] Muazzam Ali Shah S, Yu-Yen O (2023) Disto-trp: an approach for identifying transient receptor potential (trp) channels using structural information generated by alphafold. *Gene*, 871, 1–9. doi: 10.1016/j.gene.2023.147435 37075925

[218] Wang J, Zhou H, Wang Y et al. (2023) Prediction of submitochondrial proteins localization based on gene ontology. *Computers in Biology and Medicine*, 167, 1–9. doi: 10.1016/j.compbiomed.2023.107589 37883850

[219] Wang X, Han L, Wang R, et al. (2023) Dadl-schlo: protein subchloroplast localization prediction based on generative adversarial networks and pre-trained protein language model. *Briefings in Bioinformatics*, 24, bbad083. doi: 10.1093/bib/bbad083 36929854

[220] Tzavella K, Diaz A, Olsen C, et al. (2023) Combining evolution and protein language models for an interpretable cancer driver mutation prediction with d2deep. *bioRxiv*, 2023–11. 10.1093/bib/bbae664 PMC11663023 39708841

[221] Wang J, Chen S, Yuan Q et al. (2024) Predicting the effects of mutations on protein solubility using graph convolution network and protein language model representation. *Journal of Computational Chemistry*, 45, 436–445. doi: 10.1002/jcc.27249 37933773

[222] Meier J, Rao R, Verkuil R et al. (2021) Language models enable zero-shot prediction of the effects of mutations on protein function. *Advances in Neural Information Processing systems*, 34, 29287–29303. doi: 10.1101/2021.07.09.450648

[223] Strokach A, Yu Lu T, Kim PM (2021) Elaspic2 (el2): combining contextualized language models and graph neural networks to predict effects of mutations. *Journal of Molecular biology*, 433, 166810. doi: 10.1016/j.jmb.2021.166810 33450251

[224] Marquet C, Heinzinger M, Olenyi T et al. (2022) Embeddings from protein language models predict conservation and variant effects. *Human genetics*, 141, 1629–1647. doi: 10.1007/s00439-022-02481-9 34967936 PMC8716573

[225] Zeng Y, Wei Z, Yuan Q et al. (2023) Identifying b-cell epitopes using alphafold2 predicted structures and pretrained language model. *Bioinformatics*, 39, btad187. doi: 10.1093/bioinformatics/btad187 PMC10126322 37039829

[226] Zhou G, Chen W. (2022) Protein functional family classification based on multilevel feature information. In *2022 IEEE International Conference on Bioinformatics and Biomedicine (BIBM)*. IEEE, pp1836–1839.

[227] Mohammed Yusuf S, Zhang F, Zeng M, et al. (2021) Deepppf: a deep learning framework for predicting protein family. *Neurocomputing*, 428, 19–29. doi: 10.1016/j.neucom.2020.11.062

[228] Quoc Khanh Le N, Huynh T-T (2019) Identifying snares by incorporating deep learning architecture and amino acid embedding representation. *Frontiers in Physiology*, 10, 1–8. doi: 10.3389/fphys.2019.01501 31920706 PMC6914855

[229] Wang H, Zheng H, Chen DZ (2022) Tango: a go-term embedding based method for protein semantic similarity prediction. *IEEE/ACM Transactions on Computational Biology and Bioinformatics*, 20, 694–706. doi: 10.1109/TCBB.2022.3143480 35030084

[230] Weihua L, Liu W, Guo Y et al. (2023) Deep contextual representation learning for identifying essential proteins via integrating multisource protein features. *Chinese Journal of Electronics*, 32, 868–881. doi: 10.23919/cje.2022.00.053

[231] Pengli L, Yang P, Liao Y (2023) Deep learning framework for predicting essential proteins with temporal convolutional networks. *Journal of Shanghai Jiaotong University (Science)*, 28, 1–11. doi: 10.1007/s12204-023-2632-9

[232] Yue Y, Chen Y, Peng P-Y et al. (2022) A deep learning framework for identifying essential proteins based on multiple biological information. *BMC bioinformatics*, 23, 318. doi: 10.1186/s12859-022-04868-8 PMC9351218 35927611

[233] Wang N, Zeng M, Zhang J et al. (2020) Ess-nexg: predict essential proteins by constructing a weighted protein interaction network based on node embedding and xgboost. In *Bioinformatics Research and Applications: 16th International Symposium, ISBRA 2020*. Springer, *Moscow, Russia, December 1–4, 2020, Proceedings, 16*. pp95–104.

[234] Wang N, Zeng M, Yiming L et al. (2021) Essential protein prediction based on node2vec and xgboost. *Journal of Computational Biology*, 28, 687–700. doi: 10.1089/cmb.2020.0543 34152838

[235] Zeng M, Li M, Fei Z et al. (2019) A deep learning framework for identifying essential proteins by integrating multiple types of biological information. *IEEE/ACM Transactions on Computational Biology and bioinformatics*, 18, 296–305. doi: 10.1109/TCBB.2019.2897679 30736002

[236] Zeng M, Li M, Fang-Xiang W et al. (2019) Deepep: a deep learning framework for identifying essential proteins. *BMC bioinformatics*, 20, 1–10. doi: 10.1186/s12859-019-3076-y 31787076 PMC6886168

[237] Zeng M, Li M, Fei Z et al. (2018) A deep learning framework for identifying essential proteins based on protein–protein interaction network and gene expression data. In *2018 IEEE International Conference on Bioinformatics and Biomedicine (BIBM)*. IEEE, pp583–588.

[238] Liu C-M, Van-Dai T, Quoc Khanh Le N et al. (2022) Deep neural network framework based on word embedding for protein glutarylation sites prediction. *Life*, 12, 1213. doi: 10.3390/life12081213 PMC9410500 36013392

[239] Duyen Thi D, Quynh Trang Le T, Quoc Khanh Le N (2021) Using deep neural networks and biological subwords to detect protein s-sulfenylation sites. *Briefings in Bioinformatics*, 22, bbaa128. doi: 10.1093/bib/bbaa128 32613242

[240] Albu A-I, Bocicor M-I, Czibula G (2023) Mm-stackens: a new deep multimodal stacked generalization approach for protein–protein interaction prediction. *Computers in Biology and Medicine*, 153, 1–21. doi: 10.1016/j.compbiomed.2022.106526 36623437

[241] Xiao-Rui S, Lun H, You Z-H et al. (2022) Multi-view heterogeneous molecular network representation learning for protein–protein interaction prediction. *BMC bioinformatics*, 23, 234. doi: 10.1186/s12859-022-04766-z PMC9205098 35710342

[242] Pan J, You Z-H, Li-Ping L et al Dwppi: a deep learning approach for predicting protein–protein interactions in plants based on multi-source information with a large-scale biological network. *Frontiers in Bioengineering and Biotechnology*, 10, 2022. doi: 10.3389/fbioe.2022.807522 PMC8978800 35387292

[243] Nabeel Asim M, Ali Ibrahim M, Imran Malik M et al. (2022) Adh-ppi: an attention-based deep hybrid model for protein–protein interaction prediction. *Iscience*, 25, 1–28. doi: 10.1016/j.isci.2022.105169 PMC9576568 36267921

[244] Xiao-Rui S, You Z-H, Lun H et al. (2021) An efficient computational model for large-scale prediction of protein–protein interactions based on accurate and scalable graph embedding. *Frontiers in genetics*, 12, 1–10. doi: 10.3389/fgene.2021.635451 PMC7953052 33719344

[245] Zhang J, Zhu M, Qian Y (2020) protein 2vec: predicting protein–protein interactions based on lstm. *IEEE/ACM Transactions on Computational Biology and Bioinformatics*, 19, 1257–1266. doi: 10.1109/TCBB.2020.3003941 32750870

[246] Zhong X, Rajapakse JC (2020) Graph embeddings on gene ontology annotations for protein–protein interaction prediction. *BMC bioinformatics*, 21, 1–17. doi: 10.1186/s12859-020-03816-8 33323115 PMC7739483

[247] Zhou P, Zhang Y, Zeqian L et al. (2023) Protein complex identification based on heterogeneous protein information network. *Journal of Computational Biology*, 30, 985–998. doi: 10.1089/cmb.2023.0081 37669441

[248] Wang R, Huimin M, Wang C (2022) An ensemble learning framework for detecting protein complexes from ppi networks. *Frontiers in Genetics*, 13, 1–28. doi: 10.3389/fgene.2022.839949 PMC8908451 35281831

[249] Meng X, Xiang J, Zheng R et al. (2021) Dpcmne: detecting protein complexes from protein–protein interaction networks via multi-level network embedding. *IEEE/ACM Transactions on Computational Biology and Bioinformatics*, 19, 1592–1602. doi: 10.1109/TCBB.2021.3050102 33417563

[250] Zhu J, Zheng Z, Yang M et al. (2019) Protein complexes detection based on semi-supervised network embedding model. *IEEE/ACM Transactions on Computational Biology and Bioinformatics*, 18, 797–803. doi: 10.1109/TCBB.2019.2944809 31581089

[251] Yao H, Shi Y, Guan J, et al. (2019) Accurately detecting protein complexes by graph embedding and combining functions with interactions. *IEEE/ACM Transactions on Computational Biology and Bioinformatics*, 17, 777–787. doi: 10.1109/TCBB.2019.2897769 30736004

[252] Hong Z, Liu J, Chen Y (2021) An interpretable machine learning method for homo-trimeric protein interface residue-residue interaction prediction. *Biophysical Chemistry*, 278, 1–7. doi: 10.1016/j.bpc.2021.106666 34418678

[253] Yang S, Liu X, Raymond TN (2020) Proberating: a recommender system to infer binding profiles for nucleic acid-binding proteins. *Bioinformatics*, 36, 4797–4804. doi: 10.1093/bioinformatics/btaa580 32573679 PMC7750938

[254] Hui L, Bin W, Sun M et al. (2024) Cross-domain contrastive graph neural network for lncrna-protein interaction prediction. *Knowledge-Based Systems*, 296, 111901. doi: 10.1016/j.knosys.2024.111901

[255] Han Y, Zhang S-W (2023) ncrpi-lgat: prediction of ncrna-protein interactions with line graph attention network framework. *Computational and Structural Biotechnology Journal*, 21, 2286–2295. doi: 10.1016/j.csbj.2023.03.027 37035546 PMC10073990

[256] Wei M-M, Chang-Qing Y, Li-Ping L et al. (2023) Lpih2v: Lncrna-protein interactions prediction using hin2vec based on heterogeneous networks model. *Frontiers in Genetics*, 14, 1–10. doi: 10.3389/fgene.2023.1122909 PMC9950107 36845392

[257] Zhao J, Sun J, Shuai SC et al. (2023) Predicting potential interactions between lncrnas and proteins via combined graph auto-encoder methods. *Briefings in Bioinformatics*, 24, bbac527. doi: 10.1093/bib/bbac527 36515153

[258] Shen Z-A, Luo T, Zhou Y-K et al. (2021) Npi-gnn: predicting ncrna–protein interactions with deep graph neural networks. *Briefings in bioinformatics*, 22, bbab051. doi: 10.1093/bib/bbab051 33822882

[259] Hai-Cheng Y, You Z-H, Guo Z-H et al. (2020) Learning representation of molecules in association network for predicting intermolecular associations. *IEEE/ACM Transactions on Computational Biology and Bioinformatics*, 18, 2546–2554. doi: 10.1109/TCBB.2020.2973091 32070992

[260] Palhamkhani F, Alipour M, Dehnad A et al. (2023) Deepcompoundnet: enhancing compound–protein interaction prediction with multimodal convolutional neural networks. *Journal of Biomolecular Structure and Dynamics*, 43, 1–10. doi: 10.1080/07391102.2023.2291829 38084744

[261] Chen Z-H, Zhao B-W, Jian-Qiang L et al. (2023) Graphcpis: a novel graph-based computational model for potential compound-protein interactions. *Molecular Therapy-Nucleic Acids*, 32, 721–728. doi: 10.1016/j.omtn.2023.04.030 37251691 PMC10209012

[262] Wang H, Zhu H, Wenhao L et al. (2022) Predicting compound-protein interaction by deepening the systemic background via molecular network feature embedding. In *2022 IEEE International Conference on Bioinformatics and Biomedicine (BIBM)*. IEEE, pp346–353.

[263] Watanabe N, Ohnuki Y, Sakakibara Y (2021) Deep learning integration of molecular and interactome data for protein–compound interaction prediction. *Journal of Cheminformatics*, 13, 36. doi: 10.1186/s13321-021-00513-3 PMC8088618 33933121

[264] Pan J, You W, Xiaoliang L et al. (2023) Gsphi: a novel deep learning model for predicting phage-host interactions via multiple biological information. *Computational and Structural Biotechnology Journal*, 21, 3404–3413. doi: 10.1016/j.csbj.2023.06.014 37397626 PMC10314231

[265] Golzadeh A, Kamandi A, Rahami H (2023) An attributed network embedding method to predict missing links in protein–protein interaction networks. *Journal of Algorithms and Computation*, 55, 79–99. doi: 10.22059/jac.2023.92758

[266] Balogh OM, Benczik B, Horváth A et al. (2022) Efficient link prediction in the protein–protein interaction network using topological information in a generative adversarial network machine learning model. *BMC bioinformatics*, 23, 78. doi: 10.1186/s12859-022-04598-x PMC8858570 35183129

[267] Patel R, Guo Y, Alhudhaif A et al. (2022) Graph-based link prediction between human phenotypes and genes. *Mathematical Problems in Engineering*, 2022, 7111647. doi: 10.1155/2022/7111647

[268] Nasiri E, Berahmand K, Rostami M, et al. (2021) A novel link prediction algorithm for protein–protein interaction networks by attributed graph embedding. *Computers in Biology and Medicine*, 137, 1–11. doi: 10.1016/j.compbiomed.2021.104772 34450380

[269] Feng J, Zeng A, Chen Y et al. (2020) Signaling interaction link prediction using deep graph neural networks integrating protein–protein interactions and omics data. *BioRxiv*, 2020–12.

[270] Mallick K, Bandyopadhyay S, Chakraborty S et al. (2019) Topo2vec: a novel node embedding generation based on network topology for link prediction. *IEEE Transactions on Computational Social Systems*, 6, 1306–1317. doi: 10.1109/TCSS.2019.2950589

[271] Fan H, Jiang J, Yin P (2022) Prediction of potential commercially available inhibitors against sars-cov-2 by multi-task deep learning model. *Biomolecules*, 12, 1156. doi: 10.3390/biom12081156 PMC9405964 36009050

[272] Xuan P, Zhang X, Zhang Y et al. (2022) Multi-type neighbors enhanced global topology and pairwise attribute learning for drug–protein interaction prediction. *Briefings in bioinformatics*, 23, bbac120. doi: 10.1093/bib/bbac120 35514190

[273] Chen W, Chen G, Zhao L, et al. (2021) Predicting drug–target interactions with deep-embedding learning of graphs and sequences. *The Journal of Physical Chemistry A*, 125, 5633–5642. doi: 10.1021/acs.jpca.1c02419 34142824

[274] Wang Z, Yaowen G, Zheng S et al. (2023) Mgrel: a multi-graph representation learning-based ensemble learning method for gene-disease association prediction. *Computers in Biology and Medicine*, 155, 1–11. doi: 10.1016/j.compbiomed.2023.106642 36805231

[275] Chu X, Guan B, Dai L et al. (2023) Network embedding framework for driver gene discovery by combining functional and structural information. *BMC genomics*, 24, 426. doi: 10.1186/s12864-023-09515-x PMC10386255 37516822

[276] Vilela J, Asif M, Rita Marques A et al. (2023) Biomedical knowledge graph embeddings for personalized medicine: predicting disease-gene associations. *Expert Systems*, 40, e13181. doi: 10.1111/exsy.13181

[277] Ratajczak F, Joblin M, Hildebrandt M et al. (2023) Speos: an ensemble graph representation learning framework to predict core gene candidates for complex diseases. *Nature Communications*, 14, 7206. doi: 10.1038/s41467-023-42975-z PMC10632370 37938585

[278] Jagodnik KM, Shvili Y, Bartal A (2023) Hetig-predig: a heterogeneous integrated graph model for predicting human disease genes based on gene expression. *Plos one*, 18, e0280839. doi: 10.1371/journal.pone.0280839 PMC9931161 36791052

[279] Zhang L, Dianrong L, Xuehua B et al. (2023) Predicting disease genes based on multi-head attention fusion. *BMC bioinformatics*, 24, 162. doi: 10.1186/s12859-023-05285-1 PMC10122338 37085750

[280] Wang L, Mingxiao W, Yulin W et al. (2022) Prediction of the disease causal genes based on heterogeneous network and multi-feature combination method. *Computational Biology and Chemistry*, 97, 1–9. doi: 10.1016/j.compbiolchem.2022.107639 35217251

[281] Jian L, JiaRui L, Ren J et al. (2022) Functional and embedding feature analysis for pan-cancer classification. *Frontiers in Oncology*, 12, 1–15. doi: 10.3389/fonc.2022.979336 PMC9559388 36248961

[282] Wang T, Hengbo X, Zhang R et al. (2022) Hypergraph-based gene ontology embedding for disease gene prediction. In *2022 IEEE International Conference on Bioinformatics and Biomedicine (BIBM)*. IEEE, pp2424–2430.

[283] Prabhakar V, Liu K (2022) Unsupervised co-optimization of a graph neural network and a knowledge graph embedding model to prioritize causal genes for alzheimer’s disease. *medRxiv*, 2022–10.

[284] Wang T, Shao Z, Xiao Y et al. (2021) Predicting hepatoma-related genes based on representation learning of ppi network and gene ontology annotations. In *2021 IEEE International Conference on Bioinformatics and Biomedicine (BIBM)*. IEEE, pp1892–1898.

[285] Liu H, Hou L, Xu S et al. (2021) Discovering cerebral ischemic stroke associated genes based on network representation learning. *Front Genet*, 12, 728333. doi: 10.3389/fgene.2021.728333 PMC8442767 34539754

[286] Jianzong D, Lin D, Yuan R et al. (2021) Graph embedding based novel gene discovery associated with diabetes mellitus. *Frontiers in Genetics*, 12, 1–11. doi: 10.3389/fgene.2021.779186 PMC8657768 34899863

[287] Fang X, Guo G, Zhu F et al. (2021) Protein deep profile and model predictions for identifying the causal genes of male infertility based on deep learning. *Information Fusion*, 75, 70–89. doi: 10.1016/j.inffus.2021.04.012

[288] Liu H, Guan J, Li H et al. (2020) Predicting the disease genes of multiple sclerosis based on network representation learning. *Frontiers in Genetics*, 11, 1–7. doi: 10.3389/fgene.2020.00328 32373160 PMC7186413

[289] Madeddu L, Stilo G, Velardi P (2019) Network-based methods for disease-gene prediction. *ArXiv Preprint arXiv:1902.10117*.

[290] Peng J, Guan J, Shang X (2019) Predicting parkinson’s disease genes based on node2vec and autoencoder. *Frontiers in genetics*, 10, 1–6. doi: 10.3389/fgene.2019.00226 31001311 PMC6454041

[291] Luo P, Yuanyuan L, Tian L-P, et al. (2019) Enhancing the prediction of disease–gene associations with multimodal deep learning. *Bioinformatics*, 35, 3735–3742. doi: 10.1093/bioinformatics/btz155 30825303

[292] Zhu L, Hong Z, Zheng H. (2019) Predicting gene-disease associations via graph embedding and graph convolutional networks. In *2019 IEEE International Conference on Bioinformatics and Biomedicine (BIBM)*. IEEE, pp382–389.

[293] Yang K, Wang R, Liu G et al. (2018) Hergepred: heterogeneous network embedding representation for disease gene prediction. *IEEE Journal of Biomedical and Health informatics*, 23, 1805–1815. doi: 10.1109/JBHI.2018.2870728 31283472

[294] Kircali Ata S, Ou-Yang L, Fang Y et al. (2018) Integrating node embeddings and biological annotations for genes to predict disease-gene associations. *BMC Systems biology*, 12, 31–44. doi: 10.1186/s12918-018-0662-y 30598097 PMC6311944

[295] İbrahim Kuru H, İlkağan Tepeli Y, Taştan O (2022) Gege: predicting gene essentiality with graph embeddings. *Düzce ÜNiversitesi Bilim ve Teknoloji Dergisi*, 10, 1567–1577. doi: 10.29130/dubited.1028387

[296] Dai W, Chang Q, Peng W et al. (2020) Network embedding the protein–protein interaction network for human essential genes identification. *Genes*, 11, 153. doi: 10.3390/genes11020153 PMC7074227 32023848

[297] Zhang X, Guo H, Zhang F et al. (2023) Hnetgo: protein function prediction via heterogeneous network transformer. *Briefings in Bioinformatics*, 24, bbab556. doi: 10.1093/bib/bbab556 PMC10588005 37861172

[298] Ali S, Chourasia P, Patterson M (2023) When protein structure embedding meets large language models. *Genes*, 15, 25. doi: 10.3390/genes15010025 PMC10815811 38254915

[299] Kaiyi W, Zhou D, Slonim D et al. (2023) Melissa: semi-supervised embedding for protein function prediction across multiple networks. *bioRxiv*, 2023–08.

[300] Hao L, Zhang SQ, Chen L et al. (2022) Identifying functions of proteins in mice with functional embedding features. *Frontiers in Genetics*, 13, 1–12. doi: 10.3389/fgene.2022.909040 PMC9149260 35651937

[301] Tseng W-C, Chi P-H, Jia-Hua W, et al. (2021) Leveraging sequence embedding and convolutional neural network for protein function prediction. *ArXiv Preprint arXiv:2112.00344*.

[302] Sharma VS, Fossati A, Ciuffa R, et al. (2021) Towards a systematic characterization of protein complex function: a natural language processing and machine-learning framework. *bioRxiv*, 2021–02.

[303] Zhang F, Song H, Zeng M et al. (2020) A deep learning framework for gene ontology annotations with sequence-and network-based information. *IEEE/ACM Transactions on Computational Biology and bioinformatics*, 18, 2208–2217. doi: 10.1109/TCBB.2020.2968882 31985440

[304] Wan C, Cozzetto D, Rui F, et al. (2019) Using deep maxout neural networks to improve the accuracy of function prediction from protein interaction networks. *PloS one*, 14, e0209958. doi: 10.1371/journal.pone.0209958 PMC6650051 31335894

[305] Sarker B, Ritchie DW, Aridhi S. (2019) Functional annotation of proteins using domain embedding based sequence classification. In *KDIR 2019-11th International Conference on Knowledge Discovery and Information Retrieval*. SCITEPRESS-Science and Technology Publications, pp163–170.

[306] Hou Z, Yang Y, Hui L et al. (2021) ideepsubmito: identification of protein submitochondrial localization with deep learning. *Briefings in Bioinformatics*, 22, bbab288. doi: 10.1093/bib/bbab288 34337657

[307] Hayat M, Tahir M, Khaled Alarfaj F et al. (2022) Nlp-bch-ens: Nlp-based intelligent computational model for discrimination of malaria parasite. *Computers in Biology and Medicine*, 149, 1–8. doi: 10.1016/j.compbiomed.2022.105962 36049412

[308] Nguyen T-T-D, Nguyen-Quoc-Khanh L, Quang-Thai H et al. (2020) Tnfpred: identifying tumor necrosis factors using hybrid features based on word embeddings. *BMC Medical Genomics*, 13, 1–11. doi: 10.1186/s12920-020-00779-w 33087125 PMC7579990

[309] Adjuik TA, Ananey-Obiri D (2022) Word2vec neural model-based technique to generate protein vectors for combating covid-19: a machine learning approach. *International Journal of Information Technology*, 14, 3291–3299. doi: 10.1007/s41870-022-00949-2 35611155 PMC9119569

[310] Chao C-T, Tsai Y-T, Lee W-T et al. (2022) Deep learning-assisted repurposing of plant compounds for treating vascular calcification: an in silico study with experimental validation. *Oxidative Medicine and Cellular Longevity*, 2022, 4378413. doi: 10.1155/2022/4378413 PMC8754599 35035662

[311] Ostrovsky-Berman M, Frankel B, Polak P, et al. (2021) Immune2vec: embedding b/t cell receptor sequences in $\mathbb{R}^\mathrm{n}$ using natural language processing. *Frontiers in immunology*, 12, 1–13. doi: 10.3389/fimmu.2021.680687 PMC8340020 34367141

[312] Idhaya T, Suruliandi A, Raja SP (2023) Stacked framework of machine learning classifiers for protein family prediction using protein characteristics. *CURRENT SCIENCE*, 125, 508. doi: 10.18520/cs/v125/i5/508-517

[313] Saha S, Chatterjee P, Basu S, et al. (2024) Epi-sf: essential protein identification in protein interaction networks using sequence features. *PeerJ*, 12, e17010. doi: 10.7717/peerj.17010 PMC10944162 38495766

[314] Chen Y, Wu Q, Chen S et al. (2024) Ecdep: identifying essential proteins based on evolutionary community discovery and subcellular localization. *BMC genomics*, 25, 117. doi: 10.1186/s12864-024-10019-5 PMC10821549 38279081

[315] (2023) Md Inzamam-Ul-Hossain and Md Rafiqul Islam. Identification of essential protein using chemical reaction optimization and machine learning technique. *IEEE/ACM Transactions on Computational Biology and Bioinformatics*. 10.1109/TCBB.2022.3233473 37018299

[316] Md Inzamam Ul Hossain and Md Rafiqul Islam (2023) *Efficiency due to Data Balancing in the Prediction of Essential proteins*.

[317] Zhang H, Feng Z, Chong W. (2022) A non-local graph neural network for identification of essential proteins. In *2022 International Joint Conference on Neural Networks (IJCNN)*. IEEE, pp1–8.

[318] Zeng M, Wang N, Yifan W et al. (2021) Improving human essential protein prediction using only protein sequences via ensemble learning. In *2021 IEEE International Conference on Bioinformatics and Biomedicine (BIBM)*. IEEE, pp98–103.

[319] Golzadeh Kermani A, Kamandi A, Moeini A (2022) Integrating graph structure information and node attributes to predict protein–protein interactions. *Journal of Computational Science*, 64, 1–10.

[320] Wang Y, Ding P, Wang C et al. (2024) Rpi-ggcn: Prediction of RNA–protein interaction based on interpretability gated graph convolution neural network and co-regularized variational autoencoders. *IEEE Transactions on Neural Networks and Learning Systems*. 10.1109/TNNLS.2024.3390935 38709606

[321] Weian D, Zhao L, Rong W et al. (2024) Predicting drug–protein interaction with deep learning framework for molecular graphs and sequences: Potential candidates against sar-cov-2. *Plos one*, 19, e0299696. doi: 10.1371/journal.pone.0299696 PMC11086825 38728335

[322] Han B, Zhao N, Zeng C et al. (2022) Acpred-bmf: bidirectional lstm with multiple feature representations for explainable anticancer peptide prediction. *Scientific Reports*, 12, 21915. doi: 10.1038/s41598-022-24404-1 PMC9763336 36535969

[323] Akbar S, Hayat M, Iqbal M, et al. (2017) iacp-gaensc: evolutionary genetic algorithm based ensemble classification of anticancer peptides by utilizing hybrid feature space. *Artificial Intelligence in Medicine*, 79, 62–70. doi: 10.1016/j.artmed.2017.06.008 28655440

[324] Deng H, Ding M, Wang Y et al. (2023) Acp-mlc: a two-level prediction engine for identification of anticancer peptides and multi-label classification of their functional types. *Computers in Biology and Medicine*, 158, 1–14. doi: 10.1016/j.compbiomed.2023.106844 37058760

[325] Wang H, Zhao J, Zhao H et al. (2021) Cl-acp: a parallel combination of cnn and lstm anticancer peptide recognition model. *BMC bioinformatics*, 22, 1–22. doi: 10.1186/s12859-021-04433-9 34670488 PMC8527680

[326] Garai S, Thomas J, Dey P, et al. (2023) Lgbm-acp: an ensemble model for anticancer peptide prediction and in silico screening with potential drug targets. *Molecular Diversity*, 28, 1–17. doi: 10.1007/s11030-023-10602-0 36637711

[327] Yao L, Wenshuo L, Zhang Y et al. (2023) Ying-Chih Chiang, and Tzong-Yi Lee. Accelerating the discovery of anticancer peptides through deep forest architecture with deep graphical representation. *International Journal of Molecular Sciences*, 24, 4328. doi: 10.3390/ijms24054328 PMC10001941 36901759

[328] Jing X, Fuyi L, Chen L, et al. (2023) iampcn: a deep-learning approach for identifying antimicrobial peptides and their functional activities. *Briefings in Bioinformatics*, 24, bbad240. doi: 10.1093/bib/bbad240 PMC10359087 37369638

[329] Xiao X, Shao Y-T, Cheng X, et al. (2021) iamp-ca2l: a new cnn-bilstm-svm classifier based on cellular automata image for identifying antimicrobial peptides and their functional types. *Briefings in bioinformatics*, 22, bbab209. doi: 10.1093/bib/bbab209 34086856

[330] Lin Y, Cai Y, Liu J et al. (2019) An advanced approach to identify antimicrobial peptides and their function types for penaeus through machine learning strategies. *BMC bioinformatics*, 20, 1–10. doi: 10.1186/s12859-019-2766-9 31182007 PMC6557738

[331] Olcay B, Ozdemir GD, Ozdemir MA et al. (2024) Prediction of the synergistic effect of antimicrobial peptides and antimicrobial agents via supervised machine learning. *BMC Biomedical Engineering*, 6, 1. doi: 10.1186/s42490-024-00075-z PMC10792927 38233957

[332] Teimouri H, Medvedeva A, Kolomeisky AB (2023) Bacteria-specific feature selection for enhanced antimicrobial peptide activity predictions using machine-learning methods. *Journal of Chemical Information and Modeling*, 63, 1723–1733. doi: 10.1021/acs.jcim.2c01551 36912047

[333] Wang Z, Meng J, Haibin L et al. (2023) Pampred: a hierarchical evolutionary ensemble framework for identifying plant antimicrobial peptides. *Computers in Biology and Medicine*, 166, 1–11. doi: 10.1016/j.compbiomed.2023.107545 37806057

[334] Jaiswal M, Singh A, Kumar S (2023) Ptpamp: prediction tool for plant-derived antimicrobial peptides. *Amino Acids*, 55, 1–17. doi: 10.1007/s00726-022-03190-0 35864258

[335] Lin W, Dong X (2016) Imbalanced multi-label learning for identifying antimicrobial peptides and their functional types. *Bioinformatics*, 32, 3745–3752. doi: 10.1093/bioinformatics/btw560 27565585 PMC5167070

[336] Dumitrescu A, Jokinen E, Paatero A et al. (2023) Tsignal: a transformer model for signal peptide prediction. *Bioinformatics*, 39, i347–i356. doi: 10.1093/bioinformatics/btad228 37387131 PMC10311348

[337] Youmans M, Spainhour JCG, Qiu P (2019) Classification of antibacterial peptides using long short-term memory recurrent neural networks. *IEEE/ACM Transactions on Computational Biology and Bioinformatics*, 17, 1134–1140. doi: 10.1109/TCBB.2019.2903800 30843849

[338] Gaffar S, Tanveerul Hassan M, Tayara H, et al. (2024) If-aip: a machine learning method for the identification of anti-inflammatory peptides using multi-feature fusion strategy. *Computers in Biology and Medicine*, 168, 1–8. doi: 10.1016/j.compbiomed.2023.107724 37989075

[339] Zhang J, Zhang Z, Lianrong P et al. (2020) Aiepred: an ensemble predictive model of classifier chain to identify anti-inflammatory peptides. *IEEE/ACM Transactions on Computational Biology and bioinformatics*, 18, 1831–1840. doi: 10.1109/TCBB.2020.2968419 31985437

[340] Gupta S, Sharma AK, Shastri V et al. (2017) Prediction of anti-inflammatory proteins/peptides: an insilico approach. *Journal of Translational medicine*, 15, 1–11. doi: 10.1186/s12967-016-1103-6 28057002 PMC5216551

[341] Shamima Khatun M, Mehedi Hasan M, Kurata H (2019) Preaip: computational prediction of anti-inflammatory peptides by integrating multiple complementary features. *Frontiers in genetics*, 10, 1–11. doi: 10.3389/fgene.2019.00129 30891059 PMC6411759

[342] Deng H, Lou C, Zengrui W et al. (2022) Prediction of anti-inflammatory peptides by a sequence-based stacking ensemble model named aipstack. *Iscience*, 25, 1–20. doi: 10.1016/j.isci.2022.104967 PMC9449674 36093066

[343] Sun C, Tang R, Huang J et al. (2023) A deep neural network-based co-coding method to predict drug-protein interactions by analyzing the feature consistency between drugs and proteins. *IEEE/ACM Transactions on Computational Biology and Bioinformatics*. 10.1109/TCBB.2023.3237863 37021862

[344] Wang K, Min L. (2023) Fusion-based deep learning architecture for detecting drug-target binding affinity using target and drug sequence and structure. *IEEE Journal of Biomedical and Health Informatics*. 10.1109/JBHI.2023.3315073 37703165

[345] Zhu Y, Zhao L, Wen N et al. (2023) Datadta: a multi-feature and dual-interaction aggregation framework for drug–target binding affinity prediction. *Bioinformatics*, 39, btad560. doi: 10.1093/bioinformatics/btad560 PMC10516524 37688568

[346] Wang X, Yang K, Jia T et al. (2024) Kdgene: knowledge graph completion for disease gene prediction using interactional tensor decomposition. *Briefings in Bioinformatics*, 25, bbae161. doi: 10.1093/bib/bbae161 PMC11009469 38605639

[347] Gao Z, Pan Y, Ding P, et al. (2022) A knowledge graph-based disease-gene prediction system using multi-relational graph convolution networks. In *AMIA Annual Symposium Proceedings*. American Medical Informatics Association, p468. Vol. 2022. PMC10148306 37128437

[348] Mehmood F, Arshad S, Shoaib M (2023) Rppsp: a robust and precise protein solubility predictor by utilizing novel protein sequence encoder. *IEEE Access*, 11, 59397–59416. doi: 10.1109/ACCESS.2023.3284464

[349] Islam R, Talukdar MD, Rafid S et al. (2024) Deep multi-modal approach for protein function prediction and classification. In *2024 6th International Conference on Electrical Engineering and Information & Communication Technology (ICEEICT)*. IEEE, pp376–381.

[350] Routray M, Vipsita S, Sundaray A, et al. (2022) Deeprhd: an efficient hybrid feature extraction technique for protein remote homology detection using deep learning strategies. *Computational Biology and Chemistry*, 100, 1–9. doi: 10.1016/j.compbiolchem.2022.107749 35970053

[351] Ruan X, Liu K, Yang J et al. (2024) Ensemble learning method for predicting protein submitochondrial localization by multi-type feature fusion.

[352] Hajisharifi Z, Piryaiee M, Mohammad Beigi M et al. (2014) Predicting anticancer peptides with Chou’s pseudo amino acid composition and investigating their mutagenicity via AMES test. *Journal of Theoretical biology*, 341, 34–40. doi: 10.1016/j.jtbi.2013.08.037 24035842

[353] Wei L, Zhou C, Chen H et al. (2018) Acpred-fl: a sequence-based predictor using effective feature representation to improve the prediction of anti-cancer peptides. *Bioinformatics*, 34, 4007–4016. doi: 10.1093/bioinformatics/bty451 29868903 PMC6247924

[354] Chen W, Ding H, Feng P et al. (2016) iacp: a sequence-based tool for identifying anticancer peptides. *Oncotarget*, 7, 16895. doi: 10.1016/j.compbiomed.2024.108063 PMC4941358 26942877

[355] Wenjia H, Wang Y, Cui L et al. (2021) Learning embedding features based on multisense-scaled attention architecture to improve the predictive performance of anticancer peptides. *Bioinformatics*, 37, 4684–4693. doi: 10.1093/bioinformatics/btab560 34323948

[356] Chung C-R, Kuo T-R, Li-Ching W et al. (2020) Characterization and identification of antimicrobial peptides with different functional activities. *Briefings in bioinformatics*, 21, 1098–1114. doi: 10.1093/bib/bbz043 31155657

[357] Brendan Timmons P, Hewage CM (2021) Ennaact is a novel tool which employs neural networks for anticancer activity classification for therapeutic peptides. *Biomedicine & Pharmacotherapy*, 133, 1–11. doi: 10.1016/j.biopha.2020.111051 33254015

[358] Youmans M, Spainhour C, Qiu P. (2017) Long short-term memory recurrent neural networks for antibacterial peptide identification. In *2017 IEEE International Conference on Bioinformatics and Biomedicine (BIBM)*. IEEE, pp498–502.

[359] Singh V, Shrivastava S, Kumar Singh S et al. (2022) Stable-abppred: a stacked ensemble predictor based on bilstm and attention mechanism for accelerated discovery of antibacterial peptides. *Briefings in Bioinformatics*, 23, bbab439. doi: 10.1093/bib/bbab439 34750606

[360] Khaledian E, Broschat SL. Sequence-based discovery of antibacterial peptides using ensemble gradient boosting. In *Proceedings*. p6 MDPI, 2020. Vol. 66.

[361] Jan A, Hayat M, Wedyan M et al. (2022) Target-amp: computational prediction of antimicrobial peptides by coupling sequential information with evolutionary profile. *Computers in Biology and Medicine*, 151, 1–6. doi: 10.1016/j.compbiomed.2022.106311 36410097

[362] Ahmad Wani M, Garg P, Roy KK (2021) Machine learning-enabled predictive modeling to precisely identify the antimicrobial peptides. *Medical & Biological Engineering & Computing*, 59, 2397–2408. doi: 10.1007/s11517-021-02443-6 34632545

[363] Gülsüm Söylemez U, Yousef M, Kesmen Z et al. (2022) Prediction of linear cationic antimicrobial peptides active against gram-negative and gram-positive bacteria based on machine learning models. *Applied Sciences*, 12, 3631. doi: 10.3390/app12073631

[364] Sharma R, Shrivastava S, Kumar Singh S et al. (2021) Aniamppred: artificial intelligence guided discovery of novel antimicrobial peptides in animal kingdom. *Briefings in Bioinformatics*, 22, bbab242. doi: 10.1093/bib/bbac631 34259329

[365] Kavousi K, Bagheri M, Behrouzi S et al. (2020) Iampe: Nmr-assisted computational prediction of antimicrobial peptides. *Journal of Chemical Information and Modeling*, 60, 4691–4701. doi: 10.1021/acs.jcim.0c00841 32946226

[366] Wang Y, Wang L, Chengquan L et al. (2023) Amp-ebilstm: employing novel deep learning strategies for the accurate prediction of antimicrobial peptides. *Frontiers in Genetics*, 14, 1–14. doi: 10.3389/fgene.2023.1232117 PMC10405519 37554402

[367] Dong G-F, Zheng L, Huang S-H et al. (2021) Amino acid reduction can help to improve the identification of antimicrobial peptides and their functional activities. *Frontiers in Genetics*, 12, 1–11. doi: 10.3389/fgene.2021.669328 PMC8093877 33959153

[368] Qinze Y, Dong Z, Fan X et al. (2021) Hmd-amp: protein language-powered hierarchical multi-label deep forest for annotating antimicrobial peptides. *ArXiv Preprint arXiv:2111.06023*.

[369] Gull S, Shamim N, Minhas F (2019) Amap: hierarchical multi-label prediction of biologically active and antimicrobial peptides. *Computers in Biology and medicine*, 107, 172–181. doi: 10.1016/j.compbiomed.2019.02.018 30831306

[370] AlmagroArmenteros JJ, Tsirigos KD, KaaeSønderby C et al. (2019) Signalp 5.0 improves signal peptide predictions using deep neural networks. *Nature biotechnology*, 37, 420–423. doi: 10.1038/s41587-019-0036-z 30778233

[371] Savojardo C, Luigi Martelli P, Fariselli P, et al. (2018) Deepsig: deep learning improves signal peptide detection in proteins. *Bioinformatics*, 34, 1690–1696. doi: 10.1093/bioinformatics/btx818 29280997 PMC5946842

[372] Heng Choo K , Wee Tan T, and Ranganathan S. A comprehensive assessment of n-terminal signal peptides prediction methods. In *Bmc Bioinformatics*, 10, 1–12. Springer, 2009. 19958512 10.1186/1471-2105-10-S15-S2 PMC2788353

[373] Zeng S, Wang D, Dong X (2023) Peft-sp: parameter-efficient fine-tuning on large protein language models improves signal peptide prediction. *bioRxiv*, 2023–11. 10.1101/gr.279132.124 PMC11529868 39060029

[374] Zhang W-X, Pan X, Shen H-B (2020) Signal-3l 3.0: improving signal peptide prediction through combining attention deep learning with window-based scoring. *Journal of Chemical Information and Modeling*, 60, 3679–3686. doi: 10.1021/acs.jcim.0c00401 32501689

[375] Manavalan B, Shin TH, Kim MO, et al. (2018) Aippred: sequence-based prediction of anti-inflammatory peptides using random forest. *Frontiers in pharmacology*, 9, 1–12. doi: 10.3389/fphar.2018.00276 29636690 PMC5881105

[376] Kaur D, Arora A, Vigneshwar P, et al. (2023) Prediction of peptide hormones using an ensemble of machine learning and similarity-based methods. *bioRxiv*, 2023–05. 10.1002/pmic.202400004 38803012

[377] Hou J, Adhikari B, Cheng J (2018) Deepsf: deep convolutional neural network for mapping protein sequences to folds. *Bioinformatics*, 34, 1295–1303. doi: 10.1093/bioinformatics/btx780 29228193 PMC5905591

[378] Stricker M, Nabeel Asim M, Dengel A, et al. (2022) Circnet: an encoder–decoder-based convolution neural network (CNN) for circular rna identification. *Neural Computing and Applications*, 34, 1–12. doi: 10.1007/s00521-020-05673-134305326

[379] Nabeel Asim M, Imran Malik M, Zehe C et al. (2020) A robust and precise convnet for small non-coding rna classification (rpc-snrc). *IEEE Access*, 9, 19379–19390. doi: 10.1109/ACCESS.2020.3037642

[380] Dosovitskiy A, Beyer L, Kolesnikov A, et al. (2020) An image is worth 16x16 words: transformers for image recognition at scale. *ArXiv Preprint arXiv:2010.11929*.

[381] Raffel C, Shazeer N, Roberts A et al. (2020) Exploring the limits of transfer learning with a unified text-to-text transformer. *Journal of Machine Learning research*, 21, 1–67. doi: 10.5555/3455716.345585634305477

[382] Vaswani A, Shazeer N, Parmar N et al. (2017) 31st Conference on Neural Information Processing Systems (NIPS 2017). Attention is all you need. *Advances in Neural Information Processing systems*, 30. doi: 10.5555/3295222.3295349

[383] Lan Z, Chen M, Goodman S et al. (2019) Albert: a lite bert for self-supervised learning of language representations. *ArXiv Preprint arXiv:1909.11942*.

[384] Devlin J, Chang M-W, Lee K, et al. (2018) Bert: pre-training of deep bidirectional transformers for language understanding. *ArXiv Preprint arXiv:1810.04805*.

[385] Liu Y, Ott M, Goyal N et al. (2019) Roberta: a robustly optimized bert pretraining approach. *ArXiv Preprint arXiv:1907.11692*.

[386] Yang Z, Dai Z, Yang Y et al. (2019) 33rd Conference on Neural Information Processing Systems (NeurIPS 2019). Xlnet: generalized autoregressive pretraining for language understanding. *Advances in Neural Information Processing systems*, 32. doi: 10.5555/3454287.3454804

[387] Radford A, Narasimhan K, Salimans T, et al. (2018) Improving language understanding by generative pre-training.

[388] Radford A, Jeffrey W, Child R, et al. (2019) Language models are unsupervised multitask learners. *OpenAI blog*, 1, 9.

[389] Brown T, Mann B, Ryder N, et al. (2020) Language models are few-shot learners. *Advances in Neural Information Processing systems*, 33, 1877–1901. doi: 10.5555/3495724.3495883

[390] Achiam J, Adler S, Agarwal S, Ahmad L, Akkaya I, Leoni Aleman F, Almeida D, Altenschmidt J, Altman S Anadkat S et al. Gpt-4 technical report. *arXiv preprint arXiv:2303.08774*, 2023.

[391] Ruffolo JA, Chu L-S, Pooja Mahajan S, et al. (2023) Fast, accurate antibody structure prediction from deep learning on massive set of natural antibodies. *Nature communications*, 14, 2389. doi: 10.1038/s41467-023-38063-x PMC10129313 37185622

[392] Lin Z, Akin H, Rao R, et al. (1123–1130, 1123–1130) Evolutionary-scale prediction of atomic-level protein structure with a language model. *Science*, 379, 1123–1130. doi: 10.1126/science.ade2574 36927031

[393] Jumper J, Evans R, Pritzel A, et al. (2021) Highly accurate protein structure prediction with alphafold. *nature*, 596, 583–589. doi: 10.1038/s41586-021-03819-2 34265844 PMC8371605

[394] Rao RM, Liu J, Verkuil R et al. Msa transformer. In *International Conference on Machine Learning*. pp8844–8856 PMLR, 2021.

[395] Ross J, Belgodere B, Chenthamarakshan V (2022) Inkit Padhi, Youssef Mroueh, and Payel Das. Large-scale chemical language representations capture molecular structure and properties. *Nature Machine Intelligence*, 4, 1256–1264. doi: 10.1038/s42256-022-00580-7

[396] Yun S, Jeong M, Kim R et al. (2019) Graph transformer networks. 33rd Conference on Neural Information Processing Systems (NeurIPS 2019). *Advances in Neural Information Processing systems*, 32. doi: 10.48550/arXiv.1911.06455

[397] Liu D, Young F, Robertson DL, et al. (2023) Prediction of virus-host associations using protein language models and multiple instance learning. *bioRxiv*, 2023–04. 10.1371/journal.pcbi.1012597 PMC11614202 39561204

[398] Xiong Z, Liu S, Huang F et al. (2023) Multi-relational contrastive learning graph neural network for drug-drug interaction event prediction. In *Proceedings of the AAAI Conference on Artificial Intelligence*. pp5339–5347. Vol. 37.

[399] de Souza VC, Goliatt L, Capriles Goliatt PVZ. (2017) Clustering algorithms applied on analysis of protein molecular dynamics. In *2017 IEEE Latin American Conference on Computational Intelligence (LA-CCI)*. IEEE, pp1–6.

[400] Kaur Bijral R, Manhas J, Sharma V. (2022) Hierarchical clustering based characterization of protein database using molecular dynamic simulation. In *Recent Innovations in Computing: Proceedings of ICRIC 2021, Volume 1*. Springer, pp427–437.

[401] Amiri Souri E, Chenoweth A, Karagiannis SN, et al. (2023) Drug repurposing and prediction of multiple interaction types via graph embedding. *BMC bioinformatics*, 24, 202. doi: 10.1186/s12859-023-05317-w PMC10190044 37193964

[402] Huang K, Xiao C, Glass LM et al. (2020) Skipgnn: predicting molecular interactions with skip-graph networks. *Scientific reports*, 10, 21092. doi: 10.1038/s41598-020-77766-9 PMC7713130 33273494

[403] Guo Z-H, You Z-H, Hai-Cheng Y (2020) Integrative construction and analysis of molecular association network in human cells by fusing node attribute and behavior information. *Molecular Therapy-Nucleic Acids*, 19, 498–506. doi: 10.1016/j.omtn.2019.10.046 31923739 PMC6951835

[404] Akbar S, Hayat M, Tahir M et al. (2022) cacp-deepgram: classification of anticancer peptides via deep neural network and skip-gram-based word embedding model. *Artificial Intelligence in medicine*, 131, 1–8. doi: 10.1016/j.artmed.2022.102349 36100346

[405] Hamid M-N, Friedberg I (2019) Identifying antimicrobial peptides using word embedding with deep recurrent neural networks. *Bioinformatics*, 35, 2009–2016. doi: 10.1093/bioinformatics/bty937 30418485 PMC6581433

[406] Qiu W, Zhe L, Xiao X et al. (2021) Emcbow-gpcr: a method for identifying g-protein coupled receptors based on word embedding and wordbooks. *Computational and Structural Biotechnology Journal*, 19, 4961–4969. doi: 10.1016/j.csbj.2021.08.044 34527200 PMC8437786

[407] Gavali S, Ross K, Chen C et al. (2022) A knowledge graph representation learning approach to predict novel kinase–substrate interactions. *Molecular omics*, 18, 853–864. doi: 10.1039/D1MO00521 35975455 PMC9621340

[408] Khaerul Naim M, Rajab Mengko T, Hertadi R et al. (2023) Embedcaps-dbp: predicting dna-binding proteins using protein sequence embedding and capsule network. *IEEE Access*. 10.1016/j.compbiomed.2023.107241 37437362

[409] Verkuil R, Kabeli O, Yilun D et al. (2022) Language models generalize beyond natural proteins. *BioRxiv*, 2022–12.

[410] Hwang Y, Cornman AL, Kellogg EH et al. (2024) Genomic language model predicts protein co-regulation and function. *Nature communications*, 15, 2880. doi: 10.1038/s41467-024-46947-9 PMC10991518 38570504

[411] Abdine H, Chatzianastasis M, Bouyioukos C, et al. (2024) Prot2text: multimodal protein’s function generation with gnns and transformers. In *Proceedings of the AAAI Conference on Artificial Intelligence*. pp10757–10765. Vol. 38.

[412] Shin I, Kang K, Kim J et al. (2023) Aptatrans: a deep neural network for predicting aptamer-protein interaction using pretrained encoders. *BMC bioinformatics*, 24, 447. doi: 10.1186/s12859-023-05577-6 PMC10680337 38012571

[413] Abdin O, Nim S, Wen H, et al. (2022) Pepnn: a deep attention model for the identification of peptide binding sites. *Communications biology*, 5, 503. doi: 10.1038/s42003-022-03445-2 PMC9135736 35618814

[414] Yuan Q, Chen S, Wang Y et al. (2022) Alignment-free metal ion-binding site prediction from protein sequence through pretrained language model and multi-task learning. *Briefings in bioinformatics*, 23, bbac444. doi: 10.1093/bib/bbac444 36274238

[415] Zhongshen L, Jin J, Wang Y et al. (2023) Example: explainable deep learning framework for the prediction of plant small secreted peptides. *Bioinformatics*, 39, btad108. doi: 10.1093/bioinformatics/btad108 PMC10027287 36897030

[416] Zhou H, Xuefei L, Yao W et al. (2019) Improving neural protein–protein interaction extraction with knowledge selection. *Computational Biology and chemistry*, 83, 1–9. doi: 10.1016/j.compbiolchem.2019.107146 31707129

[417] Zhang R, Wang Z, Wang X et al. (2023) Mhtan-dti: metapath-based hierarchical transformer and attention network for drug–target interaction prediction. *Briefings in Bioinformatics*, 24, bbad079. doi: 10.1093/bib/bbad079 36892155

[418] Zhang T-H, Musaddaqul Hasib M, Chiu Y-C et al. (2022) Transformer for gene expression modeling (t-gem): an interpretable deep learning model for gene expression-based phenotype predictions. *Cancers*, 14, 4763. doi: 10.3390/cancers14194763 PMC9562172 36230685

[419] Wang F, Wang H, Wang L et al. (2022) Mhcroberta: pan-specific peptide–mhc class i binding prediction through transfer learning with label-agnostic protein sequences. *Briefings in Bioinformatics*, 23, bbab595. doi: 10.1093/bib/bbab595 35443027

[420] Weber L, Sänger M, Garda S et al. (2022) Chemical–protein relation extraction with ensembles of carefully tuned pretrained language models. *Database* 2022, baac098. doi: 10.1093/database/baac098 PMC9674024 36399413

[421] Kang H, Goo S, Lee H et al. (2022) Fine-tuning of bert model to accurately predict drug–target interactions. *Pharmaceutics*, 14, 1710. doi: 10.3390/pharmaceutics14081710 PMC9414546 36015336

[422] Prihoda D, Maamary J, Waight A, Juan V, Fayadat-Dilman L, Svozil D, and Bitton DA. Biophi: a platform for antibody design, humanization, and humanness evaluation based on natural antibody repertoires and deep learning. In *MAbs*, 14, 2020203. Taylor & Francis, 2022. 10.1080/19420862.2021.2020203 PMC8837241 35133949

[423] Yamaguchi S, Nakashima H, Moriwaki Y et al. (2022) Prediction of protein mononucleotide binding sites using alphafold2 and machine learning. *Computational Biology and Chemistry*, 100, 1–12. doi: 10.1016/j.compbiolchem.2022.107744 35933804

[424] Yuan Q, Chen S, Rao J et al. (2022) Alphafold2-aware protein–dna binding site prediction using graph transformer. *Briefings in Bioinformatics*, 23, bbab564. doi: 10.1093/bib/bbab564 35039821

[425] Liu Z, Pan W, Li W et al. (2022) Evaluation of the effectiveness of derived features of alphafold2 on single-sequence protein binding site prediction. *Biology*, 11, 1454. doi: 10.3390/biology11101454 PMC9598995 36290358

[426] Kalakoti Y, Yadav S, Sundar D (2022) Transdti: transformer-based language models for estimating dtis and building a drug recommendation workflow. *ACS omega*, 7, 2706–2717. doi: 10.1021/acsomega.1c05203 35097268 PMC8792915

[427] Leem J, Mitchell LS, Farmery JHR et al. (2022) Deciphering the language of antibodies using self-supervised learning. *Patterns*, 3, 1–12. doi: 10.1016/j.patter.2022.100513 PMC9278498 35845836

[428] Sun C, Yang Z, Leilei S et al. (2020) Chemical–protein interaction extraction via Gaussian probability distribution and external biomedical knowledge. *Bioinformatics*, 36, 4323–4330. doi: 10.1093/bioinformatics/btaa491 32399565

[429] Duong D, Uppunda A, Gai L et al. (2019) Evaluating representations for gene ontology terms. *biorxiv*, 765644.

[430] Zhang J, Wang M, Yao H (2024) Accurate tcr-pmhc interaction prediction using a bert-based transfer learning method. *Briefings in Bioinformatics*, 25, bbad436. doi: 10.1093/bib/bbad436 PMC10783865 38040492

[431] Wang Y, Zhang S, Zhang Y et al. (2020) Extracting protein–protein interactions affected by mutations via auxiliary task and domain pre-trained model. In *2020 IEEE International Conference on Bioinformatics and Biomedicine (BIBM)*. IEEE, pp495–498.

[432] Cheng J, Bendjama K, Rittner K, et al. (2021) Bertmhc: improved mhc–peptide class ii interaction prediction with transformer and multiple instance learning. *Bioinformatics*, 37, 4172–4179. doi: 10.1093/bioinformatics/btab422 34096999 PMC9502151

[433] Huang Y, Huang H-Y, Chen Y, et al. (2023) A robust drug–target interaction prediction framework with capsule network and transfer learning. *International Journal of Molecular Sciences*, 24, 14061. doi: 10.3390/ijms241814061 PMC10531393 37762364

[434] Zheng J, Xiao X, Qiu W-R (2022) Dti-bert: identifying drug-target interactions in cellular networking based on bert and deep learning method. *Frontiers in Genetics*, 13, 1–12. doi: 10.3389/fgene.2022.859188 PMC9213727 35754843

[435] Sun M, Haoyuan H, Pang W, et al. (2023) Acp-bc: a model for accurate identification of anticancer peptides based on fusion features of bidirectional long short-term memory and chemically derived information. *International Journal of Molecular Sciences*, 24, 15447. doi: 10.3390/ijms242015447 PMC10607064 37895128

[436] Qiao Y, Zhu X, Gong H (2022) Bert-kcr: prediction of lysine crotonylation sites by a transfer learning method with pre-trained bert models. *Bioinformatics*, 38, 648–654. doi: 10.1093/bioinformatics/btab712 34643684

[437] Morteza Pourreza Shahri and Indika Kahanda (2021) Deep semi-supervised learning ensemble framework for classifying co-mentions of human proteins and phenotypes. *BMC bioinformatics*, 22, 1–22. doi: 10.1186/s12859-021-04421-z 34656098 PMC8520253

[438] Teufel F, Juan Almagro Armenteros J, Rosenberg Johansen A et al. (2022) Signalp 6.0 predicts all five types of signal peptides using protein language models. *Nature biotechnology*, 40, 1023–1025. doi: 10.1038/s41587-021-01156-3 PMC9287161 34980915

[439] Muazzam Ali Shah S, Yu-Yen O (2021) Trp-bert: discrimination of transient receptor potential (trp) channels using contextual representations from deep bidirectional transformer based on bert. *Computers in Biology and Medicine*, 137, 1–11. doi: 10.1016/j.compbiomed.2021.104821 34508974

[440] Littmann M, Heinzinger M, Dallago C et al. (2021) Protein embeddings and deep learning predict binding residues for various ligand classes. *Scientific Reports*, 11, 23916. doi: 10.1038/s41598-021-03431-4 PMC8668950 34903827

[441] Hai Dang T, Tien Anh V (2023) Sequence-based protein–protein interaction prediction using multi-kernel deep convolutional neural networks with protein language model. *bioRxiv*, 2023–10. 10.1186/s12859-024-05725-6 PMC10924985 38461247

[442] Chen J, Zhonghui G, Youjun X et al. (2023) Quotetarget: a sequence-based transformer protein language model to identify potentially druggable protein targets. *Protein Science*, 32, e4555. doi: 10.1002/pro.4555 PMC9878469 36564866

[443] Rao R, Meier J, Sercu T et al. (2020) Transformer protein language models are unsupervised structure learners. *Biorxiv*, 2020–12.

[444] Brandes N, Goldman G, Wang CH et al. (2023) Genome-wide prediction of disease variant effects with a deep protein language model. *Nature Genetics*, 55, 1512–1522. doi: 10.1038/s41588-023-01465-0 37563329 PMC10484790

[445] Ferruz N, Schmidt S, Höcker B (2022) Protgpt2 is a deep unsupervised language model for protein design. *Nature communications*, 13, 4348. doi: 10.1038/s41467-022-32007-7 PMC9329459 35896542

[446] Elnaggar Ahmed MH, Dallago C, Rihawi G, et al. (2020) Prottrans: towards cracking the language of life’s code through self-supervised deep learning and high performance computing. *bioRxiv*.

[447] Lee J, Yoon W, Kim S et al. (2020) Biobert: a pre-trained biomedical language representation model for biomedical text mining. *Bioinformatics*, 36, 1234–1240. doi: 10.1093/bioinformatics/btz682 31501885 PMC7703786

[448] Chithrananda S, Grand G, Ramsundar B (2020) Chemberta: large-scale self-supervised pretraining for molecular property prediction. *ArXiv Preprint arXiv:2010.09885*.

[449] Zhang Y, Lin J, Zhao L et al. (2021) A novel antibacterial peptide recognition algorithm based on bert. *Briefings in bioinformatics*, 22, bbab200. doi: 10.1093/bib/bbab200 34037687

[450] Ingraham J, Garg V, Barzilay R, et al. (2019) 33rd Conference on Neural Information Processing Systems (NeurIPS 2019). Generative models for graph-based protein design. *Advances in Neural Information Processing systems*, 32.doi: 10.5555/3454287.3455704

[451] Peters ME, Neumann M, Iyyer M et al. (2018) Deep contextualized word representations. arxiv: 180205365. *arXiv*.

[452] Zhang T, Jia J, Chen C et al. (2023) Bigrud-sa: protein s-sulfenylation sites prediction based on bigru and self-attention. *Computers in Biology and Medicine*, 163, 1–9. doi: 10.1016/j.compbiomed.2023.107145 37336062

[453] Wang M, Wang J, Rong Z, et al. (2024) A bidirectional interpretable compound-protein interaction prediction framework based on cross attention. *Computers in Biology and Medicine*, 172, 108239. doi: 10.1016/j.compbiomed.2024.108239 38460309

[454] Duan T, Kuang Z, Wang J, et al. (2021) Gbdtlrl2d predicts lncrna–disease associations using metagraph2vec and k-means based on heterogeneous network. *Frontiers in Cell and Developmental Biology*, 9, 1–15. doi: 10.3389/fcell.2021.753027 PMC8718797 34977011

[455] Zhou J-R, You Z-H, Cheng L, et al. (2021) Prediction of lncrna-disease associations via an embedding learning hope in heterogeneous information networks. *Molecular Therapy-Nucleic Acids*, 23, 277–285. doi: 10.1016/j.omtn.2020.10.040 33425486 PMC7773765

[456] Al Taweraqi N, King RD (2022) Improved prediction of gene expression through integrating cell signalling models with machine learning. *BMC bioinformatics*, 23, 323. doi: 10.1186/s12859-022-04787-8 PMC9356471 35933367

[457] Tian Z, Han C, Lewen X et al. (2024) Mgcnss: mirna–disease association prediction with multi-layer graph convolution and distance-based negative sample selection strategy. *Briefings in Bioinformatics*, 25, bbae168. doi: 10.1093/bib/bbae168 PMC11018511 38622356

[458] Zhongxing X, Wang X, Meng J et al. (2023) m5u-gepred: prediction of rna 5-methyluridine sites based on sequence-derived and graph embedding features. *Frontiers in microbiology*, 14, 1–11. doi: 10.3389/fmicb.2023.1277099 PMC10627201 37937221

[459] Wang Y, Tai S, Zhang S et al. (2023) Promger: promoter prediction based on graph embedding and ensemble learning for eukaryotic sequence. *Genes*, 14, 1441. doi: 10.3390/genes14071441 PMC10379012 37510345

[460] Zhou L, Peng X, Zeng L, et al. (2024) Finding potential lncrna–disease associations using a boosting-based ensemble learning model. *Frontiers in Genetics*, 15, 1–13. doi: 10.3389/fgene.2024.1356205 PMC10940470 38495672

[461] Jianwei L, Jianing L, Kong M et al. (2021) Svdnvlda: predicting lncrna-disease associations by singular value decomposition and node2vec. *BMC bioinformatics*, 22, 1–18. doi: 10.1186/s12859-021-04457-1 34727886 PMC8561941

[462] Narayanan S, Ramachandran A, Aakur SN, et al. (2020) Genome sequence classification for animal diagnostics with graph representations and deep neural networks. *ArXiv Preprint arXiv:2007.12791*.

[463] Dai Z, Deng F (2023) Lncpndeep: a long non-coding rna classifier based on large language model with peptide and nucleotide embedding. *bioRxiv*, 2023–11. 10.1016/j.ncrna.2026.06.004 PMC13488033 42621896

[464] Zeng W, Gautam A, Huson DH (2023) Mulan-methyl—multiple transformer-based language models for accurate dna methylation prediction. *GigaScience*, 12, giad054. doi: 10.1093/gigascience/giad054 PMC10367125 37489753

[465] Weizhi A, Guo Y, Bian Y et al. (2022) Modna: motif-oriented pre-training for DNA language model. In *Proceedings of the 13th ACM International Conference on Bioinformatics, Computational Biology and Health Informatics*. pp1–5.

[466] Zou H, Boya J, Zhang M et al. (2024) Mhgtmda: molecular heterogeneous graph transformer based on biological entity graph for mirna-disease associations prediction. *Molecular Therapy-Nucleic Acids*, 35, 1–9. doi: 10.1016/j.omtn.2024.102139 PMC10879798 38384447

[467] Clauwaert J, Waegeman W (2020) Novel transformer networks for improved sequence labeling in genomics. *IEEE/ACM Transactions on Computational Biology and Bioinformatics*, 19, 97–106. doi: 10.1109/TCBB.2020.3035021 33125335

[468] Mehmood F, Arshad S, Shoaib M (2024) Adh-enhancer: an attention-based deep hybrid framework for enhancer identification and strength prediction. *Briefings in Bioinformatics*, 25, bbae030. doi: 10.1093/bib/bbae030 PMC10885011 38385876

[469] Martin Navarez A Roxas R. An evaluation of multitask transfer learning methods in identifying 6ma and 5mc methylation sites of rice and maize. *Available at SSRN 4178244*.

